# A Comprehensive Approach to Derivatization: Elemental Composition, Biochemical, and In Silico Studies of Metformin Derivatives Containing Copper and Zinc Complexes

**DOI:** 10.3390/molecules28031406

**Published:** 2023-02-01

**Authors:** Javed Ahmed, Mohsin Abbas Khan, Muhammad Ehsan Khalid, Irshad Ahmad, Irfan Pervaiz, Umair Khurshid, Saharish Khaliq, Kashif ur Rehman Khan, Muhammad Adeel Arshad, Ghadeer M. Albadrani, Ahmed E. Altyar, Amany A. Sayed, Mousa O. Germoush, Mohamed M. Abdel-Daim

**Affiliations:** 1 Department of Pharmaceutical Chemistry, Faculty of Pharmacy, The Islamia University of Bahawalpur, Bahawalpur 63100, Pakistan; 2 Frontier Medical and Dental College, Abbottabad 22020, Pakistan; 3 KITAAS, Khaldunia College of Pharmacy, Lahore 54000, Pakistan; 4 Institute of Pharmacy, Faculty of Pharmaceutical and Allied Health Sciences, Lahore College for Women University, Lahore 54000, Pakistan; 5 Department of Biology, College of Science, Princess Nourah bint Abdulrahman University, P.O. Box 84428, Riyadh 11671, Saudi Arabia; 6 Department of Pharmacy Practice, Faculty of Pharmacy, King Abdulaziz University, P.O. Box 80260, Jeddah 21589, Saudi Arabia; 7 Pharmacy Program, Batterjee Medical College, P.O. Box 6231, Jeddah 21442, Saudi Arabia; 8 Zoology Department, Faculty of Science, Cairo University, Giza 12613, Egypt; 9 Biology Department, College of Science, Jouf University, P.O. Box 2014, Sakaka 72388, Saudi Arabia; 10 Department of Pharmaceutical Sciences, Pharmacy Program, Batterjee Medical College, P.O. Box 6231, Jeddah 21442, Saudi Arabia; 11 Pharmacology Department, Faculty of Veterinary Medicine, Suez Canal University, Ismailia 41522, Egypt

**Keywords:** copper, zinc, metformin, FTIR, UV, NMR, enzyme inhibition assay, amylase, glucosidase, molecular simulation, docking

## Abstract

The current study was designed to synthesize, characterize, and screen the molecular and biological activities of different metformin derivatives that possess potent antidiabetic potential with minimal side-effects. Metformin-based derivatives containing the metal complexes Cu II (MCu1–MCu9) and Zn II (MZn1–MZn9) were generated using aromatic aldehydes and ketones in a template process. The novel metal complexes were characterized through elemental analysis, physical state, melting point, physical appearance, Fourier-transform infrared (FTIR) spectroscopy, UV/visible (UV/Vis) spectroscopy, ^1^H nuclear magnetic resonance (NMR) spectroscopy, and ^13^C-NMR spectroscopy. Screening for inhibitory activity against the enzymes α-amylase and α-glucosidase, and molecular simulations performed in Schrödinger were used to assess the synthesized derivatives’ biological potential. Met1, Met2, Met3, and Met8 all displayed activities that were on par with the reference in an enzymatic inhibition assay (amylase and glucosidase). The enzyme inhibition assay was corroborated by molecular simulation studies, which also revealed a competitive docking score compared to the gold standard. The Swiss ADME online web server was utilized to compute ADME properties of metformin analogues. Lipinski’s rule of five held true across all derivatives, making it possible to determine the percentage of absorption. Metformin derivatives showed significant antidiabetic activities against both targeted enzymes, and the results of this work suggest that these compounds could serve as lead molecules for future study and development.

## 1. Introduction

In recent decades, Schiff bases have shown their importance in the industrial and pharmacological fields due to their novel properties. Derivatives of Schiff bases are also important in studying coordination chemistry. Synthesis of novel Schiff bases is of prime interest to scientists in the discovery of medicinal drugs [1,2]. Metformin is an oral drug used to reduce blood glucose levels in patients suffering from non-insulin-dependent diabetes mellitus (NIDDM) [3]. It improves insulin sensitivity, thereby lowering the resistance to insulin that is common in NIDDM [4]. Its history is mainly linked with a herbal plant (*Galega officinalis* also known as goat’s rue) that is native to European plants and mainly used in lowering blood glucose levels; the plant extract is rich in guanidine. Different derivatives of guanidine (other than metformin) were mainly used between the 1930s as antidiabetic agents but discontinued in the market due to their toxic effects toward vital organs and the invention of insulin [5]. Therapeutic approaches to control hyperglycemia include increasing the level of insulin or decreasing insulin resistance [6]. Another pharmacological approach that is implemented in the clinics to manage postprandial hyperglycemia is the inhibition of the α-glucosidase enzyme [7]. However, some undesirable side-effects are also associated with the prolonged use of these inhibitors such as gastrointestinal side-effects, bloating, and diarrhea. Alternatives with fewer side-effects and cost-effective treatments are desired for the treatment of diabetes [8,9]. In the early 1940s, metformin was discovered and used as an antimalarial and to treat influenza, although it was observed during testing that it also causes a reduction in blood glucose levels. The antidiabetic use of metformin was first time reported by Jean Steme (a French physician) in 1957 [10].

The field of metallo-pharmaceutics has received extensive attention recently. This background has led to the development of a wide range of metal-containing drugs that exhibit notable antibacterial, anticancer, and antidiabetic properties [11,12,13]. Many researchers have synthesized different derivatives of metformin due to the increased glucose-lowering effect among others. Schiff bases are versatile and of a plastic nature; they form the majority of the ligand complexes and show a wide variety of biological effects. Metal complex chemistry containing Schiff-based ligands formed by aldehydes/ketones with amines exhibits significant biological effects, expressing the same features as metalloporphyrin with respect to the electronic and catalytic effects that mimic enzymatic effects. In the past few decades, the catalytic activity of metal complexes has been highlighted [14]. In the past few years, Schiff base metal complexes of metformin have revealed anticancer effects [15]. The strongest correlation with diabetes and cancer has been observed in breast, pancreas, and endometrial tumors [16]. Copper complexes of metformin have been synthesized, showing great levels of antidiabetic and antioxidant defense effects [17,18]. Various metals such as copper and zinc have been used to form metal complexes with metformin [19,20]. In this study, we generated different copper and zinc metal complexes with metformin-based derivatives synthesized using an aromatic aldehyde and ketone through a template reaction [21].

## 2. Results

### 2.1. Characterization of Metabolites

The characterization schemes of all the metformin metabolites and metal complexes are presented in Schemes S1–S3 (Supplementary Materials). While, IR and NMR spectras are shown in Figures S7–S31 (Supplementary Materials).

#### 2.1.1. Characterization of (Met1–Met9)

Metformin; IUPAC name: *N*,*N*-dimethyltriimidodicarbonic diamide; M.P. 222–226 °C; molecular formula: C_4_H_11_N_5_; molecular weight: 129.16 g/mol. Elemental analysis for C_4_H_11_N_5_: (calculated) 37.20; H, 8.58; N, 54.22; (found) C, 37.16; H, 8.65; N, 54.28. FT-IR ν (cm^−1^), 2971 (C–H, s, stretching), 1620 (C=N, m, bending), 1153 (C–N, m, bending), 3368, 3245 (N–H, s, stretching). ^1^H-NMR (DMSO, ppm) δ: 3.43 s, (6H, CH_3_), 1.17 s (1H), 1.19 s (1H), 1.21 s (1H), (NH), 8.15 s, (2H, NH_2_). ^13^C-NMR (DMSO, ppm) δ: 45.2 (C1), 45.2 (C2), 161.6 (C3), 150.3 (C4).

Met1; IUPAC name: *N*′-benzylidene-*N*,*N*-dimethyltriimidodicarbonic diamide; yield (65%); M.P. 160–161 °C; molecular formula: C_11_H_15_N_5_; molecular weight: 217.27 g/mol. Elemental analysis for C_11_H_15_N_5_: (calculated) C, 60.81; H, 6.96; N, 32.23; (found) C, 60.75; H, 7.05; N, 32.28. FT-IR ν (cm^−1^), 2955, 2922, 2854 (C–H, s, stretching), 1627 (C=N, m, bending), 1593, 1463 (CH=CH, w, bending), 1130 (C–N, m, bending), 3372, 3264 (N–H, s, stretching). ^1^H-NMR (DMSO, ppm) δ: 3.30 s (6H, CH_3_), 1.16 s (1H), 1.18 s (1H) 1.19 s (1H), (NH), 8.10 s (1H, CH), 7.87–7.89 d (2H, CH), 7.40–7.44 m, (3H, CH). ^13^C-NMR (DMSO, ppm) δ: 45.8 (C1,2), 150.4 (C3,4), 148.8 (C5), 131.7 (C6), 128.8 (C7,11), 128.2 (C8,10), 130.7 (C9). 

Met2; IUPAC name: *N*′-[(2-hydroxyphenyl)methylidene]-*N*,*N*-dimethyltriimidodicarbonic diamide; yield (61%); M.P. 175–176 °C; molecular formula: C_11_H_15_N_5_O; molecular weight: 233.27 g/mol. Elemental analysis for C_11_H_15_N_5_O: (calculated) C, 56.64; H, 6.48; N, 30.02; (found) C, 56.70; H, 6.41; N, 30.12. FT-IR ν (cm^−1^), 3066, 2984 (C–H, s, stretching), 1624 (C=N, m, bending), 1593, 1474 (CH=CH, w, bending), 1168 (C–N, m, bending), 1273 (C–O, s, bending), 3344, 3258 (N–H, s, stretching). ^1^H-NMR (DMSO, ppm) δ: 3.45 s (6H, CH_3_), 2.41, 2.51 s (3H, NH), 8.17 s (1H, CH), 7.46–7.47 d (1H, CH), 7.45–7.46 d (2H, CH), 7.87–7.88 t (1H, CH), 5.10 s (1H, OH). ^13^C-NMR (DMSO, ppm) δ: 45.6 (C1,2), 150.1 (C3), 156.9 (C4), 162.5 (C5), 121.9 (C6), 129.8 (C7), 128.1 (C8), 128.7 (C9), 126.5 (C10), 149.4 (C11).

Met3: IUPAC name: *N*,*N*-dimethyl-*N*′-[(1Z,2E)-3-phenylprop-2-en-1-ylidene]triimidodicarbonic diamide; yield (70%); M.P. 145–146 °C; molecular formula: C_13_H_17_N_5_; molecular weight: 243.31 g/mol. Elemental analysis for C_13_H_17_N_5_: (calculated) C, 64.17; H, 7.04; N, 28.78; (found) C, 64.24; H, 7.00; N, 28.75. FT-IR ν (cm^−1^), 1645 (C=N, m, bending), 1601, 1488 (CH=CH, w, bending), 1135 (C–N, m, bending), 3312 (N–H, s, stretching). ^1^H-NMR (DMSO, ppm) δ: 3.17s (6H, CH_3_), 3.72 s (3H), 7.86–7.88 d (1H, CH), 6.34–6.35 d (1H, CH), 7.36–7.38 d (1H, CH), 7.40–7.41 d (2H, CH), 7.41–7.43 t (2H, CH), 7.45–7.46 t (1H, CH). ^13^C-NMR (DMSO, ppm) δ: 46.6 (C1,2), 153.7 (C3), 161.2 (C4), 162.4.7 (C5), 127.9 (C6), 136.0 (C7), 136.3 (C8), 135.8 (C9), 128.9 (C10), 128.3 (C11), 128.6 (C12), 135.7 (C13).

Met4; IUPAC name: *N*,*N*-dimethyl-*N*′-methylidenetriimidodicarbonic diamide; yield (58%); M.P. 186–187 °C; molecular formula: C_5_H_11_N_5_; molecular weight: 141.17 g/mol. Elemental analysis for C_5_H_11_N_5_: (calculated) C, 42.54; H, 7.85; N, 49.61; (found) C, 42.45; H, 7.83; N, 49.70. FT-IR ν (cm^−1^), 2956, 2871 (C–H, s, stretching), 1651 (C=N, m, bending), 1147 (C–N, m, bending), 3353, 3259 (N–H, s, stretching). ^1^H-NMR (DMSO, ppm) δ: 3.44s (6H, CH_3_), 1.17 s, 7.45 s 7.48 s (3H), 7.88 s (2H, CH_2_). ^13^C-NMR (DMSO, ppm) δ: 45.6 (C1,2), 150.3 (C3), 161.4 (C4), 151.0 (C5).

Met5; IUPAC name: *N*,*N*-dimethyl-*N*′-(propan-2-ylidene)triimidodicarbonic diamide; yield (66%); M.P. 152–153 °C; molecular formula: C_7_H_15_N_5_; molecular weight: 169.23 g/mol. Elemental analysis for C_7_H_15_N_5_: (calculated) C, 49.68; H, 8.93; N, 41.38; (found) C, 49.65; H, 8.90; N, 41.44. FT-IR ν (cm^−1^), 3075 (C–H, s, stretching), 1615 (C=N, m, bending), 1145 (C–N, m, bending), 3291 (N–H, s, stretching). ^1^H-NMR (DMSO, ppm) δ: 3.39 s (6H, CH_3_), 1.19 s, 7.78 s, 7.79 s (3H), 1.15 s, 1.17s (6H, CH_3_). ^13^C-NMR (DMSO, ppm) δ: 45.6 (C1,2), 144.9 (C3), 147.7 (C4), 157.2 (C5), 13.1 (C6), 9.6 (C7).

Met6; IUPAC name: *N*,*N*-dimethyl-*N*′-[(1E)-1-phenylethylidene]triimidodicarbonic diamide; yield (68%); M.P. 174–175 °C; molecular formula: C_12_H_17_N_5_; molecular weight: 231.30 g/mol. Elemental analysis for C_12_H_17_N_5_: (calculated) C, 62.31; H, 7.41; N, 30.28; (found) C, 62.31; H, 7.41; N, 30.28. FT-IR ν (cm^−1^), 2978 (C–H, s, stretching), 1620 (C=N, m, bending), 1601, 1480 (C=C, m, bending), 1146 (C–N, m, bending). ^1^H-NMR (DMSO, ppm) δ: 1.31 s, 2.39 s (9H, CH_3_), 6.62 s (3H, NH), 7.12 d (2H, CH), 7.52 m (3H, CH). ^13^C-NMR (DMSO, ppm) δ: 55.8 (C1), 59.9 (C2), 152.8 (C3), 156.4 (C4), 163.5 (C5), 136.5 (C6), 127.5 (C7,11), 134.8 (C8,10), 136.0 (C9), 32.3 (C12).

Met7; IUPAC name: *N*′-(diphenylmethylidene)-*N*,*N*-dimethyltriimidodicarbonic diamide; yield (74%); M.P. 191–192 °C; molecular formula: C_17_H_19_N_5_; molecular weight: 293.37 g/mol. Elemental analysis for C_17_H_19_N_5_: (calculated) C, 69.60; H, 6.53; N, 23.87; (found) C, 69.55; H, 6.63; N, 23.82. FT-IR ν (cm^−1^), 3103 (C–H, s, stretching), 1615 (C=N, m, bending), 1585 (C=C, m, bending), 1134 (C–N, m, bending), 3268 (N–H, s, stretching). ^1^H-NMR (DMSO, ppm) δ: 3.03 s, 3.05 s, (6H, CH_3_), 3.09s (3H, NH), 7.33–7.35 d, 7.38–7.42 m, (5H, CH), 7.93–7.94 d, 7.95–7.96 m (5H, CH). ^13^C-NMR (DMSO, ppm) δ: 45.5 (C1,2), 150.1 (C3), 156.9 (C4), 162.5 (C5), 149.4 (C6), 137.8 (C7,13), 129.8 (C8,14), 128.7 (C9,15), 135.9 (C10,16), 128.3 (C11,17), 129.7 (C12).

Met8; IUPAC name: *N*′-[(E)-(4-hydroxy-3-methoxyphenyl)methylidene]-*N*,*N*-dimethyltriimidodicarbonic diamide; yield (60%); M.P. 170–171 °C; molecular formula: C_12_H_17_N_5_O_2_; molecular weight: 263.30 g/mol. Elemental analysis for C_12_H_17_N_5_O_2_: (calculated) C, 54.74; H, 6.51; N, 26.60; (found) C, 54.84; H, 6.45; N, 26.55. FT-IR ν (cm^−1^), 1680, 1642 (C=N, m, bending), 1590, 1474 (C=C, m, bending), 1138 (C–N, m, bending), 3407, 3321 (N–H, s, stretching). ^1^H-NMR (DMSO, ppm) δ: 2.15 s, 3.39 s (9H, CH_3_), 8.12 s(1H, CH), 1.68 s (3H, NH), 6.91 d, 7.12 d, 7.45 s (3H, CH), 4.92 s (1H, OH). ^13^C-NMR (DMSO, ppm) δ: 55.5 (C1), 54.9 (C2), 156.8 (C3), 159.4 (C4), 163.5 (C5), 127.2 (C6), 117.5 (C7), 145.6 (C8), 146.0 (C9), 112.3 (C10), 119.7 (C11), 45.2 (C12).

Met9; IUPAC name: *N*′-[(1Z)-2-hydroxy-1,2-diphenylethylidene]-*N*,*N*-dimethyltriimidodicarbonic diamide; yield (78%); M.P. 139–140 °C; molecular formula: C_18_H_21_N_5_O; molecular weight: 323.39 g/mol. Elemental analysis for C_18_H_21_N_5_O: (calculated) C, 66.85; H, 6.55; N, 21.66; (found) C, 66.82; H, 6.65; N, 21.61; FT-IR ν (cm^−1^), 3054, 2922 (C–H, s, stretching), 1647 (C=N, m, bending), 1588, 1481 (C=C, m, bending), 1157 (C–N, m, bending), 3301 (N–H, s, stretching). ^1^H-NMR (DMSO, ppm) δ: 2.21s (6H, CH_3_), 1.92 s (3H, NH), 4.5 s (1H, CH), 7.32–7.36 m(5H, CH), 7.82–7.83 d, 7.48–7.49 m (5H, CH), 3.52 3 (1H, OH). ^13^C-NMR (DMSO, ppm) δ: 56.7(C1), 57.2 (C2), 158.8 (C3), 160.2 (C4), 162.5 (C5), 61.5 (C6), 138.5 (C7), 126.8 (C8,12), 1127.8 (C9,11), 128.3 (C10), 132.3 (C13), 127.5(C14,18), 128.2 (C15,17), 130.5 (C16).

#### 2.1.2. Characterization of (MCu1–MCu9)

MCu1; yield (68%); M.P. 250 °C; molecular formula: C_22_H_40_CuN_10_O_9_S; molecular weight: 684.23 g/mol. Elemental analysis for C_22_H_40_CuN_10_O_9_S: (calculated) C, 38.62; H, 5.89; N, 20.47; S, 4.69; (found) C, 38.66; H, 5.93; N, 20.49; S, 4.71. FT-IR ν (cm^−1^), 2928, (C–H, s, stretching), 1653 (C=N, m, bending), 1601 (CH=CH, w, bending), 1178 (C–N, m, bending), 3372 (N–H, s, stretching). ^1^H-NMR (DMSO, ppm) δ: 3.01 s (12H, CH_3_), 1.18 s (2H), 1.23 s (2H), 1.16 s (2H), (NH), 7.92 s (2H, CH), 7.72–7.73 d (4H, CH), 7.41–7.42 m (6H, CH). ^13^C-NMR (DMSO, ppm) δ: 45.8 (C1,1′), 45.6 (C2,2′), 150.4 (C3,3′), 154.1 (C4,4′), 148.8 (C5,5′), 131.7 (C6,6′), 128.8 (C7,7′,11,11′), 128.2 (C8,8′,10,10′), 130.7 (C9,9′).

MCu2; yield (70%); M.P. 275–276 °C; molecular formula: C_22_H_40_CuN_10_O_11_S; molecular weight: 716.22 g/mol. Elemental analysis for C_22_H_40_CuN_10_O_11_S: (calculated) C, 36.89; H, 5.63; N, 19.56; S, 4.48; (found) C, 36.93; H, 5.60; N, 19.59; S, 4.42. FT-IR ν (cm^−1^), 3052, 2951 (C–H, s, stretching), 1645 (C=N, m, bending), 1599, 1473 (CH=CH, w, bending), 1164 (C–N), 1275 (C–O, s, bending), 3352 (N–H, s, stretching). ^1^H-NMR (DMSO, ppm) δ: 3.41 s (12H, CH_3_), 2.40, 2.58 s (6H, NH), 8.12 s (2H, CH), 7.44–7.45 d (2H, CH), 7.46–7.47 d (4H, CH), 7.81–7.82 t (2H, CH), 5.13 s (1H, OH). ^13^C-NMR (DMSO, ppm) δ: 45.5 (C1,1′), 47.6 (C2,2′), 150.2 (C3,3′), 156.6 (C4,4′), 162.3 (C5,5′), 121.9 (C6,6′), 129.8 (C7,7′), 128.3 (C8,8′), 128.8 (C9,9′), 126.8 (C10,10′), 149.3 (C11,11′).

MCu3; yield (65%); M.P. 221–222 °C; molecular formula: C_26_H_44_CuN_10_O_9_S; molecular weight: 736.30 g/mol. Elemental analysis for C_26_H_44_CuN_10_O_9_S: (calculated) C, 42.41; H, 6.02; N, 19.02; S, 4.35; (found) C, 42.48; H, 6.07; N, 19.06; S, 4.31. FT-IR ν (cm^−1^), 3025, 2968 (C–H, s, stretching), 1653 (C=N, m, bending), 1161, 1483 (CH=CH, w, bending), 1170 (C–N, m, bending), 3330 (N–H, s, stretching). ^1^H-NMR (DMSO, ppm) δ: 3.11 s (12H, CH_3_), 3.32 s (6H), 7.81–7.82 d (2H, CH), 6.31–6.32 d (2H, CH), 7.34–7.35 d (2H, CH), 7.42–7.43 d (4H, CH), 7.46–7.47 t (4H, CH), 7.49–7.50 t (2H, CH). ^13^C-NMR (DMSO, ppm) δ: 46.2 (C1,1′) 45.7 (C2,2′), 153.4 (C3,3′), 161.5 (C4,4′), 162.4.6 (C5,5′), 127.7 (C6,6′), 136.4 (C7,7′), 136.5 (C8,8′), 135.6 (C9,9′), 128.3 (C10,10′), 128.1 (C11,11′), 128.7 (C12,13), 135.6 (C13,13′).

MCu4; yield (55%); M.P. 201–202 °C; molecular formula: C_10_H_32_CuN_10_O_9_S; molecular weight: 532.03 g/mol. Elemental analysis for C_10_H_32_CuN_10_O_9_S: (calculated) C, 22.58; H, 6.06; N, 26.33; S, 6.03; (found) C, 22.62; H, 6.10; N, 26.38; S, 6.07. FT-IR ν (cm^−1^), 2951, 2892 (C–H, s, stretching), 1657 (C=N, m, bending), 1153 (C–N, m, bending), 3353 (N–H, s, stretching). ^1^H-NMR (DMSO, ppm) δ: 3.41 s (12H, CH_3_), 1.11 s, 7.44 s, 7.49 s (6H), 7.84 s (4H, CH_2_). ^13^C-NMR (DMSO, ppm) δ: 45.7 (C1,1′), 47.3 (C2,2′), 150.7 (C3,3′), 161.7 (C4,4′), 151.6 (C5,5′).

MCu5; yield (70%); M.P. 252–253 °C; molecular formula: C_14_H_40_CuN_10_O_9_S; molecular weight: 588.14 g/mol. Elemental analysis for C_14_H_40_CuN_10_O_9_S: (calculated) C, 28.59; H, 6.86; N, 23.82; S, 5.45; (found) C, 28.65; H, 6.82; N, 23.85; S, 5.42. FT-IR ν (cm^−1^), 3030 (C–H, s, stretching), 1652 (C=N, m, bending), 1178 (C–N, m, bending), 3335 (N–H, s, stretching). ^1^H-NMR (DMSO, ppm) δ: 3.01 s (12H, CH_3_), 1.21 s, 7.45 s, 7.52 s (6H, NH), 1.11 s, 1.18 s (12H, CH_3_). ^13^C-NMR (DMSO, ppm) δ: 45.2 (C1,1′), 47.3 (C2,2′), 148.9 (C3,3′), 152.3 (C4,4′), 158.2 (C5,5′), 17.4 (C6,6′), 12.5 (C7,7′).

MCu6; yield (65%); M.P. 204–205 °C; molecular formula: C_24_H_44_CuN_10_O_9_S; molecular weight: 712.28 g/mol. Elemental analysis for C_24_H_44_CuN_10_O_9_S: (calculated) C, 40.47; H, 6.23; N, 19.66; S, 4.50; (found) C, 40.52; H, 6.27; N, 19.62; S, 4.45. FT-IR ν (cm^−1^), 2952 (C–H, s, stretching), 1636 (C=N, m, bending), 1599, 1473 (C=C, m, bending), 1178 (C–N, m, bending). ^1^H-NMR (DMSO, ppm) δ: 1.35 s, 2.31 s (18H, CH_3_), 6.68 s (6H, NH), 7.11–7.12 d (4H, CH), 7.53–7.54 m(6H, CH). ^13^C NMR (DMSO, ppm) δ: 55.6 (C1,1′), 59.7 (C2,2′), 152.7 (C3,3′), 156.2 (C4,4′), 163.7 (C5,5′), 136.4 (C6,6′), 127.4 (C7,7′), 134.7 (C8,8′), 136.1 (C9,9′), 134.9 (C10,10′), 126.2 (C11,11′), 25.32 (C12,12′).

MCu7; yield (62 %); M.P. 232 °C; molecular formula: C_34_H_48_CuN_10_O_9_S; molecular weight: 836.42 g/mol. Elemental analysis for C_34_H_48_CuN_10_O_9_S: (calculated) C, 48.82; H, 5.78; N, 16.75; S, 3.83; (found) C, 48.78; H, 5.81; N, 16.78; S, 3.81. FT-IR ν (cm^−1^), 3021 (C–H, s, stretching), 1635 (C=N, m, bending), 1598 (C=C, m, bending), 1175 (C–N, m, bending), 3321 (N–H, s, stretching). ^1^H-NMR (DMSO, ppm) δ: 2.92 s, 3.01 s, 3.11 s, (12H, CH_3_), 2.77s, 2.81s (6H, NH), 7.35–7.36 d, 7.39–7.40 m (10H, CH), 7.91–7.92 d, 7.97–7.98 m (10H, CH). ^13^C-NMR (DMSO, ppm) δ: 45.6 (C1,1′), 43.1 (C2,2′), 150.3 (C3,3′), 156.7 (C4,4′), 162.2 (C5,5′), 149.3 (C6,6′), 138.7 (C7,7′), 129.7 (C8,8′), 128.7 (C9,9′) 135.5 (C10,10′), 128.5 (C11,11′), 129.7 (C12,12′), 137.6 (C13,13′), 128.5 (C14, 14′), 127.6 (C15,15′), 136.4 (C16,16′), 128.1 (C17,17′).

MCu8; yield (55%); M.P. 190 °C; molecular formula: C_24_H_44_CuN_10_O_13_S; molecular weight: 776.28 g/mol. Elemental analysis for C_24_H_44_CuN_10_O_13_S: (calculated) C, 37.13; H, 5.71; N, 18.04; S, 4.13; (found) C, 37.10; H, 5.75; N, 18.10; S, 4.18. FT-IR ν (cm^−1^), 3051 (C–H, s, stretching), 1675, 1635 (C=N, m, bending), 1597, 1471 (C=C, m, bending), 1158 (C–N, m, bending), 3331 (N–H, s, stretching). ^1^H-NMR (DMSO, ppm) δ: 2.11 s, 3.21 s, 2.01 s (18H, CH_3_), 8.09 s (2H, CH), 1.62 s, 1.52s (6H, NH), 6.91–6.92 d, 7.11–7.12 d, 7.37 s (6H, CH), 4.85 s (2H, OH). ^13^C-NMR (DMSO, ppm) δ: 55.2 (C1,1′), 54.4 (C2,2′), 156.7 (C3,3′), 159.3 (C4,4′), 163.4 (C5,5′), 127.3 (C6,6′), 119.5 (C7,7′), 145.7 (C8,8′), 146.3 (C9,9′), 112.5 (C10,10′), 119.3 (C11,11′), 45.1 (C12,12′).

MCu9; yield (78%); M.P. 139–140 °C; molecular formula: C_36_H_52_CuN_10_O_11_S; molecular weight: 896.47 g/mol. Elemental analysis for C_36_H_52_CuN_10_O_11_S: (calculated) C, 48.23; H, 5.85; N, 15.62; S, 3.58; (found) C, 48.20; H, 5.88; N, 15.68; S, 3.55. FT-IR ν (cm^−1^), 3051, 2951 (C–H, s, stretching), 1653 (C=N, m, bending), 1598, 1477 (C=C, m, bending), 1177 (C–N, m, bending), 3351 (N–H, s, stretching). ^1^H-NMR (DMSO, ppm) δ: 2.12 s 1.92 s, 1.85 s, 1.96 s (12H, CH_3_), 1.25 s, 1.32 s (6H, NH), 4.45 s (2H, CH), 7.31–7.32 m, 7.36–7.37 m (10H, CH), 7.78–7.79 d, 7.45–7.46 m (10H, CH), 3.22 s (2H, OH). ^13^C-NMR (DMSO, ppm) δ: 56.5 (C1,1′), 57.1 (C2,2′), 158.7 (C3,3′), 160.5 (C4,4′), 162.4 (C5,5′), 61.4 (C6,6′), 138.7 (C7,7′), 126.7 (C8,8′), 127.9 (C9,9′), 128.1 (C10,10′), 127.5 (C11,11′), 126.3 (C12,12′) 132.4 (C13,13′), 127.1 (C14,14′), 128.2 (C15,15′), 130.3 (C16,16′), 128.7 (C17,17′), 126.9 (C18,18′).

#### 2.1.3. Characterization of (MZn1–MZn9)

MZn1; yield (55%); M.P. 250 °C; molecular formula: C_22_H_40_N_10_O_9_SZn; molecular weight: 686.06 g/mol. Elemental analysis for C_22_H_40_N_10_O_9_SZn: (calculated) C, 38.51; H, 5.88; N, 20.42; S, 4.67; (found) C, 38.47; H, 5.82; N, 20.47; S, 4.71. FT-IR ν (cm^−1^), 2951, 2854 (C–H, s, stretching), 1653 (C=N, m, bending), 1598, 1473 (CH=CH, w, bending), 1137 (C–N, m, bending), 3372, (N–H, s, stretching). ^1^H-NMR (DMSO, ppm) δ: 3.20 s (12H, CH_3_), 1.16 s (2H), 1.18 s (2H) 1.19 s (2H), (NH), 8.01 s, (2H, CH), 7.77–7.78 d, (4H, CH), 7.37–7.38 m, (6H, CH). ^13^C-NMR (DMSO, ppm) δ: 45.8 (C1,2,1′,2′), 150.4 (C3,4,3′,4′), 148.8 (C5,5′), 131.7 (C6,6′), 128.8 (C7,11,7′,11′), 128.2 (C8,10,8′,10′), 130.7 (C9,9′). 

MZn2; yield (64%); M.P. 272 °C; molecular formula: C_22_H_40_N_10_O_11_SZn; molecular weight: 718.06 g/mol. Elemental analysis for C_22_H_40_N_10_O_11_SZn: (calculated) C, 36.80; H, 5.61; N, 19.51; S, 4.47; (found) C, 36.85; H, 5.64; N, 19.45; S, 4.46. FT-IR ν (cm^−1^), 3066 (C–H, s, stretching), 1680 (C=N, m, bending), 1603, 1473 (CH=CH, w, bending), 1165 (C–N, m, bending), 1275 (C–O, s, bending), 3344 (N–H, s, stretching). ^1^H-NMR (DMSO, ppm) δ: 3.42s (12H, CH_3_), 2.43, 2.62 s (6H, NH), 7.92 s (2H, CH), 7.41–7.42 d (2H, CH), 7.44–7.45 d (4H, CH), 7.82–7.83 t (2H, CH), 5.12 s (2H, OH). ^13^C-NMR (DMSO, ppm) δ: 45.6 (C1,1′), 47.3 (C2, 2′), 150.0 (C3,3′), 156.7 (C4,4′), 162.4 (C5,5′), 121.8 (C6,6′), 129.7 (C7,7′), 128.0 (C8,8′), 128.8 (C9,9′), 126.7 (C10,10′), 149.4 (C11,11′).

MZn3; yield (50%); M.P. 246 °C; molecular formula: C_26_H_44_N_10_O_9_SZn; molecular weight: 738.13 g/mol. Elemental analysis for C_26_H_44_N_10_O_9_SZn: (calculated) C, 42.31; H, 6.01; N, 18.98; S, 4.34; (found) C, 42.31; H, 6.01; N, 18.98; S, 4.34. FT-IR ν (cm^−1^), 2925 (C–H, s, stretching), 1645 (C=N, m, bending), 1602, 1488 (CH=CH, w, bending), 1178 (C–N, m, bending), 3366 (N–H, s, stretching). ^1^H-NMR (DMSO, ppm) δ: 3.11 s (12H, CH_3_), 3.70 s (6H), 7.81–7.82 d (2H, CH), 6.31–6.32 d (2H, CH), 7.32–7.33 d (2H, CH), 7.39–7.41 d (4H, CH), 7.43–7.44 t (4H, CH), 7.47–7.48 t (2H, CH). ^13^C-NMR (DMSO, ppm) δ: 46.6 (C1,1′), 53.4 (C2,2′), 153.6 (C3,3′), 161.4 (C4,4′), 162.5 (C5,5′), 127.8 (C6,6′), 136.0 (C7,7′), 136.5 (C8,8′), 135.7 (C9,9′), 128.8 (C10,10′), 128.4 (C11,11′), 128.7 (C12,12′), 135.3 (C13,13′).

MZn4; yield (58%); M.P. 210–211 °C; molecular formula: C_10_H_32_N_10_O_9_SZn; molecular weight: 533.87 g/mol. Elemental analysis for C_10_H_32_N_10_O_9_SZn: (calculated) C, 22.50; H, 6.04; N, 26.24; S, 6.01; (found) C, 22.55; H, 6.00; N, 26.21; S, 6.07. FT-IR ν (cm^−1^), 2961, 2889 (C–H, s, stretching), 1653 (C=N, m, bending), 1178 (C–N, m, bending), 3353 (N–H, s, stretching). ^1^H-NMR (DMSO, ppm) δ: 3.41 s (12H, CH_3_), 1.11 s, 7.51 s, 7.45 s (6H), 7.72 s (4H, CH_2_). ^13^C-NMR (DMSO, ppm) δ: 45.6 (C1,1′) 46.7 (C2,2′), 154.3 (C3,3′), 161.7 (C4,4′), 151.4 (C5,5′).

MZn5; yield (66%); M.P. 152–153 °C; molecular formula: C_14_H_40_N_10_O_9_SZn; molecular weight: 589.97 g/mol. Elemental analysis for C_14_H_40_N_10_O_9_SZn: (calculated) C, 28.50; H, 6.83; N, 23.74; S, 5.43; (found) C, 28.55; H, 6.80; N, 23.70; S, 5.46. FT-IR ν (cm^−1^), 3001 (C–H, s, stretching), 1645 (C=N, m, bending), 1235 (C–N, m, bending), 3271 (N–H, s, stretching). ^1^H-NMR (DMSO, ppm) δ: 2.71 s (12H, CH_3_), 1.51 s, 7.58 s 7.63 s (6H, NH), 1.25 s, 1.11s (12H, CH_3_). ^13^C-NMR (DMSO, ppm) δ: 45.2 (C1,1′), 47.6 (C2,2′), 151.2 (C3,3′), 149.7 (C4,4′), 158.1 (C5,5′), 25.6 (C6,6′), 12.6 (C7,7′).

MZn6; yield (74%); M.P. 211–212 °C; molecular formula: C_24_H_44_N_10_O_9_SZn; molecular weight: 714.11 g/mol. Elemental analysis for C_24_H_44_N_10_O_9_SZn: (calculated) C, 40.37; H, 6.21; N, 19.61; S, 4.49; (found) C, 40.41; H, 6.18; N, 19.63; S, 4.53. FT-IR ν (cm^−1^), 2952 (C–H, s, stretching), 1635 (C=N, m, bending), 1603, 1476 (C=C, m, bending), 1178 (C–N, m, bending). ^1^H-NMR (DMSO, ppm) δ: 1.30 s, 2.35 s (18H, CH_3_), 6.61 s (6H, NH), 7.11–7.12 d (4H, CH), 7.50–7.51 m (6H, CH). ^13^C-NMR (DMSO, ppm) δ: 55.6 (C1,1′), 59.6 (C2,2′), 152.5 (C3,3′), 156.2 (C4,4′), 163.4 (C5,5′), 136.4 (C6,6′), 127.5(C7,7′), 134.6 (C8,8′), 136.3 (C9,9′), 134.5 (10,10′) 127.3 (C11,11′), 25.3 (C12,12′).

MZn7; yield (64%); M.P. 237 °C; molecular formula: C_34_H_48_N_10_O_9_SZn; molecular weight: 838.25 g/mol. Elemental analysis for C_34_H_48_N_10_O_9_SZn: (calculated) C, 48.72; H, 5.77; N, 16.71; S, 3.83; (found) C, 48.70; H, 5.79; N, 16.75; S, 3.80. FT-IR ν (cm^−1^), 3006 (C–H, s, stretching), 1657 (C=N, m, bending), 1601, 1473 (C=C, m, bending), 1165 (C–N, m, bending), 3303 (N–H, s, stretching). ^1^H-NMR (DMSO, ppm) δ: 2.85 s, 3.05 s, 3.17 s, (12H, CH_3_), 2.81 s, 2.85 s (6H, NH), 7.41–7.42 d, 7.45–7.46 m (10H, CH), 7.81–7.82 d, 7.95–7.96 m (10H, CH). ^13^C-NMR (DMSO, ppm) δ: 45.5 (C1,1′), 43.3 (C2,2′), 151.3 (C3,3′), 156.9 (C4,4′), 162.3 (C5,5′), 149.7 (C6,6′), 138.9 (C7,7′), 129.8 (C8,8′), 128.9 (C9,9′) 135.7 (C10,10′), 128.7 (C11,11′), 129.9 (C12,12′), 137.8 (C13,13′), 128.3 (C14,14′), 127.9 (C15,15′), 136.1 (C16,16′), 128.0 (C17,17′).

MZn8; yield (67%); M.P. 197 °C; molecular formula: C_24_H_44_N_10_O_13_SZn; molecular weight: 778.11 g/mol. Elemental analysis for C_24_H_44_N_10_O_13_SZn: (calculated) C, 37.05; H, 5.70; N, 18.00; S, 4.12; (found) C, 37.10; H, 5.65; N, 17.95; S, 4.15. FT-IR ν (cm^−1^), 3051 (C–H, s, stretching), 1681, (C=N, m, bending), 1601, 1473 (C=C, m, bending), 1173 (C–N, m, bending), 3291 (N–H, s, stretching). ^1^H-NMR (DMSO, ppm) δ: 2.10 s, 3.22 s, 2.15 s, (18H, CH_3_), 8.01 s (2H, CH), 1.61 s, 1.51 s, (6H, NH), 6.90–6.91 d, 7.10–7.11 d, 7.35 s (6H, CH), 4.83 s, (2H, OH). ^13^C-NMR (DMSO, ppm) δ: 55.4 (C1,1′), 54.3 (C2,2′), 156.6 (C3,3′), 159.5 (C4,4′), 163.1 (C5,5′), 127.2 (C6,6′), 119.1 (C7,7′), 145.5 (C8,8′), 146.1 (C9,9′), 112.4 (C10,10′), 119.7 (C11,11′), 45.5 (C12,12′).

MZn9; yield (77%); M.P. 240 °C; molecular formula: C_36_H_52_N_10_O_11_SZn; molecular weight: 898.30 g/mol. Elemental analysis for C_36_H_52_N_10_O_11_SZn: (calculated) C, 48.13; H, 5.83; N, 15.59; S, 3.57; (found) C, 48.17; H, 5.88; N, 15.62; S, 3.51. FT-IR ν (cm^−1^), 3021, 2971 (C–H, s, stretching), 1657 (C=N, m, bending), 1605, 1474 (C=C, m, bending), 1157 (C–N, m, bending), 3301 (N–H, s, stretching). ^1^H-NMR (DMSO, ppm) δ: 2.11 s 1.90 s, 1.82 s, 1.98 s, (12H, CH_3_), 1.22 s, 1.31 s, (6H, NH), 4.41 s (2H, CH), 7.30–7.31 m, 7.34–7.35 m (10H, CH), 7.76–7.77 d, 7.42–7.43 m (10H, CH), 3.20 s, (2H, OH). ^13^C-NMR (DMSO, ppm) δ: 56.3 (C1,1′), 57.0 (C2,2′), 158.5 (C3,3′), 160.4 (C4,4′), 162.3 (C5,5′), 61.3 (C6,6′), 138.4 (C7,7′), 126.5 (C8,8′), 127.7 (C9,9′), 128.5 (C10,10′), 127.7 (C11,11′), 126.1 (C12,12′) 132.9 (C13,13′), 127.0 (C14,14′), 128.1 (C15,15′), 130.7 (C16,16′), 128.9 (C17,17′), 126.7 (C18,18′).

### 2.2. In Silico ADME Studies

**Figure 1:**
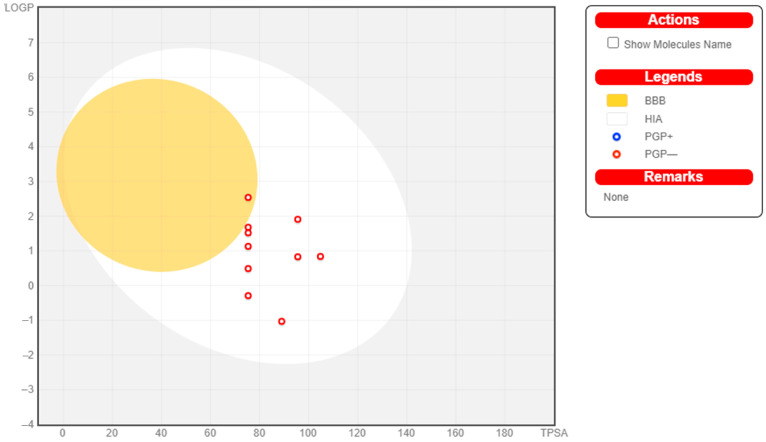
Swiss ADME Analysis of Met1–Met9 (egg plot).

**Figure 2:**
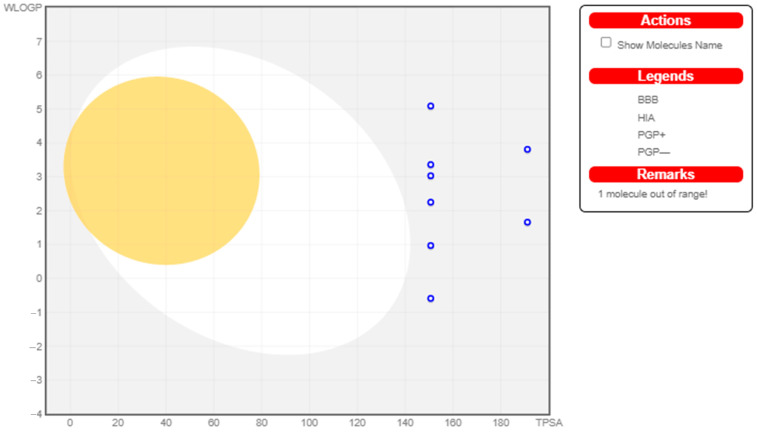
Swiss ADME Analysis of MCu1–MCu9.

**Figure 3:**
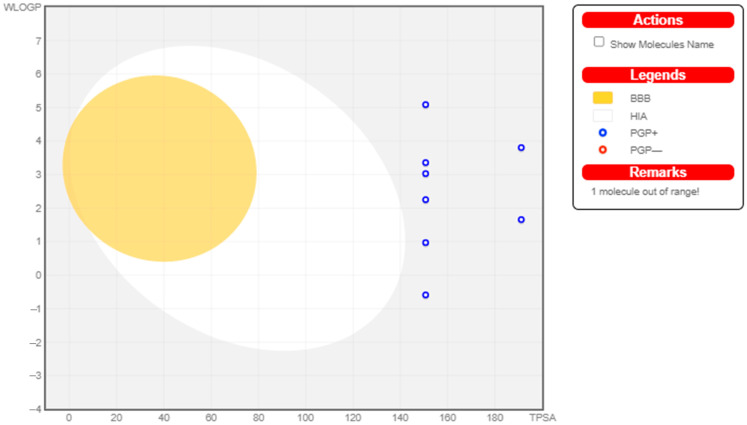
Swiss ADME Analysis of MZn1–MZn9.

Swiss ADME can help to determine different physicochemical properties of novel tested compounds such as molecular weight, hydrogen bond donors (HBDs), hydrogen bond acceptors (HBAs), partition coefficient (log P). and solvent-accessible surface area within the desired range of acceptance, with the results depicted in Table 1, Table 2 and Table 3 and represented in Figure 1, Figure 2 and Figure 3. The detailed ADME predictions are shown in Table S1 (Supplementary Materials).

### 2.3. Enzyme Inhibition Assays

#### 2.3.1. α-Glucosidase

The α-glucosidase enzyme inhibition potential of metformin derivatives is presented in Table 4, Table 5 and Table 6. The graphical depiction of the results is shown in Figures S1–S3 (Supplementary Materials). Metabolites Met8 (IC_50_ 562.64 µg/mL), MCu1 (IC_50_ 421.7 µg/mL), and MZn8 (IC_50_ 511.4 µg/mL) expressed the highest α-glucosidase inhibition activities. Furthermore, Table S3 (Supplementary Materials) shows 2D structures of all metformin derivatives with respect to glucosidase enzyme.

#### 2.3.2. α-Amylase Assay

The inhibitory potential along with the IC_50_ values of metformin derivatives against the α-amylase enzyme is displayed in Table 7, Table 8 and Table 9. The maximum percentage inhibition against α-amylase was displayed by Met8 (IC_50_ 752.376 µg/mL), MCu8 (IC_50_ 712.6 µg/mL), and MZn2 (IC_50_ 484.9 µg/mL). The graphical representation of the results is shown in Figures S4–S6 (Supplementary Materials). Table S2 (Supplementary Materials) depicts 2D structures of all Metformin derivatives.

### 2.4. Molecular Docking Studies

**Figure 4:**
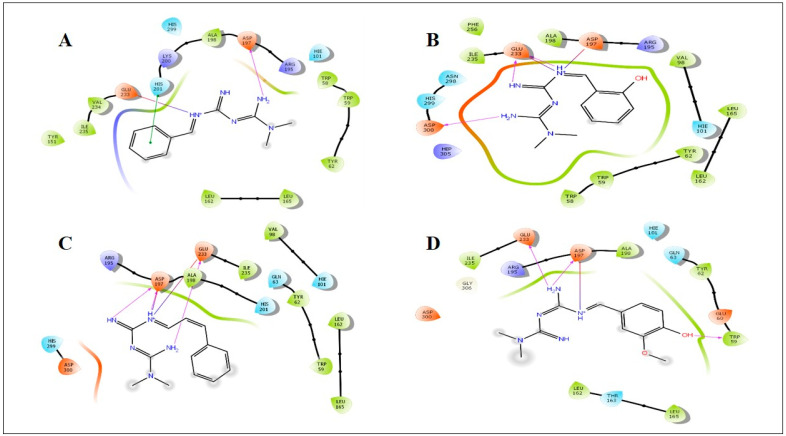
Induced fit docking study of the best pose generated on α-amylase (5E0F): (**A**) Met1 ligand-binding site; (**B**) Met2 ligand-binding site; (**C**) Met3 ligand-binding site; (**D**) Met8 ligand-binding site.

**Figure 5:**
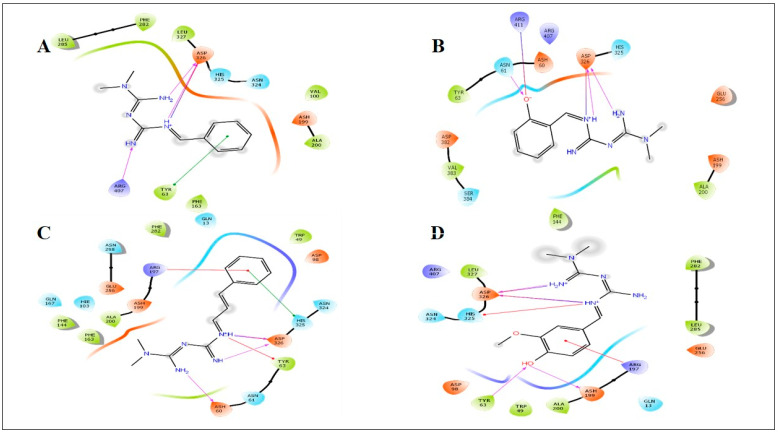
Induced fit docking study of the best pose generated on α-glucosidase (2ZEO): (**A**) Met1 ligand-binding site; (**B**) Met2 ligand-binding site; (**C**) Met3 ligand-binding site; (**D**) Met8 ligand-binding site.

**Figure 6:**
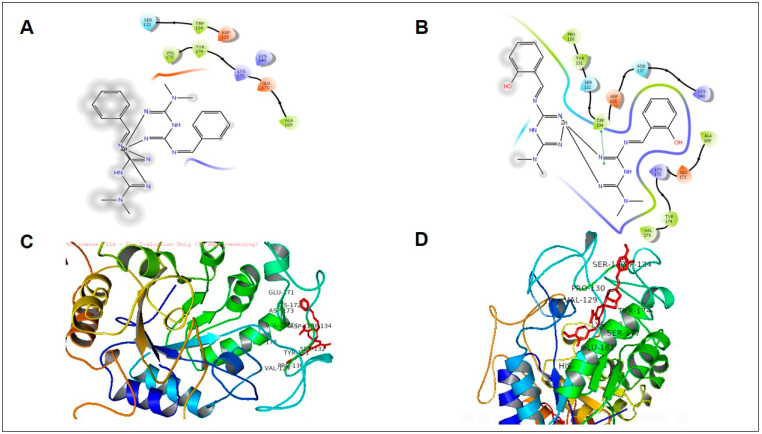
Induced fit docking study of the best pose generated on α-amylase: (**A**) MZn1 ligand-binding site; (**B**) MZn2 ligand-binding site; (**C**) MZn3 ligand-binding site; (**D**) MZn8 ligand-binding site.

**Figure 7:**
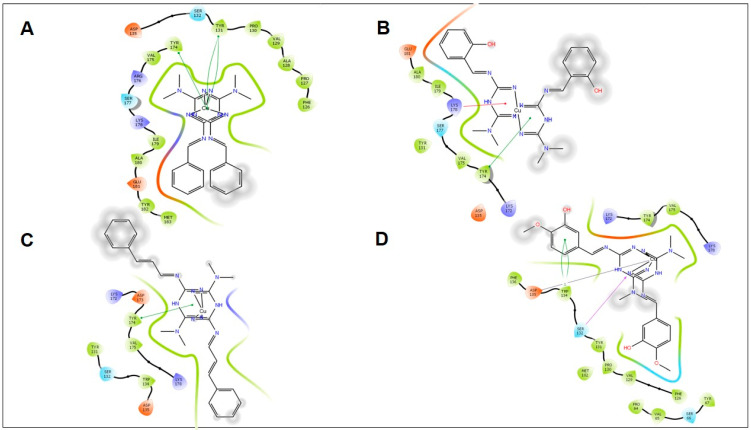
Induced fit docking study of the best pose generated on α-amylase: (**A**) MCu1 ligand-binding site; (**B**) MCu2 ligand-binding site; (**C**) MCu3 ligand-binding site; (**D**) MCu8 ligand-binding site.

**Figure 8:**
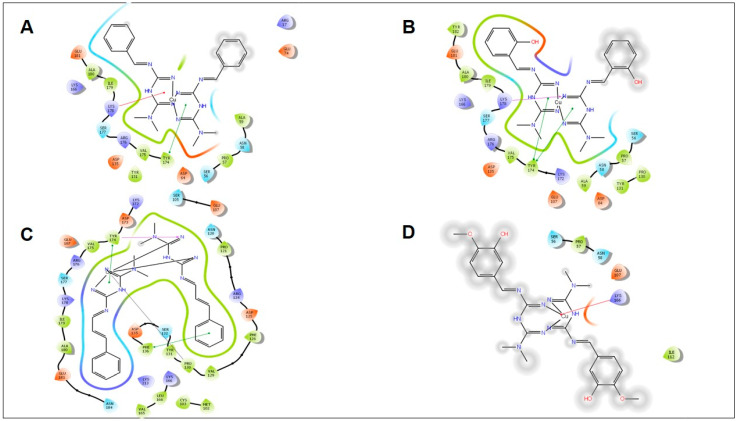
Induced fit docking study of the best pose generated on α-glucosidase: (**A**) MCu1 ligand-binding site; (**B**) MCu2 ligand-binding site; (**C**) MCu3 ligand-binding site; (**D**) MCu8 ligand-binding site.

A total of 27 ligands were docked to the enzymes. The interactions of the receptors α-amylase and α-glucosidase with the ligands are shown in Figure 4, Figure 5, Figure 6, Figure 7 and Figure 8, while the binding affinities and binding forces of the ligands and enzyme are reported in Table 10 and Table 11.

### 2.5. Three-Dimensional Coordination Geometries

The 3D coordination geometries and poses were established to show the 3D coordination of all metformin derivatives and metal complexes with respect to different interaction sites (Table S4, Supplementary Materials).

## 3. Discussion

Most drugs fail at the clinical trial stage or are withdrawn from the market due to the negative properties of ADME (absorption, distribution, metabolism, and excretion) and unavoidable side-effects. The in silico prediction of ADME properties can reduce the effort and time needed by investigators to make models for every new derivative in the development of a lead compound [22]. Swiss ADME can help to determine the different physicochemical properties of novel tested compounds such as molecular weight, hydrogen bond donors (HBDs), hydrogen bond acceptors (HBAs), partition coefficient (log P), and solvent-accessible surface area within a desired range of acceptance. All novel derivatives of metformin had the ability to cross the gastrointestinal layer, and only one derivative (MET-7) could cross the blood–brain barrier (BBB). All novel derivatives exhibited log S under the predicted value (−6.5 to 0.5). All derivatives followed Lipinski’s rule of five and were freely soluble in aqueous media. Therefore, according to the overall physicochemical prediction, most derivatives possessed drug-likeness behavior. No derivatives of copper and zinc could cross the blood–brain barrier, and they had low affinity for gastrointestinal absorption.

All derivatives showed a positive effect of enzyme inhibition on amylase and glucosidase. Met8 showed good enzyme inhibition when compared to the parent drug, as well as the standard. Met1, Met2, and Met3 showed comparable activity against glucosidase and amylase. The metal complexes with copper showed a linear graph, whereas zinc derivatives showed abrupt inhibition values.

Docking studies were performed to evaluate the binding interaction and binding energy between the receptors and small organic molecules. Docking results were further characterized with biological studies. All novel synthesized derivatives were simulated with the active pockets of α-glucosidase and α-amylase. After evaluating the docking scores (Table 7 and Table 8), only four active compounds (Met1, Met2, Met3, and Met8) were selected for deep insight into different interactions.

All synthetic derivatives were analyzed by molecular simulation studies to identify binding interactions in the active pocket site of the enzyme. The radiographic structures of human pancreatic α-amylase (PDB: 5EOF) were selected as models for this purpose. The molecular docking results showed that several interactions were available between the derivatives and active pocket residues. The top four active derivatives were Met1, Met2, Met3, and Met8, showing multiple additional hydrogen bond hydrophobic interactions with residues Tyr62, Leu165, Lys200, and Ile235. In addition to the hydrophobic interactions, multiple hydrogen bonds were also observed. The metal bond order was considered as zero order [23]. Therefore, the force field method was able to adequately handle the coordinate bonds of metal complexes. All derivatives had a nitrogen moiety to build a hydrogen bond with residues Asp197, Glu233, and Asp300. However, an additional hydrogen bond was formed by the hydroxyl group of the derivatives with Trp59. Additional interactions such as salt bridges were seen in derivatives of positively charged nitrogen atoms on residues Asp197 and Glu233. Derivative Met1 exhibited pi stacking with the phenyl group of His201. Zinc complex derivatives showed different types of interactions in the pocket site such as hydrophobic interactions, pi–pi stacking, positive hydrogen bond, and metal bonding. MZn1, MZn2, MZn3, and MZn8 exhibited hydrophobic interactions with Val175, Ala169, and Trp134. MZn2 established a positive hydrogen bond with Trp134. Copper metal derivatives exhibited a positive hydrophobic interaction with copper metal, with the benzene ring being mainly responsible for the hydrophobic interactions. MCu1, MCu2, MCu3, and MCu8 established hydrogen bonds with copper via the active pocket residues Tyr131 and Tyr174. MCu8 exhibited a strong metal bond with copper metal and with residue Asp135. 

The simulated binding of compounds resulted in several polar and nonpolar contacts with our target protein (2ZEO). Although the compounds were present in the active pocket site, some minor changes were observed. The nitrogen groups formed hydrogen bonds with active residue Asn61, Tyr63, Asp199, and Asp326, although the active pocket featured a few hydrophobic residues, e.g., Trp49, Val100, Asp199, Ala200, Leu285, and Val383. The novel derivatives established pi–pi stacking of Met1 and Met3 with the phenyl rings of residues Tyr63 and His325, as well as salt bridges between the positively charged nitrogen moieties present in the structure via residue Asp326, while Met2 formed a salt bridge with Arg411 of the hydroxyl group present in the structure.

In vitro studies showed that the Met8 derivative exhibited both amylase and glucosidase activity, while the docking studies revealed that derivative Met2 showed the highest docking scores for both enzymes. The α-glucosidase activity of derivative Met8 showed good activity in both in vitro and in silico docking studies. The metal complex with copper showed better activity than zinc complexes. The α-amylase activity results and docking studies revealed that MZn8 and MCu8 showed good activity.

## 4. Materials and Methods

### 4.1. Synthesis of Metformin Schiff Base

**Scheme 1:**
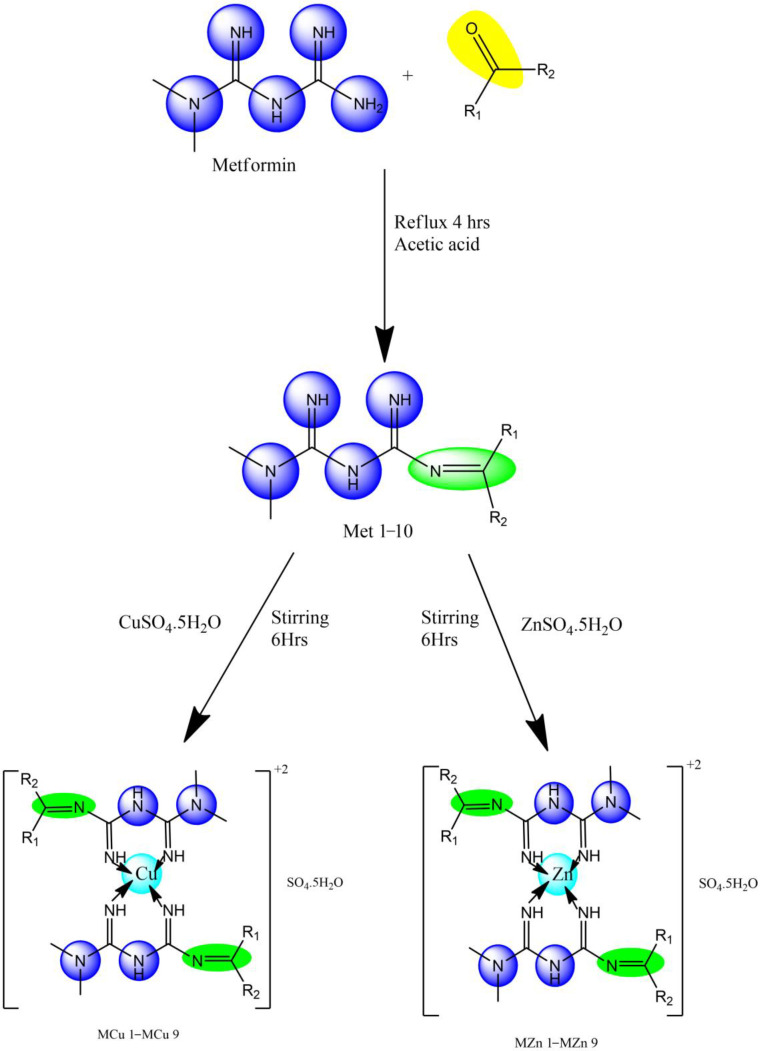
Synthesis of metformin Schiff base with copper (MCu1–MCu9) and Zinc (MZn1–MZn9) complexes.

Metformin was dissolved in 10 mL of methanol, and the methanolic solution was reacted with aromatic aldehyde/ketone added dropwise. Drops of acetic acid were added, and the mixture was refluxed with continuous stirring over 4 h. Synthesized products were recrystallized by washing with methanol. Scheme 1 represents copper and zinc metal complexes with functional groups depicted in Table 12.

### 4.2. Copper Complexes of Metformin Derivatives (Met1–Met9)

The complexes were prepared via the reaction of CuSO_4_·5H_2_O and ligands (Met1–Met9) in methanol. The methanolic solution (10 mL) of CuSO_4_·5H_2_O (0.748 g; 3 mmol) was added dropwise to the methanolic solution (20 mL) of ligand (6 mmol) with constant stirring for 6 h. The solution was then concentrated to 15 mL volume and left to stand for 48 h. The precipitate was filtered with filter paper, and then dried in a desiccator for 24 h.

### 4.3. Zinc Complexes of Metformin Derivatives (Met1–Met9)

The complexes were prepared via the reaction of ZnSO_4_·5H_2_O and ligands (Met1–Met9) in methanol. Methanolic solution (10 mL) of ZnSO_4_·5H_2_O (0.748 g; 3 mmol) was added dropwise to the methanolic solution (20 mL) of ligand (6 mmol) with constant stirring for 6 h. The solution was then concentrated to 15 mL volume and left to stand for 48 h. The precipitate was filtered with filter paper, and then dried in a desiccator for 24 h.

### 4.4. In Silico ADME Prediction

Swiss ADME, an online ADME prediction tool, was applied in the present study to predict the drug likeness and physicochemical properties of novel derivatives of metformin [24]. The structure of derivatives was translated into canonical SMILES format and then submitted to the Swiss ADME tool, which provides free access to predict different properties of compounds, e.g., drug likeness, Lipinski’s rule of five, gastrointestinal absorption, blood–brain barrier, and total polar surface area [25].

### 4.5. Enzyme Inhibition Analysis

#### 4.5.1. α-Amylase

All derivatives of metformin were analyzed for serum amylase inhibitory activity using the Yukihoko Hara method [26]. Three test tubes were labeled as blank (B), test (T), and control (C). Then, 2.5 mL of phosphate buffer (pH 6.8) was added to each test tube. Next, 1 mL of starch and 1 mL of 2 N sodium chloride solution were added to each test tube. The test tubes were incubated at 37 °C for 10 min. After incubation, 0.5 mL of derivatives was added to test tube T, followed by 0.2 mL of enzyme (α-amylase). All contents were mixed well, and then the tube was incubated at 37 °C for 10 min. In the control test tube, normal saline (2 N), starch solution (1 mL), and enzyme solution (0.2 mL) were added before incubating at 37 °C for 10 min. In the blank test tube, only 5.7 mL of distilled water was added. Dinitro-salicylic acid (0.2 mL) was added to all test tubes. The contents were mixed well, and the test tubes were kept in a boiling water bath for 15 min. The intensity of reddish orange was read at 540 nm. The percentage inhibitory action of serum amylase was calculated using the following formula:

Percentage inhibition = OD of control − OD of test tube/OD of control × 100.

#### 4.5.2. α-Glucosidase

The α-glucosidase-inhibitory activity of all derivatives of metformin was tested using the method previously described [27]. Three test tubes were marked as test (T), blank (B), and control (C). Then, 200 µL of substrate (starch) and enzyme (α-glucosidase) was added to test tubes T and C. The contents were mixed well, and then the test tubes were incubated at 37 °C for 30 min. After incubation, trichloroacetic acid was added to all test tubes, which were then centrifuged for 10 min. Free glucose was available after the reaction with anthrone reagent (1.5 mL). The contents in the test tubes were boiled for 15 min, and the absorbance was read at a wavelength at 640 nm. The percentage inhibition was calculated using the following formula:

Percentage inhibition = OD of control − OD of test tube/OD of control × 100.

### 4.6. Molecular Docking

Docking studies of novel derivatives of metformin were performed using the Glide module of Schrodinger software 4.6 [28,29]. The selected target proteins (amylase and glucosidase) were downloaded from the Protein Data Bank (RCSB) [30]. Novel derivatives of metformin were drawn using Chemdraw12.0 PerkinElmer software.

#### 4.6.1. Ligand Preparation

All ligands used in the docking studies were drawn in ChemDraw 12.0, and the structure was optimized for bond and structure alignment. Then, the structure was imported into the Schrodinger software workspace for energy minimization using the OPLS4 (version 9.03) (Optimized Potentials for Ligand Simulations) (44) force field in LigPrep wizard [31]. Maestro (12.8) was then used to hydrogens, assign bond orders, and convert the 2D ligand structure to 3D.

#### 4.6.2. Protein Preparation

The Protein preparation wizard in Maestro was used to minimize the selected proteins from RCSB. All nonprotein molecules were removed without interfering with the already attached ligand, which became the basis for the grid box generation. As a first step, charges were equally distributed, and polar hydrogens were added using Epik at pH 7.0 ± 2.0. The heat state was generated, and the protein structure was refined. Water molecules were removed before the protein was subjected to docking analyses.

#### 4.6.3. Receptor Grid Generation

The receptor grid was generated using the co-crystallized ligand. The ligand center was selected for grid box creation, and the van der Waals radius for the receptor was scaled at 1.00 Å with a partial charge of 0.25. For α-amylase (5E0F), the grid box coordinates were x −7.20, y 5.69, z −23.42 with a box size of 20 Å. For α-glucosidase (2ZE0), the grid box coordinates were x 10.39, y 4.11, z 16.74 with a box size of 20 Å.

#### 4.6.4. Docking and Analysis

Docking studies were carried out using the Glide wizard with the proteins and ligands prepared as described above. In the final step of docking, in extra precision mode, the RMSD (root-mean-square deviation) value was restrained within 0.46 Å and the atomic charge was restrained within 0.15 for the receptor and all derivatives. When the docking process was complete, the interactions were observed using XP visualizer and Pymol (1.5.7).

## 5. Conclusions

This study focused on the synthesis, characterization, design, and screening of many metformin derivatives with the potential to effectively treat diabetes while causing only a small number of adverse effects. It was determined whether any of the compounds had the ability to inhibit the enzymes α-amylase and α-glucosidase. Only derivatives 1, 2, 3, and 8 exhibited any significant activity during the amylase and glucosidase tests. Each derivative included a benzene ring with a replacement. The presence of a substituted benzene ring in the meta position, a hydroxyl group, and an ether linkage in the para position provided derivative 8 with the maximum activity. This was also due to the presence of a hydroxyl group. When two benzene rings were included, a reduction in activity was observed, e.g., in derivatives 7 and 9. Docking experiments were conducted on all compounds against the human pancreatic amylase and glucosidase enzymes, and the results showed a variety of interactions, including hydrogen bonds, pi–pi stacking, and salt bridges. Accordingly, we were able to identify novel metformin derivatives with the potential to serve as lead compounds in subsequent studies for the development of effective inhibitors.

## Figures and Tables

**Table 1:** Swiss ADME of Met1–Met9.

Compound	TPSA	% Absorption	GI Absorption	BBB Permeant	Lipinski Violations
Met1	75.33	83	High	No	0
Met2	95.56	76	High	No	0
Met3	75.33	83	High	No	0
Met4	75.33	83	High	No	0
Met5	75.33	83	High	No	0
Met6	75.33	83	High	No	0
Met7	75.33	83	High	Yes	0
Met8	104.79	73	High	No	0
Met9	95.56	76	High	No	0

**Table 2:** Swiss ADME of MCu1–MCu9.

Compound	TPSA	% Absorption	GI Absorption	BBB Permeant	Lipinski Violations
MCu1	150.66	57	Low	No	1
MCu2	191.12	43	Low	No	3
MCu3	150.66	57	Low	No	3
MCu4	150.66	57	Low	No	1
MCu5	150.66	57	Low	No	1
MCu6	150.66	57	Low	No	1
MCu7	150.66	57	Low	No	3
MCu8	209.58	37	Low	No	3
MCu9	191.12	43	Low	No	3

**Table 3:** Swiss ADME of MZn1–MZn9.

Compound	TPSA	% Absorption	GI Absorption	BBB Permeant	Lipinski Violations
MZn1	150.66	57	Low	No	1
MZn2	191.12	43	Low	No	3
MZn3	150.66	57	Low	No	3
MZn4	150.66	57	Low	No	1
MZn5	150.66	57	Low	No	1
MZn6	150.66	57	Low	No	1
MZn7	150.66	57	Low	No	3
MZn8	209.58	37	Low	No	3
MZn9	191.12	43	Low	No	3

**Table 4:** α-Glucosidase activity of Met1–Met9.

Samples	Concentration (µg/mL)	% Inhibition	IC_50_ (µmol/mL)
Met1	250	47.26 ± 0.09	0.002
	500	67.80 ± 0.15	
	1000	77.23 ± 0.25	
Met2	250	23.85 ± 0.15	0.0019
	500	39.33 ± 0.30	
	1000	63.21 ± 0.19	
Met3	250	43.33 ± 0.30	0.0017
	500	51.37 ± 0.19	
	1000	69.57 ± 0.23	
Met4	250	17.49 ± 0.29	0.0011
	500	25.77 ± 0.35	
	1000	43.72 ± 0.26	
Met5	250	22.85 ± 0.15	0.002
	500	39.43 ± 0.19	
	1000	53.44 ± 0.15	
Met6	250	16.41 ± 0.19	0.0013
	500	28.86 ± 0.15	
	1000	51.47 ± 0.26	
Met7	250	27.42 ± 0.32	0.0009
	500	41.84 ± 0.15	
	1000	55.77 ± 0.01	
Met8	250	36.50 ± 0.26	0.0021
	500	59.63 ± 0.20	
	1000	79.49 ± 0.26	
Met9	250	10.79 ± 0.10	0.0006
	500	29.86 ± 0.10	
	1000	39.09 ± 0.05	
Met	250	41.73 ± 0.10	0.0048
	500	52.83 ± 0.11	
	1000	81.30 ± 0.10	
(Acarbose)	250	41.84 ± 0.08	0.00094
	500	66.79 ± 0.09	
	1000	89.01 ± 0.01	

**Table 5:** α-Glucosidase activity of MCu1–MCu9.

Samples	Concentration (µg/mL)	% Inhibition	IC_50_ (µmol/mL)
MCu1	250	44.24 ± 0.10	0.0006
	500	61.46 ± 0.31	
	1000	81.13 ± 0.08	
MCu2	250	31.13 ± 0.04	0.00001
	500	48.50 ± 0.22	
	1000	77.32 ± 0.17	
MCu3	250	29.74 ± 0.08	0.00057
	500	49.26 ± 0.17	
	1000	78.23 ± 0.17	
MCu4	250	18.64 ± 0.07	0.0005
	500	31.28 ± 0.08	
	1000	51.20 ± 0.18	
MCu5	250	21.73 ± 0.12	0.00046
	500	38.21 ± 0.18	
	1000	56.45 ± 0.20	
MCu6	250	10.74 ± 0.11	0.00029
	500	24.44 ± 0.15	
	1000	53.44 ± 0.33	
MCu7	250	29.47 ± 0.18	0.00032
	500	37.37 ± 0.30	
	1000	48.29 ± 0.28	
MCu8	250	41.50 ± 0.26	0.00072
	500	67.35 ± 35	
	1000	83.14 ± 0.09	
MCu9	250	15.23 ± 0.12	0.0002
	500	28.37 ± 0.20	
	1000	49.49 ± 0.25	
MCu	250	33.63 ± 0.10	0.0015
	500	61.15 ± 0.08	
	1000	89.43 ± 0.08	
(Acrabose)	250	42.65 ± 0.09	0.0012
	500	62.41 ± 0.28	
	1000	91.83 ± 0.18	

**Table 6:** α-Glucosidase activity of MZn1–MZn9.

Samples	Concentration (µg/mL)	% Inhibition	IC_50_ (µmol/mL)
MZn1	250	54.74 ± 0.15	0.0007
	500	62.48 ± 0.19	
	1000	77.57 ± 0.20	
MZn2	250	43.55 ± 0.20	0.0006
	500	57.61 ± 0.15	
	1000	71.37 ± 0.30	
MZn3	250	50.22 ± 0.15	0.00054
	500	58.18 ± 0.21	
	1000	68.63 ± 0.20	
MZn4	250	11.30 ± 0.15	0.0005
	500	13.59 ± 0.20	
	1000	14.71 ± 0.26	
MZn5	250	32.86 ± 0.15	0.00029
	500	38.87 ± 0.10	
	1000	49.46 ± 0.20	
MZn6	250	36.67 ± 0.26	0.00028
	500	41.26 ± 0.32	
	1000	44.51 ± 0.19	
MZn7	250	33.67 ± 67	0.00024
	500	37.61 ± 0.20	
	1000	42.54 ± 0.26	
MZn8	250	49.24 ± 0.21	0.00065
	500	70.37 ± 0.25	
	1000	81.54 ± 0.26	
MZn9	250	28.64 ± 0.25	0.00024
	500	37.51 ± 0.25	
	1000	45.37 ± 0.20	
MZn	250	51.36 ± 0.18	0.0013
	500	73.55 ± 0.15	
	1000	95.64 ± 0.32	
Standard	250	54.12 ± 0.09	0.001
	500	74.52 ± 0.19	
	1000	98.60 ± 0.10	

**Table 7:** α-Amylase activity of Met1–Met9.

Samples	Concentration (µg/mL)	% Inhibition	IC_50_ (mmol/mL)
Met1	250	33.39 ± 0.15	0.0032
	500	48.61 ± 0.32	
	1000	70.60 ± 0.32	
Met2	250	34.52 ± 0.30	0.0031
	500	69.22 ± 0.20	
	1000	86.51 ± 0.26	
Met3	250	23.49 ± 0.26	0.00044
	500	41.39 ± 0.25	
	1000	68.30 ± 0.17	
Met4	250	15.48 ± 0.15	0.0007
	500	37.65 ± 0.19	
	1000	55.48 ± 0.23	
Met5	250	15.61 ± 0.20	0.00074
	500	39.48 ± 0.32	
	1000	63.47 ± 0.23	
Met6	250	18.48 ± 0.26	0.0017
	500	33.35 ± 0.20	
	1000	41.34 ± 0.17	
Met7	250	24.25 ± 0.20	0.0014
	500	34.45 ± 0.26	
	1000	48.33 ± 0.51	
Met8	250	37.77 ± 0.19	0.0029
	500	68.43 ± 0.30	
	1000	83.49 ± 0.34	
Met9	250	5.41 ± 0.20	0.00079
	500	8.61 ± 0.25	
	1000	19.71 ± 0.46	
Met	250	55.27 ± 0.20	0.0094
	500	63.18 ± 0.20	
	1000	85.25 ± 0.32	
(Acarbose)	250	49.65 ± 0.15	0.0025
	500	72.33 ± 0.20	
	1000	91.58 ± 0.36	

**Table 8:** α-Amylase activity of MCu1–MCu9.

Samples	Concentration (µg/mL)	% Inhibition	IC_50_ (mmol/mL)
MCu1	250	19.44 ± 0.46	0.00099
	500	43.71 ± 0.17	
	1000	73.31 ± 0.51	
MCu2	250	29.52 ± 0.28	0.00079
	500	44.56 ± 0.34	
	1000	77.56 ± 0.34	
MCu3	250	25.41 ± 0.23	0.00079
	500	38.37 ± 0.40	
	1000	69.40 ± 0.23	
MCu4	250	19.48 ± 0.40	0.0003
	500	31.46 ± 0.23	
	1000	48.32 ± 0.51	
MCu5	250	12.46 ± 0.23	0.00032
	500	38.44 ± 0.40	
	1000	61.38 ± 0.23	
MCu6	250	23.34 ± 0.51	0.00032
	500	45.45 ± 0.28	
	1000	64.65 ± 0.28	
MCu7	250	15.37 ± 0.23	0.0002
	500	38.31 ± 0.46	
	1000	55.43 ± 0.23	
MCu8	250	42.68 ± 0.46	0.0009
	500	61.35 ± 0.51	
	1000	84.35 ± 0.51	
MCu9	250	13.46 ± 0.23	0.00013
	500	34.66 ± 0.40	
	1000	67.48 ± 0.40	
MCu	250	36.52 ± 0.28	0.0013
	500	54.52 ± 0.34	
	1000	72.43 ± 0.23	
(Acrabose)	250	42.73 ± 0.46	0.0012
	500	67.52 ± 0.34	
	1000	92.73 ± 0.46	

**Table 9:** α-Amylase activity results of MZn1–MZn9.

Samples	Concentration (µg/mL)	% Inhibition	IC_50_ (mmol/mL)
MZn1	250	31.46 ± 0.34	0.00075
	500	55.54 ± 0.34	
	1000	82.60 ± 0.40	
MZn2	250	34.46 ± 0.23	0.00067
	500	69.32 ± 0.46	
	1000	86.58 ± 0.40	
MZn3	250	13.59 ± 0.34	0.00078
	500	41.55 ± 0.34	
	1000	78.57 ± 0.28	
MZn4	250	15.55 ± 0.34	0.0005
	500	31.49 ± 0.28	
	1000	54.44 ± 0.28	
MZn5	250	15.44 ± 0.28	0.00056
	500	37.60 ± 0.28	
	1000	58.35 ± 0.17	
MZn6	250	18.76 ± 0.17	0.00054
	500	33.29 ± 0.46	
	1000	51.47 ± 0.28	
MZn7	250	24.32 ± 0.51	0.00025
	500	44.52 ± 0.34	
	1000	73.43 ± 0.28	
MZn8	250	37.46 ± 0.23	0.0009
	500	68.50 ± 0.40	
	1000	83.52 ± 0.40	
MZn9	250	15.64 ± 0.28	0.0007
	500	38.64 ± 0.40	
	1000	59.70 ± 0.46	
MZn	250	55.37 ± 0.46	0.0015
	500	63.32 ± 0.51	
	1000	85.32 ± 0.51	
(Acrabose)	250	49.59 ± 0.34	0.004
	500	72.73 ± 0.46	
	1000	91.71 ± 0.46	

**Table 10:** Docking scores of metformin derivatives.

Derivative	α-Amylase (5E0F)	α-Glucosidase (2ZEO)
Met1	−4.533	−5.765
Met2	−5.42	−5.445
Met3	−5.471	−5.682
Met4	−5.049	−2.625
Met5	−3.537	−2.160
Met6	−2.569	−3.286
Met7	−4.031	−5.986
Met8	−7.08	−5.437
Met9	−5.128	−4.395
Met	−4.971	−3.008
(Acorbose)	−14.78	−15.082

**Table 11:** Docking scores of metformin metal complex derivatives.

Derivative	α-Amylase (5E0F)	α-Glucosidase (2ZEO)
MCu1	−4.318	−3.967
MCu2	−4.764	−4.186
MCu3	−4.019	−3.829
MCu4	−3.673	−1.276
MCu5	−2.918	−1.137
MCu6	−1.783	−2.137
MCu7	−2.738	−3.613
MCu8	−5.625	−4.371
MCu9	−4.391	−2.172
MCu	−2.137	−3.008
MZn1	−5.469	−5.173
MZn2	−4.918	−4.371
MZn3	−4.467	−4.016
MZn4	−2.521	−1.276
MZn5	−1.957	−1.173
MZn6	−0.981	−1.864
MZn7	−1.852	−2.279
MZn8	−5.614	−4.537
MZn9	−3.584	−2.179
MZn	−3.383	−4.162
(Acorbose)	−14.78	−15.082

**Table 12:** Copper and Zinc metal complexes with functional groups.

Code	Copper Complexes	Zinc Complexes	R_1_	R_2_
Met1	MCu1	MZn1	H	C_6_H_5_
Met2	MCu2	MZn2	H	C_6_H_4_(OH)
Met3	MCu3	MZn3	H	CH=CH–C_6_H_5_
Met4	MCu4	MZn4	H	H
Met5	MCu5	MZn5	CH_3_	CH_3_
Met 6	MCu6	MZn6	CH_3_	C_6_H_5_
Met 7	MCu7	MZn7	C_6_H_5_	C_6_H_5_
Met 8	MCu8	MZn8	H	C_6_H_3_(OCH_3_)(OH)
Met 9	MCu9	MZn9	C_6_H_5_	CH(OH)C_6_H_5_

## Data Availability

The data used to support the findings of this study are included within the article.

## Supplementary Materials

The following supporting information can be downloaded at: https://www.mdpi.com/article/10.3390/molecules28031406/s1, Schemes S1–S3: The characterization schemes of all the metformin metabolites and metal complexes; Table S1: ADME predictions of Metformin metabolites and metal complexes; Table S2: 2D structures of all Metformin derivatives with respect to amylase enzyme; Table S3: 2D structures of all Metformin derivatives with respect to glucosidase enzyme; Table S4: 3D pose and geometries of all the metformin derivatives; Figure S1: Graphical representation of α-glucosidase activity of Met-1–Met-9; Figure S2: Graphical representation of α-glucosidase activity of MCu1–MCu9; Figure S3: Graphical representation of α-glucosidase activity of MZn1–MZn9; Figure S4: Graphical representation of α-amylase activity of Met1–Met9; Figure S5: Graphical representation of α-amylase activity of MCu1–MCu9; Figure S6: Graphical representation of α-amylase activity of MZn1-MZn9; Figures S7–S31: IR and NMR spectras of all the metformin metabolites and metal complexes.

## Sample Availability

Samples of the compounds are available from the authors.
