# Supplementary material for: A Comprehensive Approach to Derivatization: Elemental Composition, Biochemical, and In Silico Studies of Metformin Derivatives Containing Copper and Zinc Complexes
Source: Molecules. 2023 Feb 1;28(3):1406. doi: 10.3390/molecules28031406 (PMC9919699; doi:10.3390/molecules28031406)
Supplement: Supplementary file 1 [file molecules-28-01406-s001.zip › molecules-2153998-supplementary.pdf]

molecules

MDPI

Supplementary Materials

A Comprehensive Approach to Derivatization: Elemental Composition, Biochemical, and In Silico Studies of Metformin Derivatives Containing Copper and Zinc Complexes

Javed Ahmed [1], Mohsin Abbas Khan [1,*], Muhammad Ehsan Khalid [2], Irshad Ahmad [1], Irfan Pervaiz [3], Umair Khurshid [1,*], Saharish Khaliq [1], Kashif ur Rehman Khan [1], Muhammad Adeel Arshad [4], Ghadeer M. Albadrani [5], Ahmed E. Altyar [6,7], Amany A. Sayed [8] Mousa O. Germoush [9], and Mohamed M. Abdel-Daim [10,11]

[1] Department of Pharmaceutical Chemistry, Faculty of Pharmacy, The Islamia University of Bahawalpur, Bahawalpur 63100, Pakistan; javedpharmacist82@yahoo.com (J.A.); irshad.iub@hotmail.com (I.A.); saharish.convent@hotmail.com (S.K.); kashifur.rahman@iub.edu.pk (K.u.R.K.)
[2] Frontier Medical and Dental College, Abbottabad 22020, Pakistan; mehsankhalid786@gmail.com
[3] KITAAS, Khaldunia College of Pharmacy, Lahore 54000, Pakistan; irfanpharmacist@gmail.com
[4] Institute of Pharmacy, Faculty of Pharmaceutical and Allied Health Sciences, Lahore College for Women University, Lahore 54000, Pakistan; adeel.arshad@lcwu.edu.pk
[5] Department of Biology, College of Science, Princess Nourah bint Abdulrahman University, P.O. Box 84428, Riyadh 11671, Saudi Arabia; gmalbadrani@pnu.edu.sa
[6] Department of Pharmacy Practice, Faculty of Pharmacy, King Abdulaziz University, P.O. Box 80260, Jeddah 21589, Saudi Arabia; aealtyar@kau.edu.sa
[7] Pharmacy Program, Batterjee Medical College, P.O. Box 6231 Jeddah 21442, Saudi Arabia
[8] Zoology Department, Faculty of Science, Cairo University, Giza 12613, Egypt; amanyasayed@sci.cu.edu.eg
[9] Biology Department, College of Science, Jouf University, P.O. Box 2014, Sakaka, Saudi Arabia; mogermoush@ju.edu.sa
[10] Department of Pharmaceutical Sciences, Pharmacy Program, Batterjee Medical College, P.O. Box 6231 Jeddah 21442, Saudi Arabia; abdeldaim.m@vet.suez.edu.eg
[11] Pharmacology Department, Faculty of Veterinary Medicine, Suez Canal University, Ismailia 41522, Egypt
* Correspondence: lasharibloch@hotmail.com (M.A.K.); umair.khurshid@iub.edu.pk (U.K.)

Citation: Ahmed, J.; Khan, M.A.; Khalid, M.E.; Ahmad, I.; Pervaiz, I.; Khurshid, U.; Khaliq, S.; Khan, K.R.; Arshad, M.A.; Albadrani, G.M.; et al. A Comprehensive Approach to Derivatization: Elemental Composition, Biochemical, and In Silico Studies of Metformin Derivatives Containing Copper and Zinc Complexes. *Molecules* **2023**, *28*, 1406. https://doi.org/10.3390/molecules28031406

Academic Editors: Bilal Ahmed, Khan Mohammad Saghir and Mohammad Oves

Received: 26 December 2022
Revised: 21 January 2023
Accepted: 23 January 2023
Published: date

Copyright: © 2023 by the authors. Licensee MDPI, Basel, Switzerland. This article is an open access article distributed under the terms and conditions of the Creative Commons Attribution (CC BY) license (https://creativecommons.org/licenses/by/4.0/).

Abstract: The current study was designed to synthesis, characterize, and screen molecular and biological activities of different metformin derivatives that possess potent anti-diabetic potential with minimal side effects. Copper II (MCu1 – Mcu9) and Zinc II (MZn1 – MZn9) metal complexes of metformin base derivatives were synthesized by aromatic aldehydes and ketones through template reaction. The novel metal complexes were characterized through elemental analysis, physical state, melting point, physical appearance, Fourier transform infrared (FTIR) spectroscopy, UV-Visible (UV-Vis) spectroscopy, 1H Nuclear Magnetic Resonance (NMR) spectroscopy, 13C NMR spectroscopy. Biological activities of the synthesized derivatives were evaluated through α-amylase and α-glucosidase enzyme inhibitory screening as well as further confirmation by molecular simulation studies through Schrödinger. Enzyme inhibition assay (α-amylase & α-glucosidase) revealed that MET1, MET2, MET3, and MET8 showed comparable activities with the standard. Molecular simulation studies supported the results of the enzyme inhibition assay and showed a competitive docking score with the standard. The ADME properties of metformin derivatives were calculated by using Swiss ADME online web server. All derivatives follow Lipinski rule of five and the percentage of absorption can be calculated. The results of the present study suggested that metformin derivatives presented significant antidiabetic properties against both targeted enzymes and could serve as lead molecules for further research and development.

Keywords: Copper; Zinc; Metformin; FTIR; UV; NMR; Enzyme Inhibition Assay; Amylase; Glucosidase; Molecular Simulation; and Docking

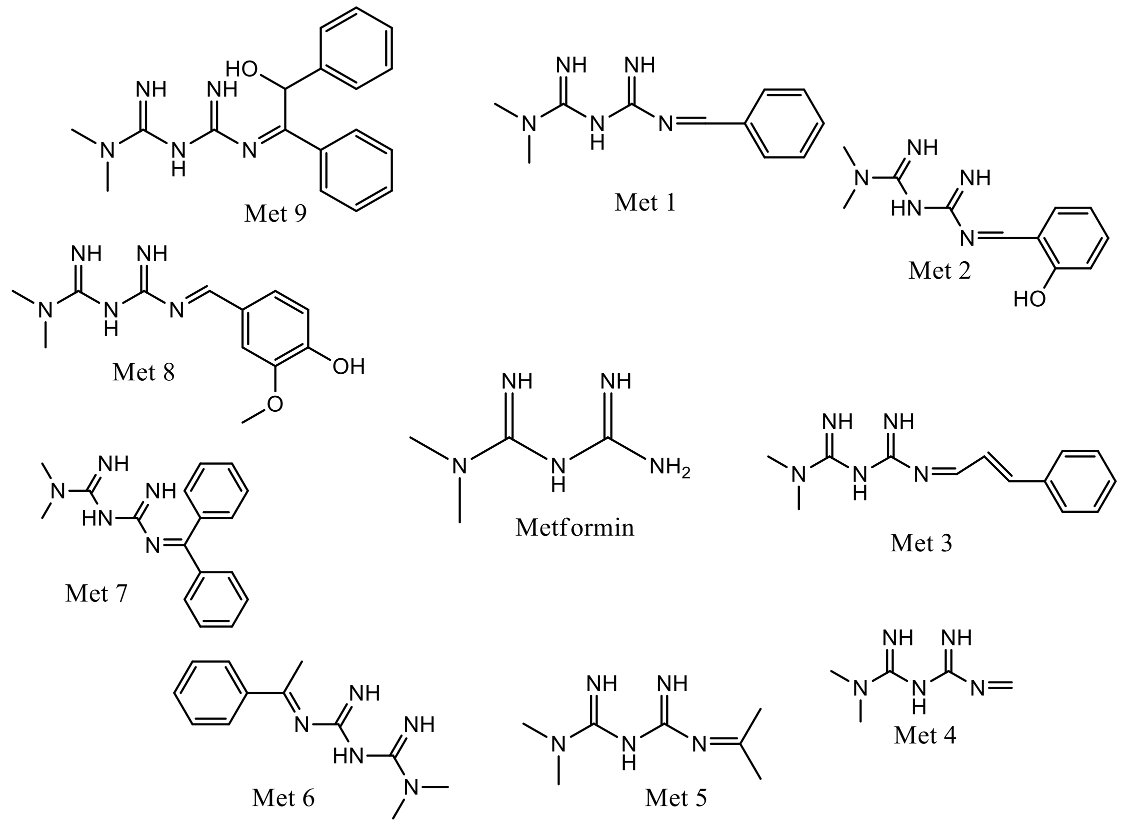

Scheme S1. Characterization of (Met1 - Met9).

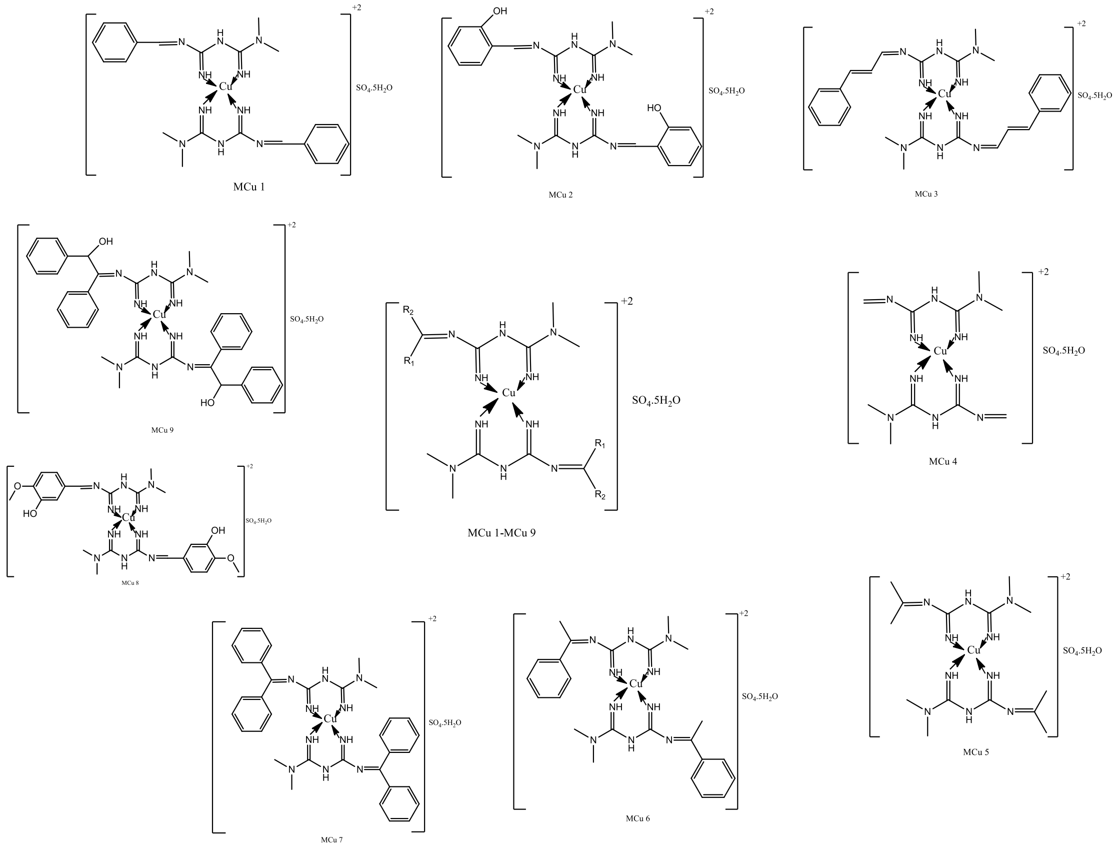

Scheme S2. Characterization of MCu1-MCu9.

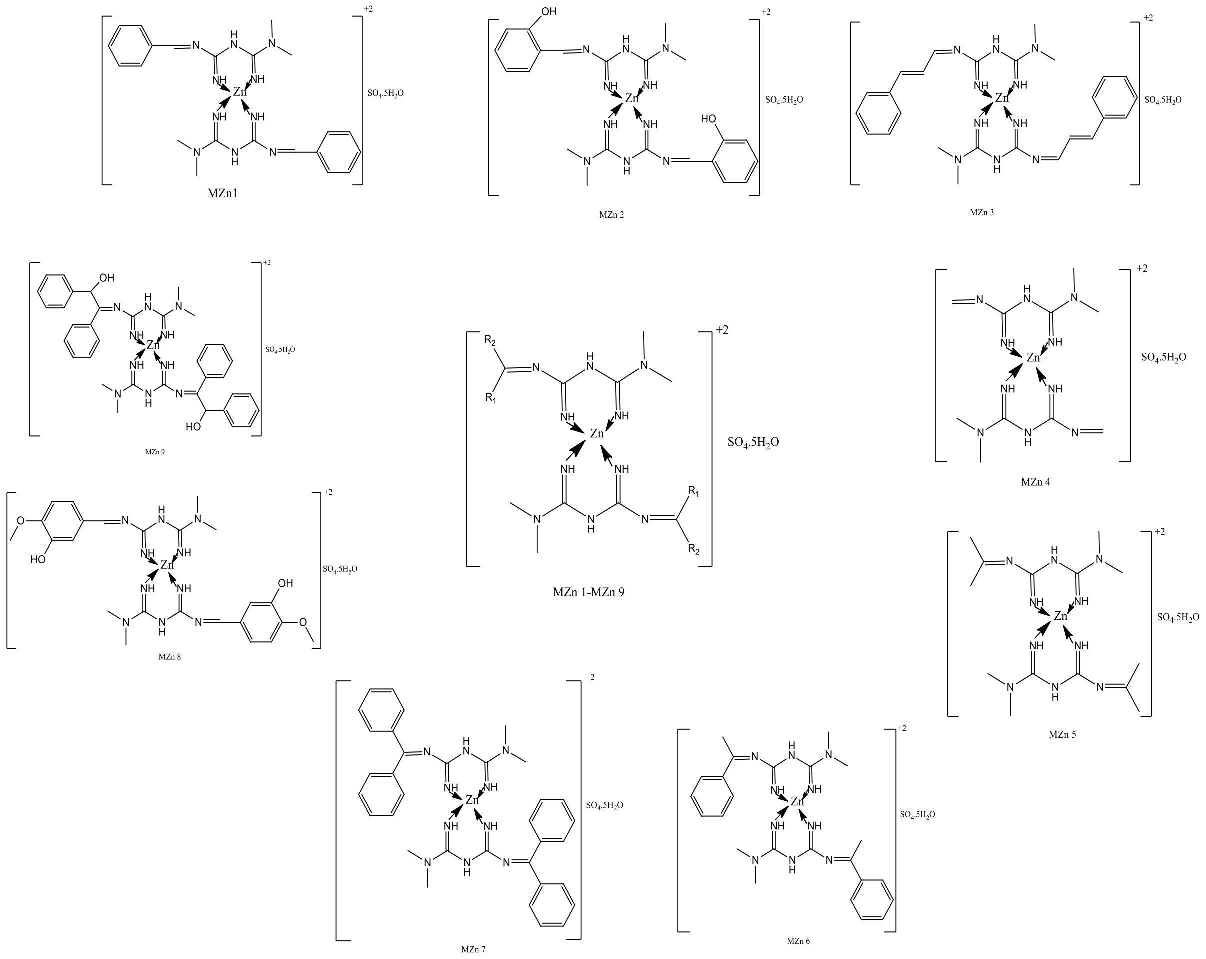

Scheme S3. Characterization of MZn1-MZn9.

Table S1. ADME predictions of Metformin metabolites and metal complexes.

Derivative code	3D Pose
MET-1	Molecule 1 (below)
MET-2	Molecule 2 (below)

MET-1

Molecule 1

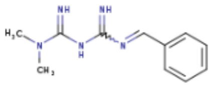

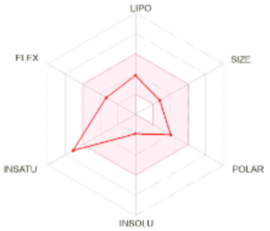

SMILES CN(C(=N)NC(=N)N=Cc1ccccc1)C

Physicochemical Properties	
Formula	C11H15N5
Molecular weight	217.27 g/mol
Num. heavy atoms	16
Num. arom. heavy atoms	6
Fraction Csp3	0.18
Num. rotatable bonds	5
Num. H-bond acceptors	3
Num. H-bond donors	3
Molar Refractivity	67.20
TPSA	75.33 Å²
**Lipophilicity**	
Log $P_{o/w}$ (iLOGP)	1.74
Log $P_{o/w}$ (XLOGP3)	1.24
Log $P_{o/w}$ (WLOGP)	1.13
Log $P_{o/w}$ (MLOGP)	1.88
Log $P_{o/w}$ (SILICOS-IT)	1.06
Consensus Log $P_{o/w}$	1.41

Water Solubility	
Log *S* (ESOL)	-1.92
Solubility	2.64e+00 mg/ml ; 1.21e-02 mol/l
Class	Very soluble
Log *S* (Ali)	-2.42
Solubility	8.26e-01 mg/ml ; 3.80e-03 mol/l
Class	Soluble
Log *S* (SILICOS-IT)	-2.85
Solubility	3.09e-01 mg/ml ; 1.42e-03 mol/l
Class	Soluble
**Pharmacokinetics**	
GI absorption	High
BBB permeant	No
P-gp substrate	No
CYP1A2 inhibitor	No
CYP2C19 inhibitor	No
CYP2C9 inhibitor	No
CYP2D6 inhibitor	No
CYP3A4 inhibitor	No
Log K_p (skin permeation)	-6.74 cm/s
**Druglikeness**	
Lipinski	Yes; 0 violation
Ghose	Yes
Veber	Yes
Egan	Yes
Muegge	Yes
Bioavailability Score	0.55
**Medicinal Chemistry**	
PAINS	0 alert
Brenk	2 alerts: imine_1, imine_2
Leadlikeness	No; 1 violation: MW<250
Synthetic accessibility	2.77

MET-2

Molecule 2

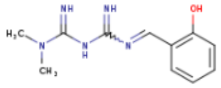

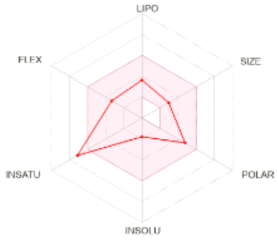

SMILES N=C(NC(=N)N(C)C)N=Cc1ccccc1O

Physicochemical Properties	
Formula	C11H15N5O
Molecular weight	233.27 g/mol
Num. heavy atoms	17
Num. arom. heavy atoms	6
Fraction Csp3	0.18
Num. rotatable bonds	5
Num. H-bond acceptors	4
Num. H-bond donors	4
Molar Refractivity	69.22
TPSA	95.56 Å²
**Lipophilicity**	
Log $P_{o/w}$ (iLOGP)	1.51
Log $P_{o/w}$ (XLOGP3)	0.89
Log $P_{o/w}$ (WLOGP)	0.83
Log $P_{o/w}$ (MLOGP)	1.33
Log $P_{o/w}$ (SILICOS-IT)	0.61
Consensus Log $P_{o/w}$	1.03

Water Solubility	
Log *S* (ESOL)	-1.78
Solubility	3.89e+00 mg/ml ; 1.67e-02 mol/l
Class	Very soluble
Log *S* (Ali)	-2.48
Solubility	7.70e-01 mg/ml ; 3.30e-03 mol/l
Class	Soluble
Log *S* (SILICOS-IT)	-2.27
Solubility	1.25e+00 mg/ml ; 5.35e-03 mol/l
Class	Soluble
**Pharmacokinetics**	
GI absorption	High
BBB permeant	No
P-gp substrate	No
CYP1A2 inhibitor	No
CYP2C19 inhibitor	No
CYP2C9 inhibitor	No
CYP2D6 inhibitor	No
CYP3A4 inhibitor	No
Log K_p (skin permeation)	-7.09 cm/s
**Druglikeness**	
Lipinski	Yes; 0 violation
Ghose	Yes
Veber	Yes
Egan	Yes
Muegge	Yes
Bioavailability Score	0.55
**Medicinal Chemistry**	
PAINS	0 alert
Brenk	2 alerts: imine_1, imine_2
Leadlikeness	No; 1 violation: MW<250
Synthetic accessibility	2.81

MET-3

Molecule 3

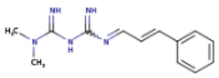

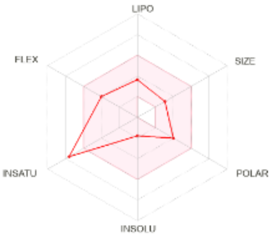

SMILES CN(C(=N)NC(=N)/N=C\C=C\c1ccccc1)C

Physicochemical Properties	
Formula	C13H17N5
Molecular weight	243.31 g/mol
Num. heavy atoms	18
Num. arom. heavy atoms	6
Fraction Csp3	0.15
Num. rotatable bonds	6
Num. H-bond acceptors	3
Num. H-bond donors	3
Molar Refractivity	77.13
TPSA	75.33 Å²

Lipophilicity	
Log $P_{o/w}$ (iLOGP)	1.51
Log $P_{o/w}$ (XLOGP3)	0.89
Log $P_{o/w}$ (WLOGP)	1.68
Log $P_{o/w}$ (MLOGP)	1.33
Log $P_{o/w}$ (SILICOS-IT)	0.61
Consensus Log $P_{o/w}$	1.03

Water Solubility	
Log *S* (ESOL)	-1.78
Solubility	3.89e+00 mg/ml ; 1.67e-02 mol/l
Class	Very soluble
Log *S* (Ali)	-2.48
Solubility	7.70e-01 mg/ml ; 3.30e-03 mol/l
Class	Soluble
Log *S* (SILICOS-IT)	-2.27
Solubility	1.25e+00 mg/ml ; 5.35e-03 mol/l
Class	Soluble

Pharmacokinetics	
GI absorption	High
BBB permeant	No
P-gp substrate	No
CYP1A2 inhibitor	No
CYP2C19 inhibitor	No
CYP2C9 inhibitor	No
CYP2D6 inhibitor	No
CYP3A4 inhibitor	No
Log K_p (skin permeation)	-7.09 cm/s

Druglikeness	
Lipinski	Yes; 0 violation
Ghose	Yes
Veber	Yes
Egan	Yes
Muegge	Yes
Bioavailability Score	0.55

Medicinal Chemistry	
PAINS	0 alert
Brenk	2 alerts: imine_1, imine_2
Leadlikeness	No; 1 violation: MW<250
Synthetic accessibility	2.81

MET-4

Molecule 4

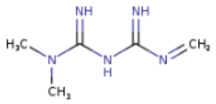

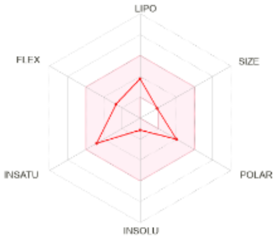

SMILES C=NC(=N)NC(=N)N(C)C

Physicochemical Properties	
Formula	C5H11N5
Molecular weight	141.17 g/mol
Num. heavy atoms	10
Num. arom. heavy atoms	0
Fraction Csp3	0.40
Num. rotatable bonds	4
Num. H-bond acceptors	3
Num. H-bond donors	3
Molar Refractivity	42.71
TPSA	75.33 Å²

Lipophilicity	
Log $P_{o/w}$ (iLOGP)	0.06
Log $P_{o/w}$ (XLOGP3)	1.10
Log $P_{o/w}$ (WLOGP)	-0.29
Log $P_{o/w}$ (MLOGP)	0.16
Log $P_{o/w}$ (SILICOS-IT)	-0.52
Consensus Log $P_{o/w}$	0.10

Water Solubility	
Log *S* (ESOL)	-1.14
Solubility	1.01e+01 mg/ml ; 7.17e-02 mol/l
Class	Very soluble
Log *S* (Ali)	-2.27
Solubility	7.50e-01 mg/ml ; 5.31e-03 mol/l
Class	Soluble
Log *S* (SILICOS-IT)	-0.65
Solubility	3.14e+01 mg/ml ; 2.22e-01 mol/l
Class	Soluble

Pharmacokinetics	
GI absorption	High
BBB permeant	No
P-gp substrate	No
CYP1A2 inhibitor	No
CYP2C19 inhibitor	No
CYP2C9 inhibitor	No
CYP2D6 inhibitor	No
CYP3A4 inhibitor	No
Log K_p (skin permeation)	-6.38 cm/s

Druglikeness	
Lipinski	Yes; 0 violation
Ghose	No; 1 violation: MW<160
Veber	Yes
Egan	Yes
Muegge	No; 1 violation: MW<200
Bioavailability Score	0.55

Medicinal Chemistry	
PAINS	0 alert
Brenk	2 alerts: imine_1, imine_2
Leadlikeness	No; 1 violation: MW<250
Synthetic accessibility	3.41

MET-5

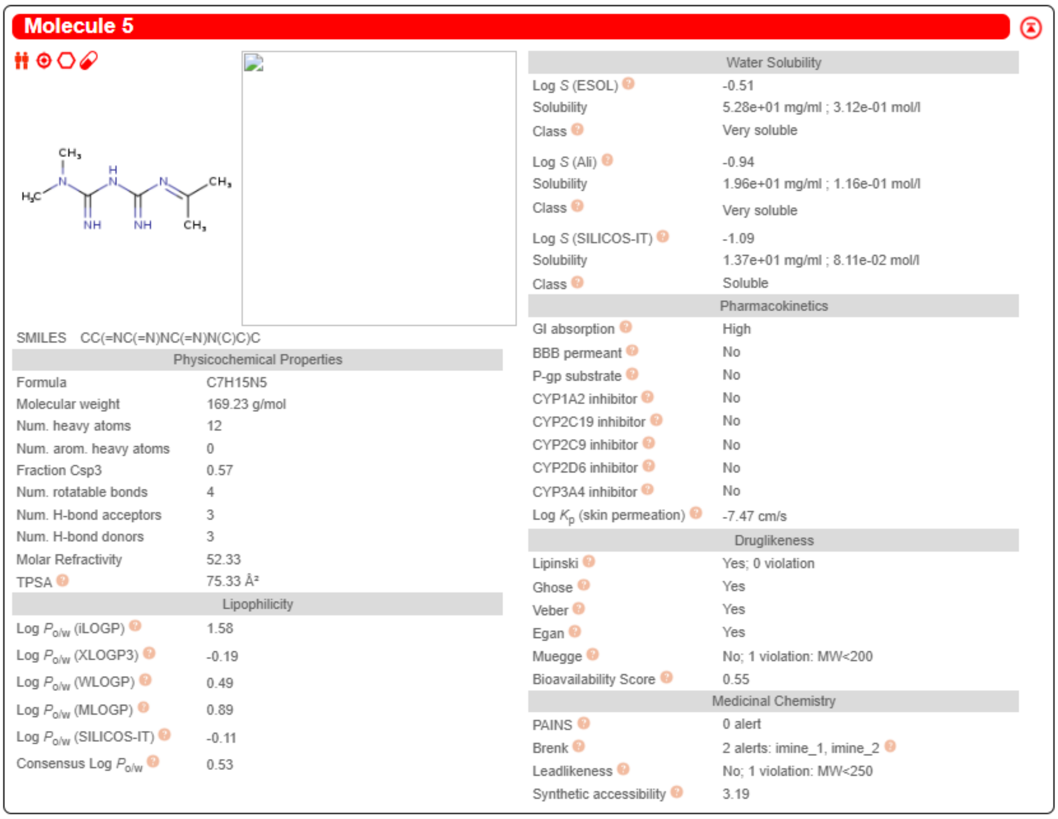

Molecule 5

SMILES CC(=NC(=N)NC(=N)N(C)C)C

Physicochemical Properties	
Formula	C7H15N5
Molecular weight	169.23 g/mol
Num. heavy atoms	12
Num. arom. heavy atoms	0
Fraction Csp3	0.57
Num. rotatable bonds	4
Num. H-bond acceptors	3
Num. H-bond donors	3
Molar Refractivity	52.33
TPSA	75.33 Å²
**Lipophilicity**	
Log $P_{o/w}$ (iLOGP)	1.58
Log $P_{o/w}$ (XLOGP3)	-0.19
Log $P_{o/w}$ (WLOGP)	0.49
Log $P_{o/w}$ (MLOGP)	0.89
Log $P_{o/w}$ (SILICOS-IT)	-0.11
Consensus Log $P_{o/w}$	0.53

Water Solubility	
Log *S* (ESOL)	-0.51
Solubility	5.28e+01 mg/ml ; 3.12e-01 mol/l
Class	Very soluble
Log *S* (Ali)	-0.94
Solubility	1.96e+01 mg/ml ; 1.16e-01 mol/l
Class	Very soluble
Log *S* (SILICOS-IT)	-1.09
Solubility	1.37e+01 mg/ml ; 8.11e-02 mol/l
Class	Soluble
**Pharmacokinetics**	
GI absorption	High
BBB permeant	No
P-gp substrate	No
CYP1A2 inhibitor	No
CYP2C19 inhibitor	No
CYP2C9 inhibitor	No
CYP2D6 inhibitor	No
CYP3A4 inhibitor	No
Log K_p (skin permeation)	-7.47 cm/s
**Druglikeness**	
Lipinski	Yes; 0 violation
Ghose	Yes
Veber	Yes
Egan	Yes
Muegge	No; 1 violation: MW<200
Bioavailability Score	0.55
**Medicinal Chemistry**	
PAINS	0 alert
Brenk	2 alerts: imine_1, imine_2
Leadlikeness	No; 1 violation: MW<250
Synthetic accessibility	3.19

MET-6

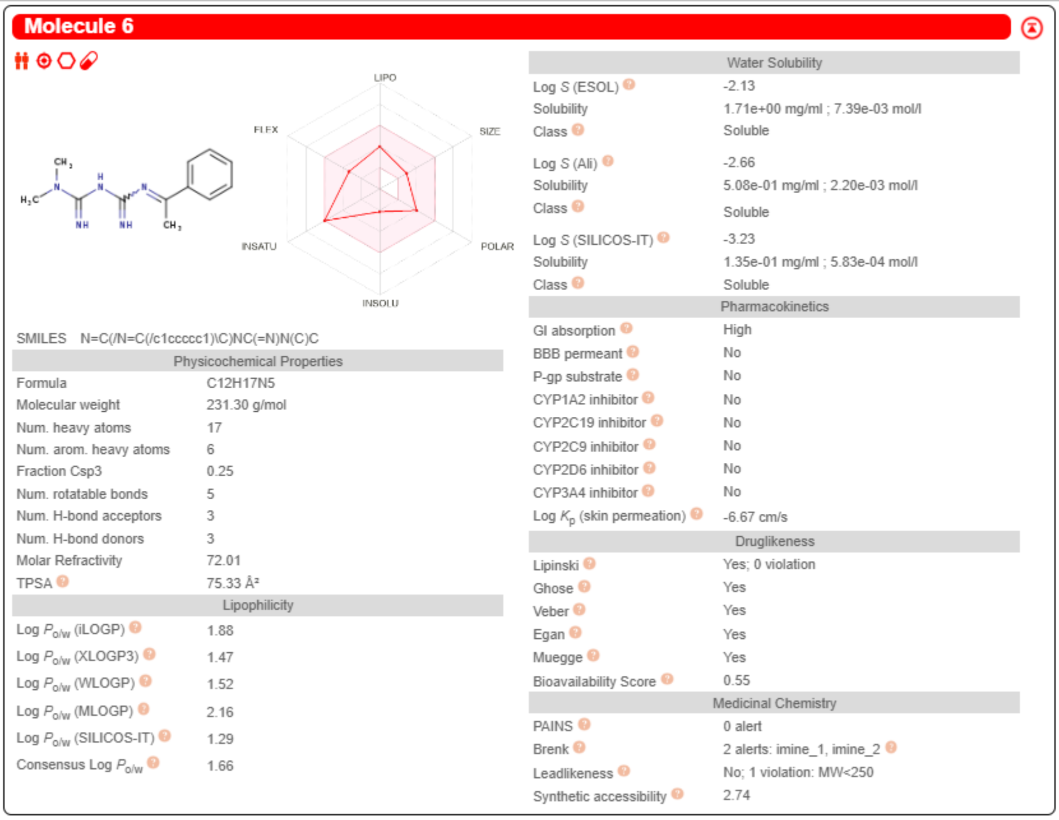

Molecule 6

SMILES N=C(/N=C(/c1ccccc1)\C)NC(=N)N(C)C

Physicochemical Properties	
Formula	C12H17N5
Molecular weight	231.30 g/mol
Num. heavy atoms	17
Num. arom. heavy atoms	6
Fraction Csp3	0.25
Num. rotatable bonds	5
Num. H-bond acceptors	3
Num. H-bond donors	3
Molar Refractivity	72.01
TPSA	75.33 Å²
**Lipophilicity**	
Log $P_{o/w}$ (iLOGP)	1.88
Log $P_{o/w}$ (XLOGP3)	1.47
Log $P_{o/w}$ (WLOGP)	1.52
Log $P_{o/w}$ (MLOGP)	2.16
Log $P_{o/w}$ (SILICOS-IT)	1.29
Consensus Log $P_{o/w}$	1.66

Water Solubility	
Log *S* (ESOL)	-2.13
Solubility	1.71e+00 mg/ml ; 7.39e-03 mol/l
Class	Soluble
Log *S* (Ali)	-2.66
Solubility	5.08e-01 mg/ml ; 2.20e-03 mol/l
Class	Soluble
Log *S* (SILICOS-IT)	-3.23
Solubility	1.35e-01 mg/ml ; 5.83e-04 mol/l
Class	Soluble
**Pharmacokinetics**	
GI absorption	High
BBB permeant	No
P-gp substrate	No
CYP1A2 inhibitor	No
CYP2C19 inhibitor	No
CYP2C9 inhibitor	No
CYP2D6 inhibitor	No
CYP3A4 inhibitor	No
Log K_p (skin permeation)	-6.67 cm/s
**Druglikeness**	
Lipinski	Yes; 0 violation
Ghose	Yes
Veber	Yes
Egan	Yes
Muegge	Yes
Bioavailability Score	0.55
**Medicinal Chemistry**	
PAINS	0 alert
Brenk	2 alerts: imine_1, imine_2
Leadlikeness	No; 1 violation: MW<250
Synthetic accessibility	2.74

MET-7

Molecule 7

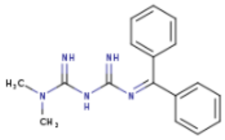

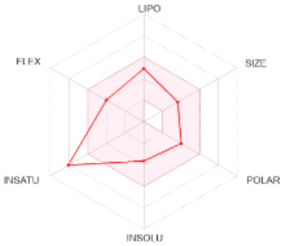

SMILES N=C(N=C(c1ccccc1)c1ccccc1)NC(=N)N(C)C

Physicochemical Properties	
Formula	C17H19N5
Molecular weight	293.37 g/mol
Num. heavy atoms	22
Num. arom. heavy atoms	12
Fraction Csp3	0.12
Num. rotatable bonds	6
Num. H-bond acceptors	3
Num. H-bond donors	3
Molar Refractivity	91.69
TPSA	75.33 Å²

Lipophilicity	
Log $P_{o/w}$ (iLOGP)	2.36
Log $P_{o/w}$ (XLOGP3)	3.12
Log $P_{o/w}$ (WLOGP)	2.54
Log $P_{o/w}$ (MLOGP)	3.21
Log $P_{o/w}$ (SILICOS-IT)	2.51
Consensus Log $P_{o/w}$	2.75

Water Solubility	
Log *S* (ESOL)	-3.63
Solubility	6.84e-02 mg/ml ; 2.33e-04 mol/l
Class	Soluble
Log *S* (Ali)	-4.37
Solubility	1.25e-02 mg/ml ; 4.26e-05 mol/l
Class	Moderately soluble
Log *S* (SILICOS-IT)	-5.35
Solubility	1.31e-03 mg/ml ; 4.48e-06 mol/l
Class	Moderately soluble

Pharmacokinetics	
GI absorption	High
BBB permeant	Yes
P-gp substrate	No
CYP1A2 inhibitor	No
CYP2C19 inhibitor	No
CYP2C9 inhibitor	No
CYP2D6 inhibitor	No
CYP3A4 inhibitor	No
Log K_p (skin permeation)	-5.87 cm/s

Druglikeness	
Lipinski	Yes; 0 violation
Ghose	Yes
Veber	Yes
Egan	Yes
Muegge	Yes
Bioavailability Score	0.55

Medicinal Chemistry	
PAINS	0 alert
Brenk	2 alerts: imine_1, imine_2
Leadlikeness	Yes
Synthetic accessibility	3.08

MET-8

Molecule 8

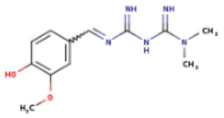

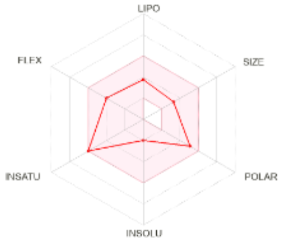

SMILES COc1cc(/C=N/C(=N)NC(=N)N(C)C)ccc1O

Physicochemical Properties	
Formula	C12H17N5O2
Molecular weight	263.30 g/mol
Num. heavy atoms	19
Num. arom. heavy atoms	6
Fraction Csp3	0.25
Num. rotatable bonds	6
Num. H-bond acceptors	5
Num. H-bond donors	4
Molar Refractivity	75.72
TPSA	104.79 Å²

Lipophilicity	
Log $P_{o/w}$ (iLOGP)	2.03
Log $P_{o/w}$ (XLOGP3)	1.07
Log $P_{o/w}$ (WLOGP)	0.84
Log $P_{o/w}$ (MLOGP)	1.08
Log $P_{o/w}$ (SILICOS-IT)	0.70
Consensus Log $P_{o/w}$	1.14

Water Solubility	
Log *S* (ESOL)	-1.98
Solubility	2.73e+00 mg/ml ; 1.04e-02 mol/l
Class	Very soluble
Log *S* (Ali)	-2.86
Solubility	3.62e-01 mg/ml ; 1.37e-03 mol/l
Class	Soluble
Log *S* (SILICOS-IT)	-2.40
Solubility	1.06e+00 mg/ml ; 4.03e-03 mol/l
Class	Soluble

Pharmacokinetics	
GI absorption	High
BBB permeant	No
P-gp substrate	No
CYP1A2 inhibitor	No
CYP2C19 inhibitor	No
CYP2C9 inhibitor	No
CYP2D6 inhibitor	No
CYP3A4 inhibitor	No
Log K_p (skin permeation)	-7.15 cm/s

Druglikeness	
Lipinski	Yes; 0 violation
Ghose	Yes
Veber	Yes
Egan	Yes
Muegge	Yes
Bioavailability Score	0.55

Medicinal Chemistry	
PAINS	0 alert
Brenk	2 alerts: imine_1, imine_2
Leadlikeness	Yes
Synthetic accessibility	2.81

MET-9

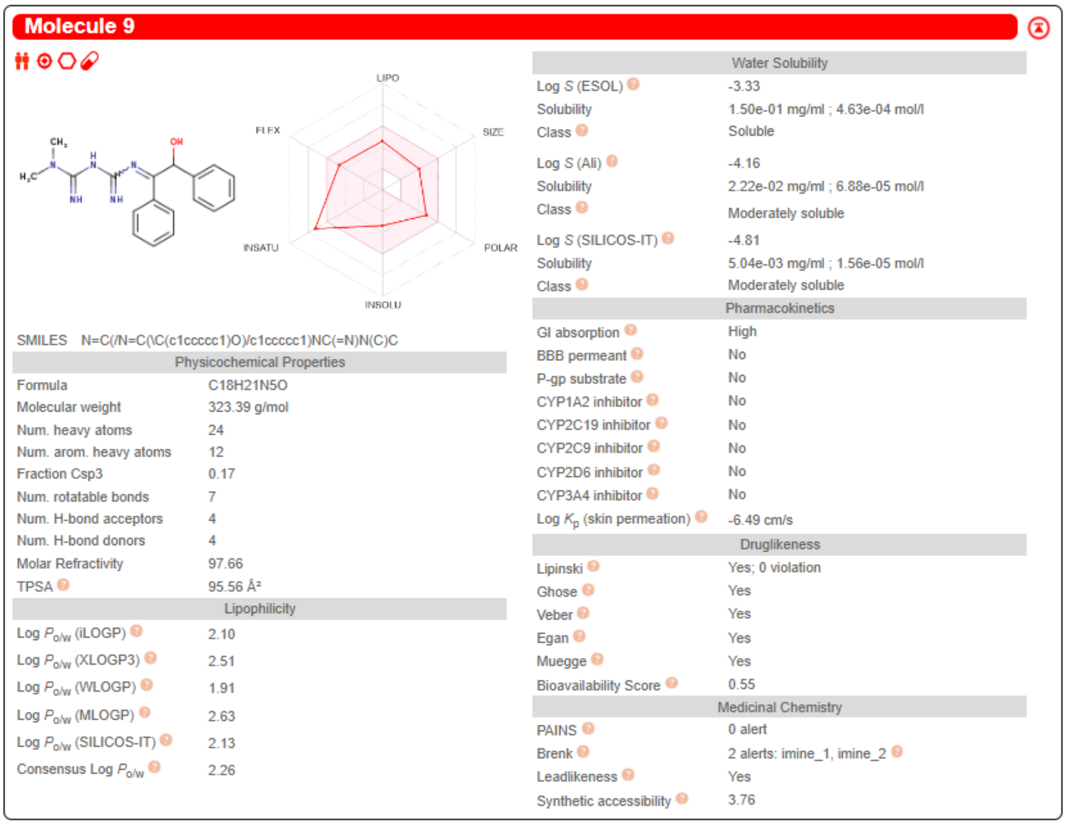

Molecule 9

SMILES N=C(/N=C(\C(c1ccccc1)O)/c1ccccc1)NC(=N)N(C)C

Physicochemical Properties	
Formula	C18H21N5O
Molecular weight	323.39 g/mol
Num. heavy atoms	24
Num. arom. heavy atoms	12
Fraction Csp3	0.17
Num. rotatable bonds	7
Num. H-bond acceptors	4
Num. H-bond donors	4
Molar Refractivity	97.66
TPSA	95.56 Å²

Lipophilicity	
Log $P_{o/w}$ (iLOGP)	2.10
Log $P_{o/w}$ (XLOGP3)	2.51
Log $P_{o/w}$ (WLOGP)	1.91
Log $P_{o/w}$ (MLOGP)	2.63
Log $P_{o/w}$ (SILICOS-IT)	2.13
Consensus Log $P_{o/w}$	2.26

Water Solubility	
Log *S* (ESOL)	-3.33
Solubility	1.50e-01 mg/ml ; 4.63e-04 mol/l
Class	Soluble
Log *S* (Ali)	-4.16
Solubility	2.22e-02 mg/ml ; 6.88e-05 mol/l
Class	Moderately soluble
Log *S* (SILICOS-IT)	-4.81
Solubility	5.04e-03 mg/ml ; 1.56e-05 mol/l
Class	Moderately soluble

Pharmacokinetics	
GI absorption	High
BBB permeant	No
P-gp substrate	No
CYP1A2 inhibitor	No
CYP2C19 inhibitor	No
CYP2C9 inhibitor	No
CYP2D6 inhibitor	No
CYP3A4 inhibitor	No
Log K_p (skin permeation)	-6.49 cm/s

Druglikeness	
Lipinski	Yes; 0 violation
Ghose	Yes
Veber	Yes
Egan	Yes
Muegge	Yes
Bioavailability Score	0.55

Medicinal Chemistry	
PAINS	0 alert
Brenk	2 alerts: imine_1, imine_2
Leadlikeness	Yes
Synthetic accessibility	3.76

MET

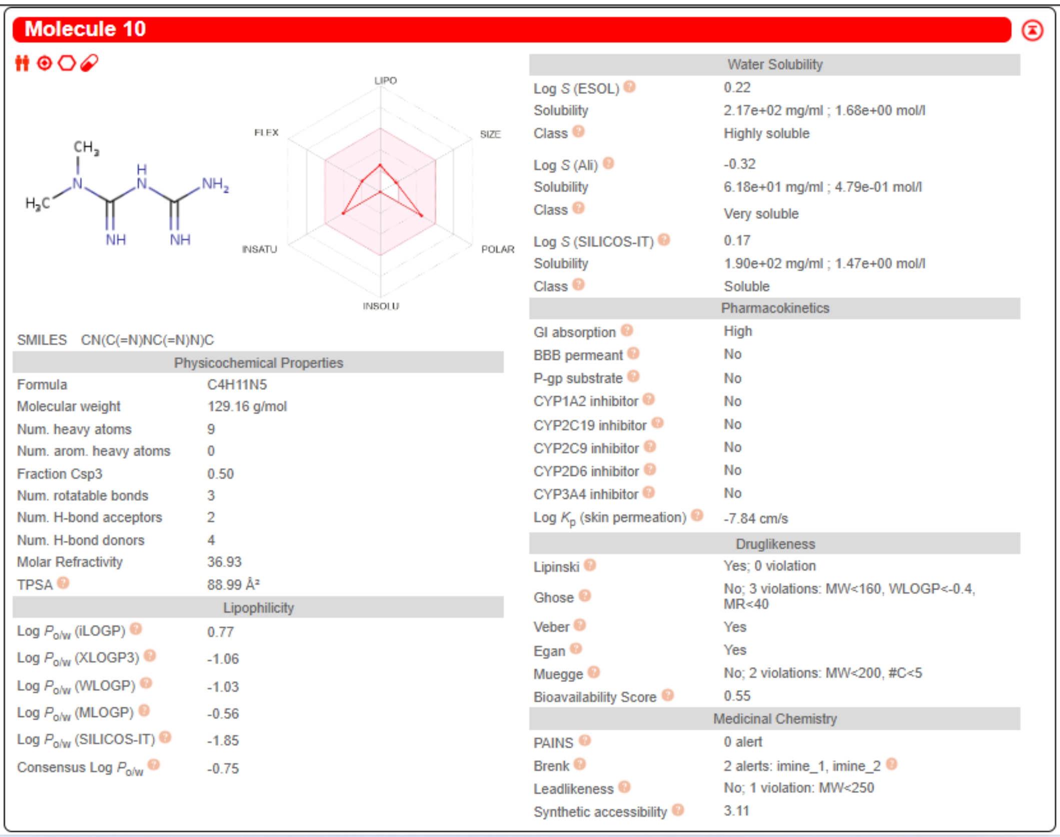

Molecule 10

SMILES CN(C(=N)NC(=N)N)C

Physicochemical Properties	
Formula	C4H11N5
Molecular weight	129.16 g/mol
Num. heavy atoms	9
Num. arom. heavy atoms	0
Fraction Csp3	0.50
Num. rotatable bonds	3
Num. H-bond acceptors	2
Num. H-bond donors	4
Molar Refractivity	36.93
TPSA	88.99 Å²

Lipophilicity	
Log $P_{o/w}$ (iLOGP)	0.77
Log $P_{o/w}$ (XLOGP3)	-1.06
Log $P_{o/w}$ (WLOGP)	-1.03
Log $P_{o/w}$ (MLOGP)	-0.56
Log $P_{o/w}$ (SILICOS-IT)	-1.85
Consensus Log $P_{o/w}$	-0.75

Water Solubility	
Log *S* (ESOL)	0.22
Solubility	2.17e+02 mg/ml ; 1.68e+00 mol/l
Class	Highly soluble
Log *S* (Ali)	-0.32
Solubility	6.18e+01 mg/ml ; 4.79e-01 mol/l
Class	Very soluble
Log *S* (SILICOS-IT)	0.17
Solubility	1.90e+02 mg/ml ; 1.47e+00 mol/l
Class	Soluble

Pharmacokinetics	
GI absorption	High
BBB permeant	No
P-gp substrate	No
CYP1A2 inhibitor	No
CYP2C19 inhibitor	No
CYP2C9 inhibitor	No
CYP2D6 inhibitor	No
CYP3A4 inhibitor	No
Log K_p (skin permeation)	-7.84 cm/s

Druglikeness	
Lipinski	Yes; 0 violation
Ghose	No; 3 violations: MW<160, WLOGP<-0.4, MR<40
Veber	Yes
Egan	Yes
Muegge	No; 2 violations: MW<200, #C<5
Bioavailability Score	0.55

Medicinal Chemistry	
PAINS	0 alert
Brenk	2 alerts: imine_1, imine_2
Leadlikeness	No; 1 violation: MW<250
Synthetic accessibility	3.11

MZN-1

Molecule 1

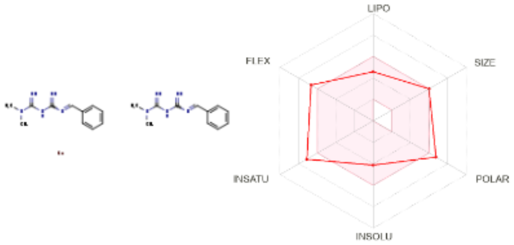

SMILES CN(C(=N)NC(=N)N=Cc1ccccc1)C.CN(C(=N)NC(=N)N=Cc1ccccc1)C.[Zn]

Physicochemical Properties	
Formula	C22H30N10Zn
Molecular weight	499.92 g/mol
Num. heavy atoms	33
Num. arom. heavy atoms	12
Fraction Csp3	0.18
Num. rotatable bonds	10
Num. H-bond acceptors	6
Num. H-bond donors	6
Molar Refractivity	134.40
TPSA	150.66 Å²
**Lipophilicity**	
Log $P_{o/w}$ (iLOGP)	-44.47
Log $P_{o/w}$ (XLOGP3)	2.49
Log $P_{o/w}$ (WLOGP)	2.25
Log $P_{o/w}$ (MLOGP)	3.03
Log $P_{o/w}$ (SILICOS-IT)	1.06
Consensus Log $P_{o/w}$	-7.13

Water Solubility	
Log *S* (ESOL)	-4.12
Solubility	3.82e-02 mg/ml ; 7.63e-05 mol/l
Class	Moderately soluble
Log *S* (Ali)	-5.30
Solubility	2.51e-03 mg/ml ; 5.02e-06 mol/l
Class	Moderately soluble
Log *S* (SILICOS-IT)	-2.85
Solubility	7.11e-01 mg/ml ; 1.42e-03 mol/l
Class	Soluble
**Pharmacokinetics**	
GI absorption	Low
BBB permeant	No
P-gp substrate	Yes
CYP1A2 inhibitor	No
CYP2C19 inhibitor	No
CYP2C9 inhibitor	No
CYP2D6 inhibitor	No
CYP3A4 inhibitor	No
Log K_p (skin permeation)	-7.58 cm/s
**Druglikeness**	
Lipinski	Yes; 1 violation: NHorOH>5
Ghose	No; 2 violations: MW>480, MR>130
Veber	No; 1 violation: TPSA>140
Egan	No; 1 violation: TPSA>131.6
Muegge	No; 2 violations: TPSA>150, H-don>5
Bioavailability Score	0.55
**Medicinal Chemistry**	
PAINS	0 alert
Brenk	3 alerts: heavy_metal, imine_1, imine_2
Leadlikeness	No; 2 violations: MW>350, Rotors>7
Synthetic accessibility	3.96

MZN-2

Molecule 2

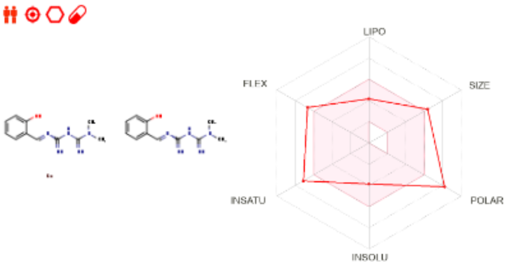

SMILES N=C(NC(=N)N(C)C)N=Cc1ccccc1O.N=C(NC(=N)N(C)C)N=Cc1ccccc1O.[Zn]

Physicochemical Properties	
Formula	C22H30N10O2Zn
Molecular weight	531.92 g/mol
Num. heavy atoms	35
Num. arom. heavy atoms	12
Fraction Csp3	0.18
Num. rotatable bonds	10
Num. H-bond acceptors	8
Num. H-bond donors	8
Molar Refractivity	138.45
TPSA	191.12 Å²
**Lipophilicity**	
Log $P_{o/w}$ (iLOGP)	-45.93
Log $P_{o/w}$ (XLOGP3)	1.78
Log $P_{o/w}$ (WLOGP)	1.66
Log $P_{o/w}$ (MLOGP)	1.23
Log $P_{o/w}$ (SILICOS-IT)	0.61
Consensus Log $P_{o/w}$	-8.13

Water Solubility	
Log *S* (ESOL)	-3.85
Solubility	7.46e-02 mg/ml ; 1.40e-04 mol/l
Class	Soluble
Log *S* (Ali)	-5.41
Solubility	2.06e-03 mg/ml ; 3.87e-06 mol/l
Class	Moderately soluble
Log *S* (SILICOS-IT)	-2.27
Solubility	2.84e+00 mg/ml ; 5.35e-03 mol/l
Class	Soluble
**Pharmacokinetics**	
GI absorption	Low
BBB permeant	No
P-gp substrate	Yes
CYP1A2 inhibitor	No
CYP2C19 inhibitor	No
CYP2C9 inhibitor	No
CYP2D6 inhibitor	No
CYP3A4 inhibitor	No
Log K_p (skin permeation)	-8.28 cm/s
**Druglikeness**	
Lipinski	No; 3 violations: MW>500, NorO>10, NHorOH>5
Ghose	No; 2 violations: MW>480, MR>130
Veber	No; 1 violation: TPSA>140
Egan	No; 1 violation: TPSA>131.6
Muegge	No; 2 violations: TPSA>150, H-don>5
Bioavailability Score	0.17
**Medicinal Chemistry**	
PAINS	0 alert
Brenk	3 alerts: heavy_metal, imine_1, imine_2
Leadlikeness	No; 2 violations: MW>350, Rotors>7
Synthetic accessibility	4.04

MZN-3

Molecule 3

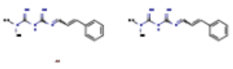

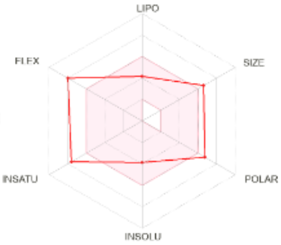

SMILES CN(C(=N)NC(=N)/N=C\C=C\c1ccccc1)C.CN(C(=N)NC(=N)/N=C\C=C\c1ccccc1)C.[Zn]

Physicochemical Properties	
Formula	C26H34N10Zn
Molecular weight	552.00 g/mol
Num. heavy atoms	37
Num. arom. heavy atoms	12
Fraction Csp3	0.15
Num. rotatable bonds	12
Num. H-bond acceptors	6
Num. H-bond donors	6
Molar Refractivity	154.26
TPSA	150.66 Å²
**Lipophilicity**	
Log $P_{o/w}$ (iLOGP)	-45.93
Log $P_{o/w}$ (XLOGP3)	1.78
Log $P_{o/w}$ (WLOGP)	3.36
Log $P_{o/w}$ (MLOGP)	1.23
Log $P_{o/w}$ (SILICOS-IT)	0.61
Consensus Log $P_{o/w}$	-8.13

Water Solubility	
Log *S* (ESOL)	-3.85
Solubility	7.46e-02 mg/ml ; 1.40e-04 mol/l
Class	Soluble
Log *S* (Ali)	-5.41
Solubility	2.06e-03 mg/ml ; 3.87e-06 mol/l
Class	Moderately soluble
Log *S* (SILICOS-IT)	-2.27
Solubility	2.84e+00 mg/ml ; 5.35e-03 mol/l
Class	Soluble
**Pharmacokinetics**	
GI absorption	Low
BBB permeant	No
P-gp substrate	Yes
CYP1A2 inhibitor	No
CYP2C19 inhibitor	No
CYP2C9 inhibitor	No
CYP2D6 inhibitor	No
CYP3A4 inhibitor	No
Log K_p (skin permeation)	-8.28 cm/s
**Druglikeness**	
Lipinski	No; 3 violations: MW>500, NorO>10, NHorOH>5
Ghose	No; 2 violations: MW>480, MR>130
Veber	No; 1 violation: TPSA>140
Egan	No; 1 violation: TPSA>131.6
Muegge	No; 2 violations: TPSA>150, H-don>5
Bioavailability Score	0.17
**Medicinal Chemistry**	
PAINS	0 alert
Brenk	3 alerts: heavy_metal, imine_1, imine_2
Leadlikeness	No; 2 violations: MW>350, Rotors>7
Synthetic accessibility	4.04

MZN-4

Molecule 4

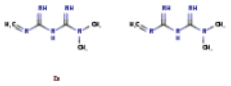

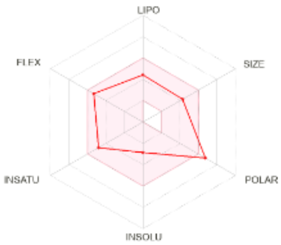

SMILES C=NC(=N)NC(=N)N(C)C.C=NC(=N)NC(=N)N(C)C.[Zn]

Physicochemical Properties	
Formula	C10H22N10Zn
Molecular weight	347.73 g/mol
Num. heavy atoms	21
Num. arom. heavy atoms	0
Fraction Csp3	0.40
Num. rotatable bonds	8
Num. H-bond acceptors	6
Num. H-bond donors	6
Molar Refractivity	85.43
TPSA	150.66 Å²
**Lipophilicity**	
Log $P_{o/w}$ (iLOGP)	-47.13
Log $P_{o/w}$ (XLOGP3)	2.21
Log $P_{o/w}$ (WLOGP)	-0.59
Log $P_{o/w}$ (MLOGP)	0.48
Log $P_{o/w}$ (SILICOS-IT)	-0.52
Consensus Log $P_{o/w}$	-9.11

Water Solubility	
Log *S* (ESOL)	-2.86
Solubility	4.80e-01 mg/ml ; 1.38e-03 mol/l
Class	Soluble
Log *S* (Ali)	-5.01
Solubility	3.41e-03 mg/ml ; 9.81e-06 mol/l
Class	Moderately soluble
Log *S* (SILICOS-IT)	-0.65
Solubility	7.73e+01 mg/ml ; 2.22e-01 mol/l
Class	Soluble
**Pharmacokinetics**	
GI absorption	Low
BBB permeant	No
P-gp substrate	Yes
CYP1A2 inhibitor	No
CYP2C19 inhibitor	No
CYP2C9 inhibitor	No
CYP2D6 inhibitor	No
CYP3A4 inhibitor	No
Log K_p (skin permeation)	-6.85 cm/s
**Druglikeness**	
Lipinski	Yes; 1 violation: NHorOH>5
Ghose	No; 1 violation: WLOGP<-0.4
Veber	No; 1 violation: TPSA>140
Egan	No; 1 violation: TPSA>131.6
Muegge	No; 2 violations: TPSA>150, H-don>5
Bioavailability Score	0.55
**Medicinal Chemistry**	
PAINS	0 alert
Brenk	3 alerts: heavy_metal, imine_1, imine_2
Leadlikeness	No; 1 violation: Rotors>7
Synthetic accessibility	4.14

MZN-5

Molecule 5

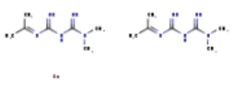

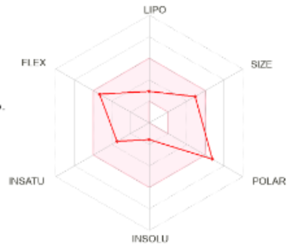

SMILES CC(=NC(=N)NC(=N)N(C)C)C.CC(=NC(=N)NC(=N)N(C)C)C.[Zn]

Physicochemical Properties	
Formula	C14H30N10Zn
Molecular weight	403.84 g/mol
Num. heavy atoms	25
Num. arom. heavy atoms	0
Fraction Csp3	0.57
Num. rotatable bonds	8
Num. H-bond acceptors	6
Num. H-bond donors	6
Molar Refractivity	104.65
TPSA	150.66 Å²

Lipophilicity	
Log $P_{o/w}$ (iLOGP)	-46.14
Log $P_{o/w}$ (XLOGP3)	-0.39
Log $P_{o/w}$ (WLOGP)	0.97
Log $P_{o/w}$ (MLOGP)	1.59
Log $P_{o/w}$ (SILICOS-IT)	-0.11
Consensus Log $P_{o/w}$	-8.82

Water Solubility	
Log *S* (ESOL)	-1.57
Solubility	1.09e+01 mg/ml ; 2.69e-02 mol/l
Class	Very soluble
Log *S* (Ali)	-2.31
Solubility	1.98e+00 mg/ml ; 4.89e-03 mol/l
Class	Soluble
Log *S* (SILICOS-IT)	-1.09
Solubility	3.27e+01 mg/ml ; 8.11e-02 mol/l
Class	Soluble

Pharmacokinetics	
GI absorption	Low
BBB permeant	No
P-gp substrate	Yes
CYP1A2 inhibitor	No
CYP2C19 inhibitor	No
CYP2C9 inhibitor	No
CYP2D6 inhibitor	No
CYP3A4 inhibitor	No
Log K_p (skin permeation)	-9.04 cm/s

Druglikeness	
Lipinski	Yes; 1 violation: NHorOH>5
Ghose	Yes
Veber	No; 1 violation: TPSA>140
Egan	No; 1 violation: TPSA>131.6
Muegge	No; 2 violations: TPSA>150, H-don>5
Bioavailability Score	0.55

Medicinal Chemistry	
PAINS	0 alert
Brenk	3 alerts: heavy_metal, imine_1, imine_2
Leadlikeness	No; 2 violations: MW>350, Rotors>7
Synthetic accessibility	4.19

MZN-6

Molecule 6

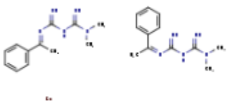

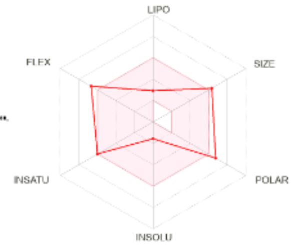

SMILES N=C(/N=C(/c1ccccc1)\C)NC(=N)N(C)C.N=C(/N=C(\c1ccccc1)/C)NC(=N)N(C)C.[Zn]

Physicochemical Properties	
Formula	C24H34N10Zn
Molecular weight	527.97 g/mol
Num. heavy atoms	35
Num. arom. heavy atoms	12
Fraction Csp3	0.25
Num. rotatable bonds	10
Num. H-bond acceptors	6
Num. H-bond donors	6
Molar Refractivity	144.01
TPSA	150.66 Å²

Lipophilicity	
Log $P_{o/w}$ (iLOGP)	-46.14
Log $P_{o/w}$ (XLOGP3)	-0.39
Log $P_{o/w}$ (WLOGP)	3.03
Log $P_{o/w}$ (MLOGP)	1.59
Log $P_{o/w}$ (SILICOS-IT)	-0.11
Consensus Log $P_{o/w}$	-8.82

Water Solubility	
Log *S* (ESOL)	-1.57
Solubility	1.09e+01 mg/ml ; 2.69e-02 mol/l
Class	Very soluble
Log *S* (Ali)	-2.31
Solubility	1.98e+00 mg/ml ; 4.89e-03 mol/l
Class	Soluble
Log *S* (SILICOS-IT)	-1.09
Solubility	3.27e+01 mg/ml ; 8.11e-02 mol/l
Class	Soluble

Pharmacokinetics	
GI absorption	Low
BBB permeant	No
P-gp substrate	Yes
CYP1A2 inhibitor	No
CYP2C19 inhibitor	No
CYP2C9 inhibitor	No
CYP2D6 inhibitor	No
CYP3A4 inhibitor	No
Log K_p (skin permeation)	-9.04 cm/s

Druglikeness	
Lipinski	Yes; 1 violation: NHorOH>5
Ghose	Yes
Veber	No; 1 violation: TPSA>140
Egan	No; 1 violation: TPSA>131.6
Muegge	No; 2 violations: TPSA>150, H-don>5
Bioavailability Score	0.55

Medicinal Chemistry	
PAINS	0 alert
Brenk	3 alerts: heavy_metal, imine_1, imine_2
Leadlikeness	No; 2 violations: MW>350, Rotors>7
Synthetic accessibility	4.19

MZN-7

Molecule 7

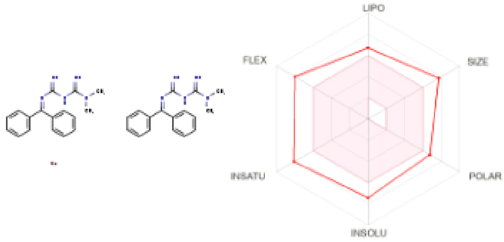

SMILES N=C(N=C(c1ccccc1)c1ccccc1)NC(=N)N(C)C.N=C(N=C(c1ccccc1)c1ccccc1)NC(=N)N(C)C.[Zn]

Physicochemical Properties	
Formula	C34H38N10Zn
Molecular weight	652.11 g/mol
Num. heavy atoms	45
Num. arom. heavy atoms	24
Fraction Csp3	0.12
Num. rotatable bonds	12
Num. H-bond acceptors	6
Num. H-bond donors	6
Molar Refractivity	183.37
TPSA	150.66 Å²
**Lipophilicity**	
Log $P_{o/w}$ (iLOGP)	-45.39
Log $P_{o/w}$ (XLOGP3)	6.25
Log $P_{o/w}$ (WLOGP)	5.09
Log $P_{o/w}$ (MLOGP)	4.99
Log $P_{o/w}$ (SILICOS-IT)	2.51
Consensus Log $P_{o/w}$	-5.31

Water Solubility	
Log *S* (ESOL)	-7.42
Solubility	2.46e-05 mg/ml ; 3.77e-08 mol/l
Class	Poorly soluble
Log *S* (Ali)	-9.20
Solubility	4.11e-07 mg/ml ; 6.30e-10 mol/l
Class	Poorly soluble
Log *S* (SILICOS-IT)	-5.35
Solubility	2.92e-03 mg/ml ; 4.48e-06 mol/l
Class	Moderately soluble
**Pharmacokinetics**	
GI absorption	Low
BBB permeant	No
P-gp substrate	Yes
CYP1A2 inhibitor	No
CYP2C19 inhibitor	No
CYP2C9 inhibitor	No
CYP2D6 inhibitor	No
CYP3A4 inhibitor	No
Log K_p (skin permeation)	-5.84 cm/s
**Druglikeness**	
Lipinski	No; 3 violations: MW>500, MLOGP>4.15, NHorOH>5
Ghose	No; 3 violations: MW>480, MR>130, #atoms>70
Veber	No; 2 violations: Rotors>10, TPSA>140
Egan	No; 1 violation: TPSA>131.6
Muegge	No; 4 violations: MW>600, XLOGP3>5, TPSA>150, H-don>5
Bioavailability Score	0.17
**Medicinal Chemistry**	
PAINS	0 alert
Brenk	3 alerts: heavy_metal, imine_1, imine_2
Leadlikeness	No; 3 violations: MW>350, Rotors>7, XLOGP3>3.5
Synthetic accessibility	4.73

MZN-8

Molecule 8

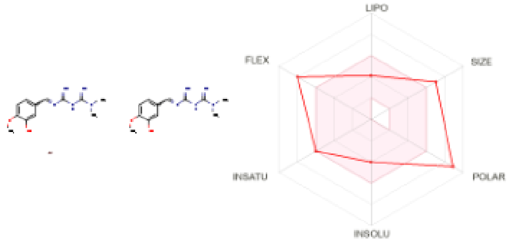

SMILES COc1ccc(cc1O)C=NC(=N)NC(=N)N(C)C.COc1ccc(cc1O)C=NC(=N)NC(=N)N(C)C.[Zn]

Physicochemical Properties	
Formula	C24H34N10O4Zn
Molecular weight	591.97 g/mol
Num. heavy atoms	39
Num. arom. heavy atoms	12
Fraction Csp3	0.25
Num. rotatable bonds	12
Num. H-bond acceptors	10
Num. H-bond donors	8
Molar Refractivity	151.43
TPSA	209.58 Å²
**Lipophilicity**	
Log $P_{o/w}$ (iLOGP)	-45.69
Log $P_{o/w}$ (XLOGP3)	1.72
Log $P_{o/w}$ (WLOGP)	1.68
Log $P_{o/w}$ (MLOGP)	1.51
Log $P_{o/w}$ (SILICOS-IT)	0.70
Consensus Log $P_{o/w}$	-8.01

Water Solubility	
Log *S* (ESOL)	-4.03
Solubility	5.53e-02 mg/ml ; 9.34e-05 mol/l
Class	Moderately soluble
Log *S* (Ali)	-5.74
Solubility	1.08e-03 mg/ml ; 1.83e-06 mol/l
Class	Moderately soluble
Log *S* (SILICOS-IT)	-2.40
Solubility	2.38e+00 mg/ml ; 4.03e-03 mol/l
Class	Soluble
**Pharmacokinetics**	
GI absorption	Low
BBB permeant	No
P-gp substrate	Yes
CYP1A2 inhibitor	No
CYP2C19 inhibitor	No
CYP2C9 inhibitor	No
CYP2D6 inhibitor	No
CYP3A4 inhibitor	No
Log K_p (skin permeation)	-8.69 cm/s
**Druglikeness**	
Lipinski	No; 3 violations: MW>500, NorO>10, NHorOH>5
Ghose	No; 3 violations: MW>480, MR>130, #atoms>70
Veber	No; 2 violations: Rotors>10, TPSA>140
Egan	No; 1 violation: TPSA>131.6
Muegge	No; 2 violations: TPSA>150, H-don>5
Bioavailability Score	0.17
**Medicinal Chemistry**	
PAINS	0 alert
Brenk	3 alerts: heavy_metal, imine_1, imine_2
Leadlikeness	No; 2 violations: MW>350, Rotors>7
Synthetic accessibility	4.21

MZN-9

Molecule 9

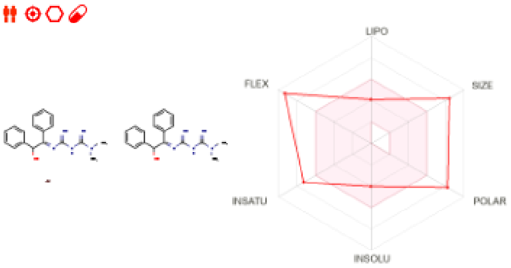

SMILES	N=C(/N=C(/C(c1ccccc1)O)\c1ccccc1)NC(=N)N(C)C.N=C(/N=C(/C(c1ccccc1)O)\c1ccccc1)NC(=N)N(C)C.[Zn]
**Physicochemical Properties**	
Formula	C36H42N10O2Zn
Molecular weight	712.16 g/mol
Num. heavy atoms	49
Num. arom. heavy atoms	24
Fraction Csp3	0.17
Num. rotatable bonds	14
Num. H-bond acceptors	8
Num. H-bond donors	8
Molar Refractivity	195.31
TPSA	191.12 Å²
**Lipophilicity**	
Log $P_{o/w}$ (iLOGP)	-45.69
Log $P_{o/w}$ (XLOGP3)	1.72
Log $P_{o/w}$ (WLOGP)	3.81
Log $P_{o/w}$ (MLOGP)	1.51
Log $P_{o/w}$ (SILICOS-IT)	0.70
Consensus Log $P_{o/w}$	-8.01
**Water Solubility**	
Log *S* (ESOL)	-4.03
Solubility	5.53e-02 mg/ml ; 9.34e-05 mol/l
Class	Moderately soluble
Log *S* (Ali)	-5.74
Solubility	1.08e-03 mg/ml ; 1.83e-06 mol/l
Class	Moderately soluble
Log *S* (SILICOS-IT)	-2.40
Solubility	2.38e+00 mg/ml ; 4.03e-03 mol/l
Class	Soluble
**Pharmacokinetics**	
GI absorption	Low
BBB permeant	No
P-gp substrate	Yes
CYP1A2 inhibitor	No
CYP2C19 inhibitor	No
CYP2C9 inhibitor	No
CYP2D6 inhibitor	No
CYP3A4 inhibitor	No
Log K_p (skin permeation)	-8.69 cm/s
**Druglikeness**	
Lipinski	No; 3 violations: MW>500, NorO>10, NHorOH>5
Ghose	No; 3 violations: MW>480, MR>130, #atoms>70
Veber	No; 2 violations: Rotors>10, TPSA>140
Egan	No; 1 violation: TPSA>131.6
Muegge	No; 2 violations: TPSA>150, H-don>5
Bioavailability Score	0.17
**Medicinal Chemistry**	
PAINS	0 alert
Brenk	3 alerts: heavy_metal, imine_1, imine_2
Leadlikeness	No; 2 violations: MW>350, Rotors>7
Synthetic accessibility	4.21

MCu-1

Molecule 1

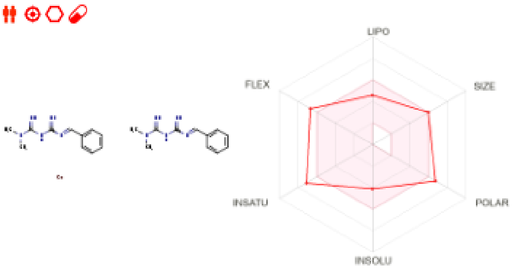

SMILES	CN(C(=N)NC(=N)N=Cc1ccccc1)C.CN(C(=N)NC(=N)N=Cc1ccccc1)C.[Cu]
**Physicochemical Properties**	
Formula	C22H30CuN10
Molecular weight	498.09 g/mol
Num. heavy atoms	33
Num. arom. heavy atoms	12
Fraction Csp3	0.18
Num. rotatable bonds	10
Num. H-bond acceptors	6
Num. H-bond donors	6
Molar Refractivity	134.40
TPSA	150.66 Å²
**Lipophilicity**	
Log $P_{o/w}$ (iLOGP)	-35.75
Log $P_{o/w}$ (XLOGP3)	2.49
Log $P_{o/w}$ (WLOGP)	2.25
Log $P_{o/w}$ (MLOGP)	3.03
Log $P_{o/w}$ (SILICOS-IT)	1.06
Consensus Log $P_{o/w}$	-5.38
**Water Solubility**	
Log *S* (ESOL)	-4.11
Solubility	3.90e-02 mg/ml ; 7.84e-05 mol/l
Class	Moderately soluble
Log *S* (Ali)	-5.30
Solubility	2.50e-03 mg/ml ; 5.02e-06 mol/l
Class	Moderately soluble
Log *S* (SILICOS-IT)	-2.85
Solubility	7.08e-01 mg/ml ; 1.42e-03 mol/l
Class	Soluble
**Pharmacokinetics**	
GI absorption	Low
BBB permeant	No
P-gp substrate	Yes
CYP1A2 inhibitor	No
CYP2C19 inhibitor	No
CYP2C9 inhibitor	No
CYP2D6 inhibitor	No
CYP3A4 inhibitor	No
Log K_p (skin permeation)	-7.57 cm/s
**Druglikeness**	
Lipinski	Yes; 1 violation: NHorOH>5
Ghose	No; 2 violations: MW>480, MR>130
Veber	No; 1 violation: TPSA>140
Egan	No; 1 violation: TPSA>131.6
Muegge	No; 2 violations: TPSA>150, H-don>5
Bioavailability Score	0.55
**Medicinal Chemistry**	
PAINS	0 alert
Brenk	2 alerts: imine_1, imine_2
Leadlikeness	No; 2 violations: MW>350, Rotors>7
Synthetic accessibility	3.95

MCu-2

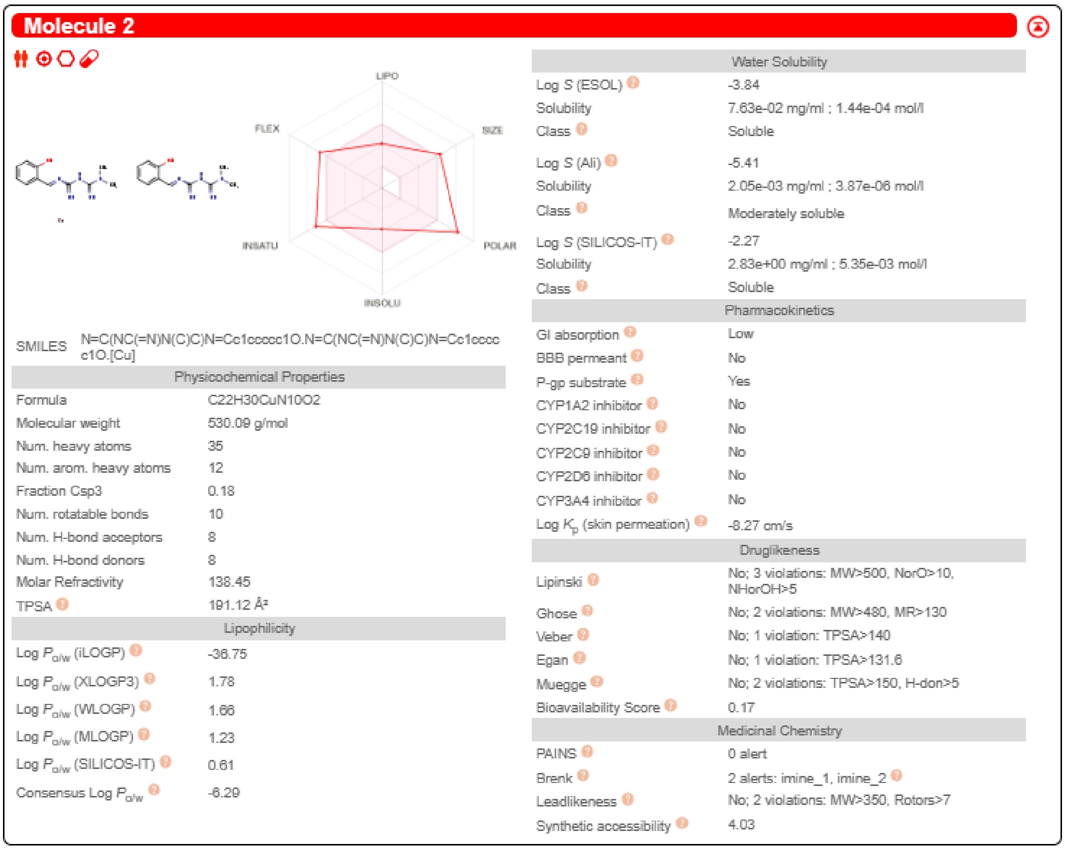

SMILES N=C(NC(=N)N(C)C)N=Cc1ccccc1O.N=C(NC(=N)N(C)C)N=Cc1cccc c1O.[Cu]

Physicochemical Properties	
Formula	C22H30CuN10O2
Molecular weight	530.09 g/mol
Num. heavy atoms	35
Num. arom. heavy atoms	12
Fraction Csp3	0.18
Num. rotatable bonds	10
Num. H-bond acceptors	8
Num. H-bond donors	8
Molar Refractivity	138.45
TPSA	191.12 Å²

Lipophilicity	
Log $P_{o/w}$ (iLOGP)	-36.75
Log $P_{o/w}$ (XLOGP3)	1.78
Log $P_{o/w}$ (WLOGP)	1.66
Log $P_{o/w}$ (MLOGP)	1.23
Log $P_{o/w}$ (SILICOS-IT)	0.61
Consensus Log $P_{o/w}$	-6.29

Water Solubility	
Log *S* (ESOL)	-3.84
Solubility	7.63e-02 mg/ml ; 1.44e-04 mol/l
Class	Soluble
Log *S* (Ali)	-5.41
Solubility	2.05e-03 mg/ml ; 3.87e-06 mol/l
Class	Moderately soluble
Log *S* (SILICOS-IT)	-2.27
Solubility	2.83e+00 mg/ml ; 5.35e-03 mol/l
Class	Soluble

Pharmacokinetics	
GI absorption	Low
BBB permeant	No
P-gp substrate	Yes
CYP1A2 inhibitor	No
CYP2C19 inhibitor	No
CYP2C9 inhibitor	No
CYP2D6 inhibitor	No
CYP3A4 inhibitor	No
Log K_p (skin permeation)	-8.27 cm/s

Druglikeness	
Lipinski	No; 3 violations: MW>500, NorO>10, NHorOH>5
Ghose	No; 2 violations: MW>480, MR>130
Veber	No; 1 violation: TPSA>140
Egan	No; 1 violation: TPSA>131.6
Muegge	No; 2 violations: TPSA>150, H-don>5
Bioavailability Score	0.17

Medicinal Chemistry	
PAINS	0 alert
Brenk	2 alerts: imine_1, imine_2
Leadlikeness	No; 2 violations: MW>350, Rotors>7
Synthetic accessibility	4.03

MCu-3

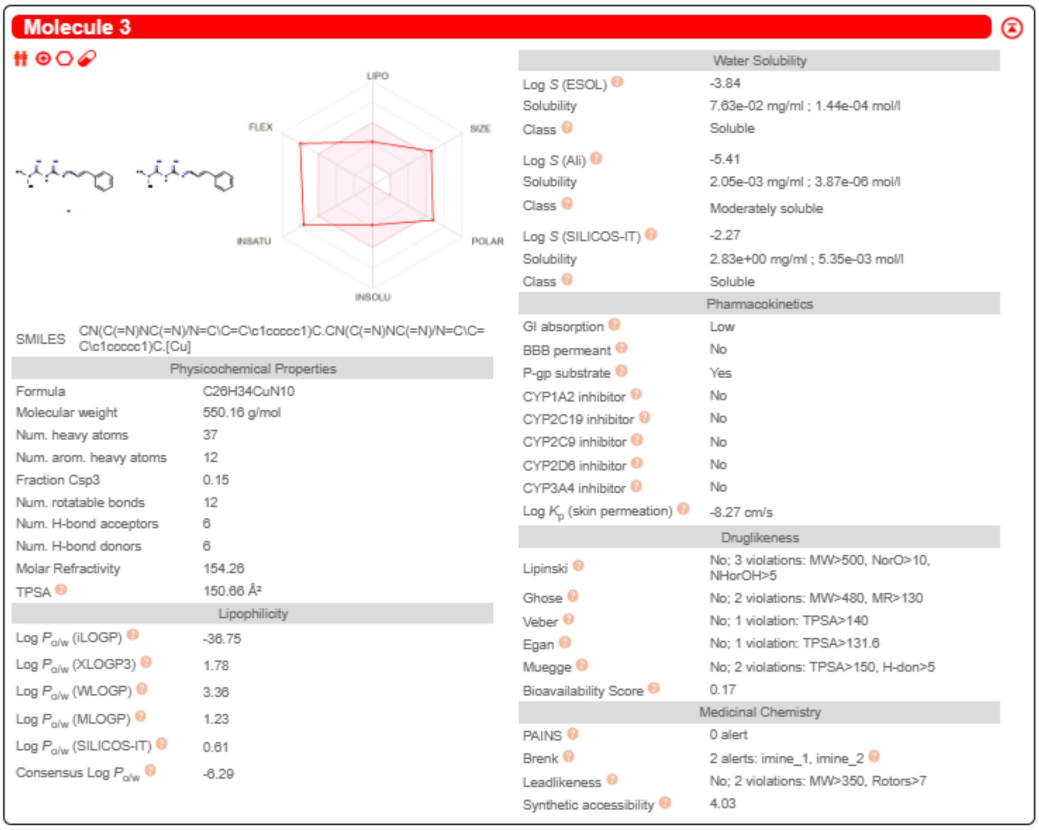

SMILES CN(C(=N)NC(=N)/N=C/C=C/c1ccccc1)C.CN(C(=N)NC(=N)/N=C/C=C/c1ccccc1)C.[Cu]

Physicochemical Properties	
Formula	C26H34CuN10
Molecular weight	550.16 g/mol
Num. heavy atoms	37
Num. arom. heavy atoms	12
Fraction Csp3	0.15
Num. rotatable bonds	12
Num. H-bond acceptors	6
Num. H-bond donors	6
Molar Refractivity	154.26
TPSA	150.66 Å²

Lipophilicity	
Log $P_{o/w}$ (iLOGP)	-36.75
Log $P_{o/w}$ (XLOGP3)	1.78
Log $P_{o/w}$ (WLOGP)	3.36
Log $P_{o/w}$ (MLOGP)	1.23
Log $P_{o/w}$ (SILICOS-IT)	0.61
Consensus Log $P_{o/w}$	-6.29

Water Solubility	
Log *S* (ESOL)	-3.84
Solubility	7.63e-02 mg/ml ; 1.44e-04 mol/l
Class	Soluble
Log *S* (Ali)	-5.41
Solubility	2.05e-03 mg/ml ; 3.87e-06 mol/l
Class	Moderately soluble
Log *S* (SILICOS-IT)	-2.27
Solubility	2.83e+00 mg/ml ; 5.35e-03 mol/l
Class	Soluble

Pharmacokinetics	
GI absorption	Low
BBB permeant	No
P-gp substrate	Yes
CYP1A2 inhibitor	No
CYP2C19 inhibitor	No
CYP2C9 inhibitor	No
CYP2D6 inhibitor	No
CYP3A4 inhibitor	No
Log K_p (skin permeation)	-8.27 cm/s

Druglikeness	
Lipinski	No; 3 violations: MW>500, NorO>10, NHorOH>5
Ghose	No; 2 violations: MW>480, MR>130
Veber	No; 1 violation: TPSA>140
Egan	No; 1 violation: TPSA>131.6
Muegge	No; 2 violations: TPSA>150, H-don>5
Bioavailability Score	0.17

Medicinal Chemistry	
PAINS	0 alert
Brenk	2 alerts: imine_1, imine_2
Leadlikeness	No; 2 violations: MW>350, Rotors>7
Synthetic accessibility	4.03

MCu-4

Molecule 4

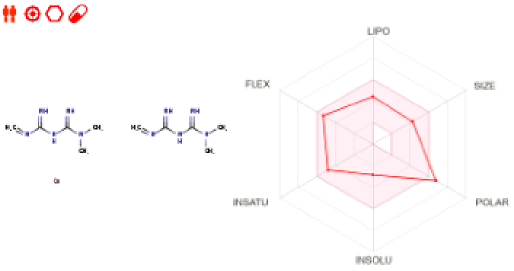

SMILES C=NC(=N)NC(=N)N(C)C.C=NC(=N)NC(=N)N(C)C.[Cu]

Physicochemical Properties	
Formula	C10H22CuN10
Molecular weight	345.89 g/mol
Num. heavy atoms	21
Num. arom. heavy atoms	0
Fraction Csp3	0.40
Num. rotatable bonds	8
Num. H-bond acceptors	6
Num. H-bond donors	6
Molar Refractivity	85.43
TPSA	150.66 Å²

Lipophilicity	
Log $P_{o/w}$ (iLOGP)	-38.41
Log $P_{o/w}$ (XLOGP3)	2.21
Log $P_{o/w}$ (WLOGP)	-0.59
Log $P_{o/w}$ (MLOGP)	0.48
Log $P_{o/w}$ (SILICOS-IT)	-0.52
Consensus Log $P_{o/w}$	-7.36

Water Solubility	
Log *S* (ESOL)	-2.85
Solubility	4.90e-01 mg/ml ; 1.42e-03 mol/l
Class	Soluble
Log *S* (Ali)	-5.01
Solubility	3.39e-03 mg/ml ; 9.81e-06 mol/l
Class	Moderately soluble
Log *S* (SILICOS-IT)	-0.65
Solubility	7.69e+01 mg/ml ; 2.22e-01 mol/l
Class	Soluble

Pharmacokinetics	
GI absorption	Low
BBB permeant	No
P-gp substrate	Yes
CYP1A2 inhibitor	No
CYP2C19 inhibitor	No
CYP2C9 inhibitor	No
CYP2D6 inhibitor	No
CYP3A4 inhibitor	No
Log K_p (skin permeation)	-6.84 cm/s

Druglikeness	
Lipinski	Yes; 1 violation: NHorOH>5
Ghose	No; 1 violation: WLOGP<-0.4
Veber	No; 1 violation: TPSA>140
Egan	No; 1 violation: TPSA>131.6
Muegge	No; 2 violations: TPSA>150, H-don>5
Bioavailability Score	0.55

Medicinal Chemistry	
PAINS	0 alert
Brenk	2 alerts: imine_1, imine_2
Leadlikeness	No; 1 violation: Rotors>7
Synthetic accessibility	4.11

MCu-5

Molecule 5

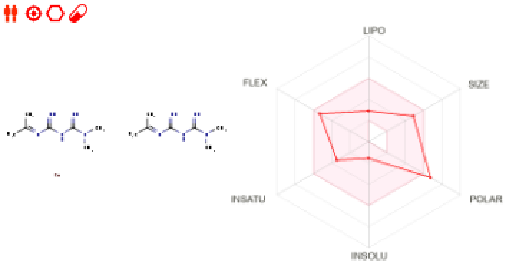

SMILES CC(=NC(=N)NC(=N)N(C)C)C.CC(=NC(=N)NC(=N)N(C)C)C.[Cu]

Physicochemical Properties	
Formula	C14H30CuN10
Molecular weight	402.00 g/mol
Num. heavy atoms	25
Num. arom. heavy atoms	0
Fraction Csp3	0.57
Num. rotatable bonds	8
Num. H-bond acceptors	6
Num. H-bond donors	6
Molar Refractivity	104.65
TPSA	150.66 Å²

Lipophilicity	
Log $P_{o/w}$ (iLOGP)	-36.80
Log $P_{o/w}$ (XLOGP3)	-0.39
Log $P_{o/w}$ (WLOGP)	0.97
Log $P_{o/w}$ (MLOGP)	1.59
Log $P_{o/w}$ (SILICOS-IT)	-0.11
Consensus Log $P_{o/w}$	-6.95

Water Solubility	
Log *S* (ESOL)	-1.56
Solubility	1.11e+01 mg/ml ; 2.76e-02 mol/l
Class	Very soluble
Log *S* (Ali)	-2.31
Solubility	1.97e+00 mg/ml ; 4.89e-03 mol/l
Class	Soluble
Log *S* (SILICOS-IT)	-1.09
Solubility	3.26e+01 mg/ml ; 8.11e-02 mol/l
Class	Soluble

Pharmacokinetics	
GI absorption	Low
BBB permeant	No
P-gp substrate	Yes
CYP1A2 inhibitor	No
CYP2C19 inhibitor	No
CYP2C9 inhibitor	No
CYP2D6 inhibitor	No
CYP3A4 inhibitor	No
Log K_p (skin permeation)	-9.03 cm/s

Druglikeness	
Lipinski	Yes; 1 violation: NHorOH>5
Ghose	Yes
Veber	No; 1 violation: TPSA>140
Egan	No; 1 violation: TPSA>131.6
Muegge	No; 2 violations: TPSA>150, H-don>5
Bioavailability Score	0.55

Medicinal Chemistry	
PAINS	0 alert
Brenk	2 alerts: imine_1, imine_2
Leadlikeness	No; 2 violations: MW>350, Rotors>7
Synthetic accessibility	4.17

MCu-6

Molecule 6

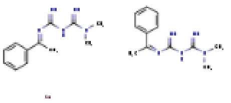

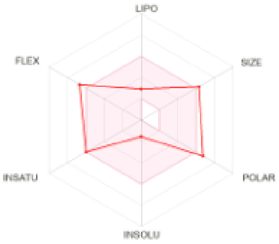

SMILES N=C(/N=C(/c1ccccc1)\C)NC(=N)N(C)C.N=C(/N=C(\c1ccccc1)/C)NC(=N)N(C)C.[Cu]

Physicochemical Properties	
Formula	C24H34CuN10
Molecular weight	526.14 g/mol
Num. heavy atoms	35
Num. arom. heavy atoms	12
Fraction Csp3	0.25
Num. rotatable bonds	10
Num. H-bond acceptors	6
Num. H-bond donors	6
Molar Refractivity	144.01
TPSA	150.66 Å²
**Lipophilicity**	
Log $P_{o/w}$ (iLOGP)	-36.80
Log $P_{o/w}$ (XLOGP3)	-0.39
Log $P_{o/w}$ (WLOGP)	3.03
Log $P_{o/w}$ (MLOGP)	1.59
Log $P_{o/w}$ (SILICOS-IT)	-0.11
Consensus Log $P_{o/w}$	-6.95

Water Solubility	
Log *S* (ESOL)	-1.56
Solubility	1.11e+01 mg/ml ; 2.76e-02 mol/l
Class	Very soluble
Log *S* (Ali)	-2.31
Solubility	1.97e+00 mg/ml ; 4.89e-03 mol/l
Class	Soluble
Log *S* (SILICOS-IT)	-1.09
Solubility	3.26e+01 mg/ml ; 8.11e-02 mol/l
Class	Soluble
**Pharmacokinetics**	
GI absorption	Low
BBB permeant	No
P-gp substrate	Yes
CYP1A2 inhibitor	No
CYP2C19 inhibitor	No
CYP2C9 inhibitor	No
CYP2D6 inhibitor	No
CYP3A4 inhibitor	No
Log K_p (skin permeation)	-9.03 cm/s
**Druglikeness**	
Lipinski	Yes; 1 violation: NHorOH>5
Ghose	Yes
Veber	No; 1 violation: TPSA>140
Egan	No; 1 violation: TPSA>131.6
Muegge	No; 2 violations: TPSA>150, H-don>5
Bioavailability Score	0.55
**Medicinal Chemistry**	
PAINS	0 alert
Brenk	2 alerts: imine_1, imine_2
Leadlikeness	No; 2 violations: MW>350, Rotors>7
Synthetic accessibility	4.17

MCu-7

Molecule 7

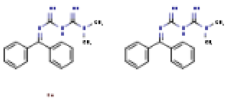

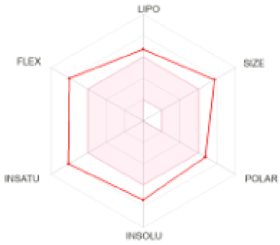

SMILES N=C(N=C(c1ccccc1)c1ccccc1)NC(=N)N(C)C.N=C(N=C(c1ccccc1)c1ccccc1)NC(=N)N(C)C.[Cu]

Physicochemical Properties	
Formula	C34H38CuN10
Molecular weight	650.28 g/mol
Num. heavy atoms	45
Num. arom. heavy atoms	24
Fraction Csp3	0.12
Num. rotatable bonds	12
Num. H-bond acceptors	6
Num. H-bond donors	6
Molar Refractivity	183.37
TPSA	150.66 Å²
**Lipophilicity**	
Log $P_{o/w}$ (iLOGP)	-36.37
Log $P_{o/w}$ (XLOGP3)	6.25
Log $P_{o/w}$ (WLOGP)	5.09
Log $P_{o/w}$ (MLOGP)	4.99
Log $P_{o/w}$ (SILICOS-IT)	2.51
Consensus Log $P_{o/w}$	-3.51

Water Solubility	
Log *S* (ESOL)	-7.41
Solubility	2.52e-05 mg/ml ; 3.87e-08 mol/l
Class	Poorly soluble
Log *S* (Ali)	-9.20
Solubility	4.10e-07 mg/ml ; 6.30e-10 mol/l
Class	Poorly soluble
Log *S* (SILICOS-IT)	-5.35
Solubility	2.91e-03 mg/ml ; 4.48e-06 mol/l
Class	Moderately soluble
**Pharmacokinetics**	
GI absorption	Low
BBB permeant	No
P-gp substrate	Yes
CYP1A2 inhibitor	No
CYP2C19 inhibitor	No
CYP2C9 inhibitor	No
CYP2D6 inhibitor	No
CYP3A4 inhibitor	No
Log K_p (skin permeation)	-5.83 cm/s
**Druglikeness**	
Lipinski	No; 3 violations: MW>500, MLOGP>4.15, NHorOH>5
Ghose	No; 3 violations: MW>480, MR>130, #atoms>70
Veber	No; 2 violations: Rotors>10, TPSA>140
Egan	No; 1 violation: TPSA>131.6
Muegge	No; 4 violations: MW>600, XLOGP3>5, TPSA>150, H-don>5
Bioavailability Score	0.17
**Medicinal Chemistry**	
PAINS	0 alert
Brenk	2 alerts: imine_1, imine_2
Leadlikeness	No; 3 violations: MW>350, Rotors>7, XLOGP3>3.5
Synthetic accessibility	4.72

MCu-8

Molecule 8

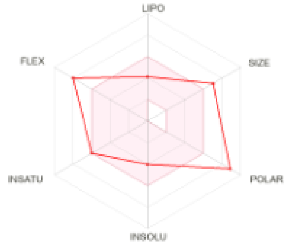

SMILES COc1ccc(cc1O)C=NC(=N)NC(=N)N(C)C.COc1ccc(cc1O)C=NC(=N)NC(=N)N(C)C.[Cu]

Physicochemical Properties	
Formula	C24H34CuN10O4
Molecular weight	590.14 g/mol
Num. heavy atoms	39
Num. arom. heavy atoms	12
Fraction Csp3	0.25
Num. rotatable bonds	12
Num. H-bond acceptors	10
Num. H-bond donors	8
Molar Refractivity	151.43
TPSA	209.58 Å²

Lipophilicity	
Log $P_{o/w}$ (iLOGP)	-36.71
Log $P_{o/w}$ (XLOGP3)	1.72
Log $P_{o/w}$ (WLOGP)	1.68
Log $P_{o/w}$ (MLOGP)	1.51
Log $P_{o/w}$ (SILICOS-IT)	0.70
Consensus Log $P_{o/w}$	-6.22

Water Solubility	
Log *S* (ESOL)	-4.02
Solubility	5.66e-02 mg/ml ; 9.59e-05 mol/l
Class	Moderately soluble
Log *S* (Ali)	-5.74
Solubility	1.08e-03 mg/ml ; 1.83e-06 mol/l
Class	Moderately soluble
Log *S* (SILICOS-IT)	-2.40
Solubility	2.38e+00 mg/ml ; 4.03e-03 mol/l
Class	Soluble

Pharmacokinetics	
GI absorption	Low
BBB permeant	No
P-gp substrate	Yes
CYP1A2 inhibitor	No
CYP2C19 inhibitor	No
CYP2C9 inhibitor	No
CYP2D6 inhibitor	No
CYP3A4 inhibitor	No
Log K_p (skin permeation)	-8.68 cm/s

Druglikeness	
Lipinski	No; 3 violations: MW>500, NorO>10, NHorOH>5
Ghose	No; 3 violations: MW>480, MR>130, #atoms>70
Veber	No; 2 violations: Rotors>10, TPSA>140
Egan	No; 1 violation: TPSA>131.6
Muegge	No; 2 violations: TPSA>150, H-don>5
Bioavailability Score	0.17

Medicinal Chemistry	
PAINS	0 alert
Brenk	2 alerts: imine_1, imine_2
Leadlikeness	No; 2 violations: MW>350, Rotors>7
Synthetic accessibility	4.21

MCu-9

Molecule 9

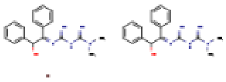

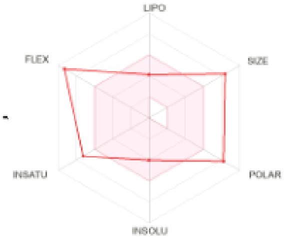

SMILES N=C(/N=C(/C(c1ccccc1)O)\c1ccccc1)NC(=N)N(C)C.N=C(/N=C(/C(c1ccccc1)O)\c1ccccc1)NC(=N)N(C)C.[Cu]

Physicochemical Properties	
Formula	C36H42CuN10O2
Molecular weight	710.33 g/mol
Num. heavy atoms	49
Num. arom. heavy atoms	24
Fraction Csp3	0.17
Num. rotatable bonds	14
Num. H-bond acceptors	8
Num. H-bond donors	8
Molar Refractivity	195.31
TPSA	191.12 Å²

Lipophilicity	
Log $P_{o/w}$ (iLOGP)	-36.71
Log $P_{o/w}$ (XLOGP3)	1.72
Log $P_{o/w}$ (WLOGP)	3.81
Log $P_{o/w}$ (MLOGP)	1.51
Log $P_{o/w}$ (SILICOS-IT)	0.70
Consensus Log $P_{o/w}$	-6.22

Water Solubility	
Log *S* (ESOL)	-4.02
Solubility	5.66e-02 mg/ml ; 9.59e-05 mol/l
Class	Moderately soluble
Log *S* (Ali)	-5.74
Solubility	1.08e-03 mg/ml ; 1.83e-06 mol/l
Class	Moderately soluble
Log *S* (SILICOS-IT)	-2.40
Solubility	2.38e+00 mg/ml ; 4.03e-03 mol/l
Class	Soluble

Pharmacokinetics	
GI absorption	Low
BBB permeant	No
P-gp substrate	Yes
CYP1A2 inhibitor	No
CYP2C19 inhibitor	No
CYP2C9 inhibitor	No
CYP2D6 inhibitor	No
CYP3A4 inhibitor	No
Log K_p (skin permeation)	-8.68 cm/s

Druglikeness	
Lipinski	No; 3 violations: MW>500, NorO>10, NHorOH>5
Ghose	No; 3 violations: MW>480, MR>130, #atoms>70
Veber	No; 2 violations: Rotors>10, TPSA>140
Egan	No; 1 violation: TPSA>131.6
Muegge	No; 2 violations: TPSA>150, H-don>5
Bioavailability Score	0.17

Medicinal Chemistry	
PAINS	0 alert
Brenk	2 alerts: imine_1, imine_2
Leadlikeness	No; 2 violations: MW>350, Rotors>7
Synthetic accessibility	4.21

Table S2. 2D structures of all Metformin derivatives with respect to amylase enzyme.

Derivative code	2D Pose
MET-1	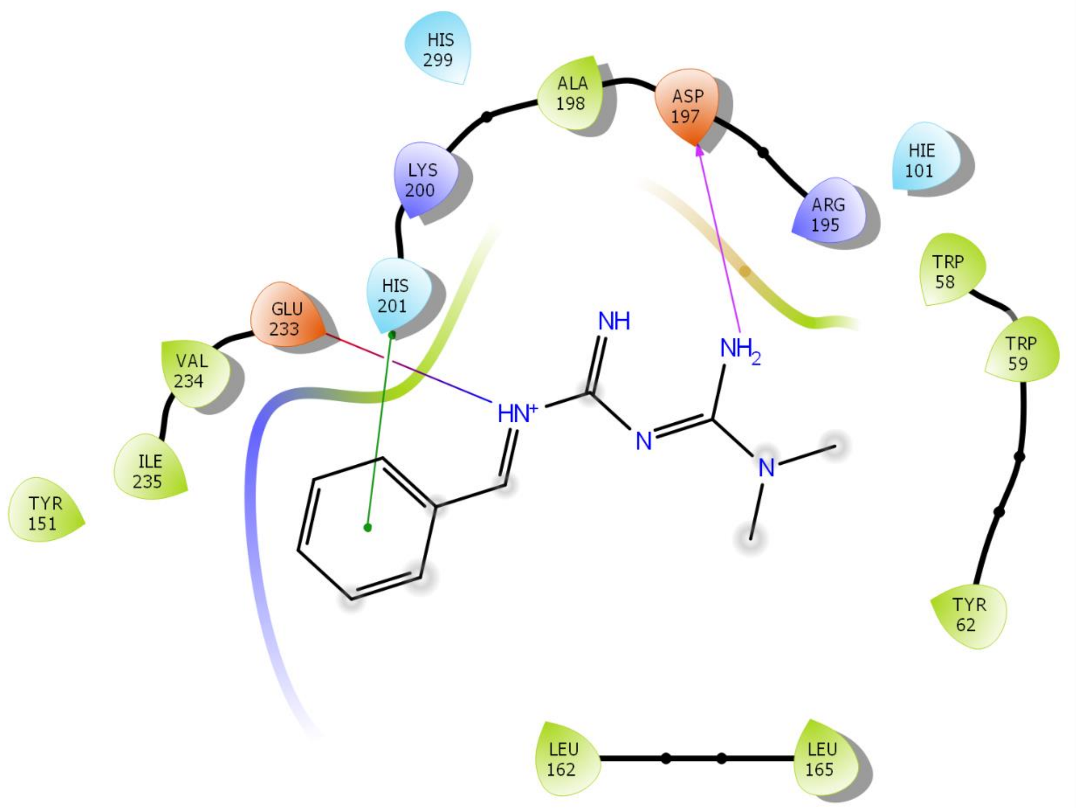
MET-2	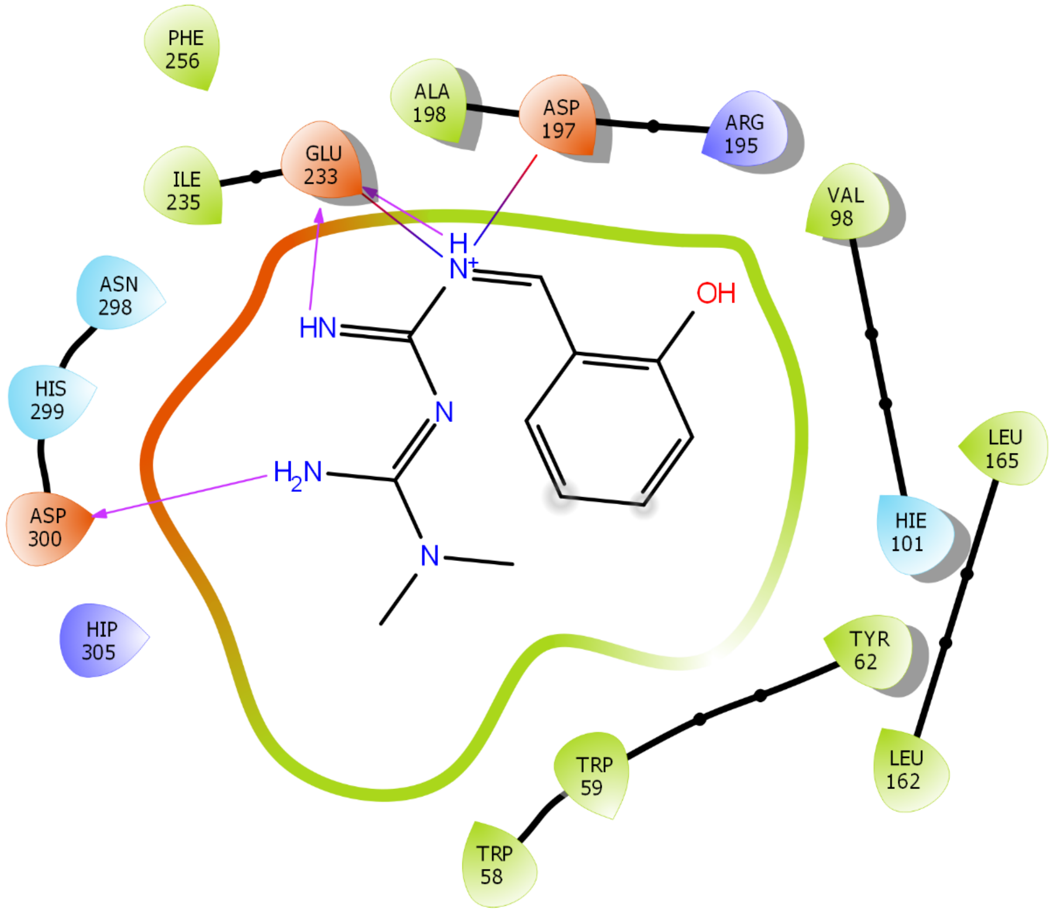

MET-3	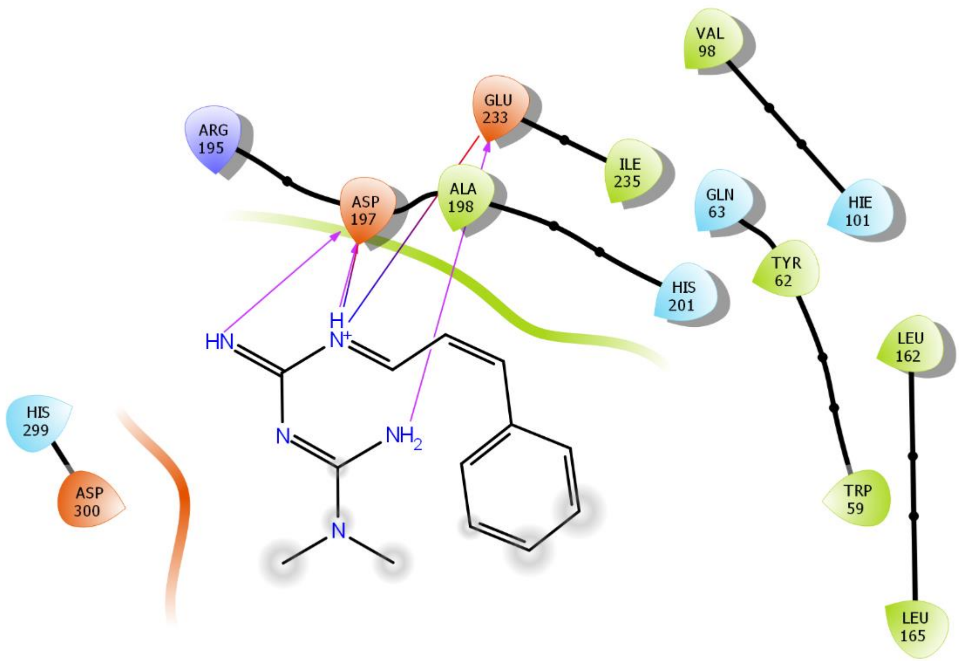
MET-4	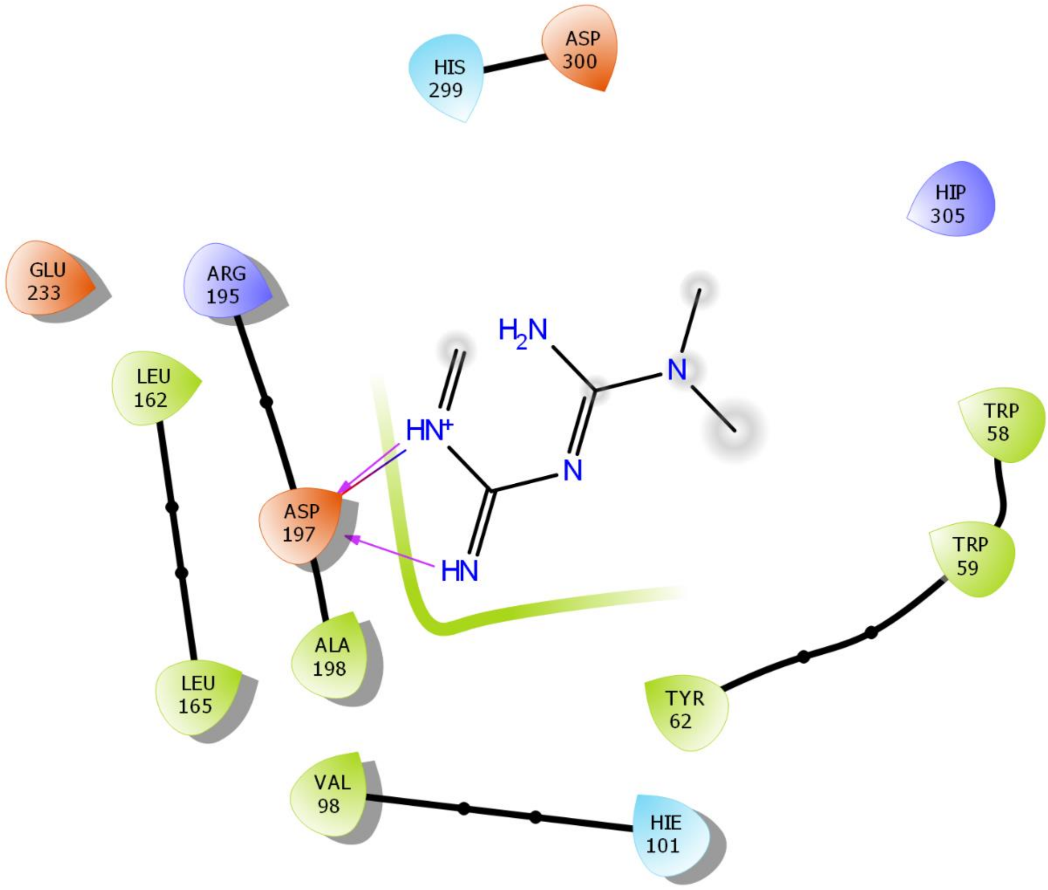

VAL 98
GLU 233
ARG 195
ILE 235
ASP 197
ALA 198
GLN 63
HIE 101
TYR 62
HIS 201
LEU 162
HIS 299
ASP 300
TRP 59
LEU 165
HN
H
N+
N
NH2
N

HIS 299
ASP 300
HIP 305
GLU 233
ARG 195
LEU 162
H2N
N
HN+
TRP 58
N
ASP 197
HN
TRP 59
ALA 198
LEU 165
TYR 62
VAL 98
HIE 101

MET-5	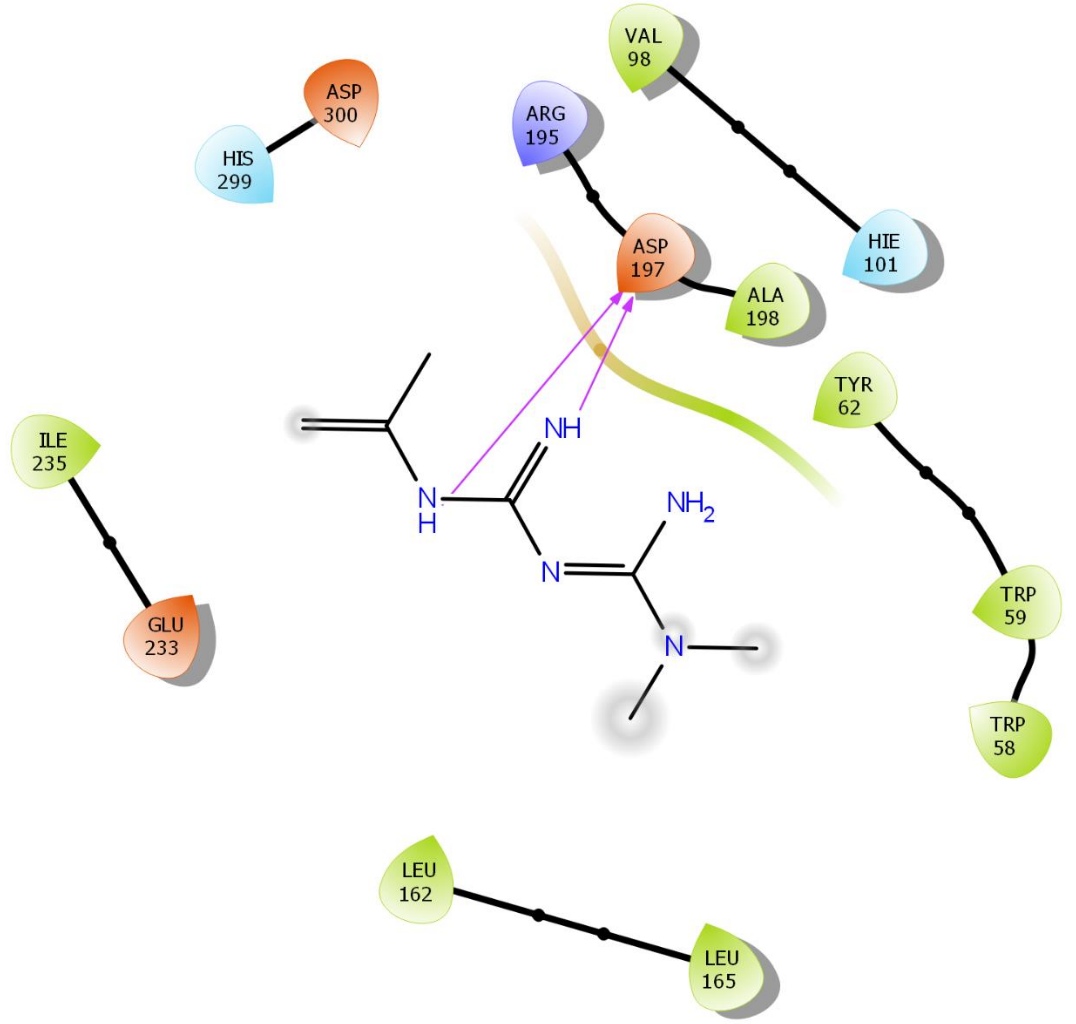ASP 300<br>HIS 299<br>VAL 98<br>ARG 195<br>ASP 197<br>HIE 101<br>ALA 198<br>TYR 62<br>ILE 235<br>NH<br>N H<br>NH_2<br>N<br>N<br>TRP 59<br>GLU 233<br>TRP 58<br>LEU 162<br>LEU 165

MET-6	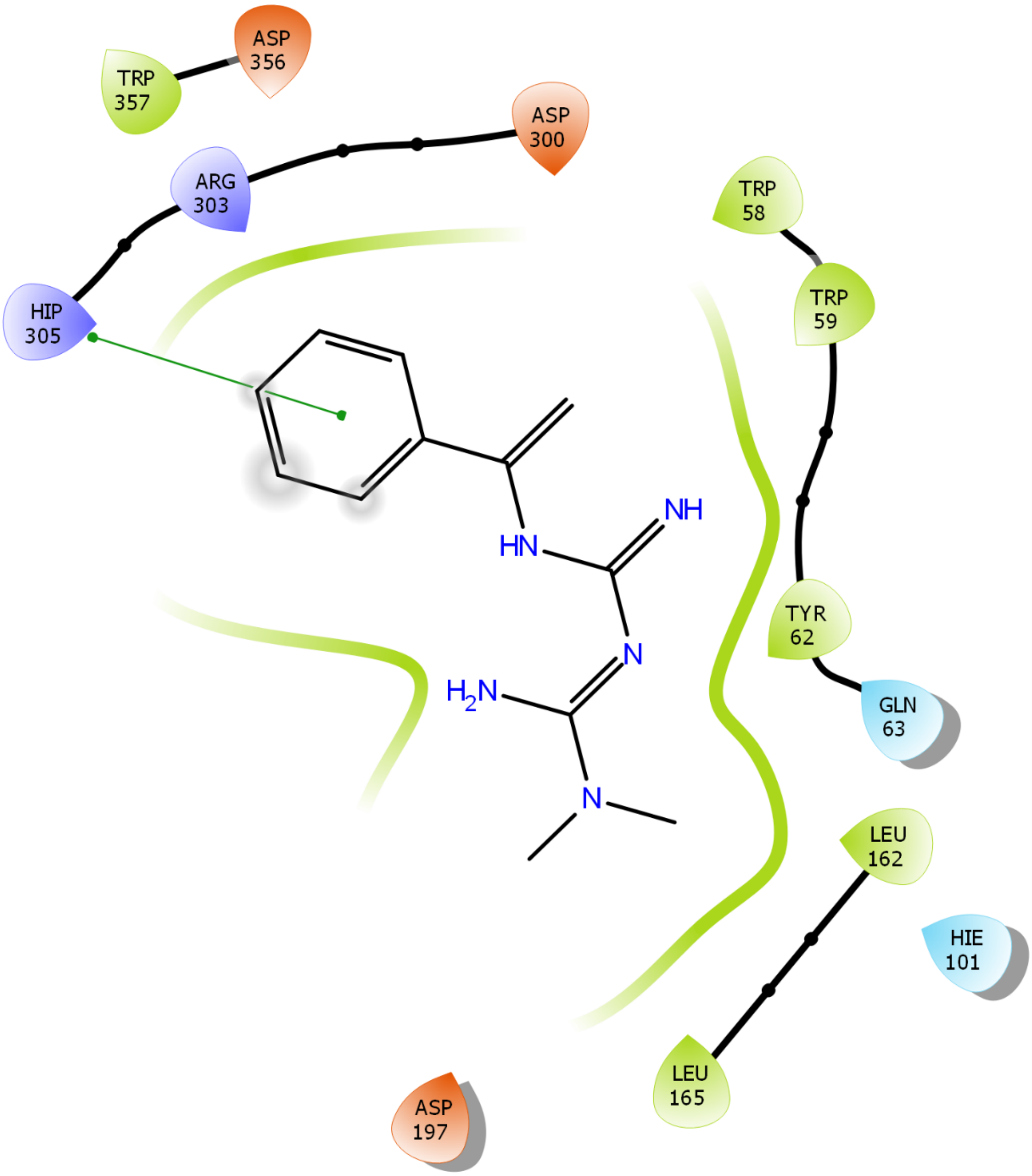

ASP
356
TRP
357
ASP
300
ARG
303
TRP
58
HIP
305
TRP
59
NH
HN
TYR
62
N
H_2N
GLN
63
N
LEU
162
HIE
101
LEU
165
ASP
197

MET-7	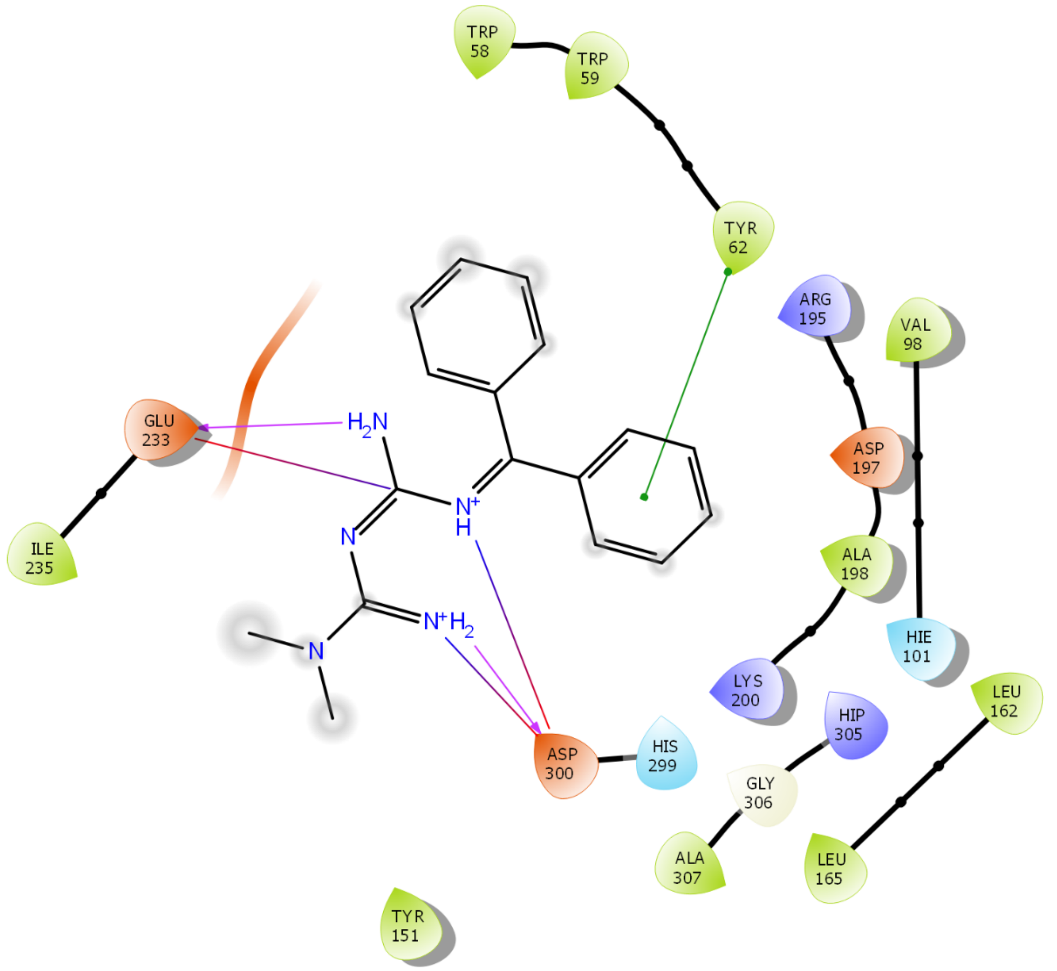TRP 58<br>TRP 59<br>TYR 62<br>ARG 195<br>VAL 98<br>ASP 197<br>ALA 198<br>HIE 101<br>LYS 200<br>LEU 162<br>HIP 305<br>GLY 306<br>ALA 307<br>LEU 165<br>ASP 300<br>HIS 299<br>GLU 233<br>ILE 235<br>TYR 151<br>H2N<br>N+ H<br>N<br>N+H2<br>N

MET-8	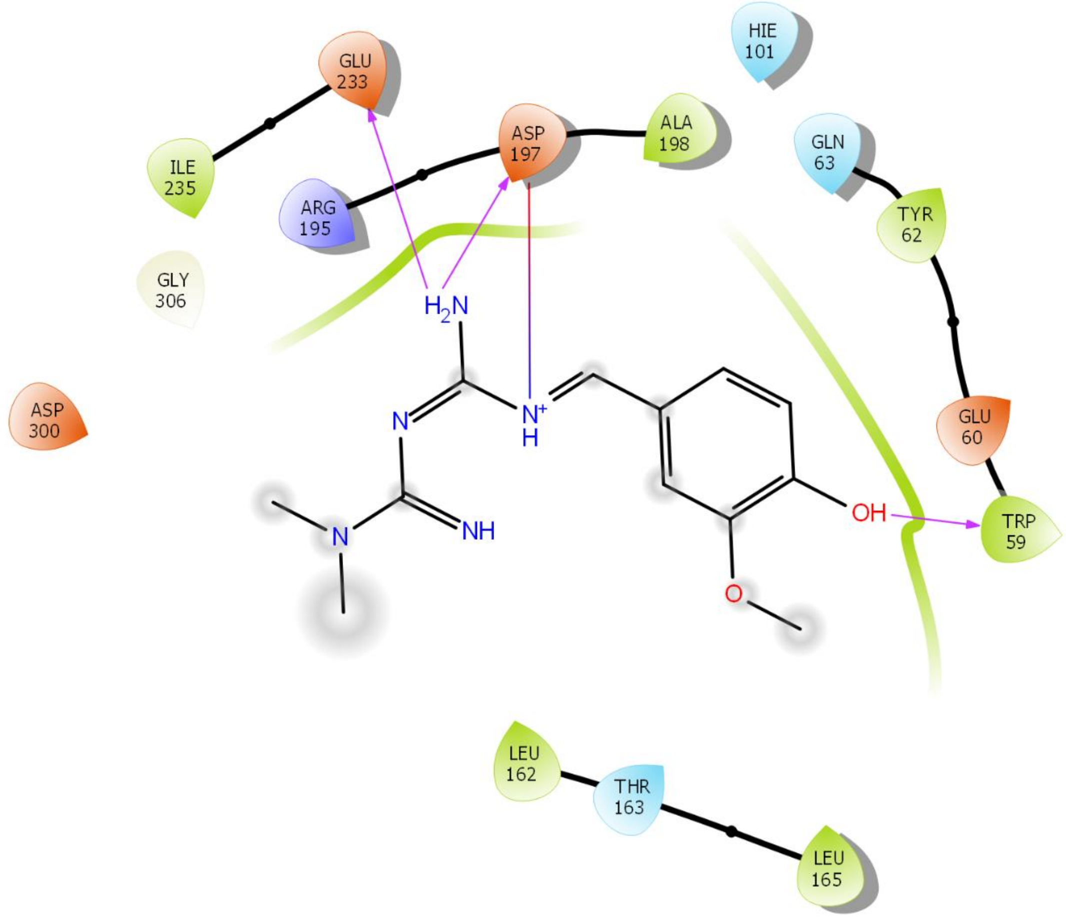

GLU 233
ILE 235
ASP 197
ALA 198
HIE 101
GLN 63
ARG 195
TYR 62
GLY 306
ASP 300
GLU 60
TRP 59
LEU 162
THR 163
LEU 165

MET-9	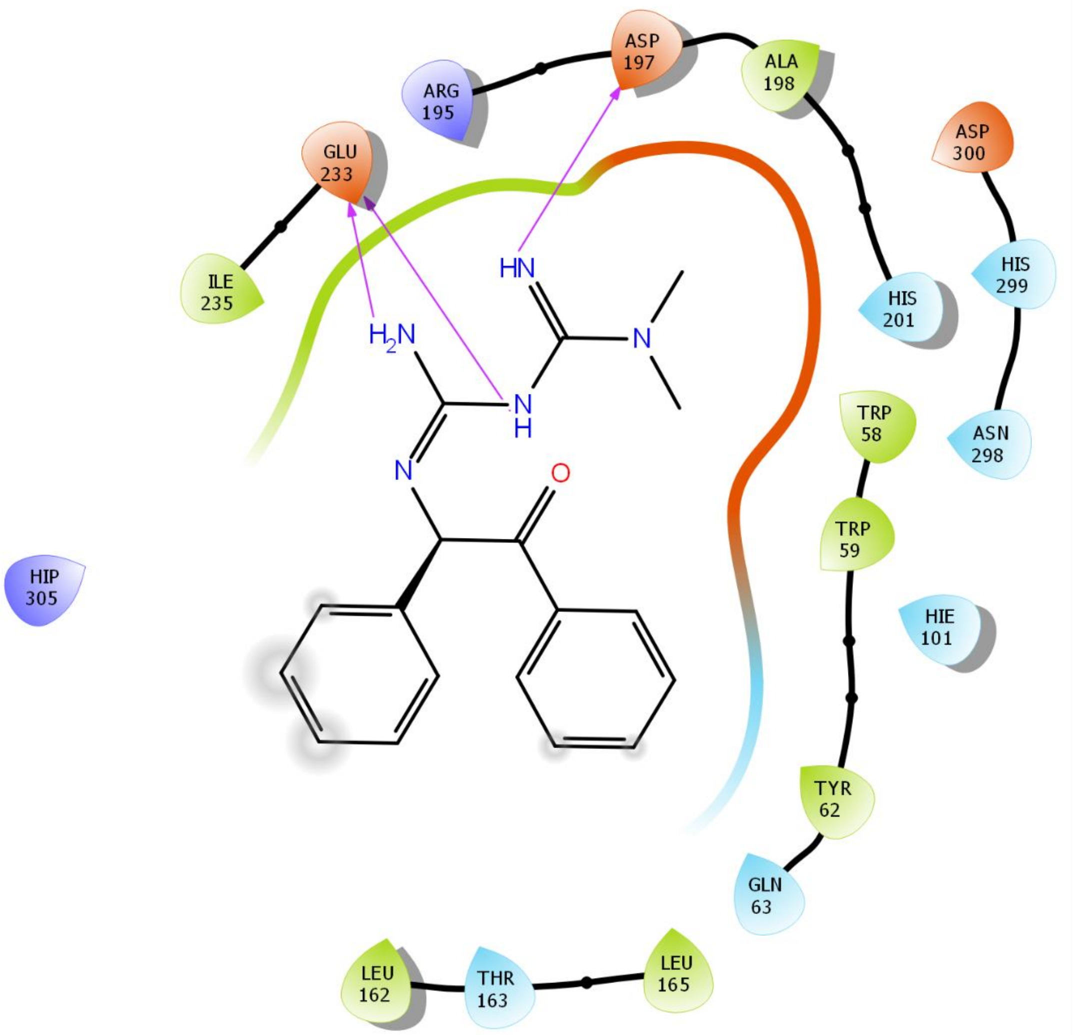

ARG 195
ASP 197
ALA 198
GLU 233
ASP 300
ILE 235
HIS 201
HIS 299
TRP 58
ASN 298
TRP 59
HIP 305
HIE 101
TYR 62
GLN 63
LEU 162
THR 163
LEU 165
HN
H_2N
N
NH
N
O

MET	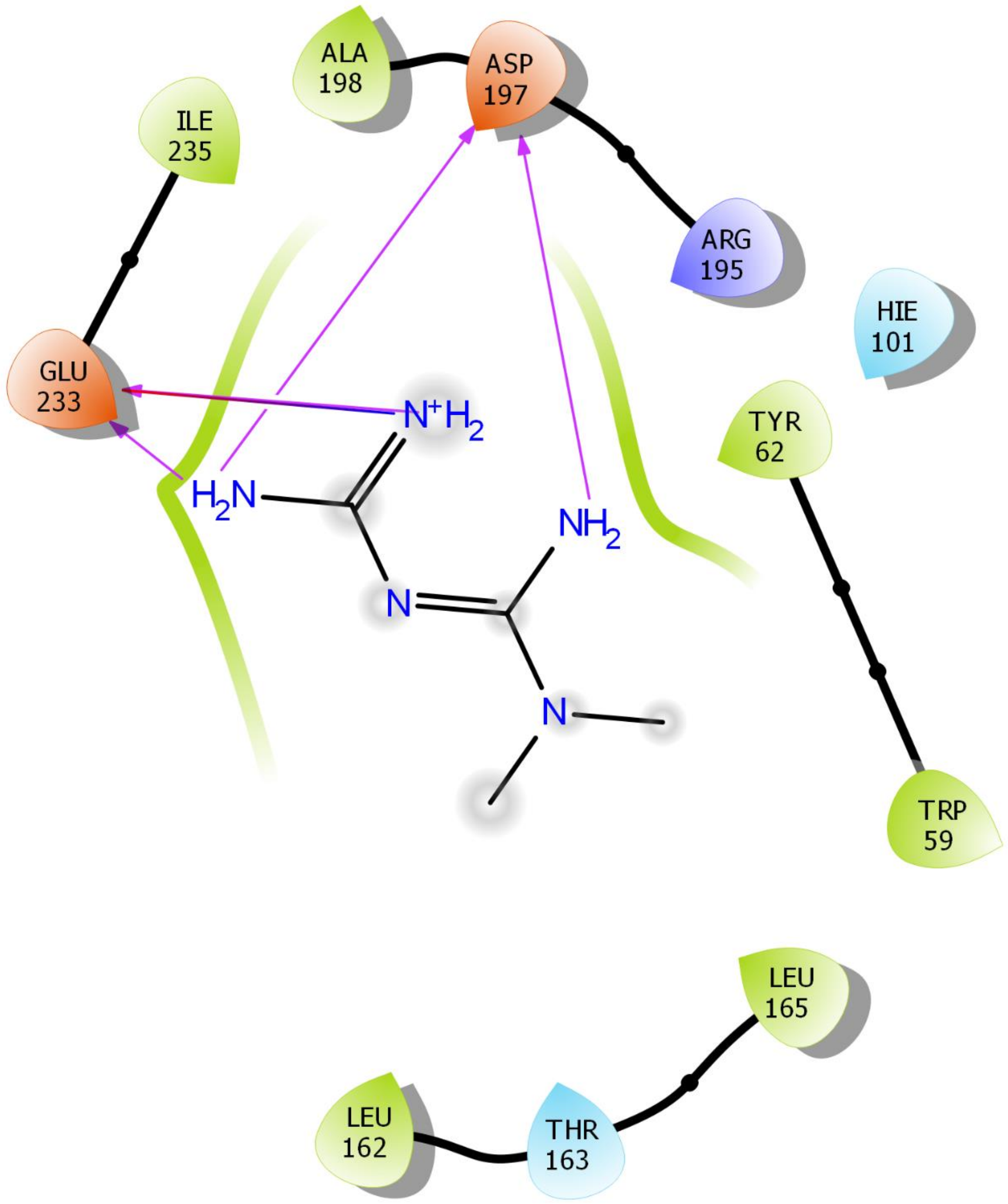

ALA 198
ASP 197
ILE 235
ARG 195
HIE 101
GLU 233
TYR 62
TRP 59
LEU 165
LEU 162
THR 163

MZN-1	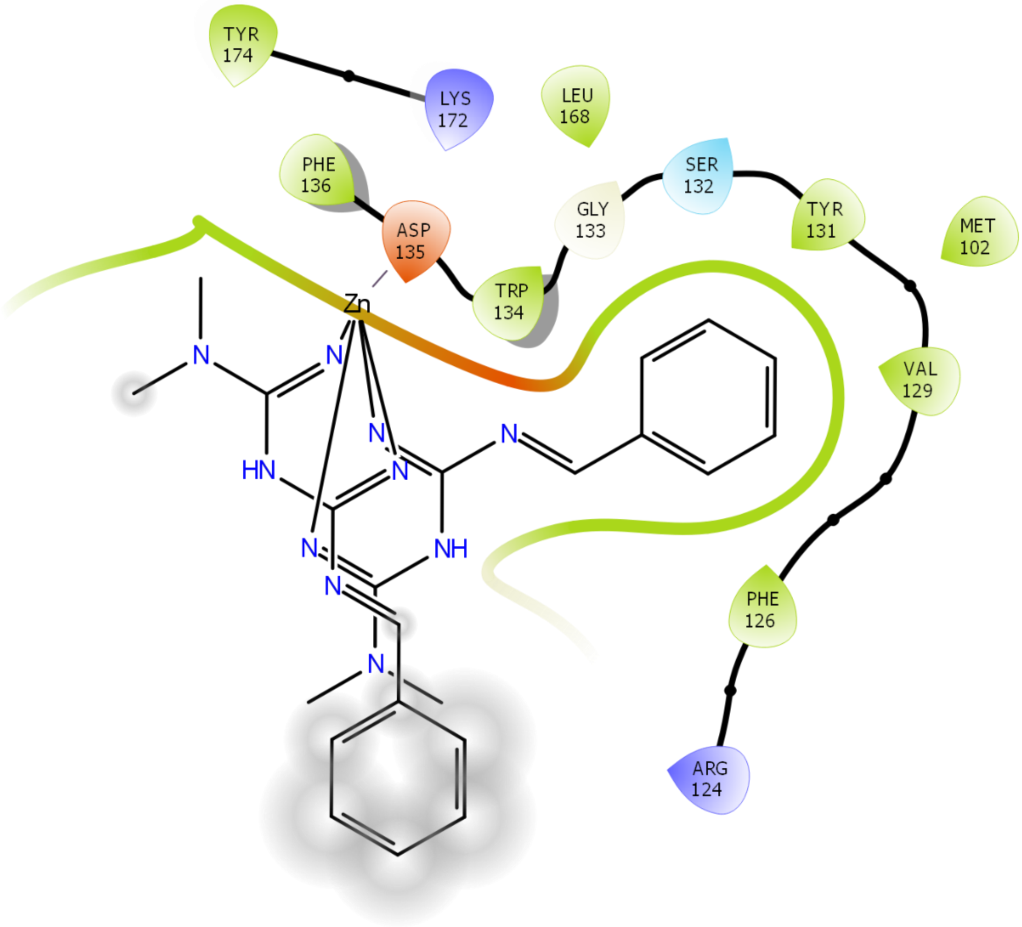

TYR 174
LYS 172
LEU 168
PHE 136
ASP 135
GLY 133
SER 132
TYR 131
MET 102
TRP 134
VAL 129
PHE 126
ARG 124
Zn
N
HN
NH

MZN-2

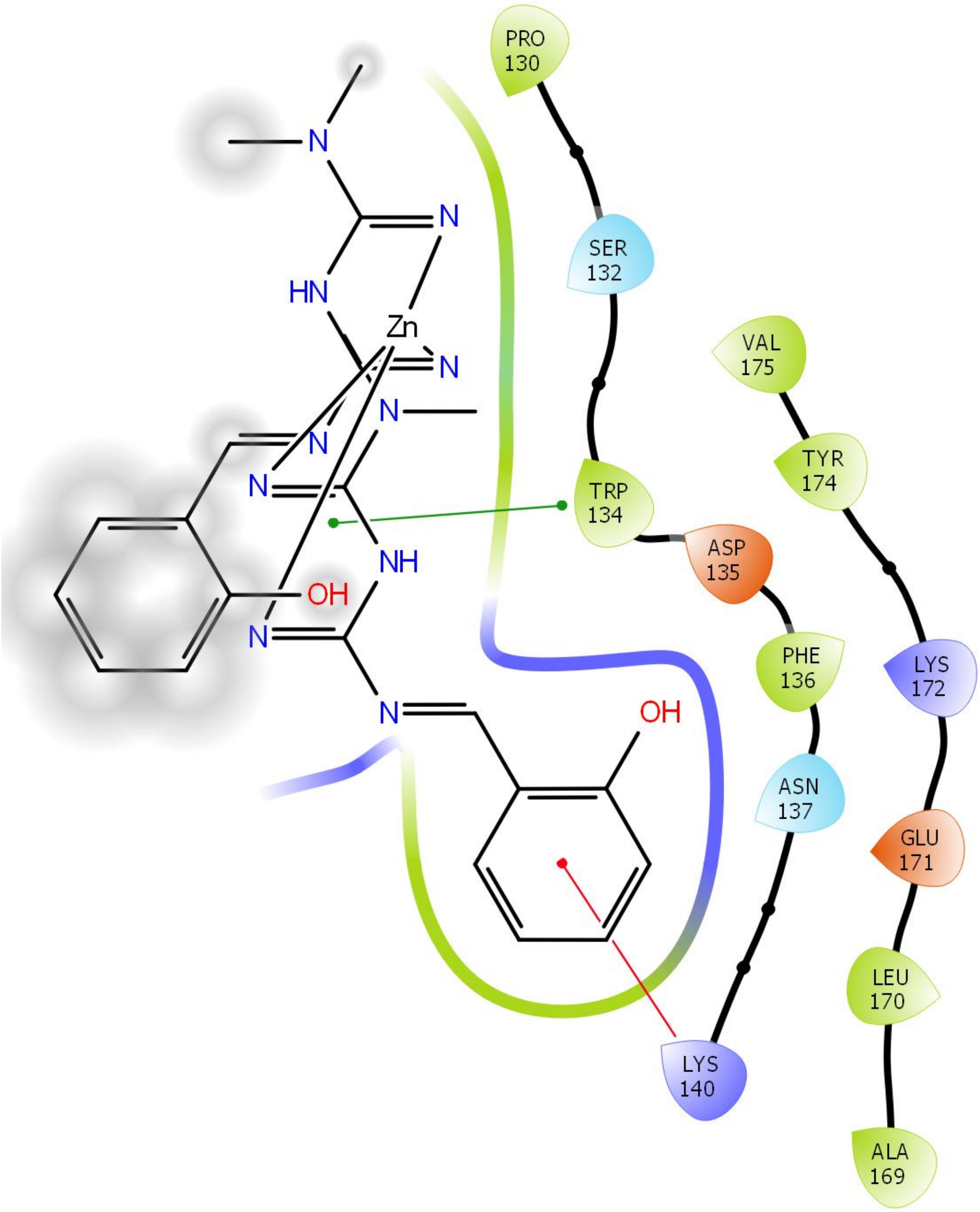

N
N
HN
Zn
N
N
N
N
NH
OH
N
N
OH
PRO 130
SER 132
VAL 175
TYR 174
TRP 134
ASP 135
PHE 136
LYS 172
ASN 137
GLU 171
LEU 170
LYS 140
ALA 169

MZN-3	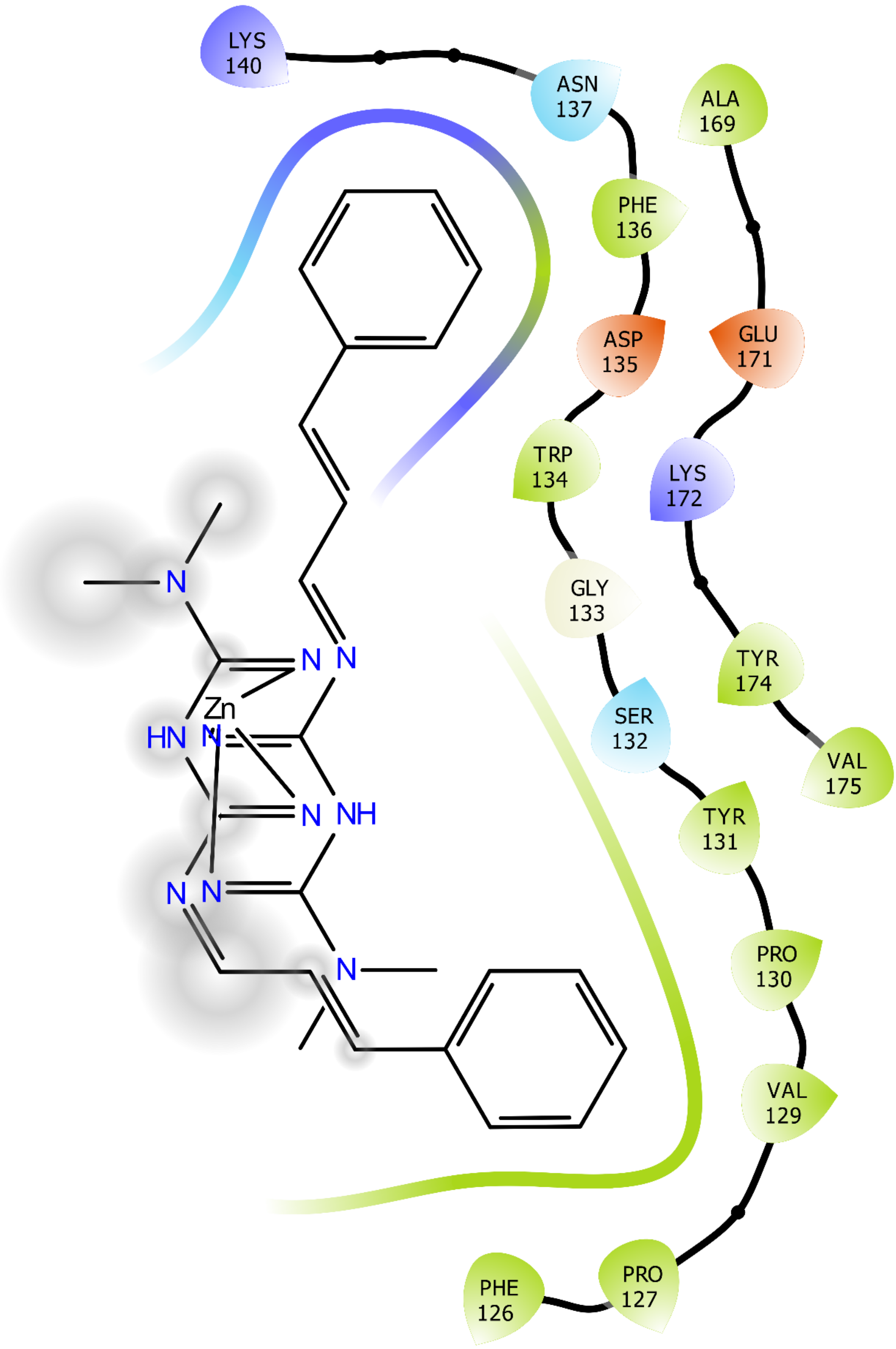

LYS 140
ASN 137
ALA 169
PHE 136
ASP 135
GLU 171
TRP 134
LYS 172
GLY 133
TYR 174
SER 132
VAL 175
TYR 131
PRO 130
VAL 129
PRO 127
PHE 126
Zn
N
HN
NH

MZN-4	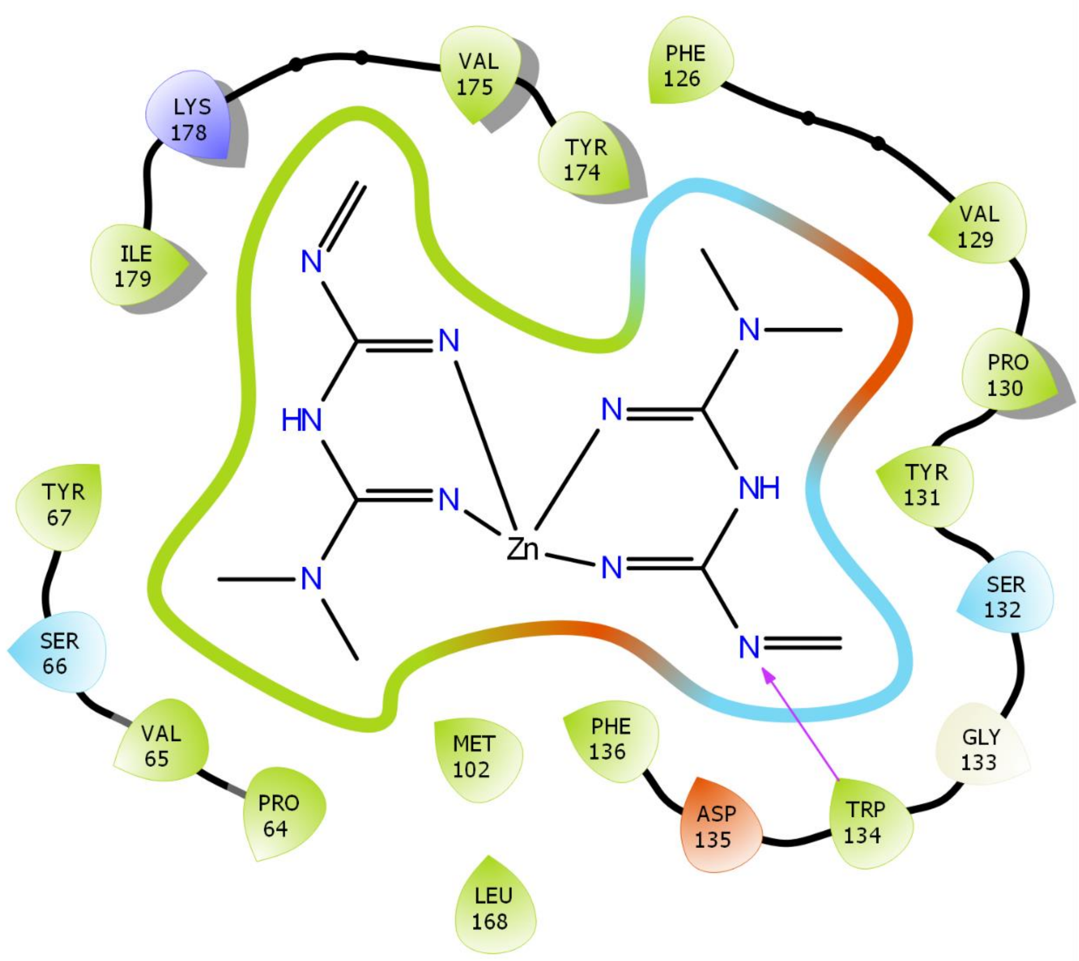 LYS 178<br>VAL 175<br>PHE 126<br>TYR 174<br>ILE 179<br>VAL 129<br>PRO 130<br>TYR 131<br>TYR 67<br>SER 132<br>SER 66<br>VAL 65<br>GLY 133<br>MET 102<br>PHE 136<br>PRO 64<br>ASP 135<br>TRP 134<br>LEU 168<br>N<br>HN<br>Zn<br>NH

MZN-5

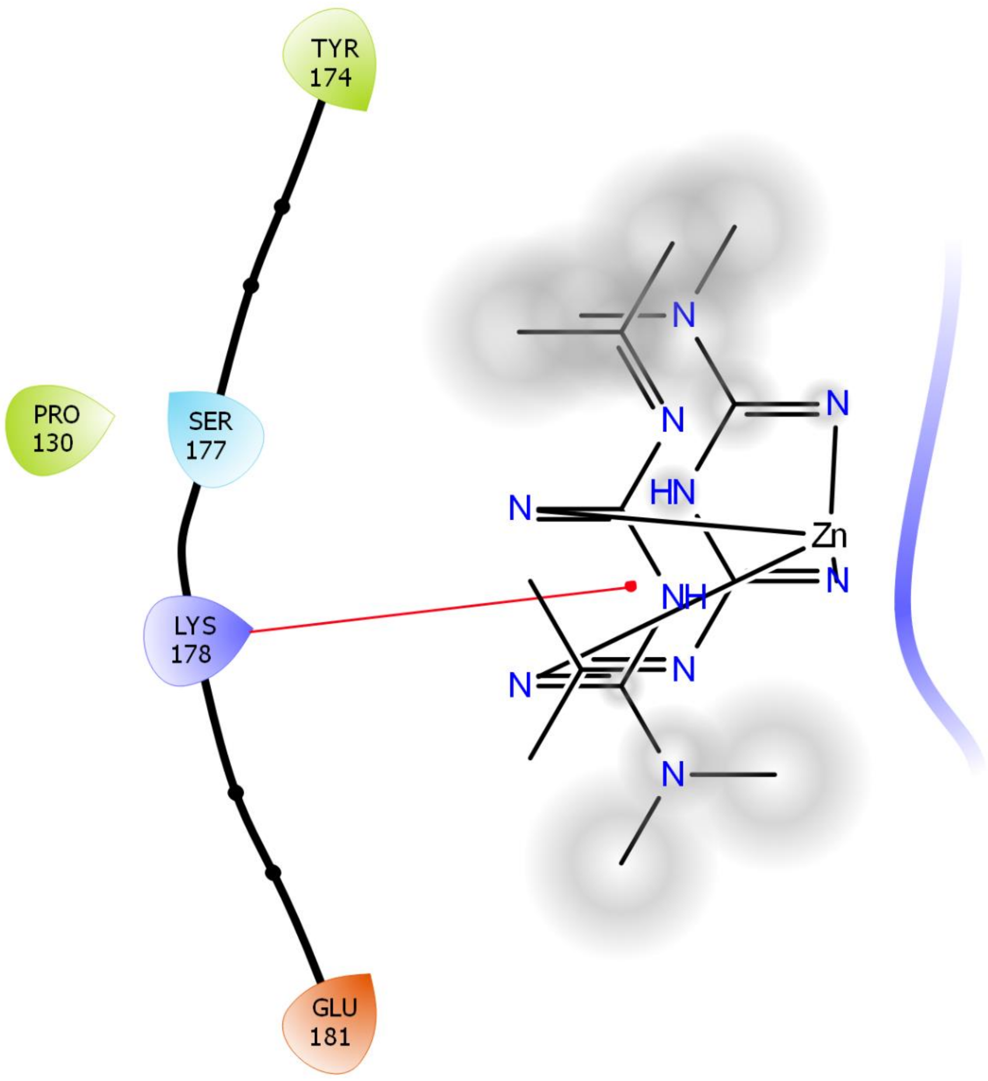

MZN-6

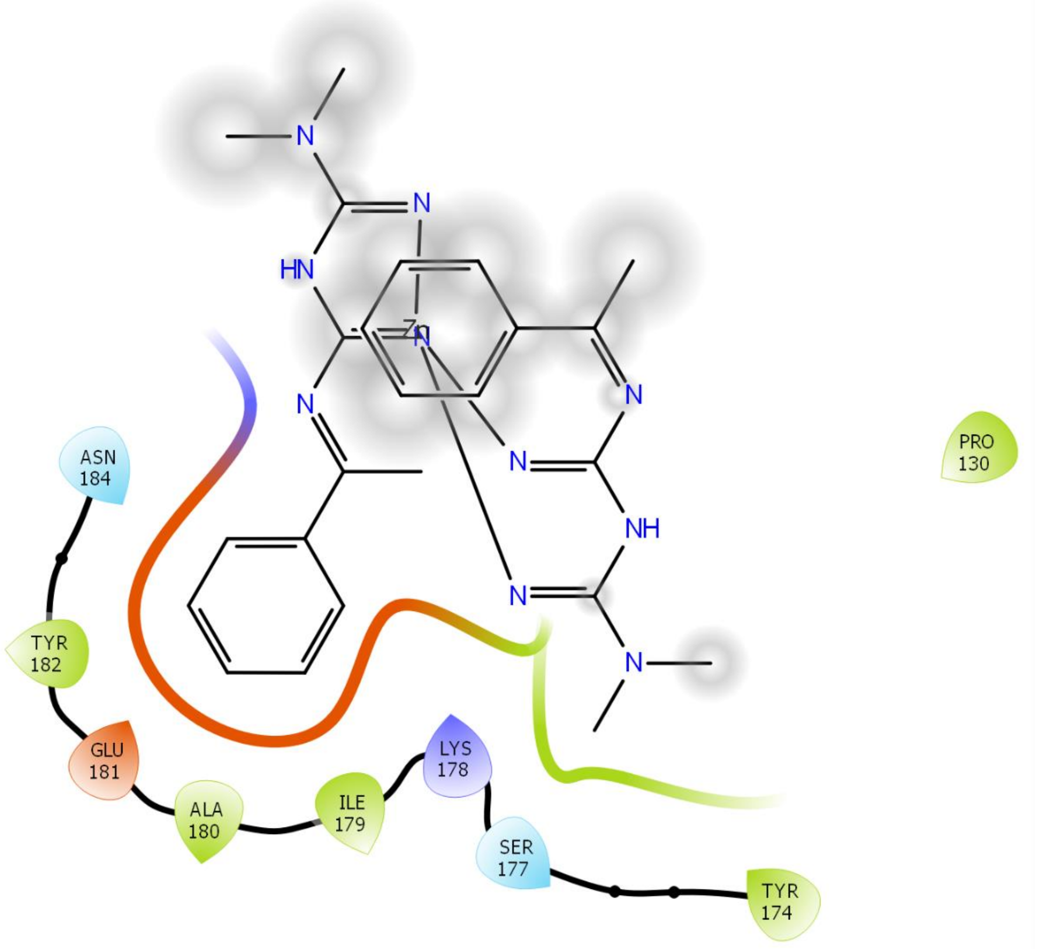

N
N
HN
Zn
N
N
N
N
NH
N
N
ASN
184
TYR
182
GLU
181
ALA
180
ILE
179
LYS
178
SER
177
TYR
174
PRO
130

MZN-7	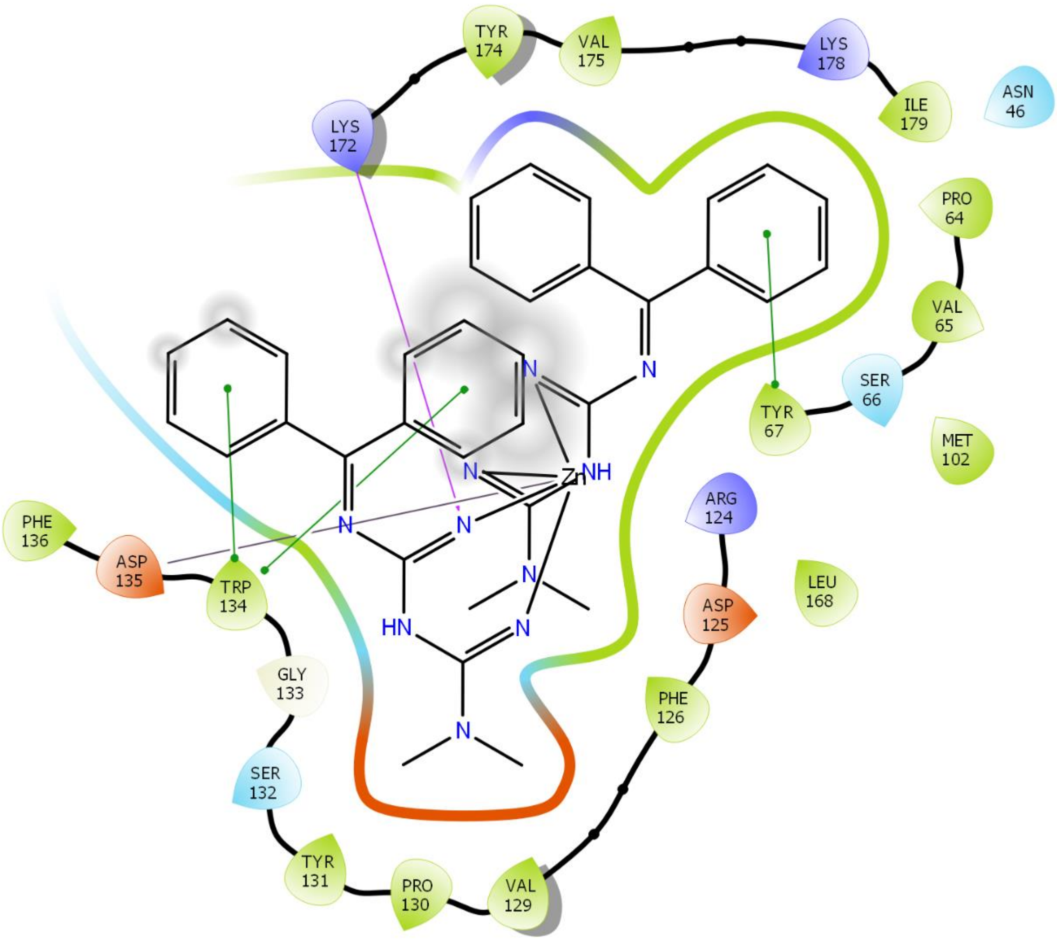

TYR 174
VAL 175
LYS 178
ILE 179
ASN 46
LYS 172
PRO 64
VAL 65
SER 66
TYR 67
MET 102
ARG 124
ASP 125
LEU 168
PHE 126
PHE 136
ASP 135
TRP 134
GLY 133
SER 132
TYR 131
PRO 130
VAL 129
N
NH
HN
Zn

MZN-8

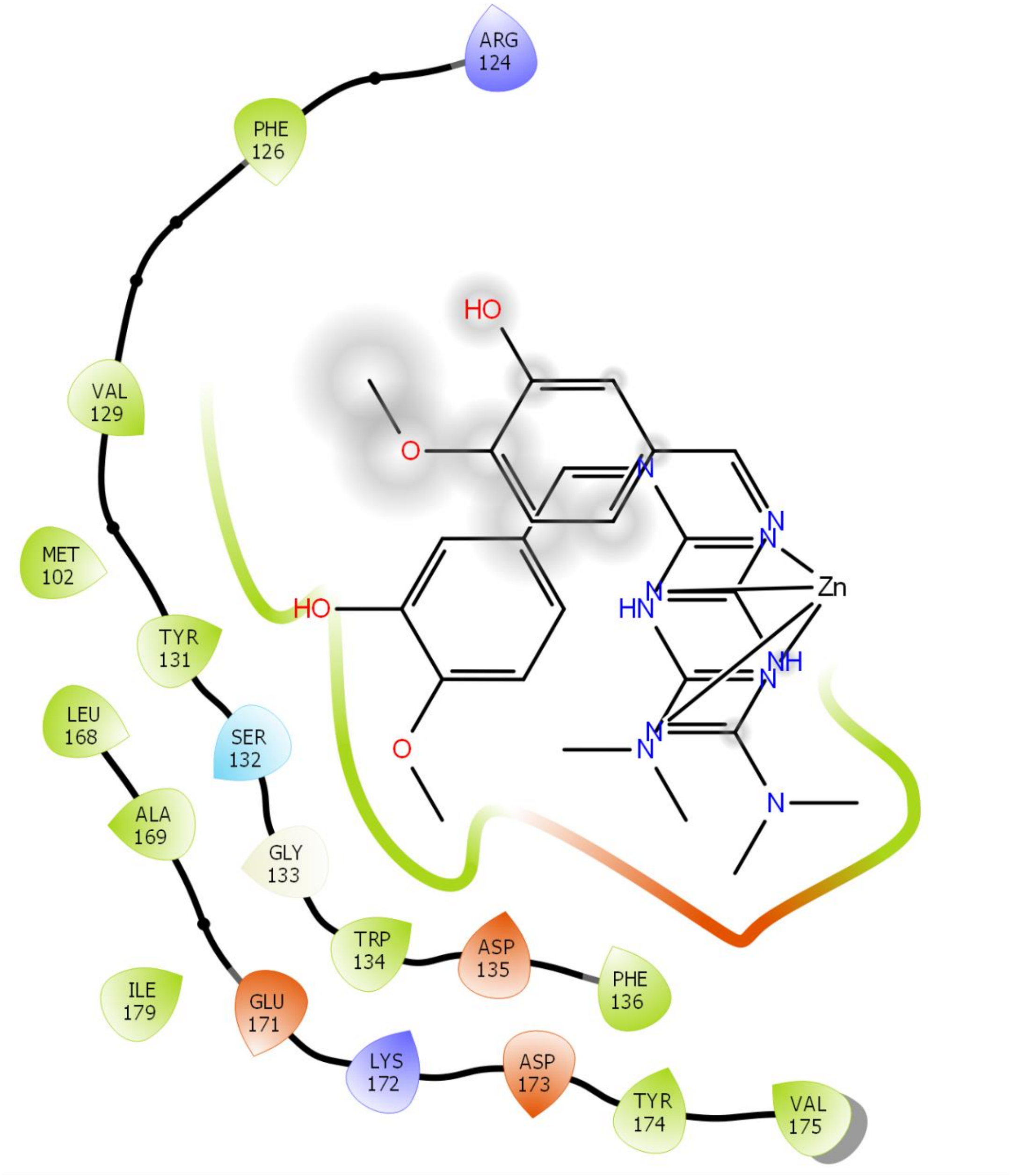
ARG 124
PHE 126
VAL 129
MET 102
TYR 131
LEU 168
SER 132
ALA 169
GLY 133
TRP 134
ASP 135
PHE 136
ILE 179
GLU 171
LYS 172
ASP 173
TYR 174
VAL 175
HO
O
HO
O
N
N
N
HN
Zn
NH
N
N
N
N

MZN-9

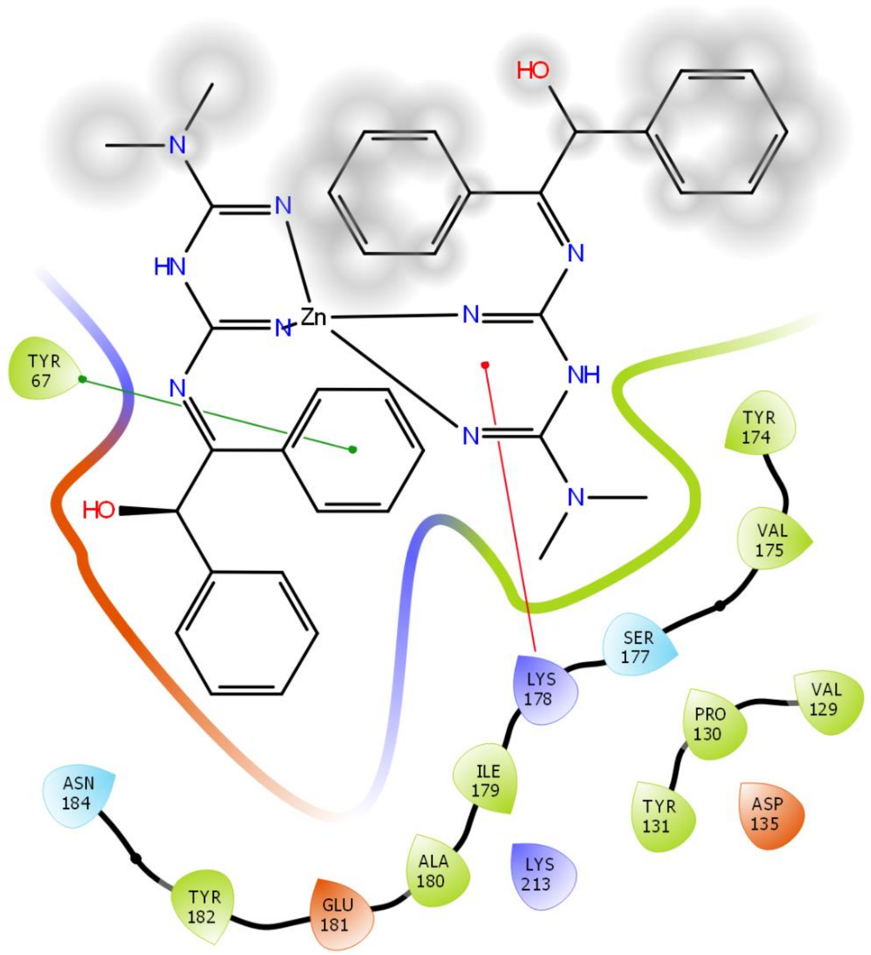

HO
N
N
HN
Zn
N
N
N
NH
N
N
HO
TYR 67
TYR 174
VAL 175
SER 177
LYS 178
VAL 129
PRO 130
ILE 179
ASN 184
TYR 131
ASP 135
ALA 180
LYS 213
TYR 182
GLU 181

MCu-1

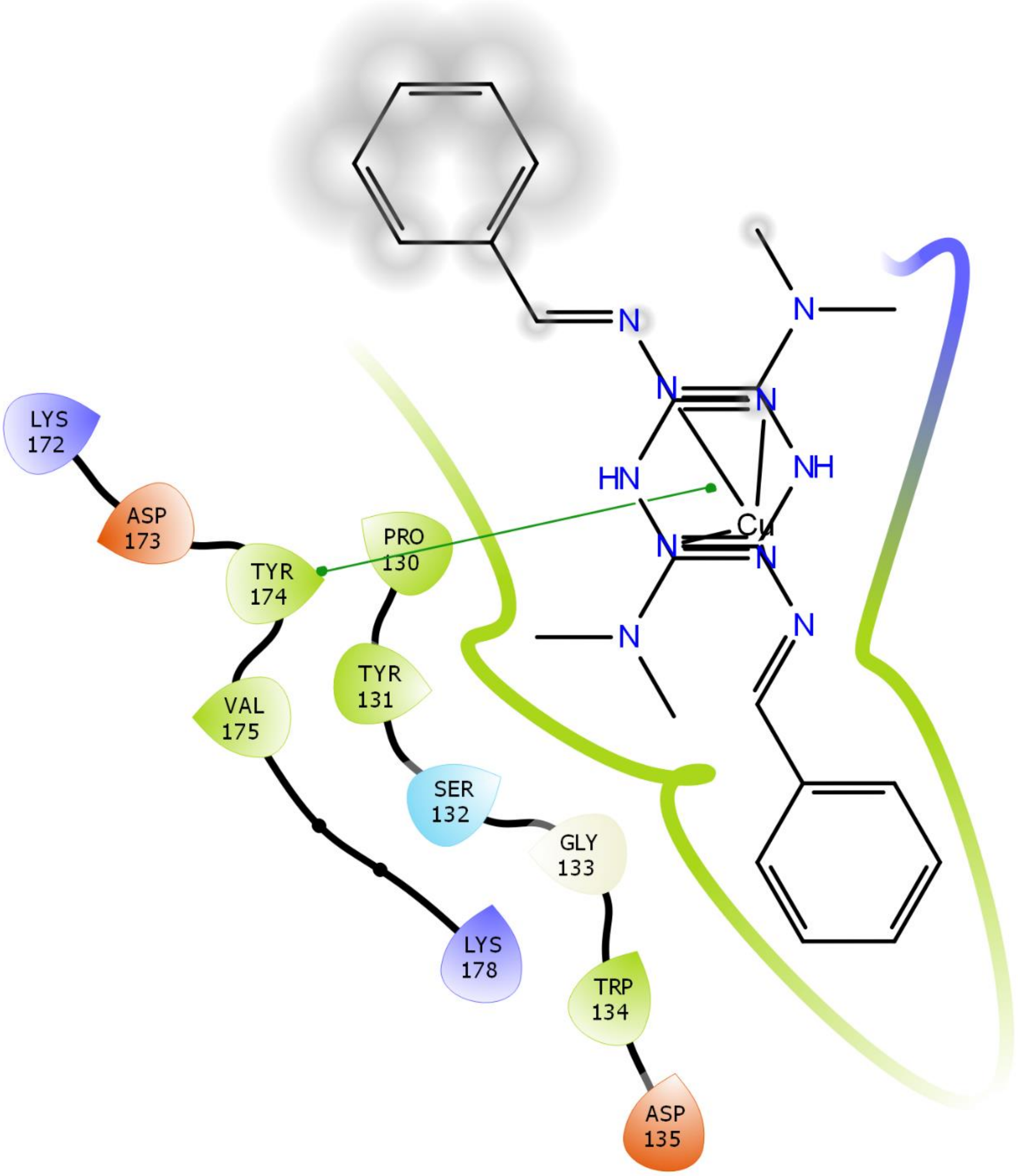

LYS
172
ASP
173
TYR
174
VAL
175
LYS
178
PRO
130
TYR
131
SER
132
GLY
133
TRP
134
ASP
135
N
HN
NH
Cu

MCu-2	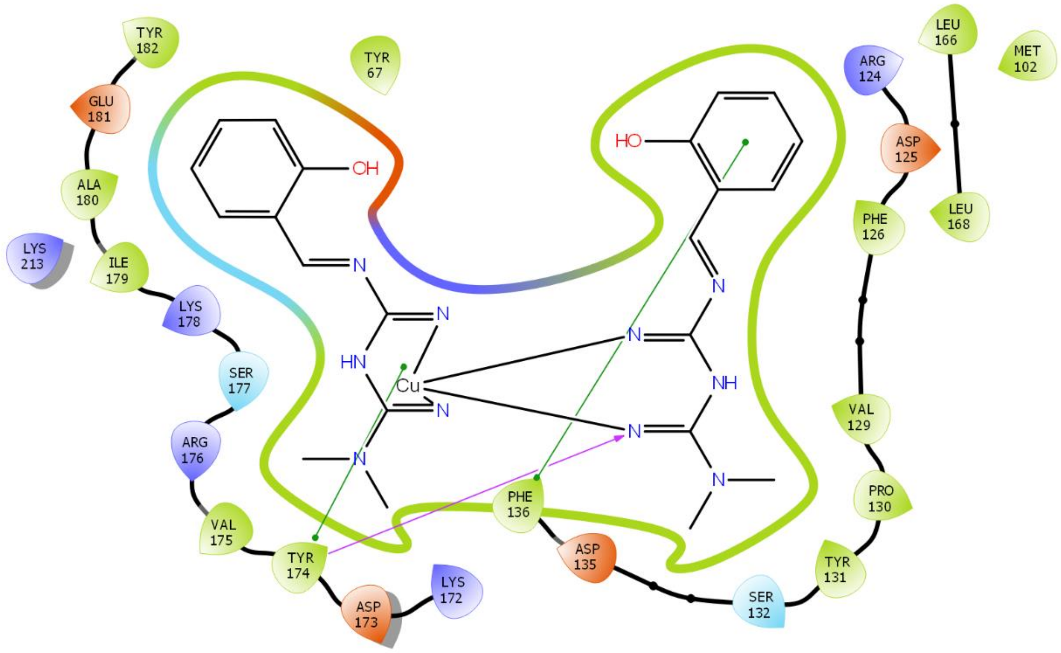

TYR 182
GLU 181
ALA 180
LYS 213
ILE 179
LYS 178
SER 177
ARG 176
VAL 175
TYR 174
ASP 173
LYS 172
TYR 67
PHE 136
ASP 135
SER 132
TYR 131
PRO 130
VAL 129
PHE 126
ASP 125
ARG 124
LEU 166
LEU 168
MET 102
OH
HO
N
HN
NH
Cu

MCu-3	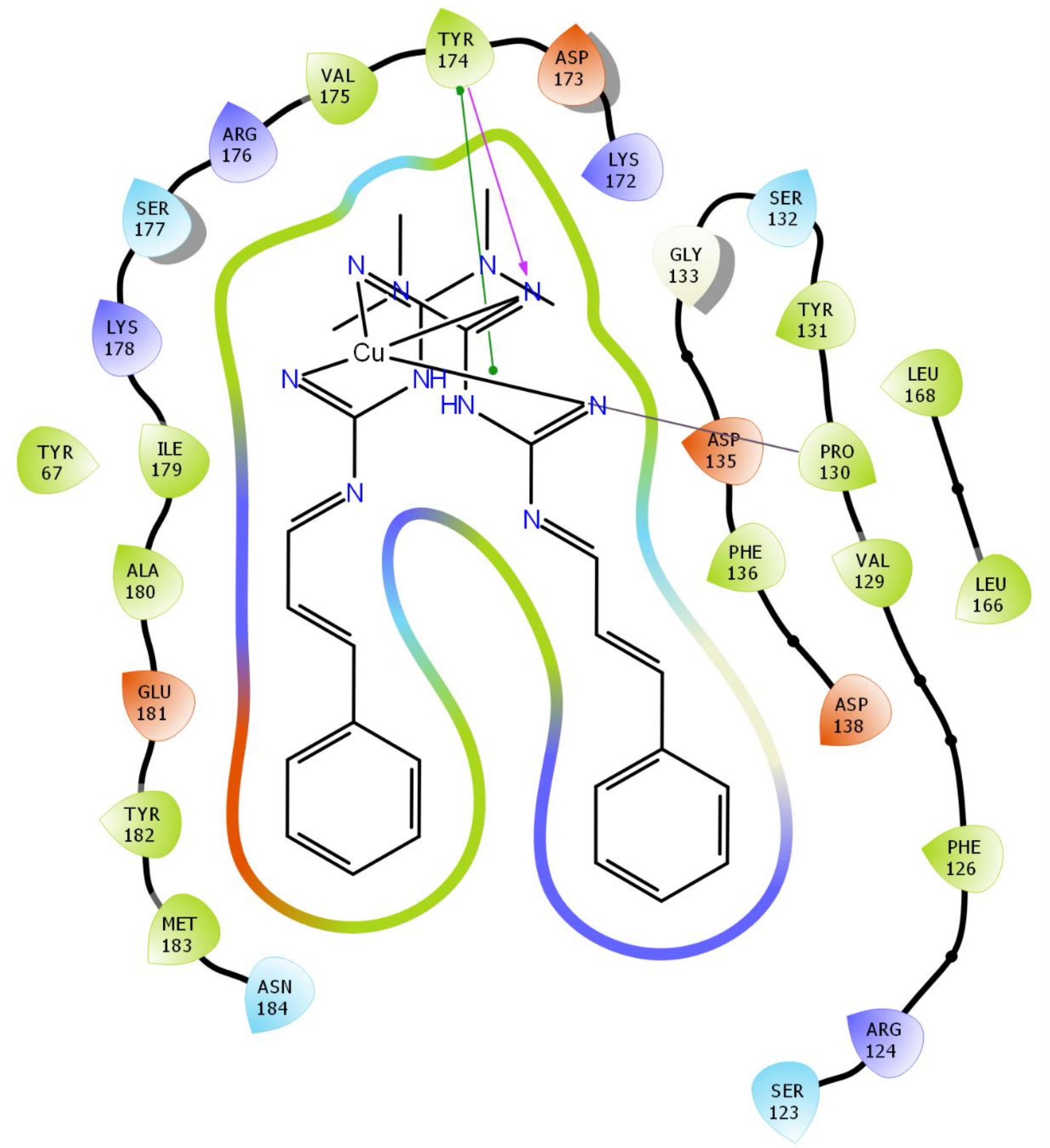TYR 174<br>ASP 173<br>VAL 175<br>ARG 176<br>LYS 172<br>SER 177<br>SER 132<br>GLY 133<br>LYS 178<br>TYR 131<br>LEU 168<br>TYR 67<br>ILE 179<br>ASP 135<br>PRO 130<br>PHE 136<br>VAL 129<br>LEU 166<br>ALA 180<br>GLU 181<br>ASP 138<br>TYR 182<br>PHE 126<br>MET 183<br>ASN 184<br>ARG 124<br>SER 123<br>Cu<br>N<br>NH<br>HN

MCu-4

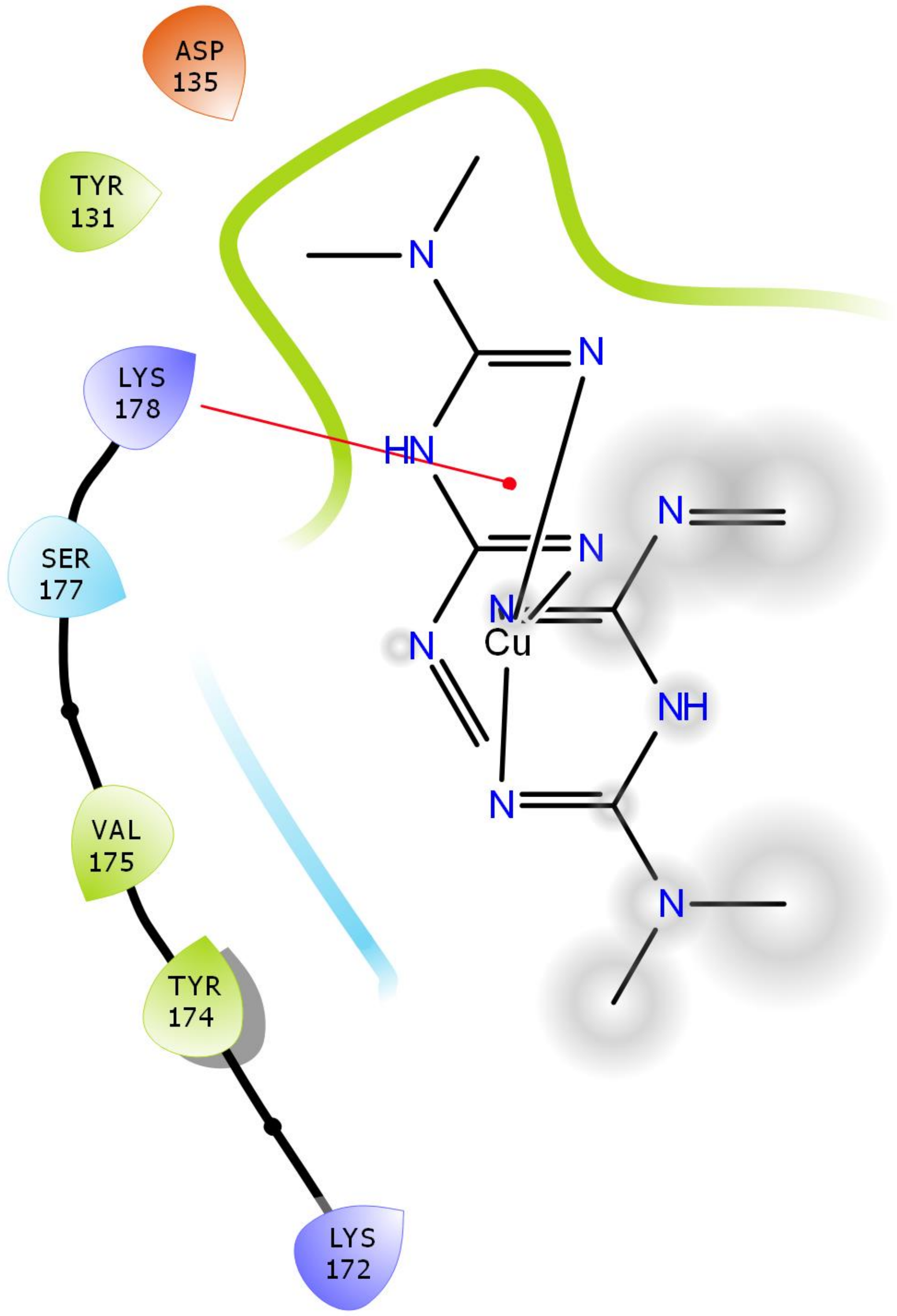
ASP
135
TYR
131
LYS
178
SER
177
VAL
175
TYR
174
LYS
172
N
N
HN
N
N
Cu
N
N
NH
N
N

MCu-5	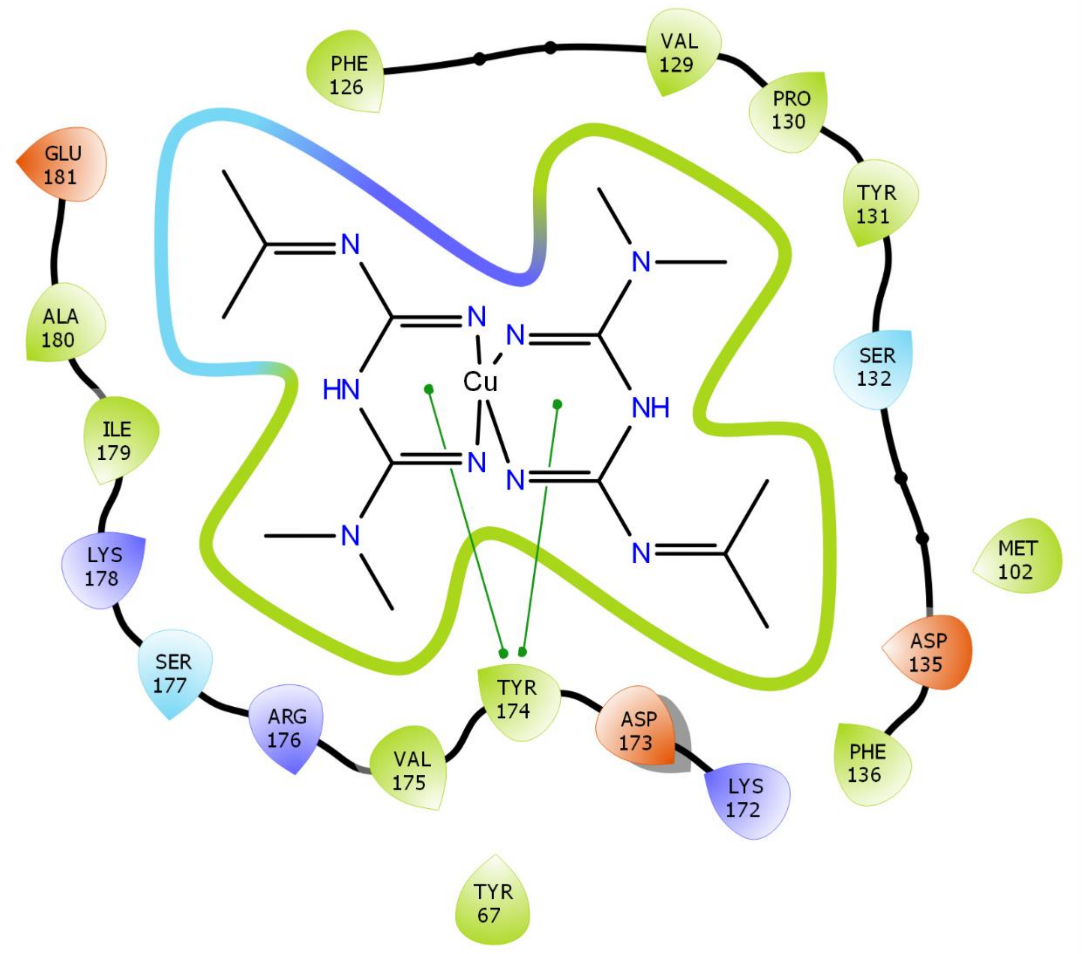

PHE 126
VAL 129
PRO 130
TYR 131
SER 132
MET 102
ASP 135
PHE 136
LYS 172
ASP 173
TYR 174
VAL 175
ARG 176
SER 177
LYS 178
ILE 179
ALA 180
GLU 181
TYR 67
N
HN
NH
Cu

MCu-6	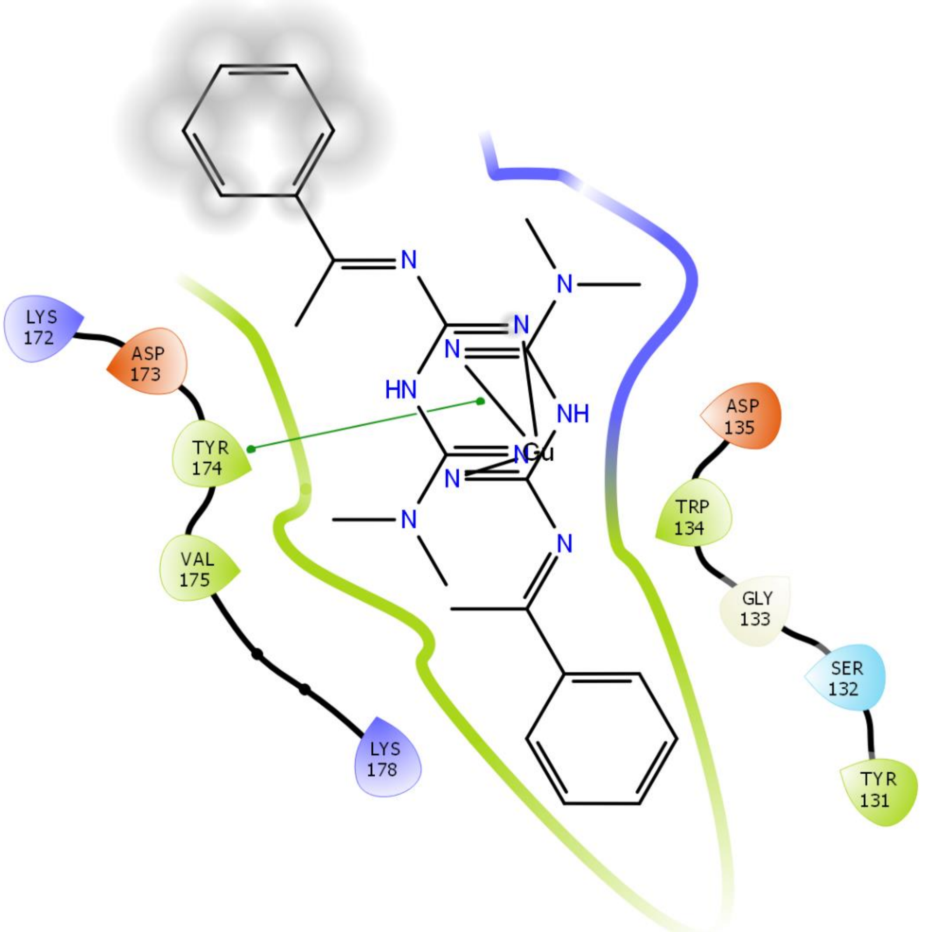LYS 172<br>ASP 173<br>TYR 174<br>VAL 175<br>LYS 178<br>ASP 135<br>TRP 134<br>GLY 133<br>SER 132<br>TYR 131<br>N<br>HN<br>NH<br>Cu

MCu-7	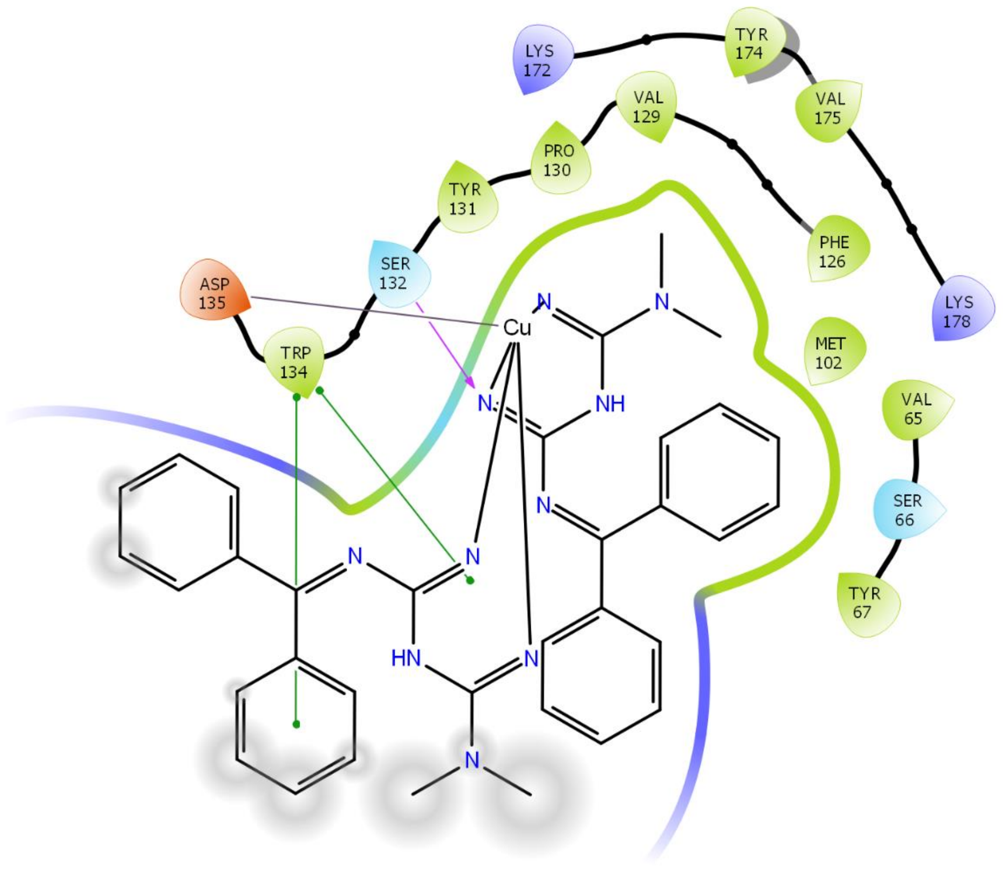LYS 172<br>TYR 174<br>VAL 129<br>VAL 175<br>PRO 130<br>TYR 131<br>SER 132<br>ASP 135<br>PHE 126<br>LYS 178<br>TRP 134<br>MET 102<br>VAL 65<br>SER 66<br>TYR 67<br>Cu<br>N<br>NH<br>HN

MCu-8

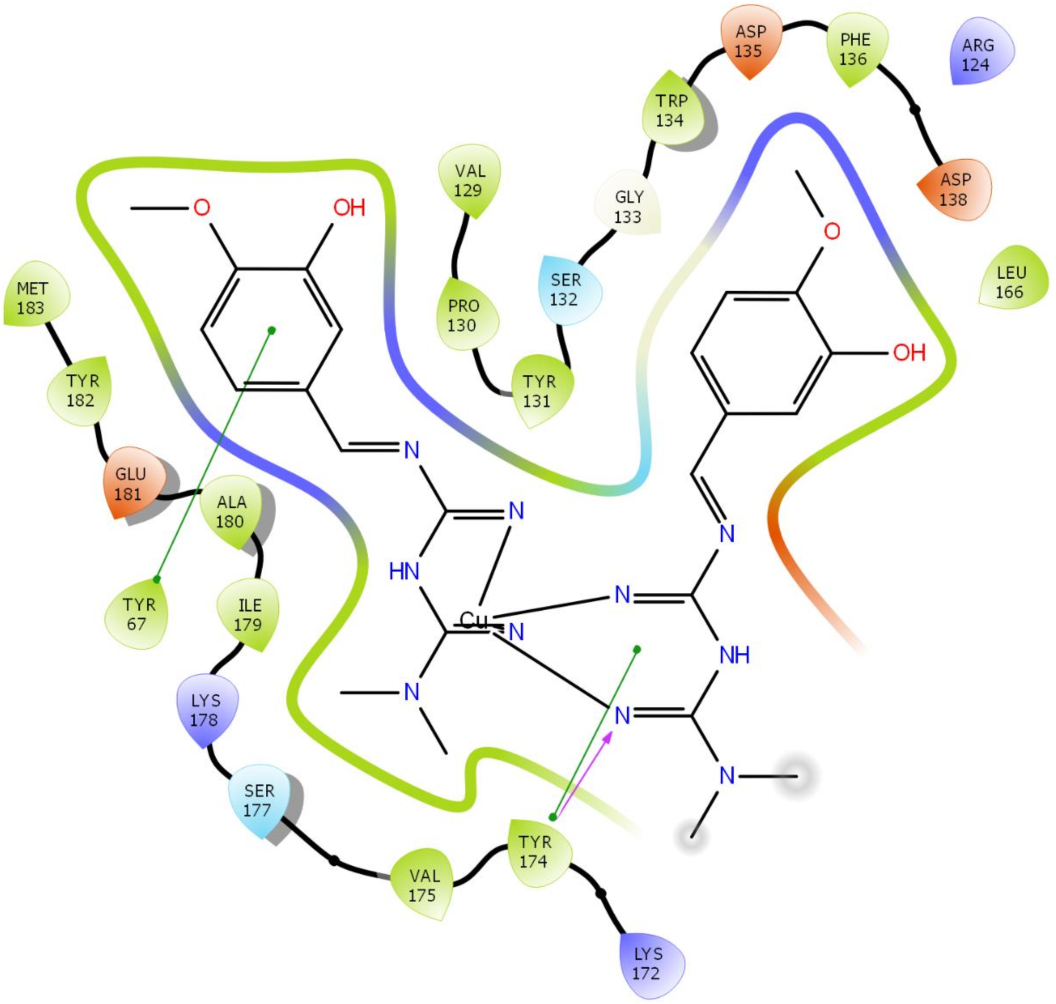

ASP 135
PHE 136
ARG 124
TRP 134
ASP 138
VAL 129
GLY 133
OH
O
SER 132
LEU 166
MET 183
PRO 130
OH
TYR 182
TYR 131
N
GLU 181
ALA 180
N
N
HN
N
TYR 67
ILE 179
Cu
N
NH
LYS 178
N
N
N
SER 177
TYR 174
VAL 175
LYS 172

MCu-9	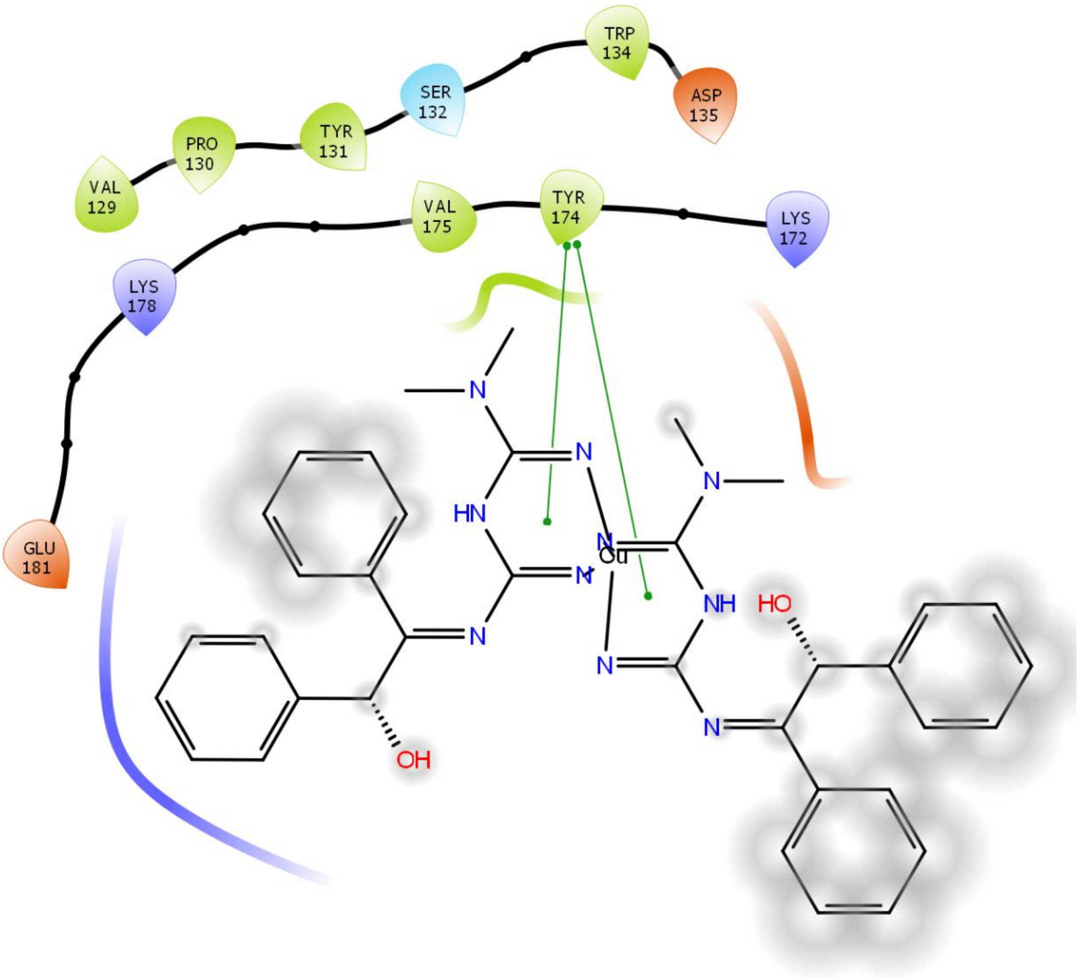

TRP 134
ASP 135
SER 132
TYR 131
PRO 130
VAL 129
VAL 175
TYR 174
LYS 172
LYS 178
GLU 181
N
N
HN
N
N
Cu
N
N
NH
HO
N
N
OH

Table S3. 2D structures of all Metformin derivatives with respect to glucosidase enzyme.

Derivative code	2D Pose
MET-1	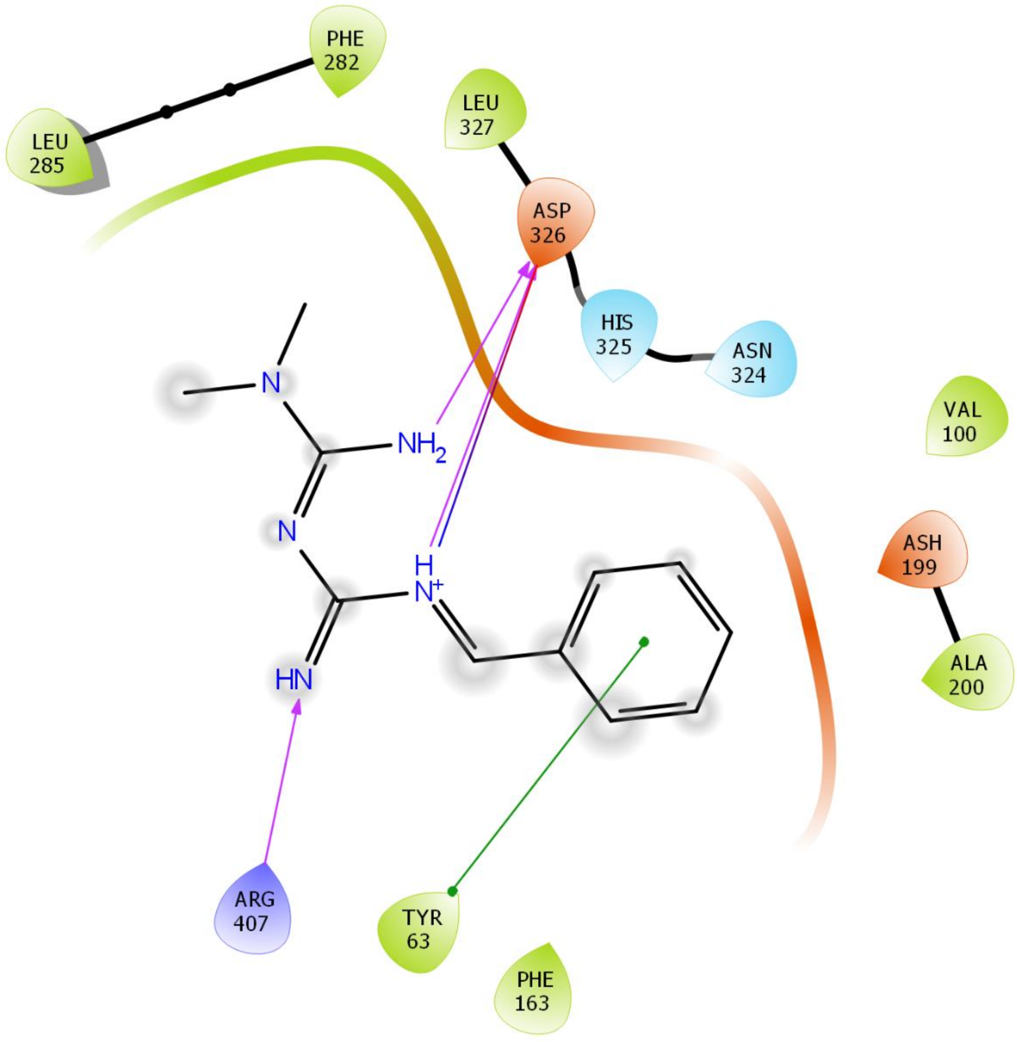

MET-2	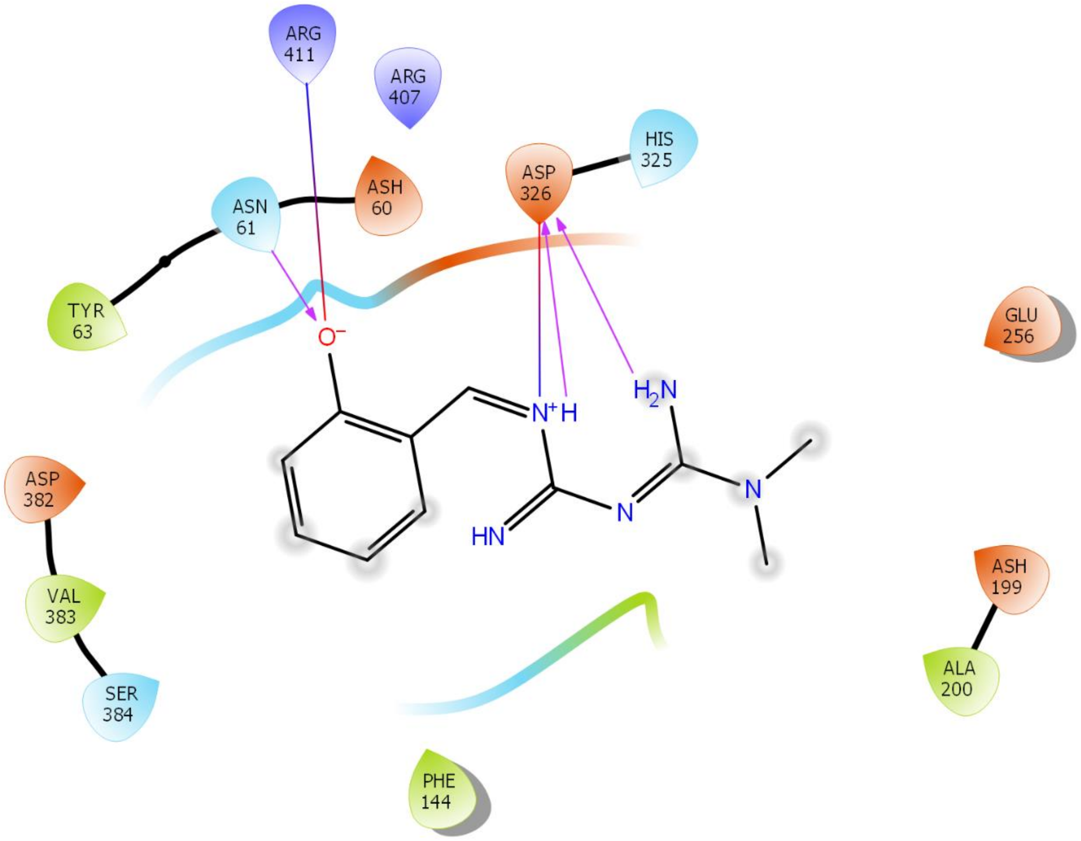 ARG 411<br>ARG 407<br>HIS 325<br>ASP 326<br>ASN 61<br>ASH 60<br>TYR 63<br>GLU 256<br>O−<br>N+H<br>H2N<br>HN<br>N<br>N<br>ASP 382<br>VAL 383<br>SER 384<br>ASH 199<br>ALA 200<br>PHE 144

MET-3	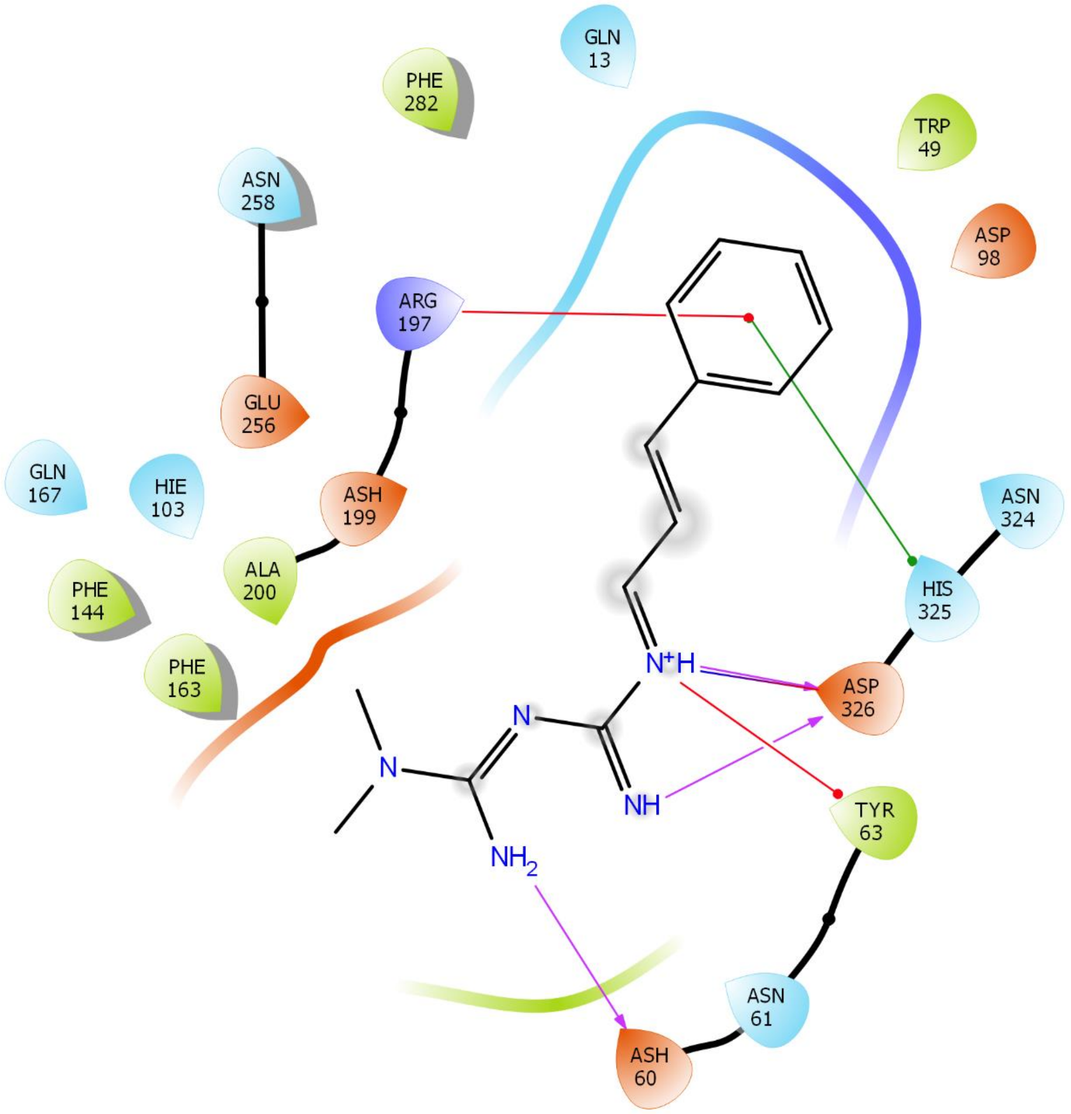

PHE 282
GLN 13
TRP 49
ASN 258
ASP 98
ARG 197
GLU 256
GLN 167
HIE 103
ASH 199
ASN 324
ALA 200
HIS 325
PHE 144
PHE 163
N⁺H
ASP 326
N
N
NH
TYR 63
NH2
ASN 61
ASH 60

MET-4	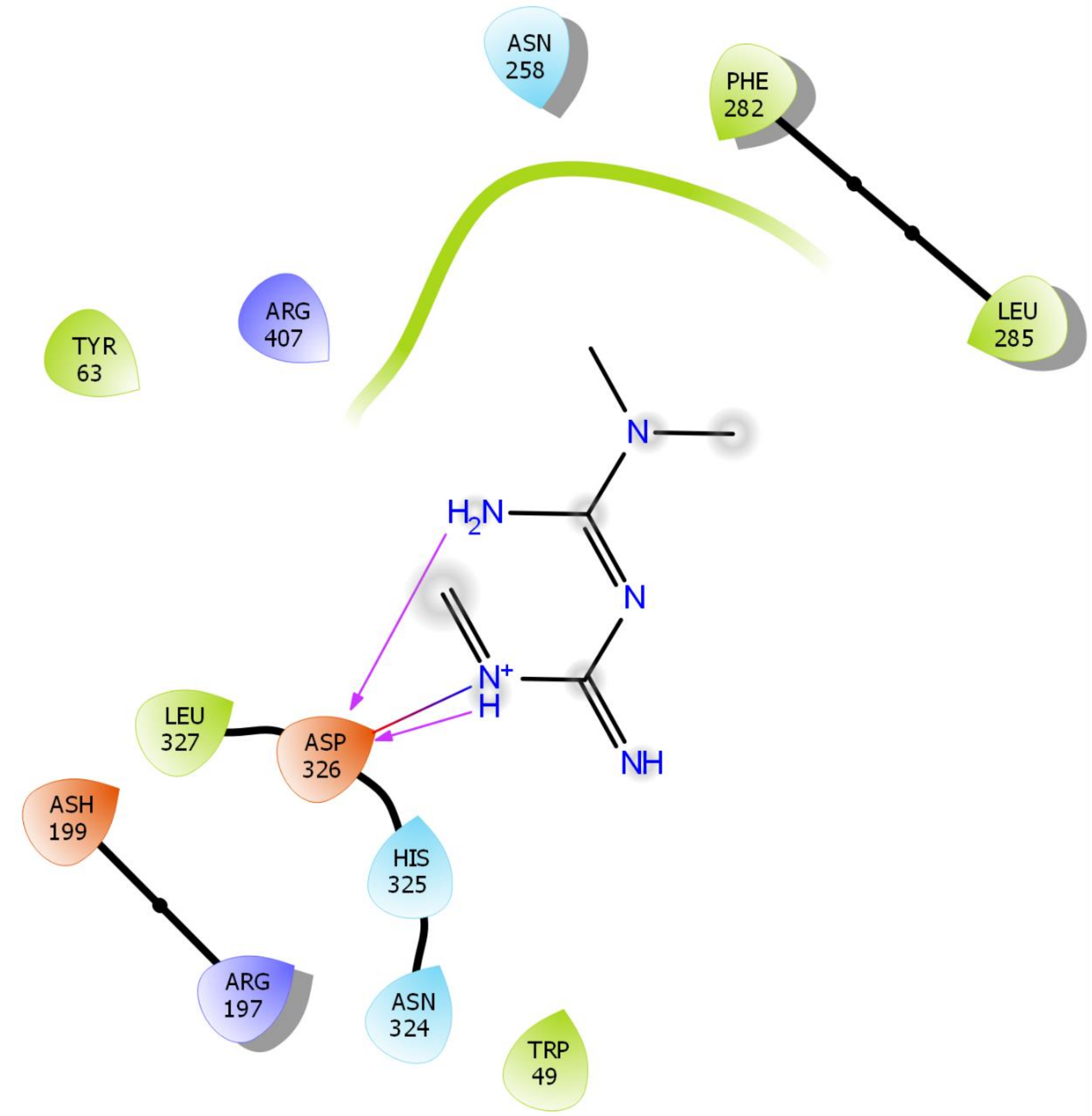

ASN
258
PHE
282
LEU
285
TYR
63
ARG
407
N
H_2N
N
N^+
H
NH
LEU
327
ASP
326
ASH
199
HIS
325
ARG
197
ASN
324
TRP
49

MET-5	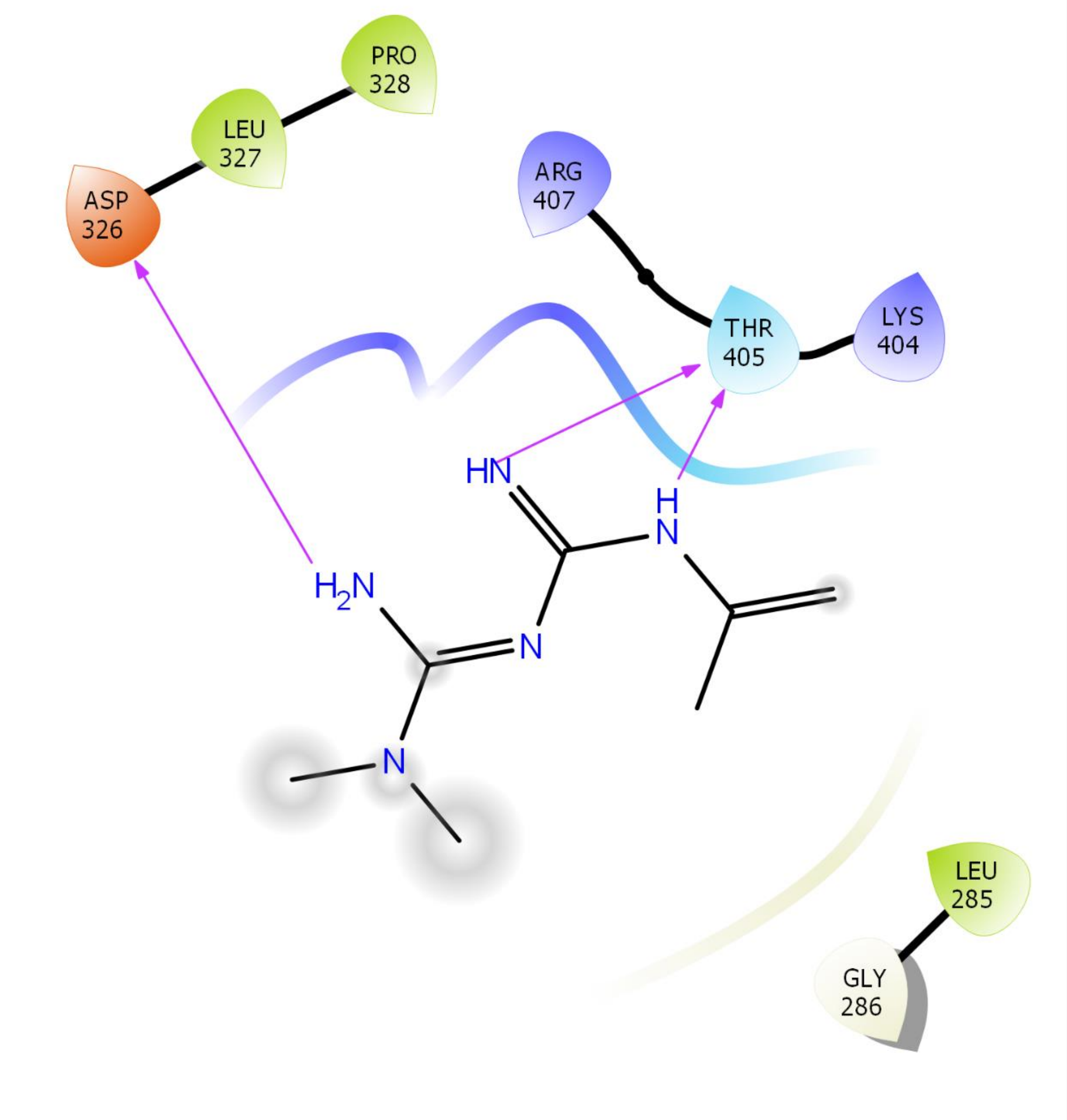PRO 328<br>LEU 327<br>ASP 326<br>ARG 407<br>THR 405<br>LYS 404<br>HN<br>H N<br>H_2N<br>N<br>N<br>LEU 285<br>GLY 286

MET-6	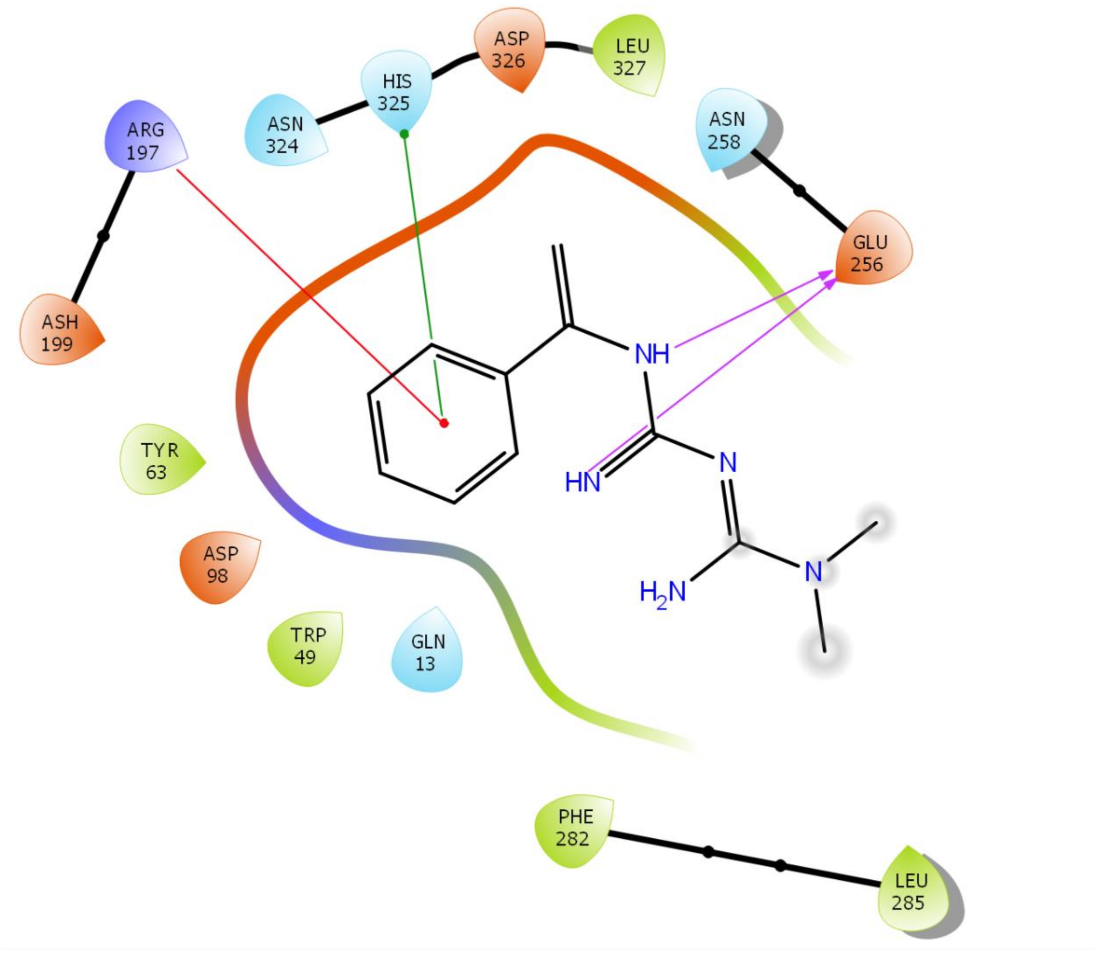

ASP 326
LEU 327
HIS 325
ASN 324
ARG 197
ASN 258
GLU 256
ASH 199
NH
TYR 63
HN
N
ASP 98
N
H_2N
TRP 49
GLN 13
PHE 282
LEU 285

MET-7	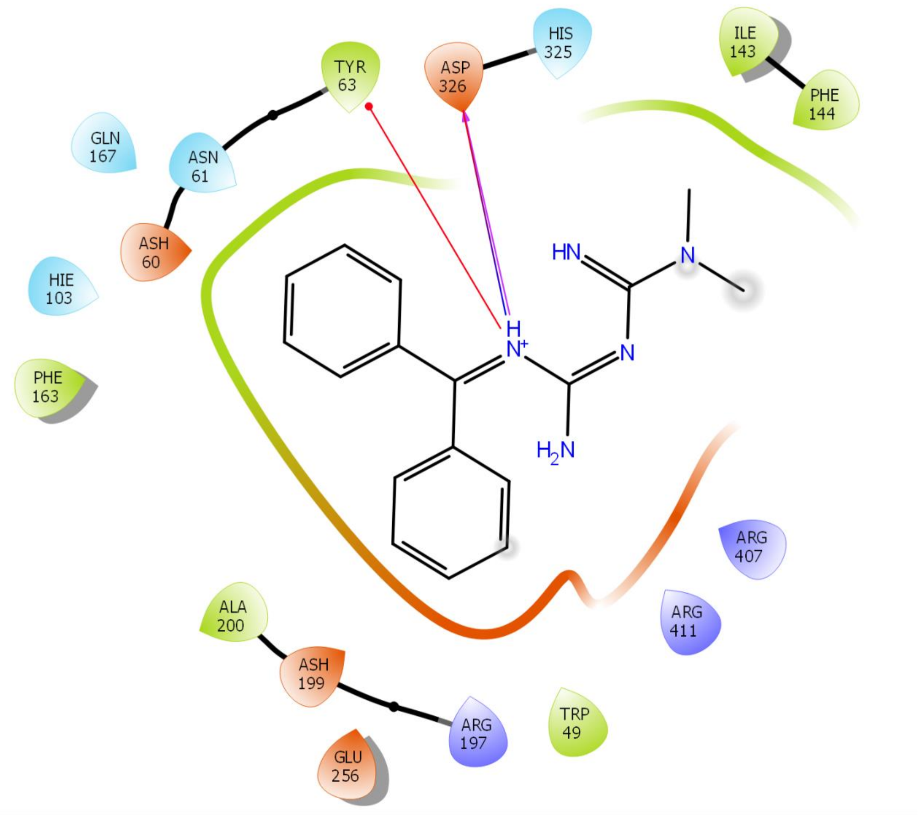 TYR 63<br>ASP 326<br>HIS 325<br>ILE 143<br>PHE 144<br>GLN 167<br>ASN 61<br>ASH 60<br>HIE 103<br>PHE 163<br>HN<br>N<br>H<br>N+<br>N<br>H2N<br>ARG 407<br>ARG 411<br>ALA 200<br>ASH 199<br>ARG 197<br>TRP 49<br>GLU 256
MET-8	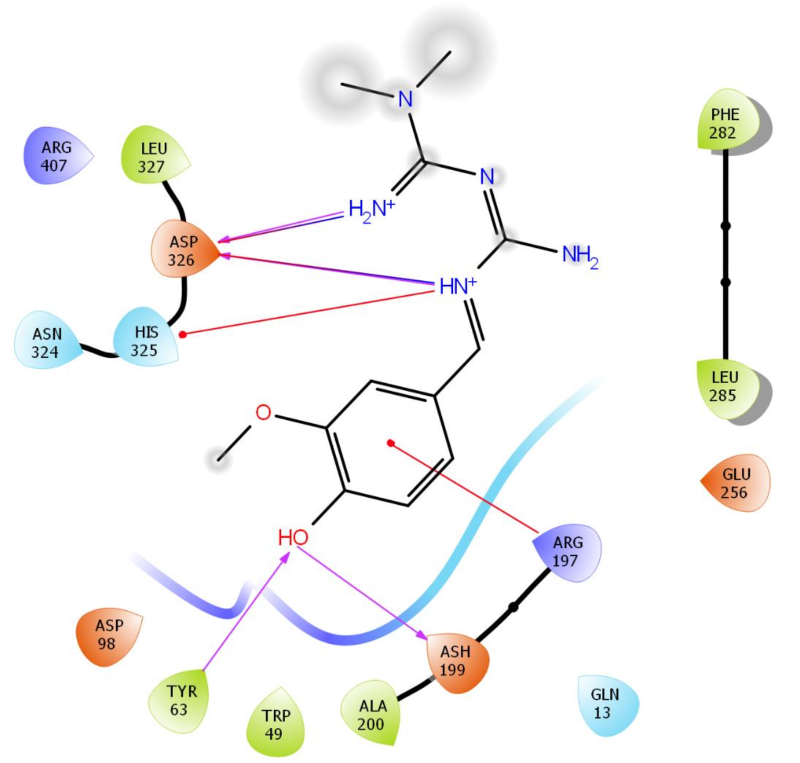 N<br>N<br>PHE 282<br>ARG 407<br>LEU 327<br>H2N+<br>ASP 326<br>NH2<br>HN+<br>ASN 324<br>HIS 325<br>LEU 285<br>O<br>GLU 256<br>HO<br>ARG 197<br>ASP 98<br>ASH 199<br>TYR 63<br>TRP 49<br>ALA 200<br>GLN 13

MET-9	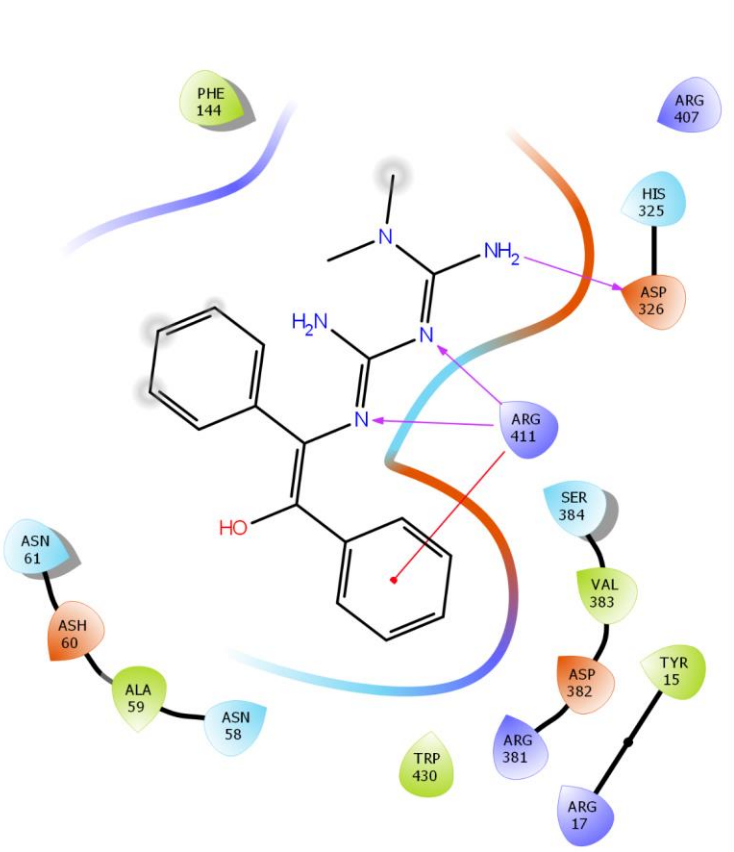PHE 144<br>ARG 407<br>HIS 325<br>NH2<br>ASP 326<br>N<br>H2N<br>ARG 411<br>SER 384<br>HO<br>ASN 61<br>VAL 383<br>ASH 60<br>ASP 382<br>TYR 15<br>ALA 59<br>ASN 58<br>ARG 381<br>TRP 430<br>ARG 17
MET	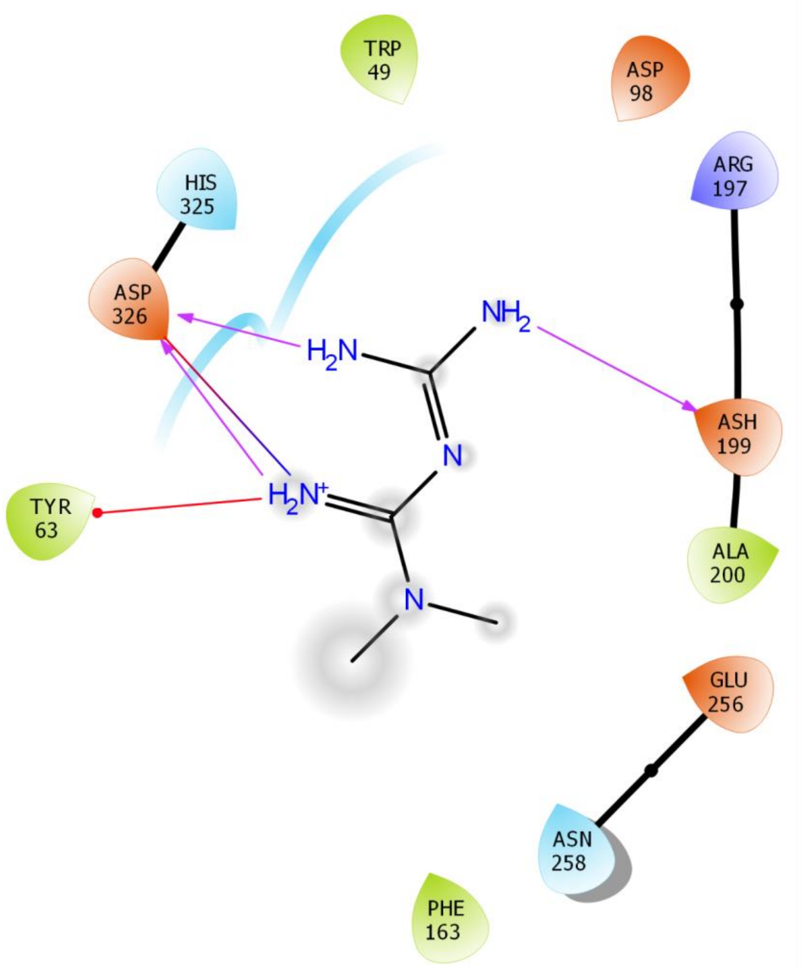TRP 49<br>ASP 98<br>HIS 325<br>ARG 197<br>ASP 326<br>NH2<br>H2N<br>ASH 199<br>N<br>TYR 63<br>H2N+<br>ALA 200<br>N<br>GLU 256<br>ASN 258<br>PHE 163

MZN-1	
MZN-2	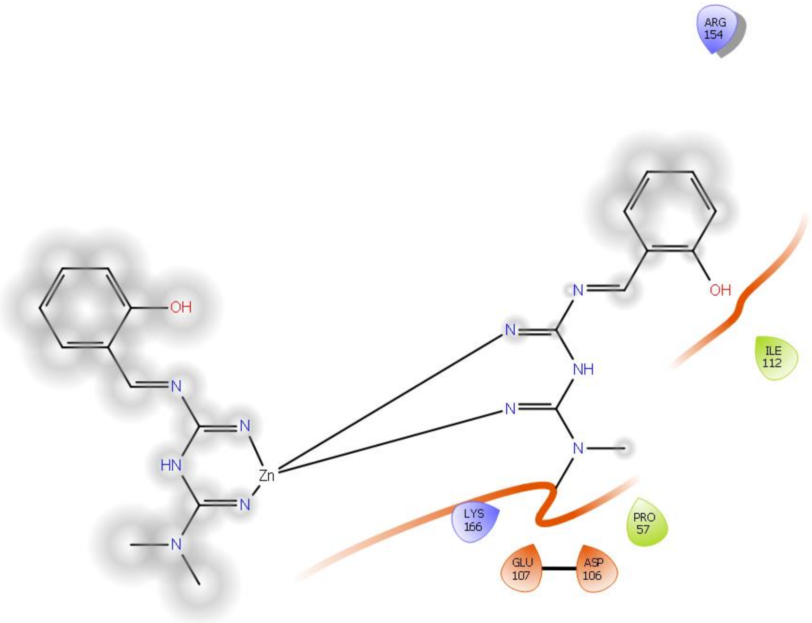
MZN-3	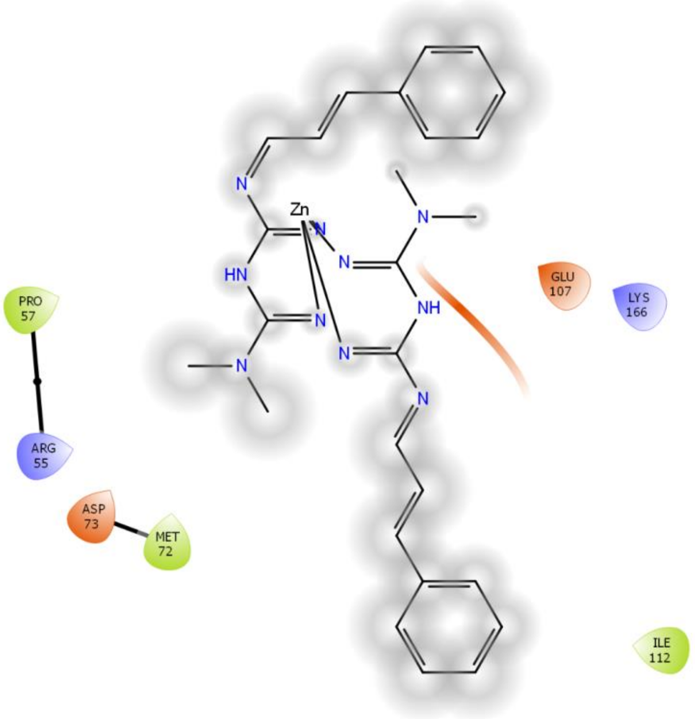
MZN-4	

MZN-5	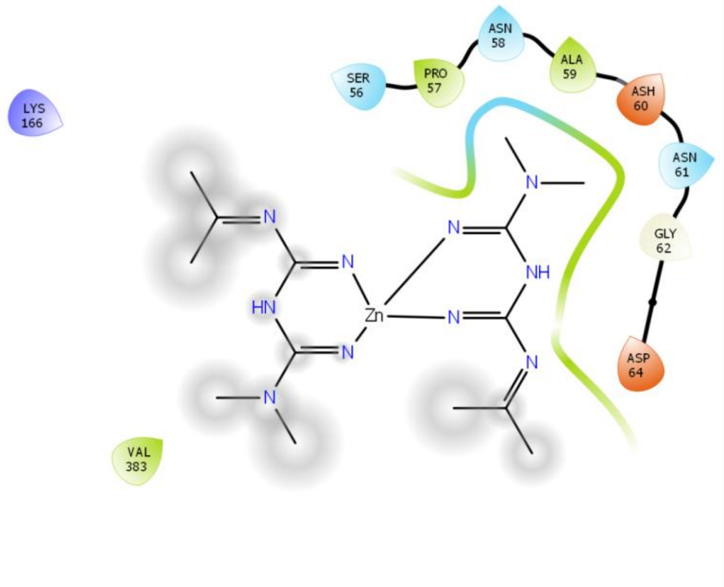 SER 56<br>PRO 57<br>ASN 58<br>ALA 59<br>ASH 60<br>ASN 61<br>GLY 62<br>ASP 64<br>LYS 166<br>VAL 383<br>N<br>HN<br>NH<br>Zn
MZN-6	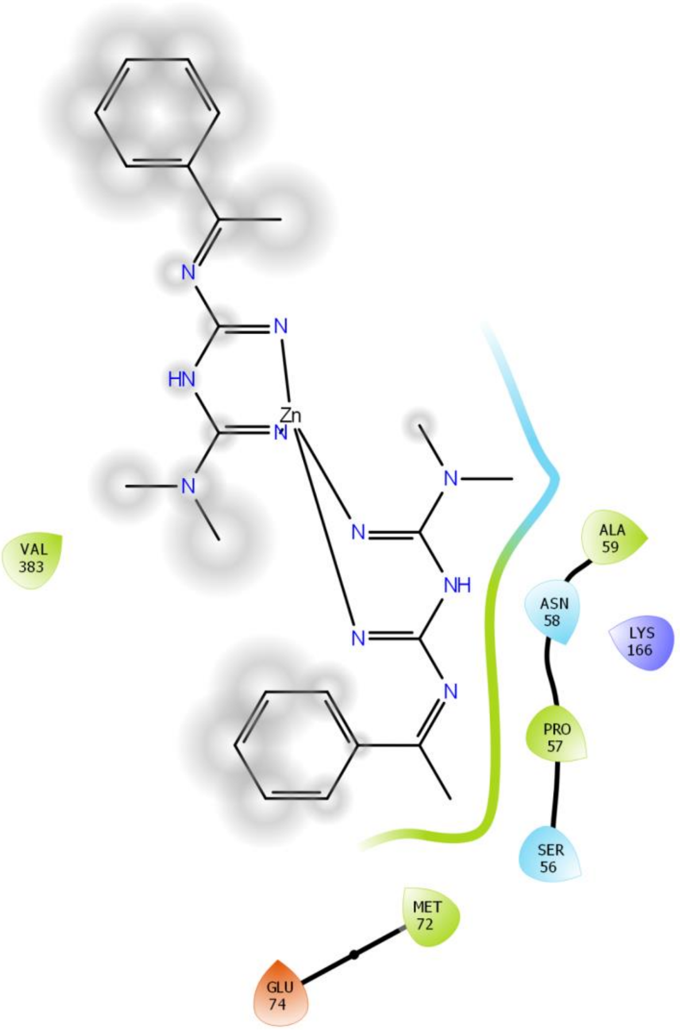 ALA 59<br>ASN 58<br>LYS 166<br>PRO 57<br>SER 56<br>MET 72<br>GLU 74<br>VAL 383<br>N<br>HN<br>NH<br>Zn

MZN-7

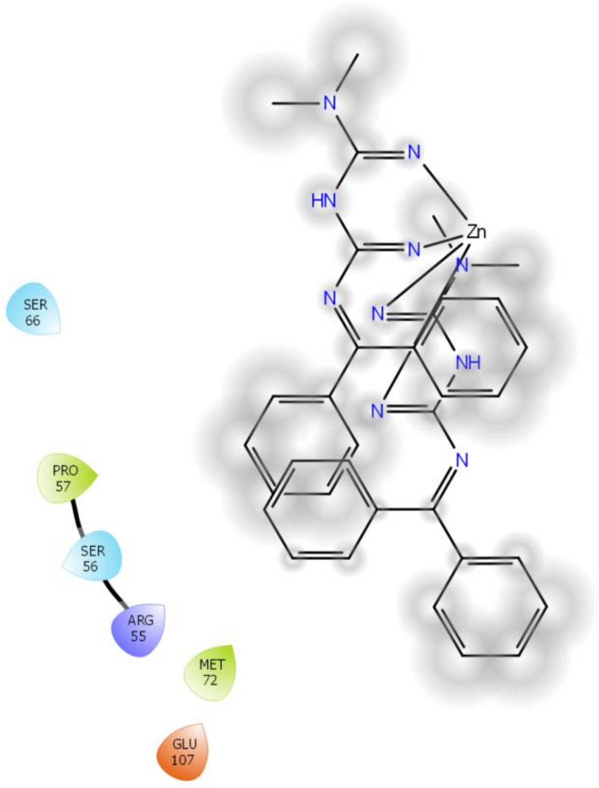

SER 66
PRO 57
SER 56
ARG 55
MET 72
GLU 107
Zn

MZN-8

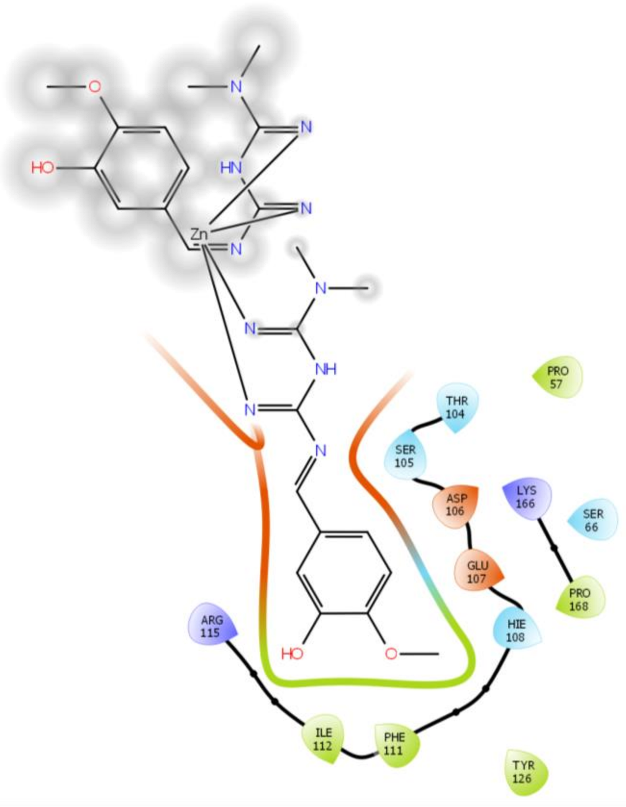

Zn
HO
THR 104
PRO 57
SER 105
ASP 106
LYS 166
SER 66
GLU 107
PRO 168
HIE 108
ARG 115
ILE 112
PHE 111
TYR 126

MZN-9	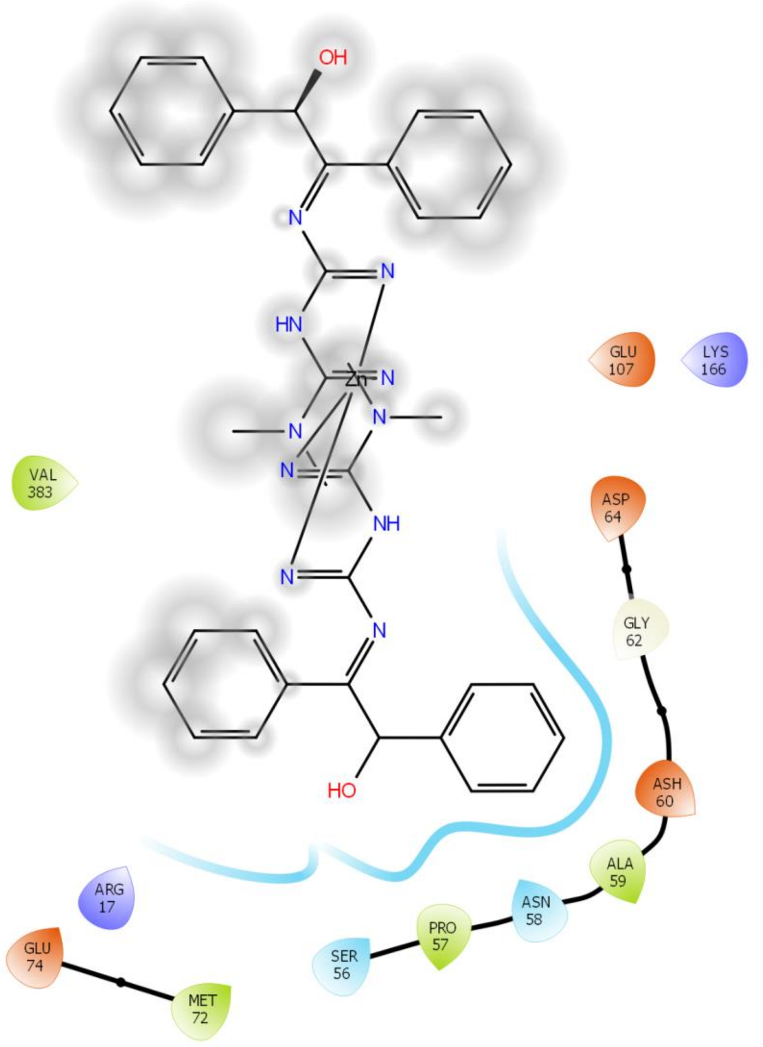 OH<br>N<br>HN<br>Zn<br>N<br>NH<br>HO<br>GLU 107<br>LYS 166<br>VAL 383<br>ASP 64<br>GLY 62<br>ASH 60<br>ALA 59<br>ASN 58<br>PRO 57<br>SER 56<br>ARG 17<br>GLU 74<br>MET 72
MCu-1	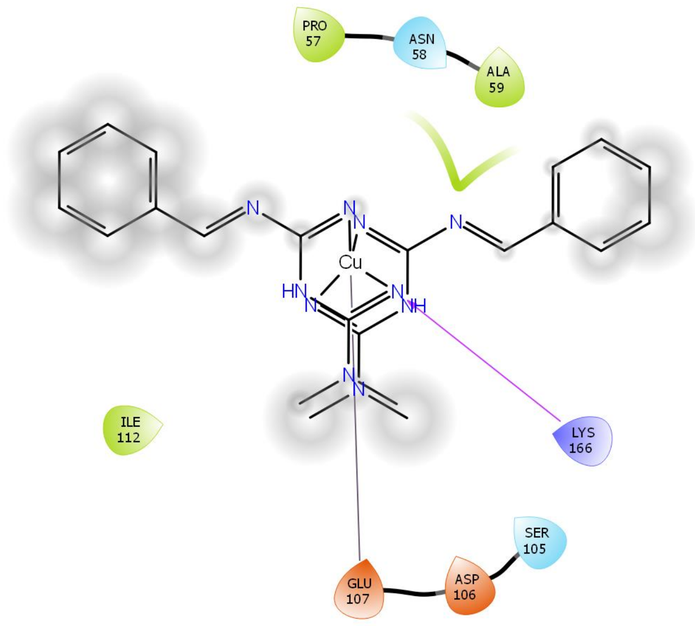 PRO 57<br>ASN 58<br>ALA 59<br>N<br>Cu<br>HN<br>NH<br>ILE 112<br>LYS 166<br>SER 105<br>GLU 107<br>ASP 106

MCu-2	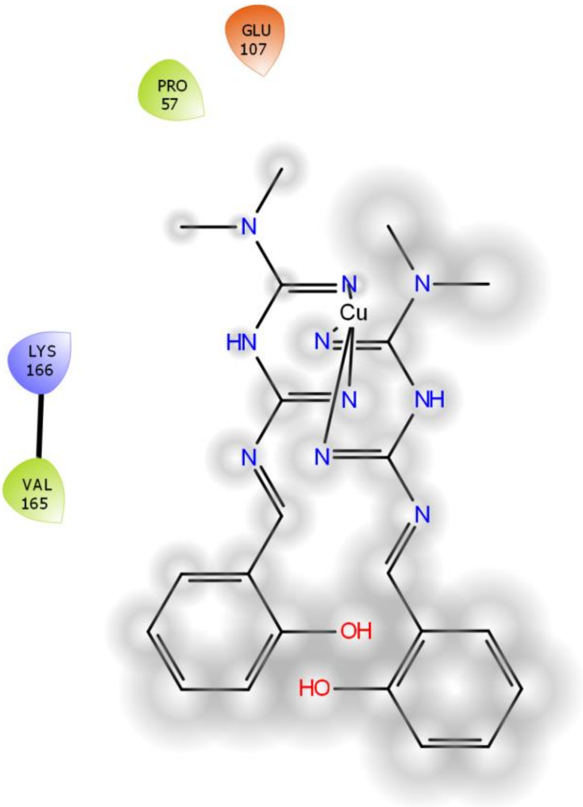GLU 107<br>PRO 57<br>LYS 166<br>VAL 165<br>Cu<br>OH<br>HO
MCu-3	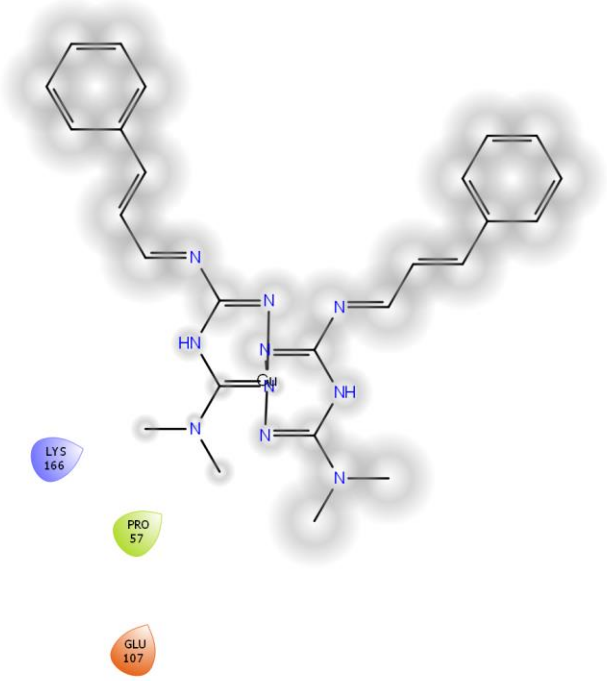LYS 166<br>PRO 57<br>GLU 107<br>Cu

MCu-4

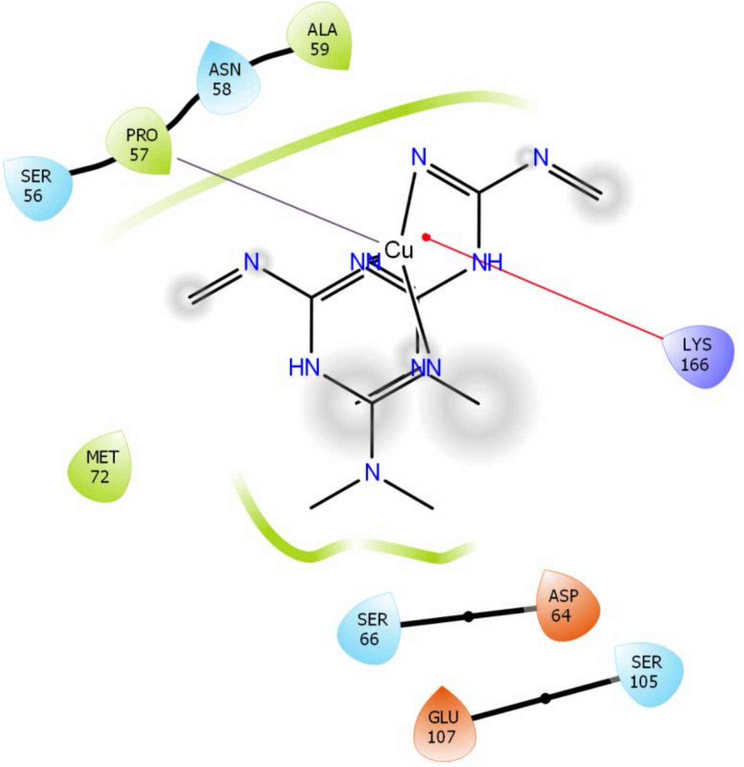
ALA 59
ASN 58
PRO 57
SER 56
LYS 166
MET 72
SER 66
ASP 64
SER 105
GLU 107
Cu
N
NH
HN

MCu-5

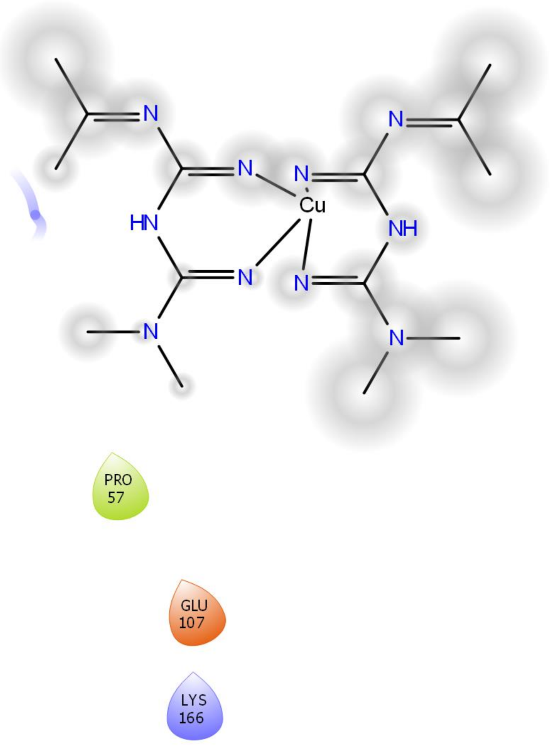
Cu
N
HN
NH
PRO 57
GLU 107
LYS 166

MCu-6	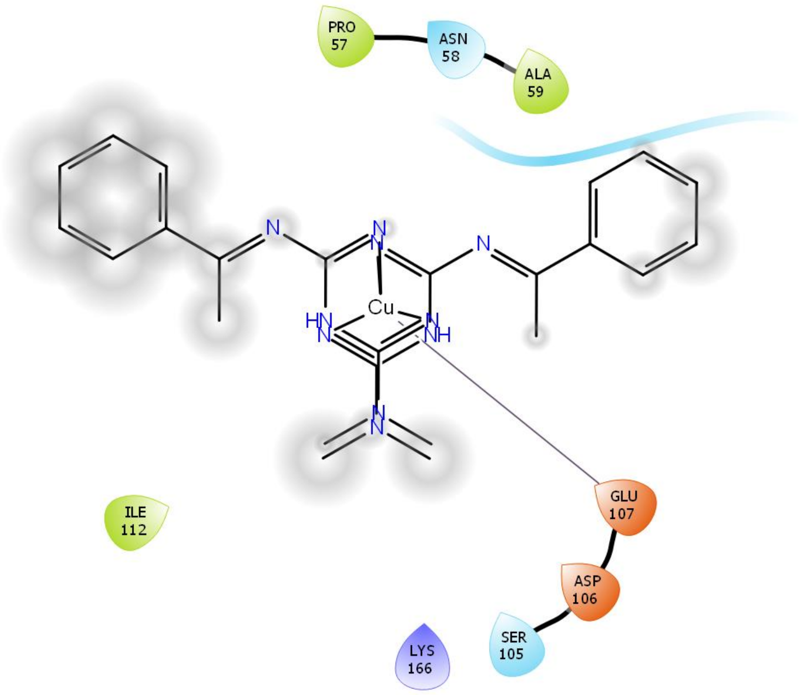PRO 57<br>ASN 58<br>ALA 59<br>Cu<br>ILE 112<br>GLU 107<br>ASP 106<br>SER 105<br>LYS 166
MCu-7	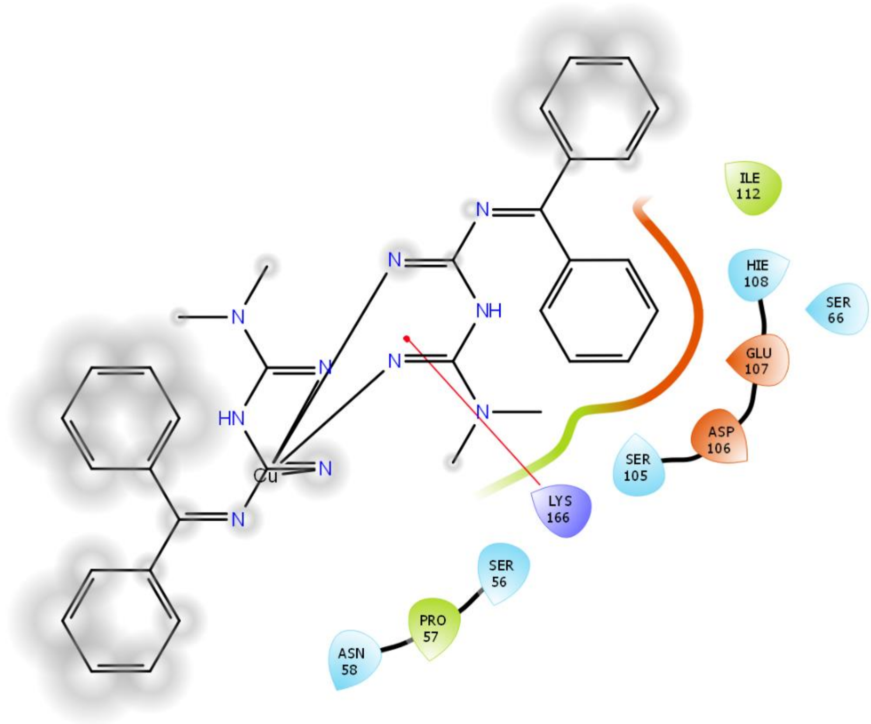ILE 112<br>HIE 108<br>SER 66<br>GLU 107<br>ASP 106<br>SER 105<br>LYS 166<br>SER 56<br>PRO 57<br>ASN 58<br>Cu

MCu-8	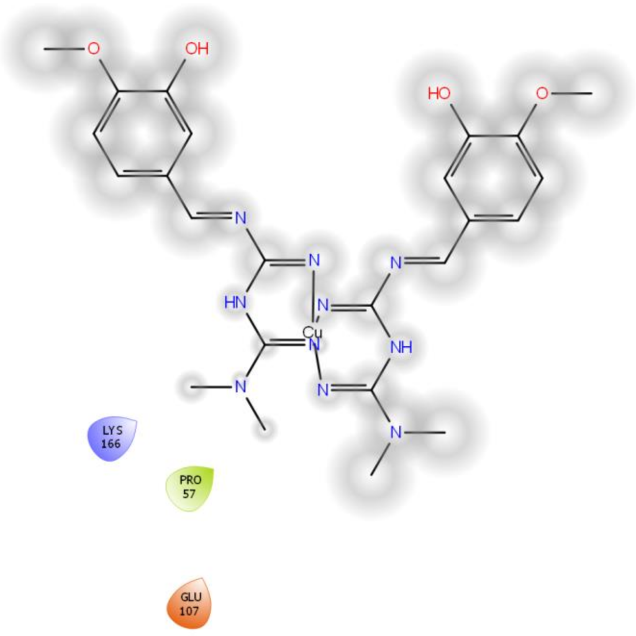OH<br>HO<br>Cu<br>LYS 166<br>PRO 57<br>GLU 107
MCu-9	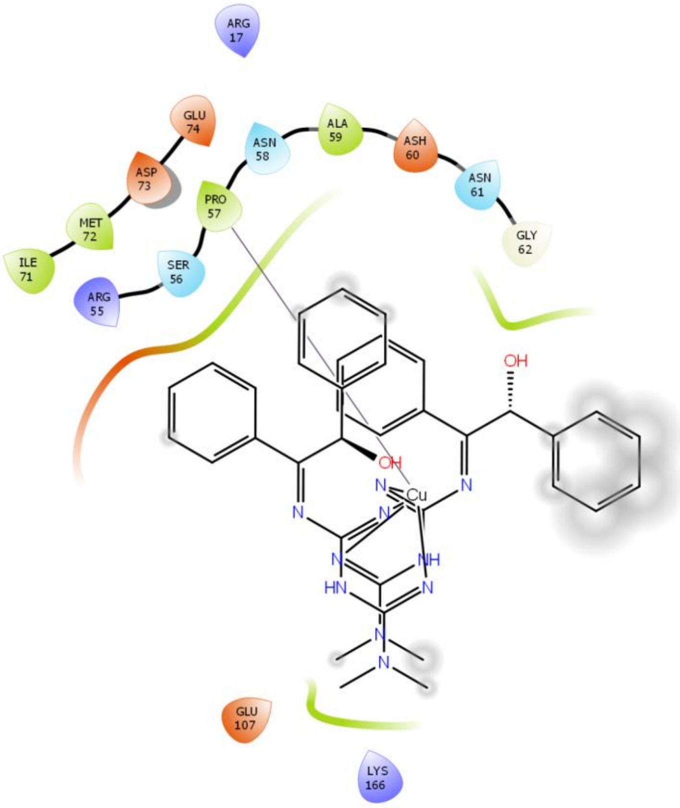ARG 17<br>GLU 74<br>ASP 73<br>MET 72<br>ILE 71<br>ARG 55<br>SER 56<br>PRO 57<br>ASN 58<br>ALA 59<br>ASH 60<br>ASN 61<br>GLY 62<br>OH<br>Cu<br>GLU 107<br>LYS 166

Table S4. 3D pose and geometries of all the metformin derivatives.

**Derivative code**	**3D Pose**
MET-1	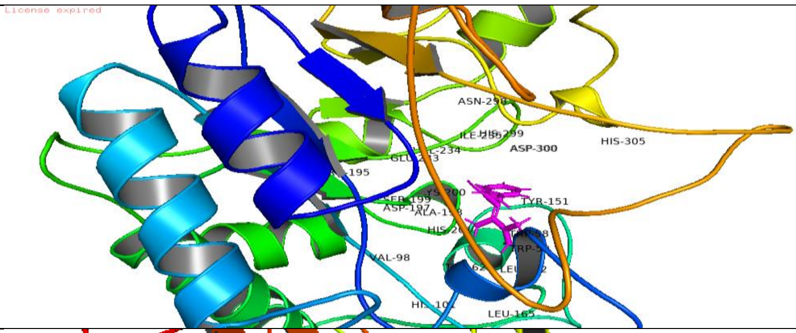
MET-2	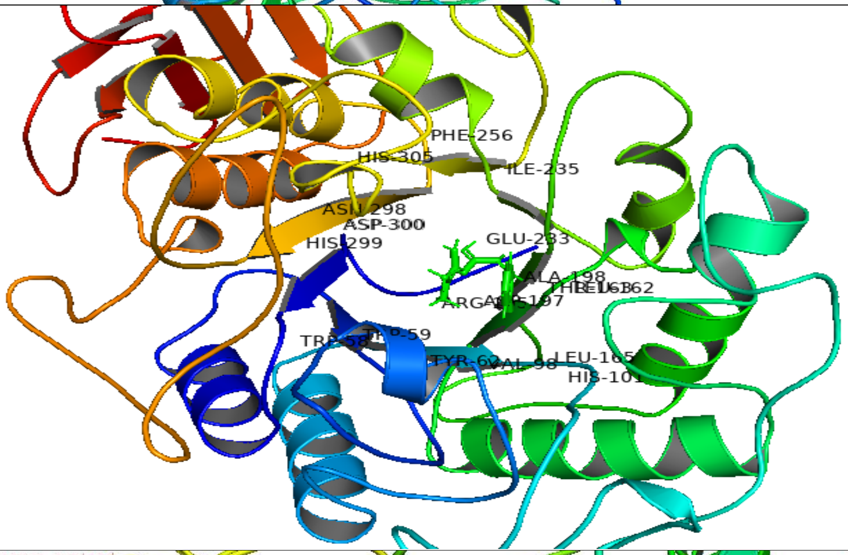
MET-3	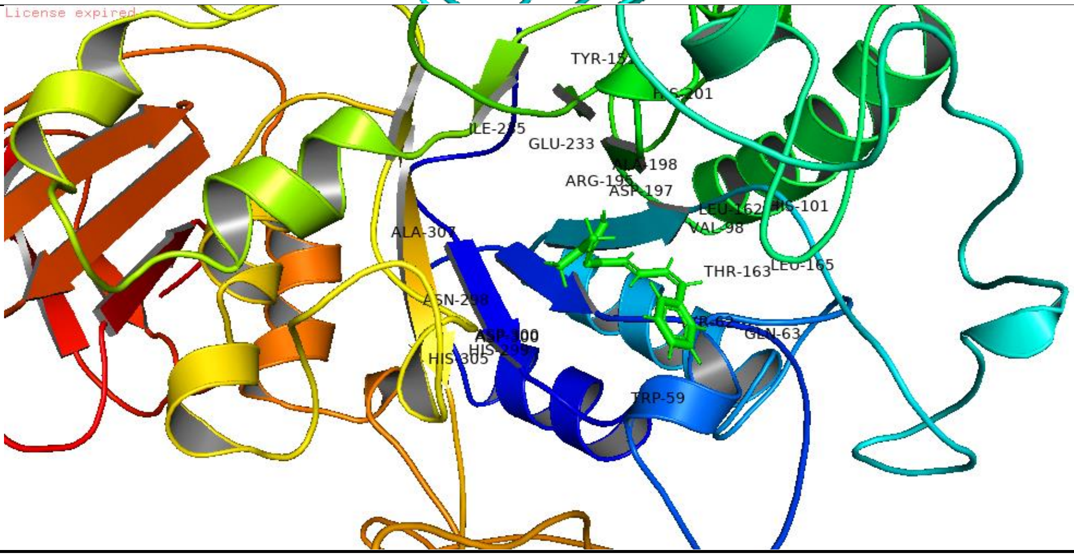

MET-4	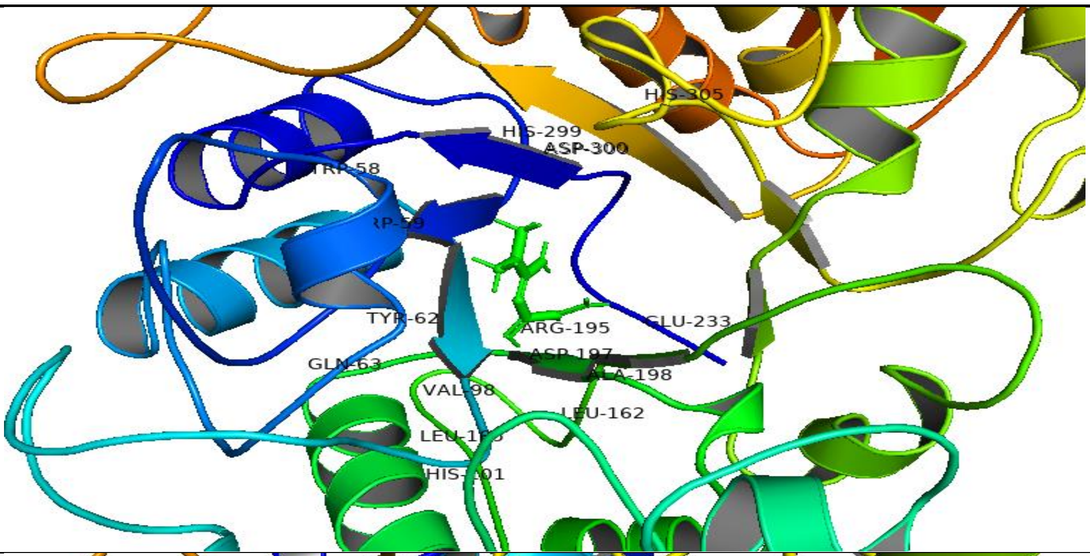
MET-5	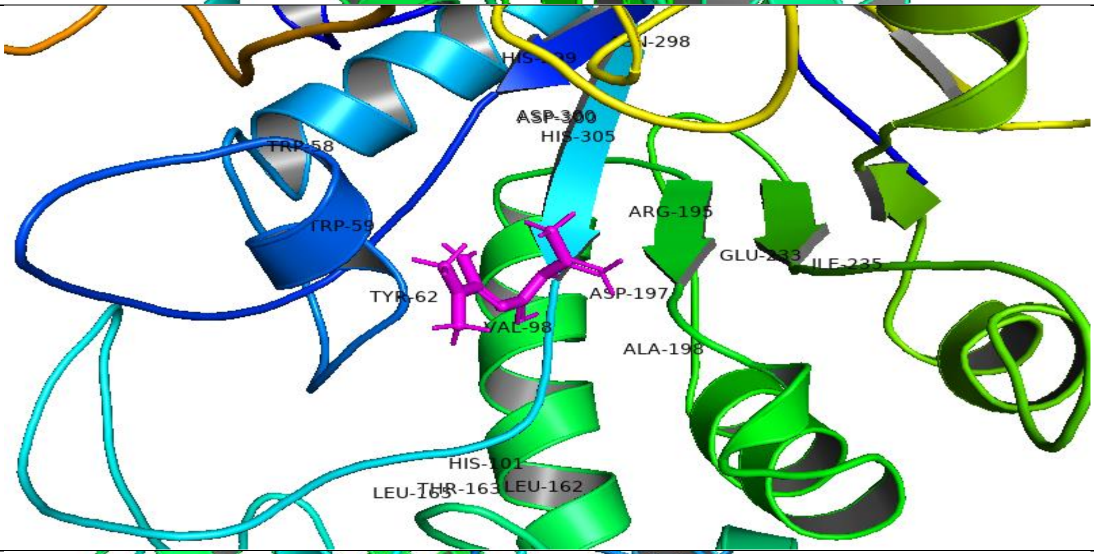
MET-6	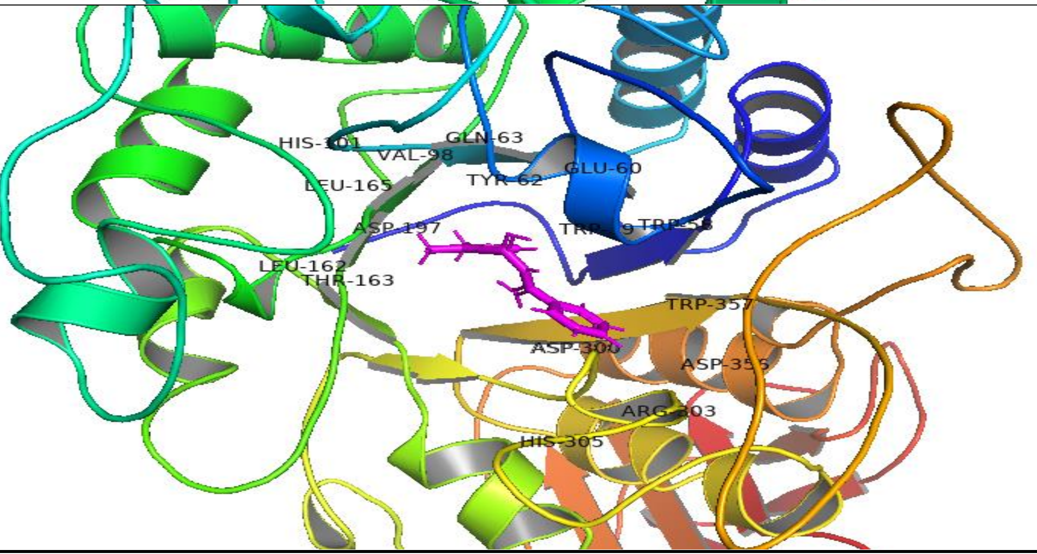

MET-7

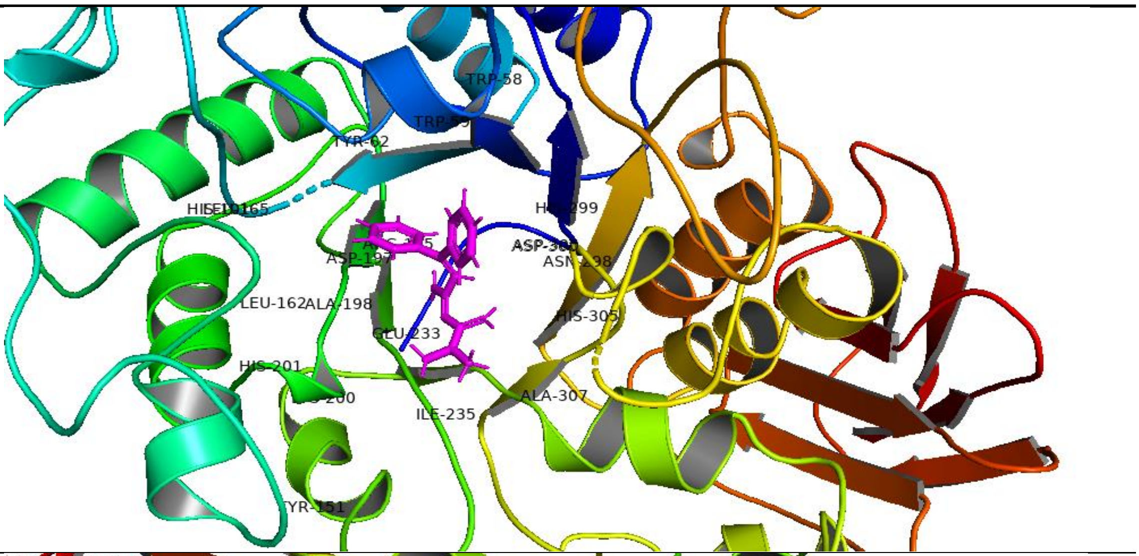

MET-8

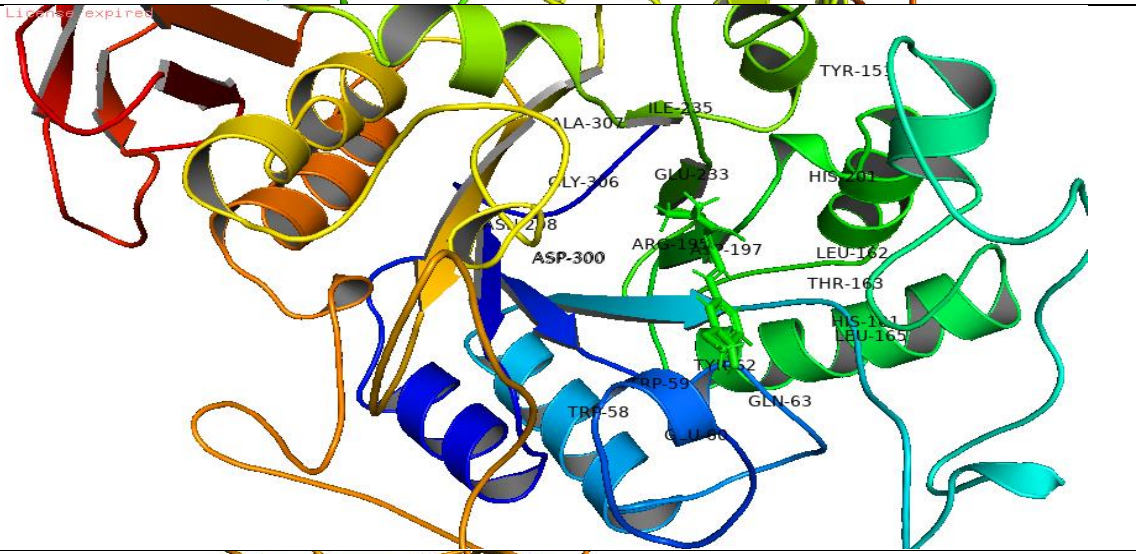

MET-9

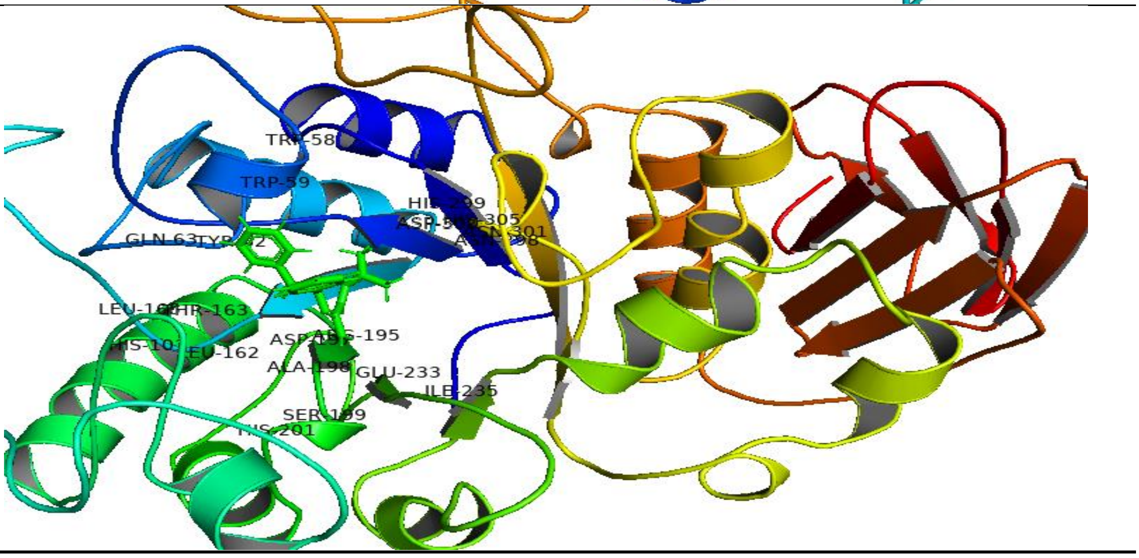

<table>
<tr><td>MET</td><td>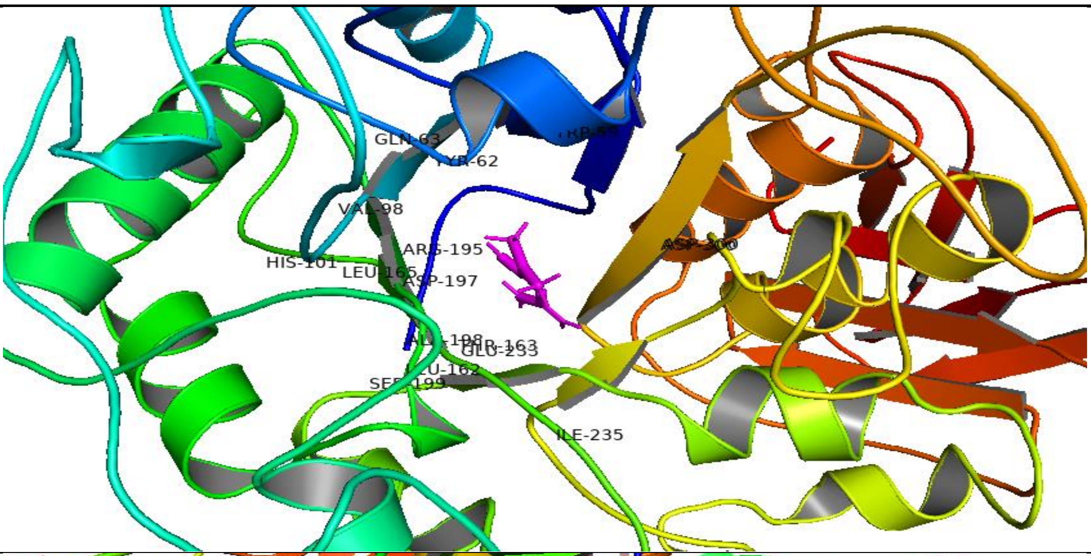
</td></tr>
<tr><td>MZN-1</td><td>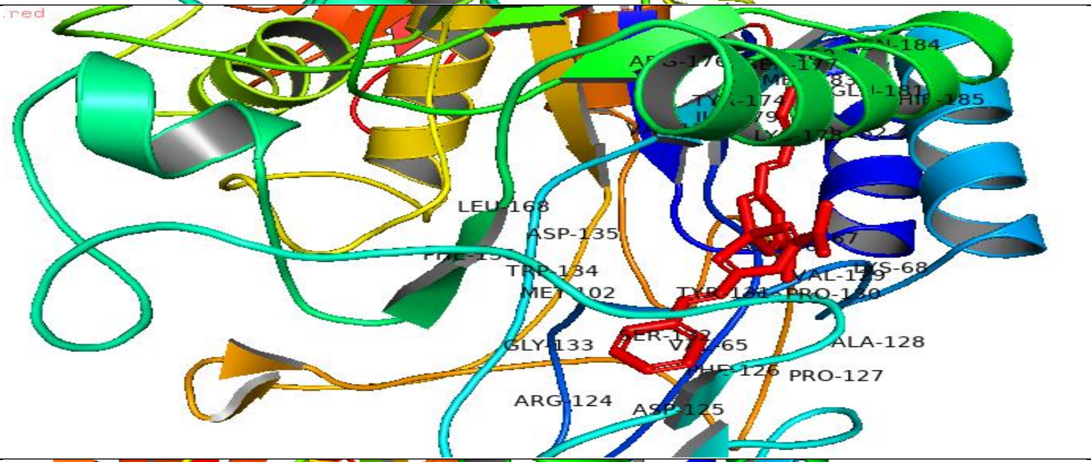
</td></tr>
<tr><td>MZN-2</td><td>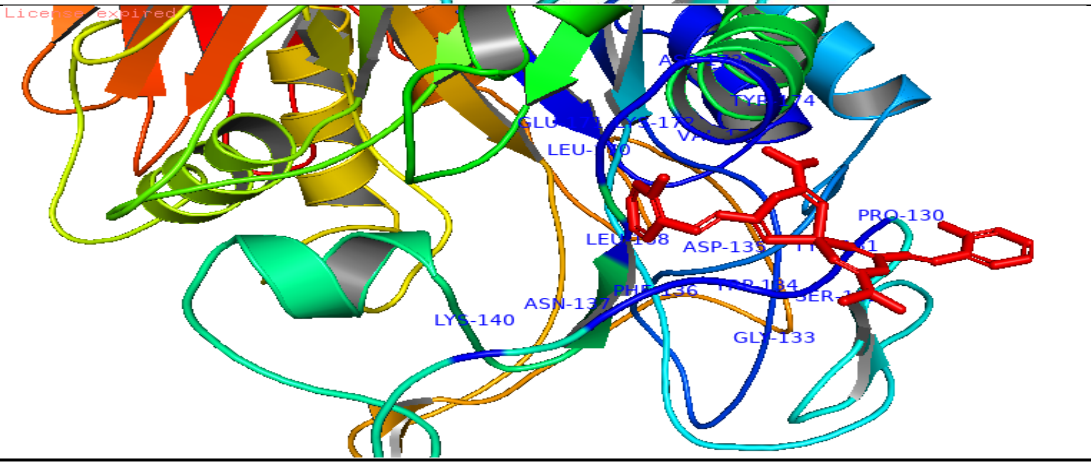
</td></tr>
</table>

MZN-3

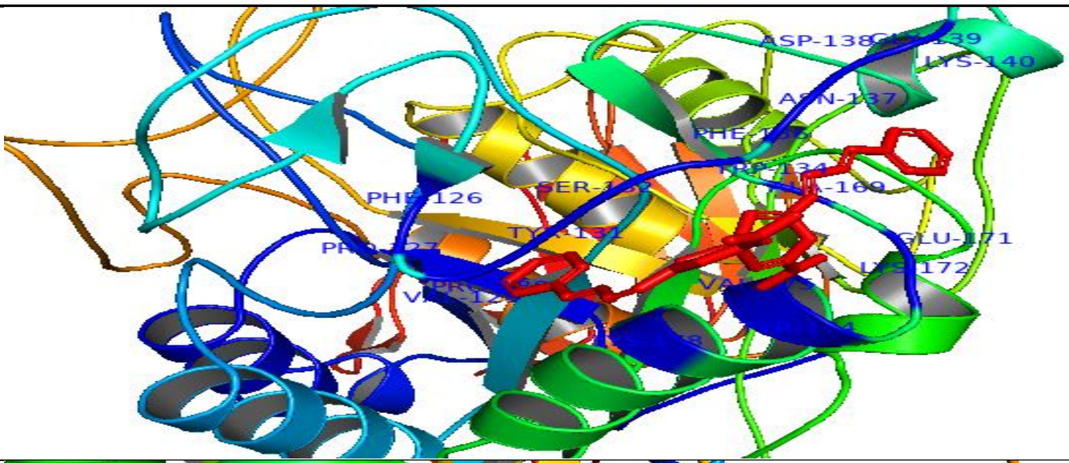

MZN-4

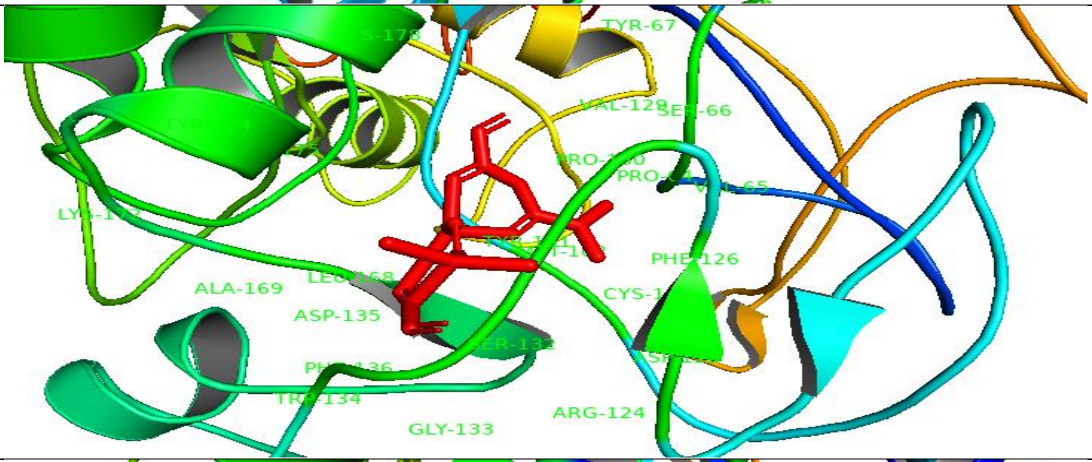

MZN-5

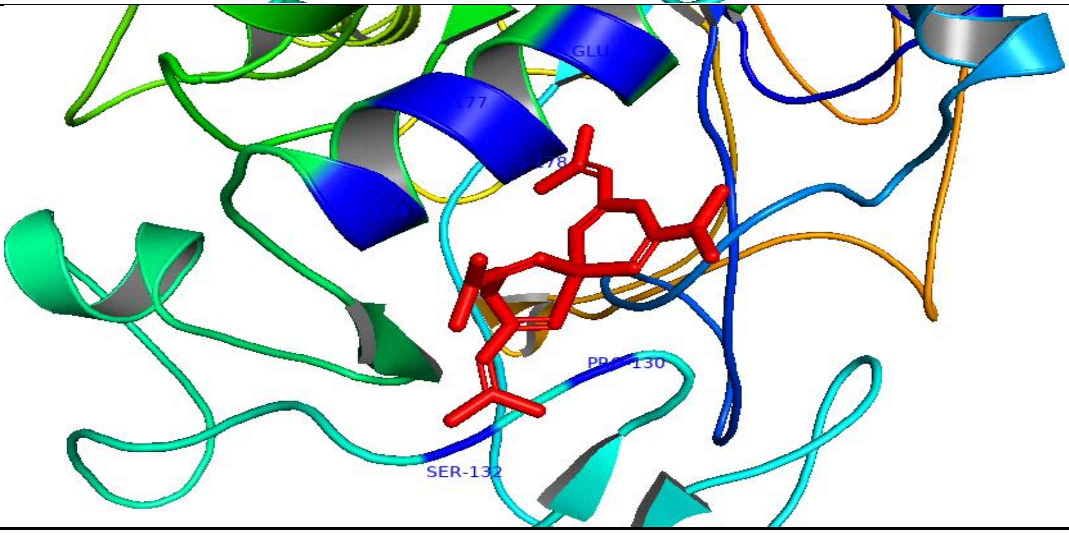

MZN-6	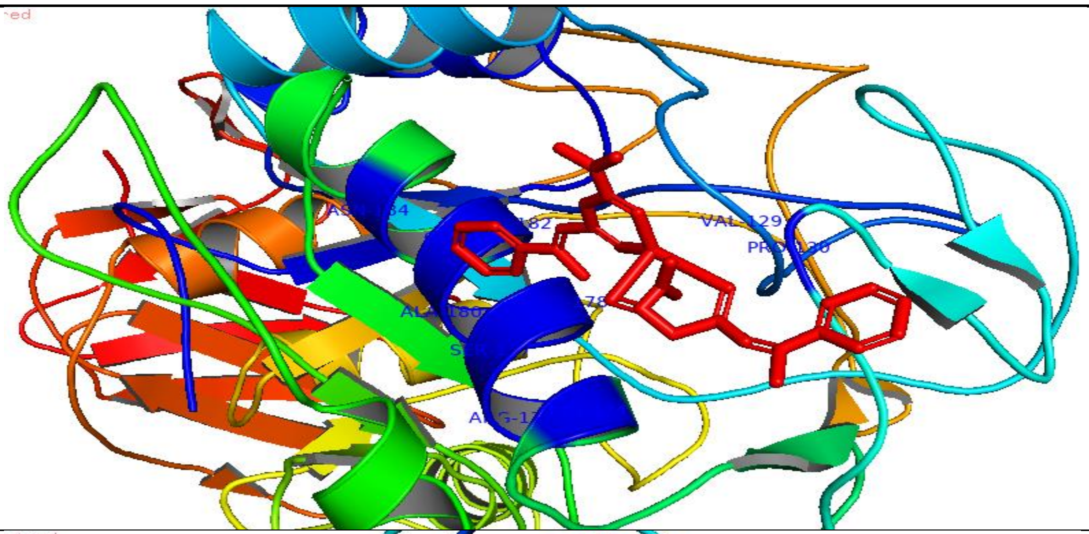
MZN-7	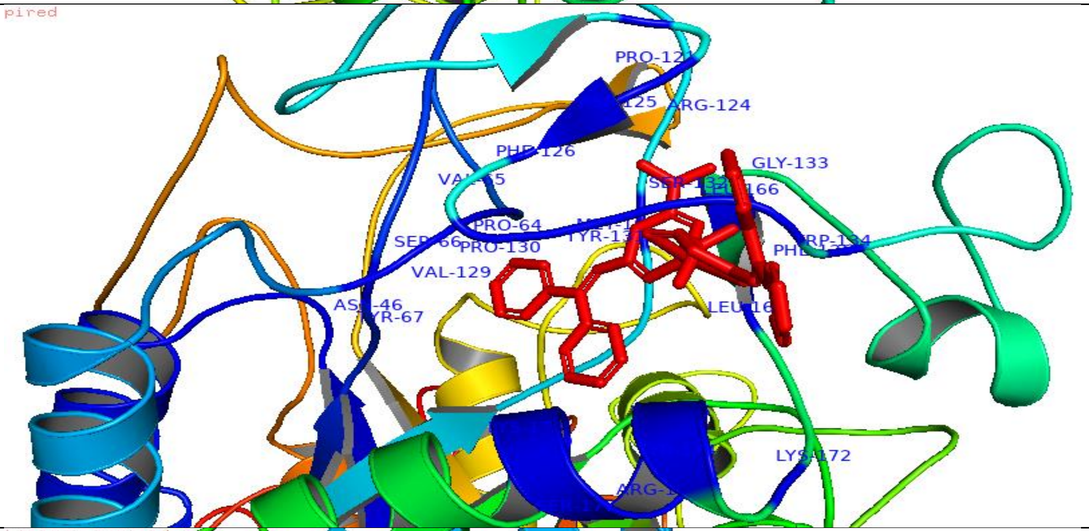
MZN-8	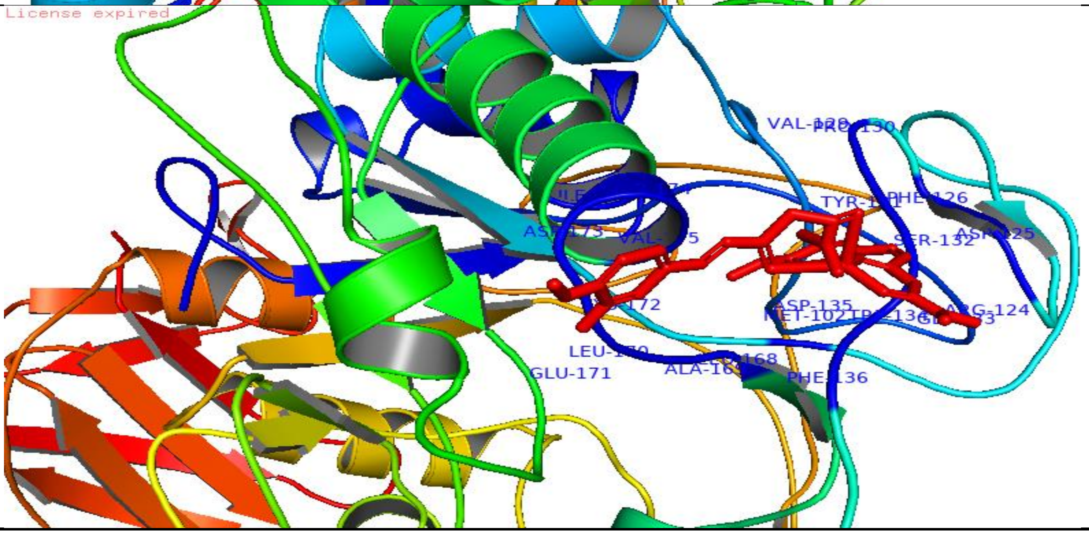

MZN-9

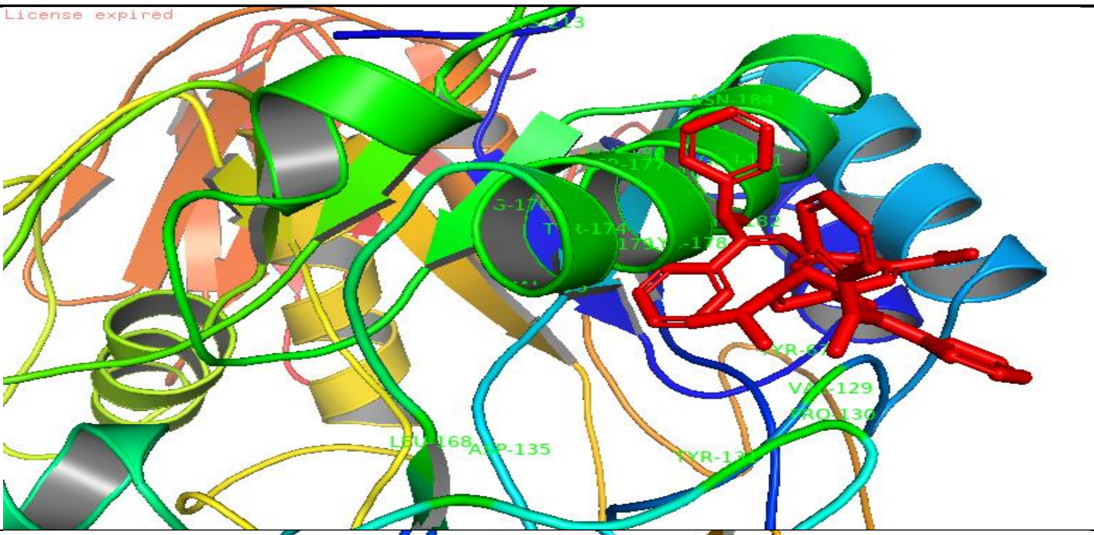

MCu-1

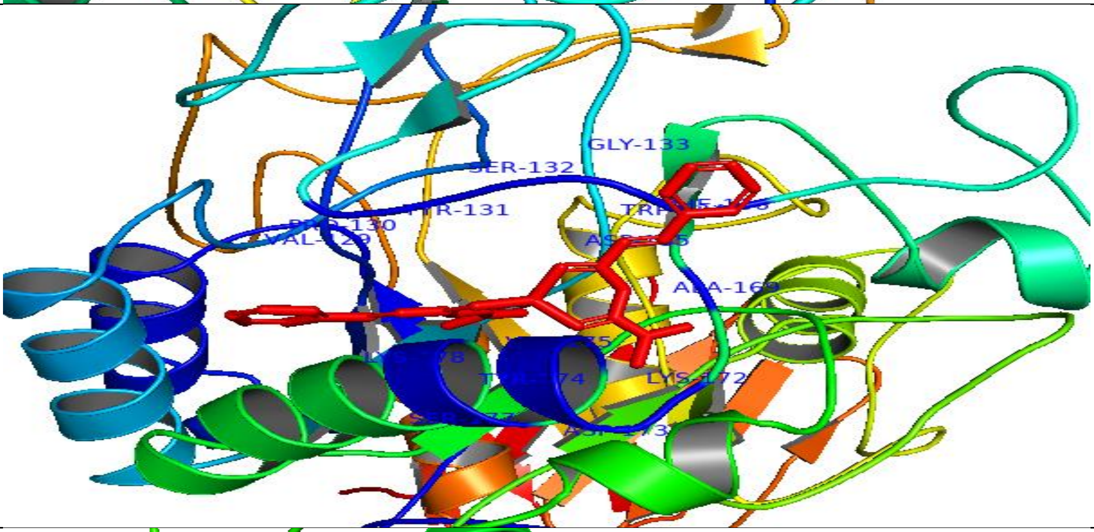

MCu-2

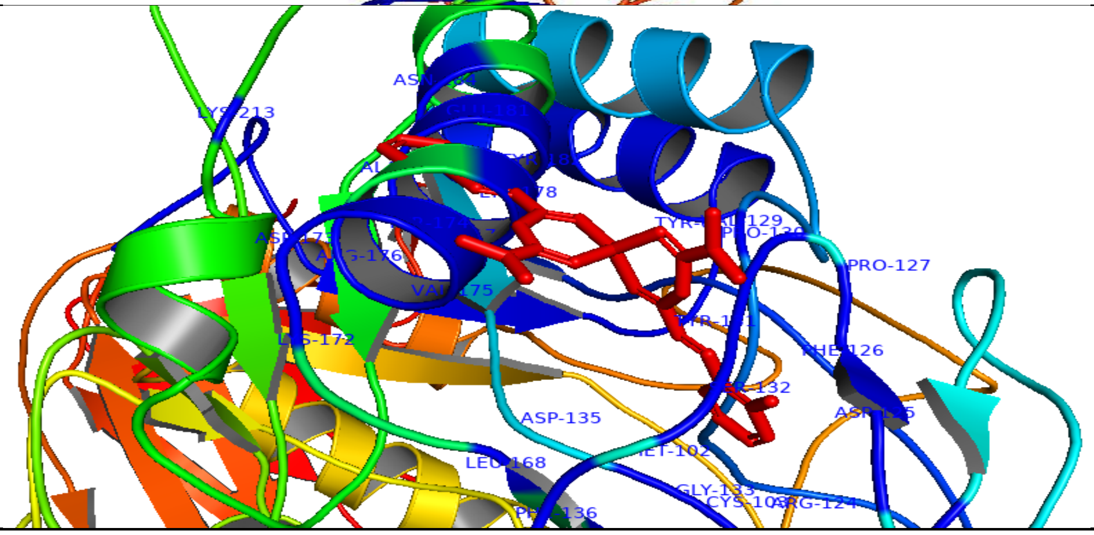

MCu-3	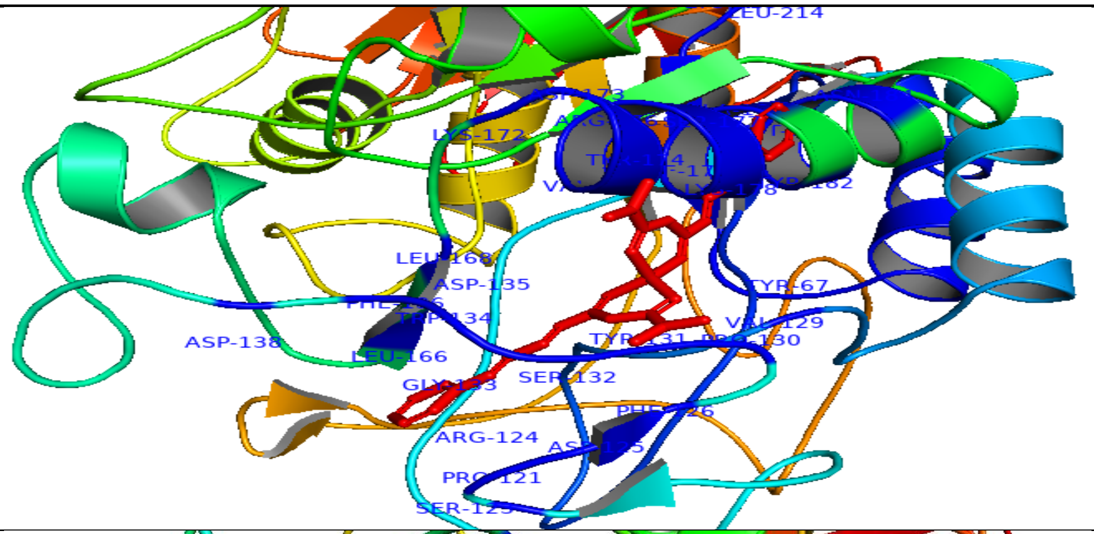
MCu-4	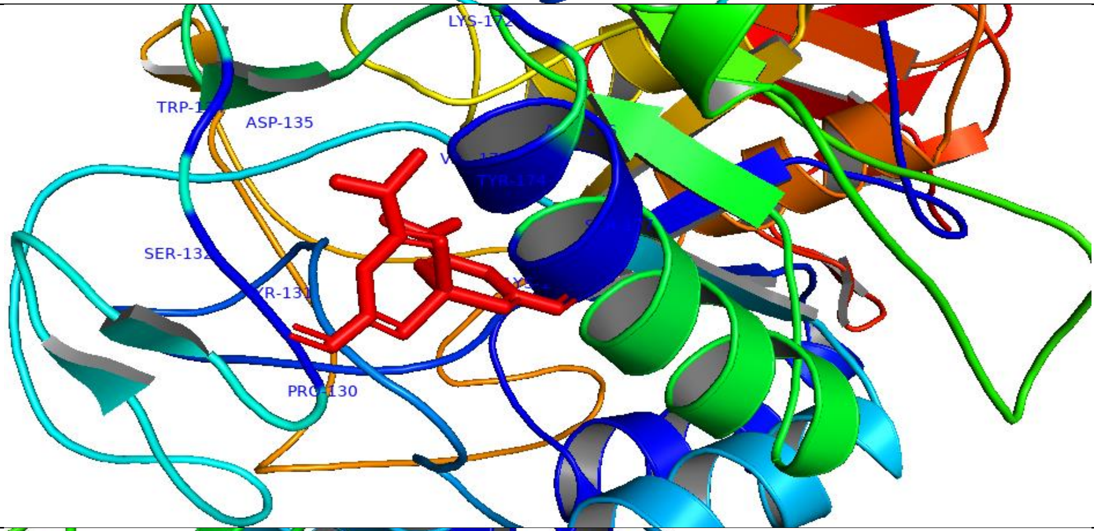
MCu-5	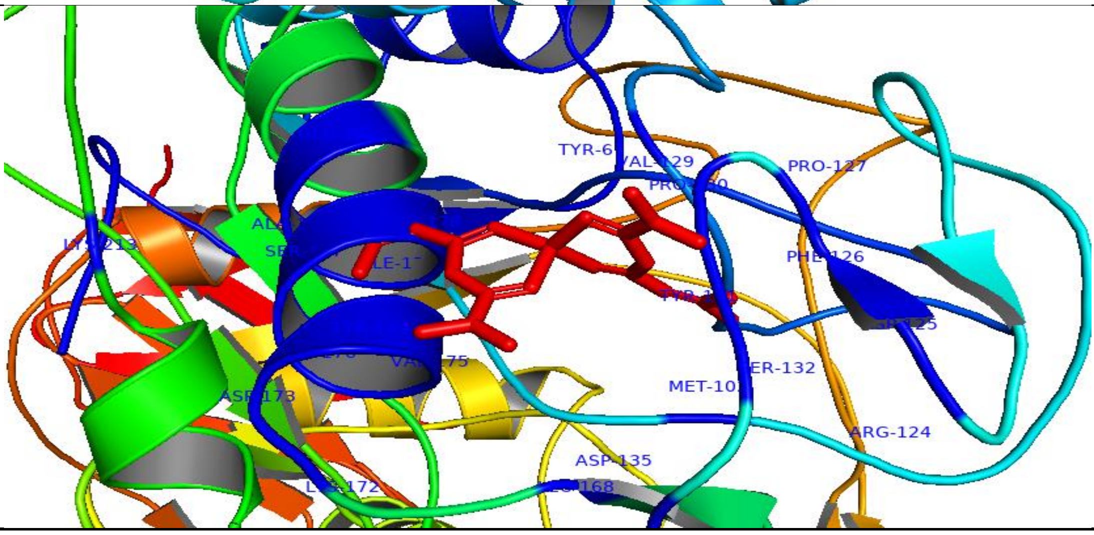

MCu-6	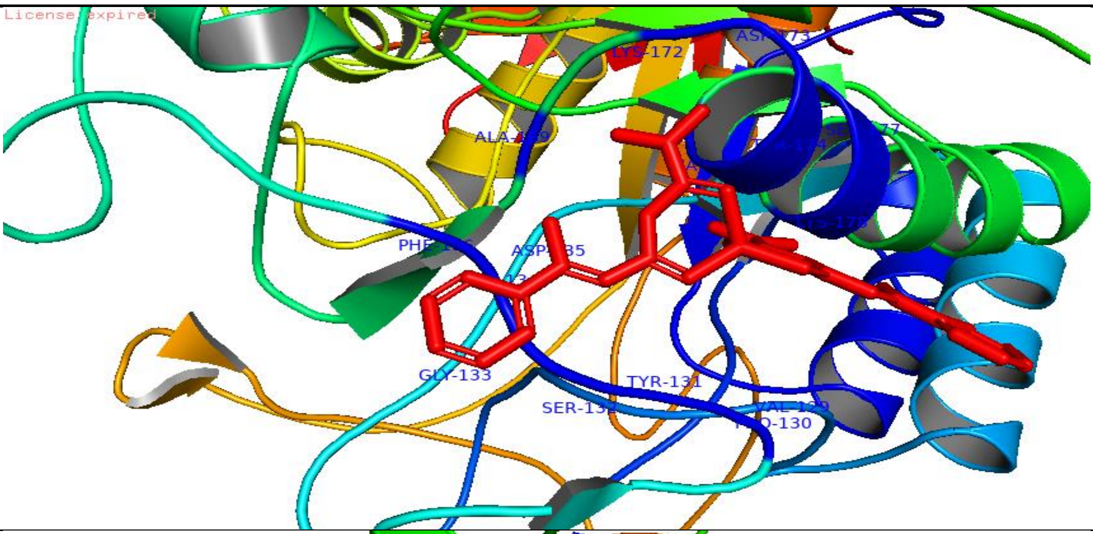
MCu-7	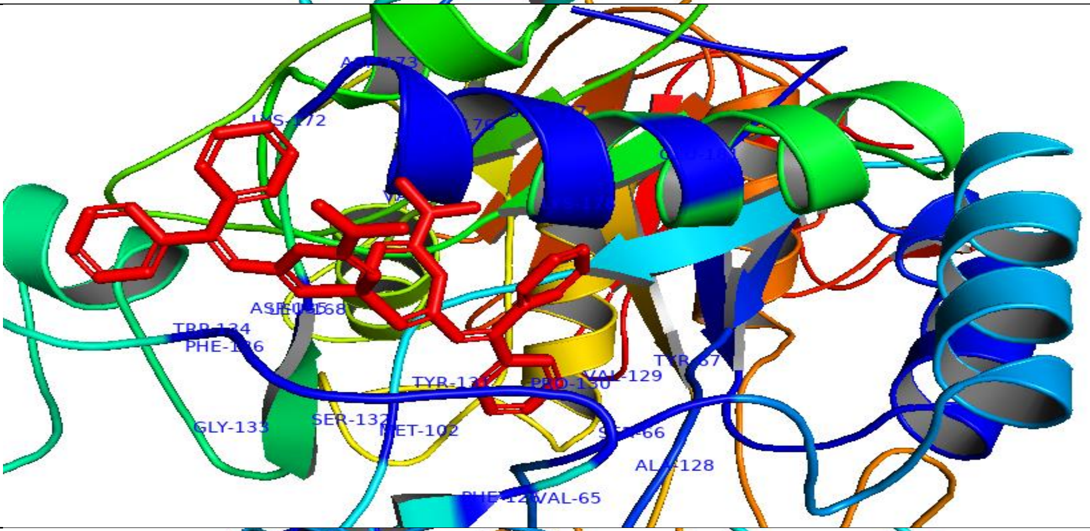
MCu-8	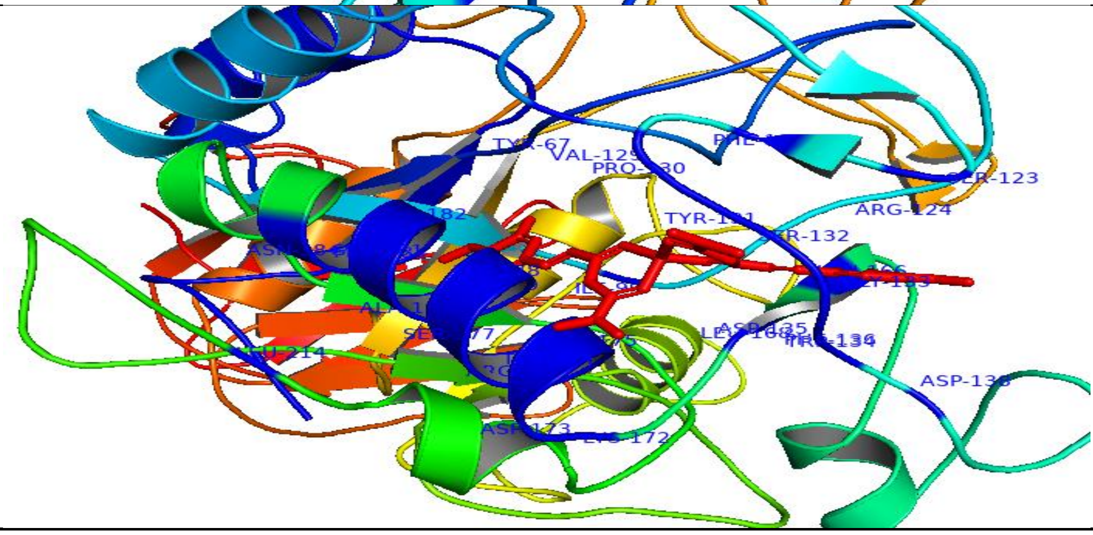

MCu-9	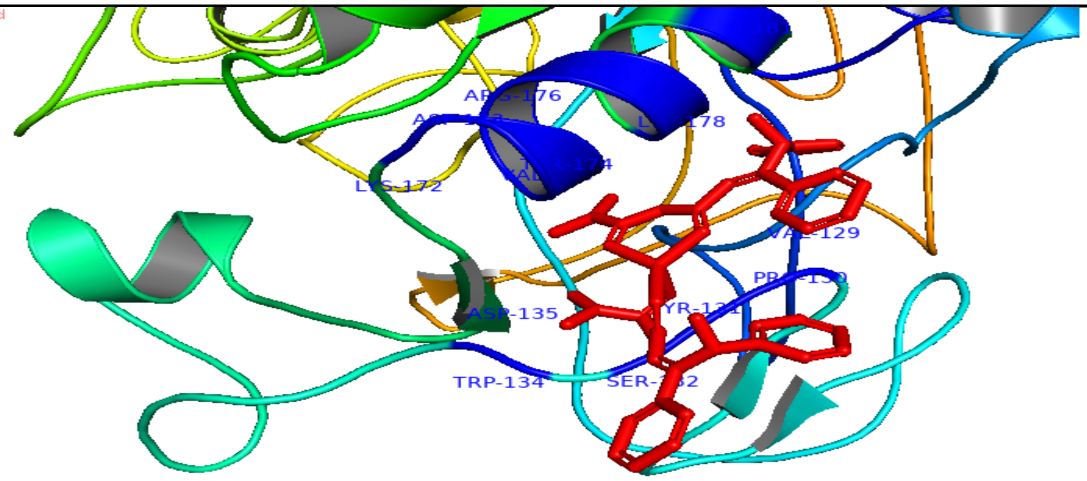

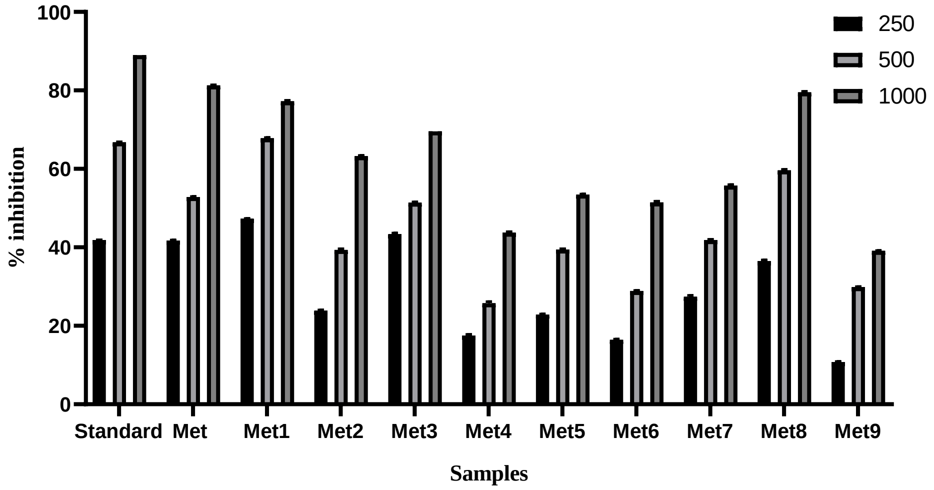

Figure S1. Graphical representation of α-glucosidase activity of Met-1-Met-9.

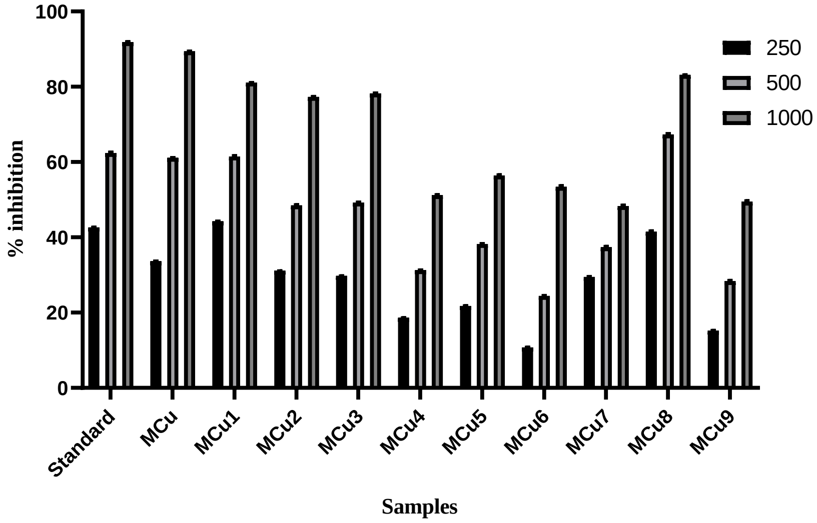

Figure S2. Graphical representation of α-glucosidase activity of MCu1-MCu9.

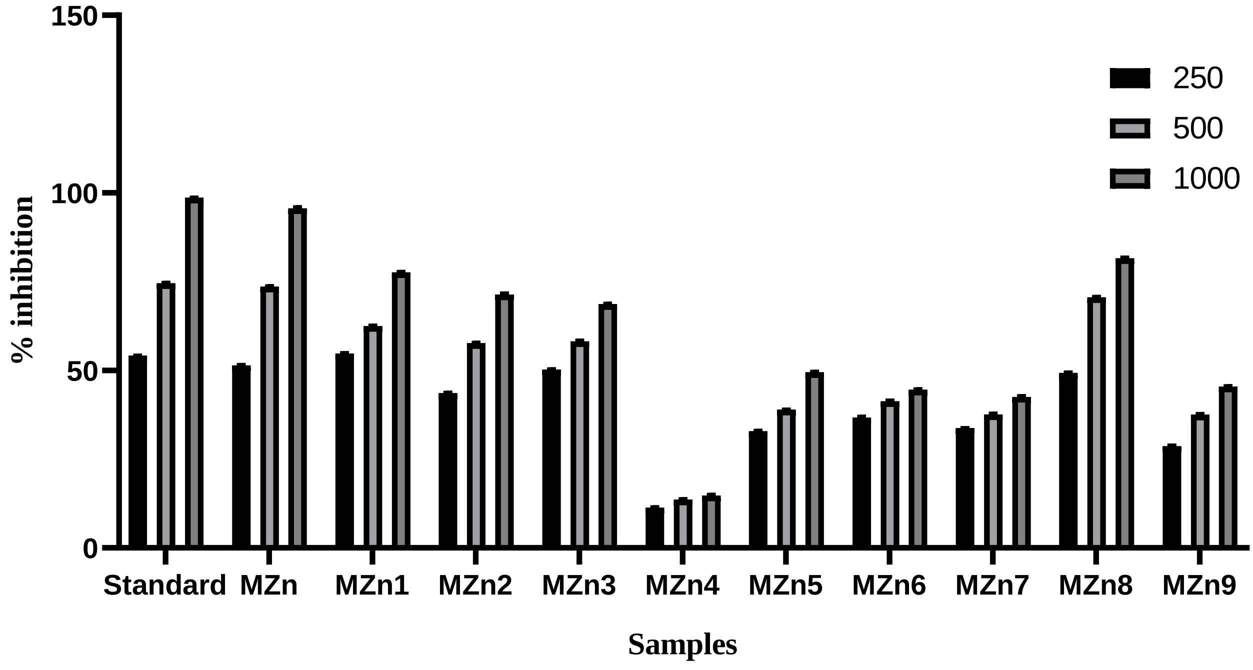

Figure S3. Graphical representation of α-glucosidase activity of MZn1-MZn9.

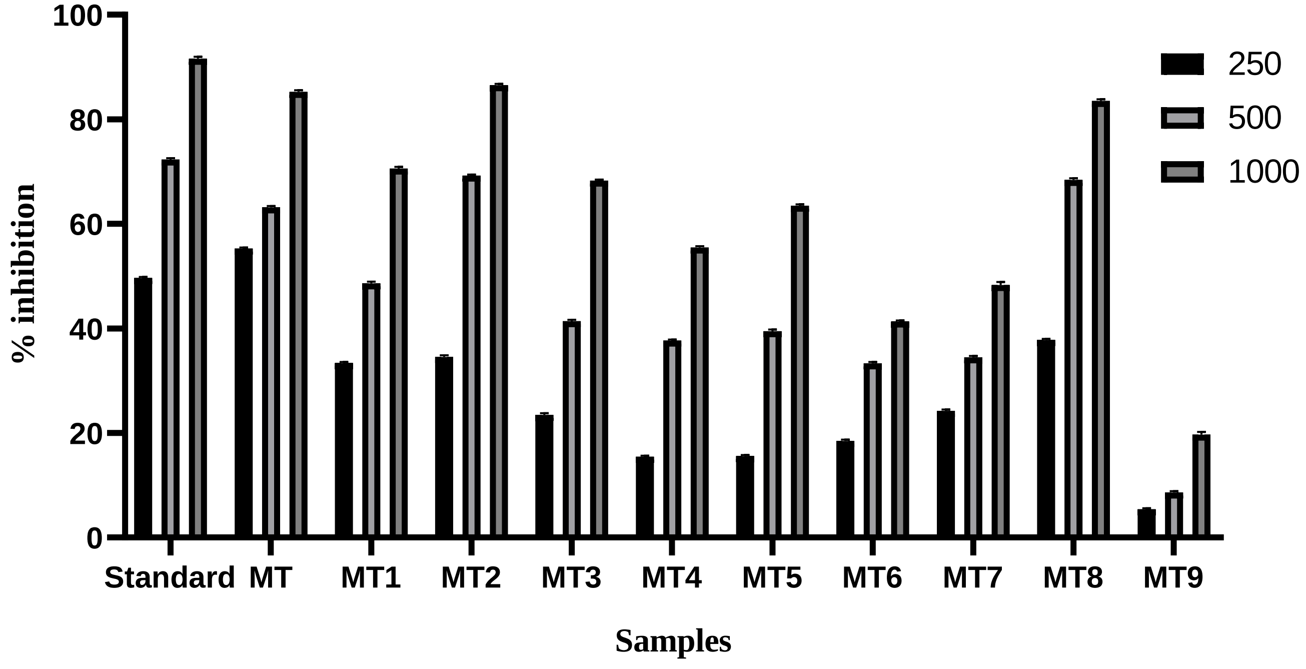

Figure S4. Graphical representation of α-amylase activity of Met1-Met9.

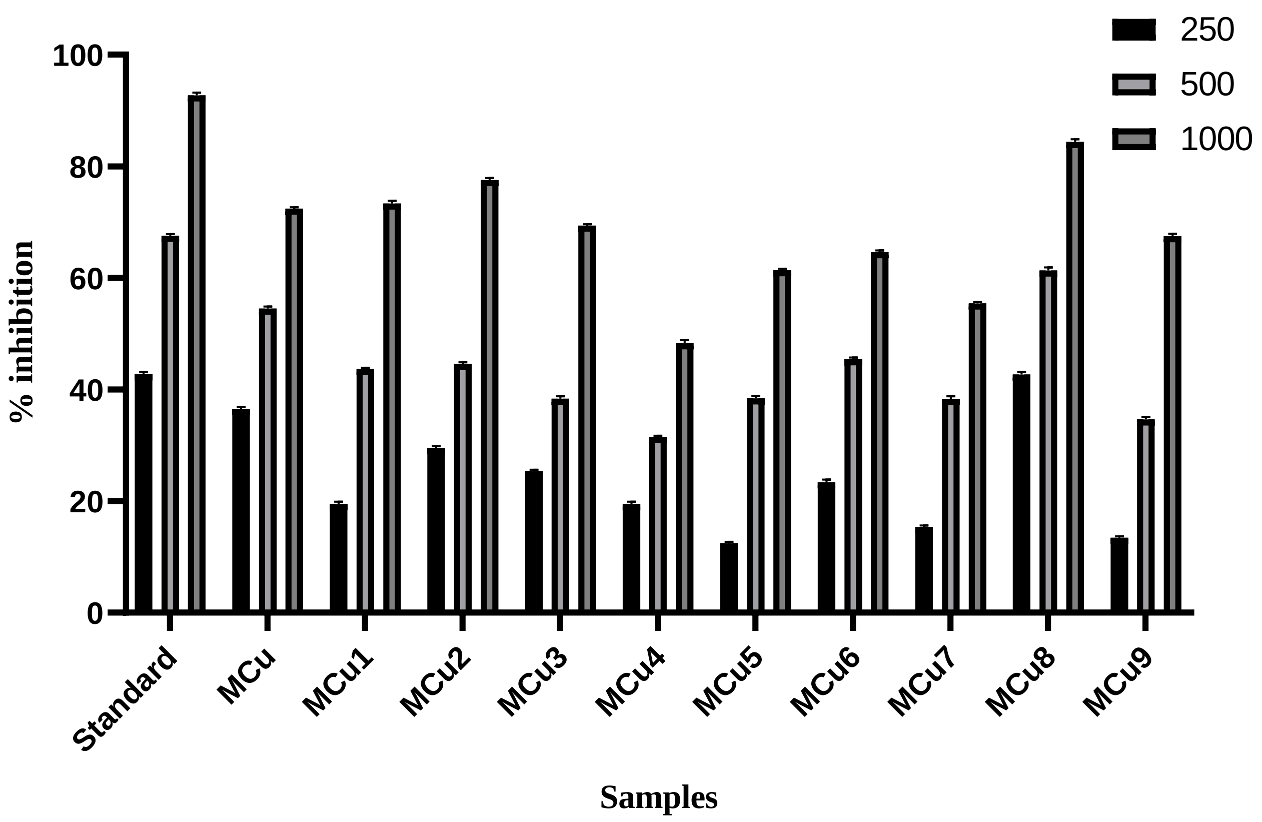

Figure S5. Graphical representation of α-amylase activity of MCu1-MCu9.

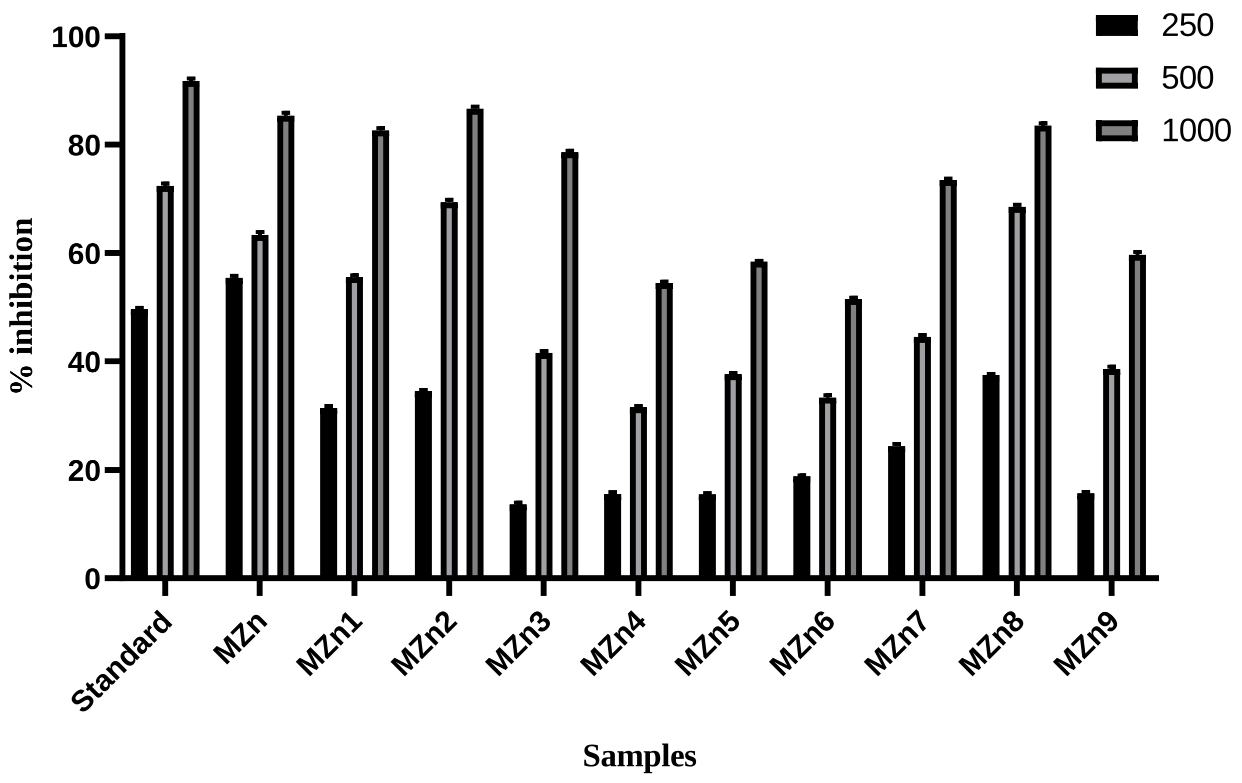

Figure S6. Graphical representation of α-amylase activity of MZn1-MZn9.

NMR Spectra of Metformin Derivatives:

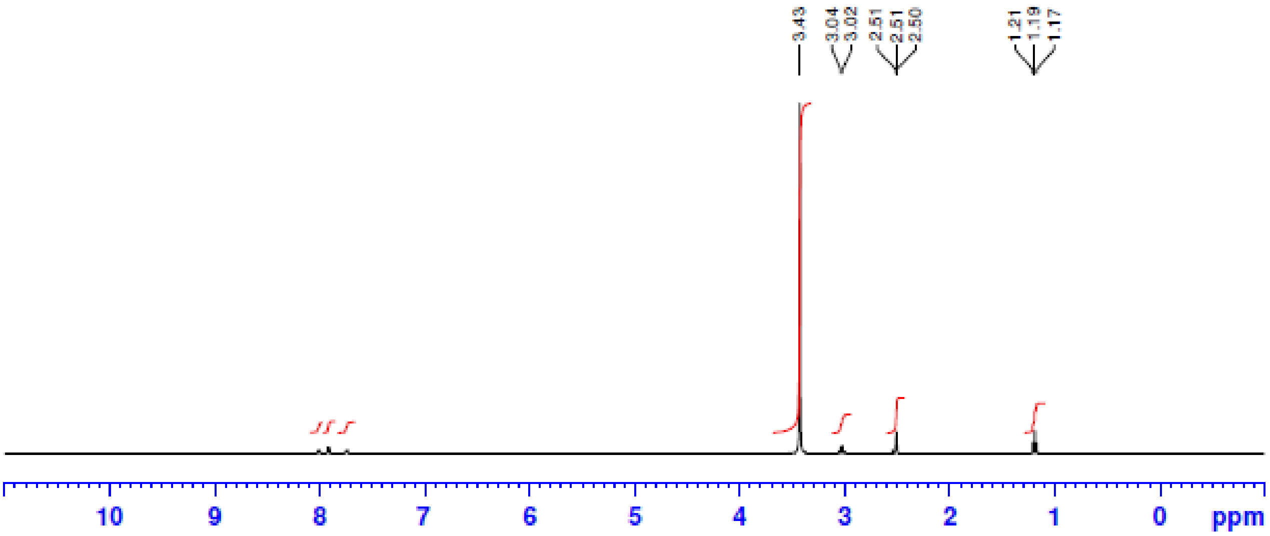

Figure S7. H^1 spectra of Metformin.

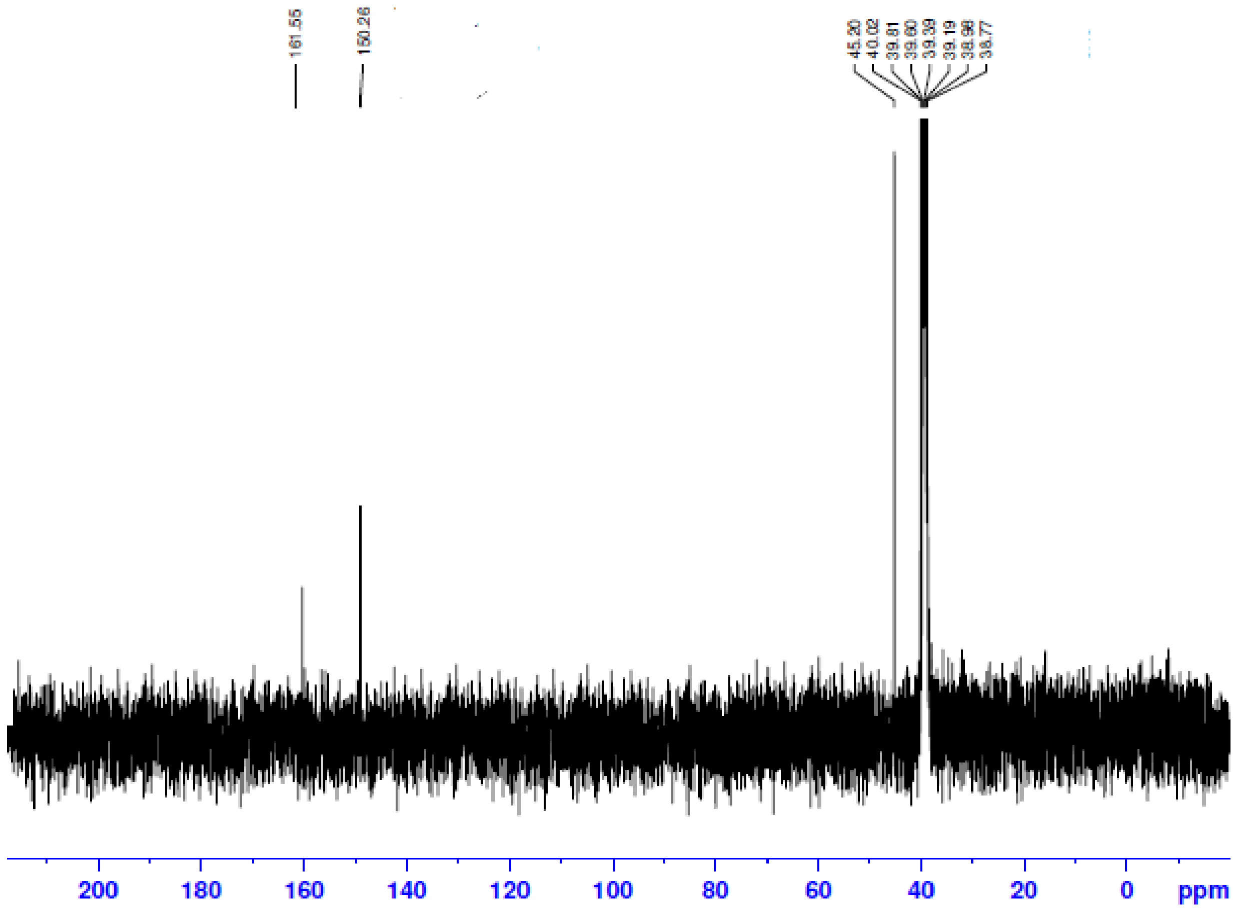

Figure S8. C^{13} spectra of Metformin.

Met1;

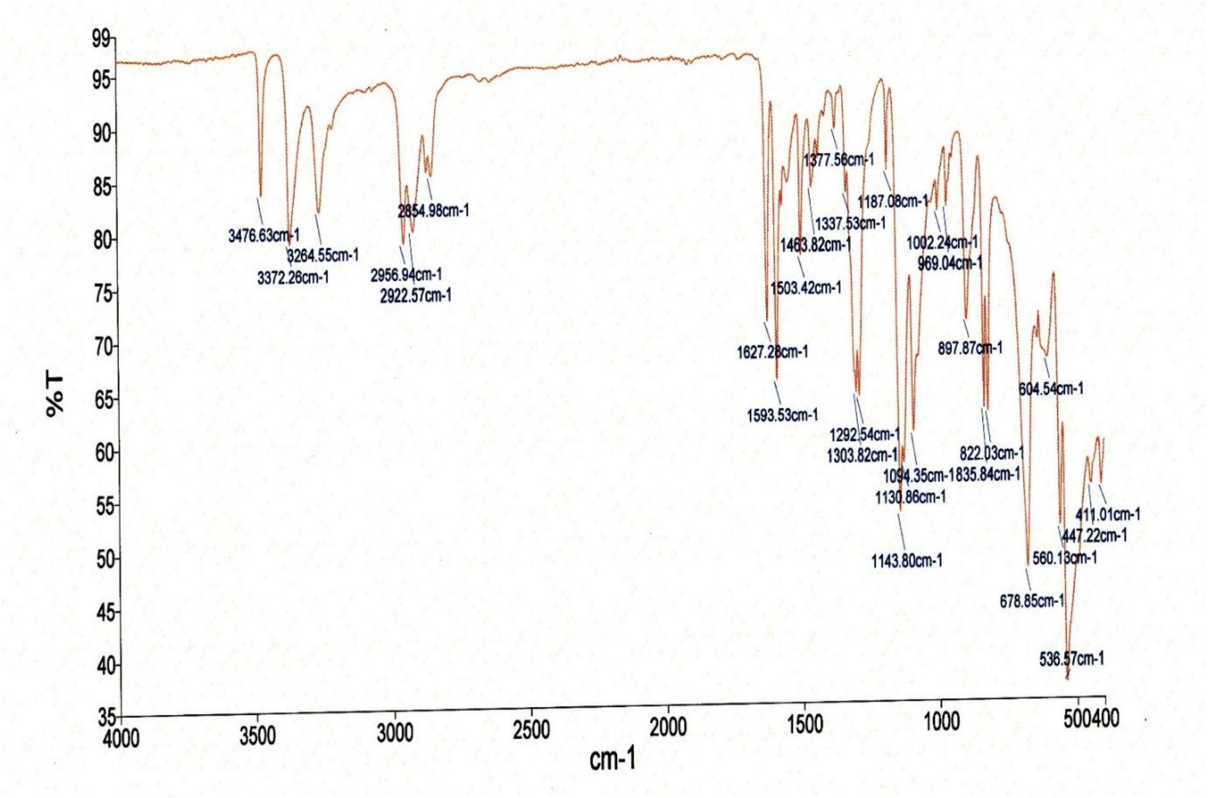

Figure S9. IR spectra of Metformin derivative Met1.

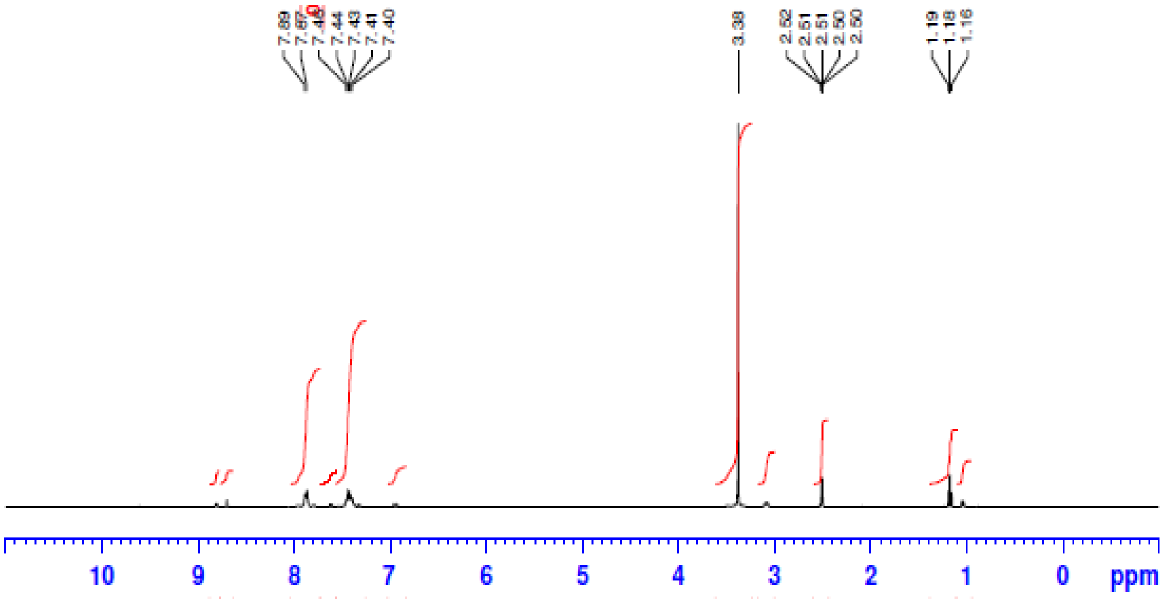

Figure S10. H[1] spectra of Metformin derivative Met1.

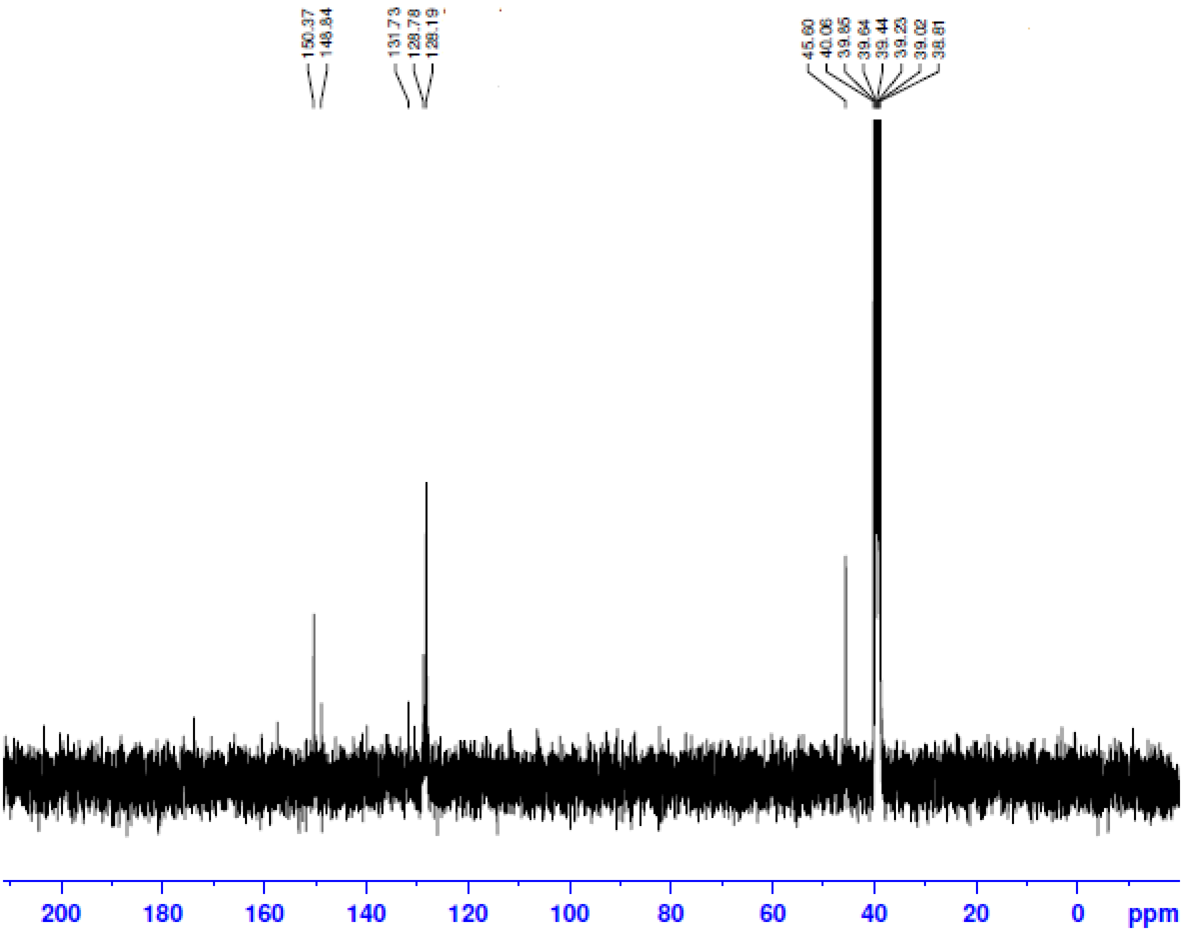

Figure S11. C[13] spectra of Metformin derivative Met1.

Met 2;

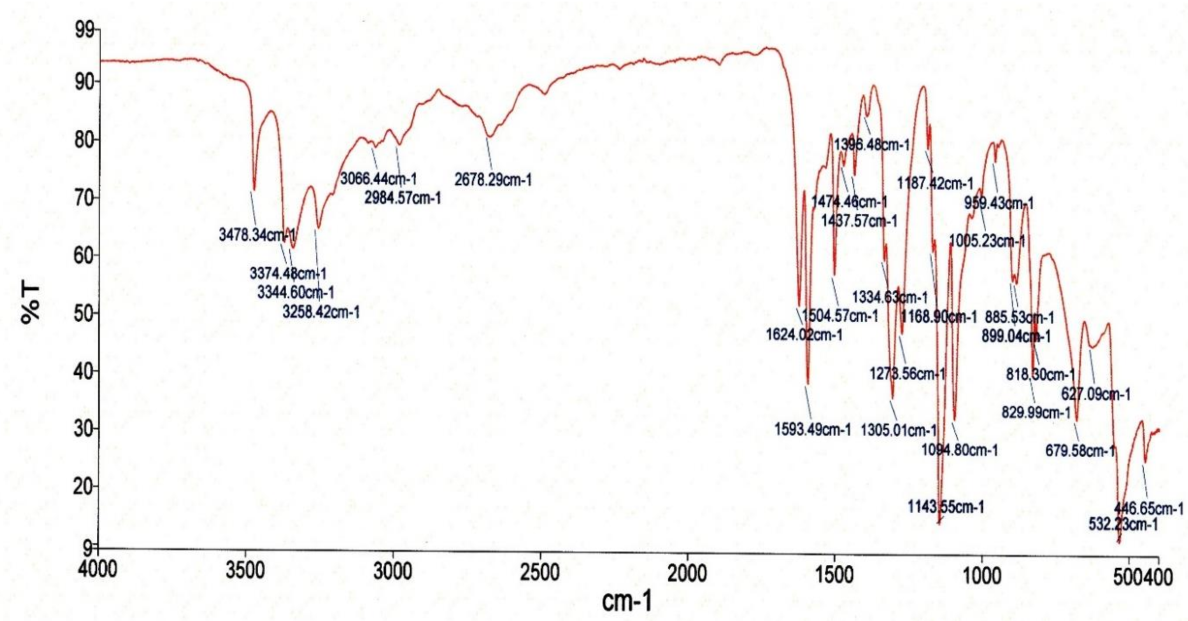

Figure S12. IR spectra of Metformin derivative Met2.

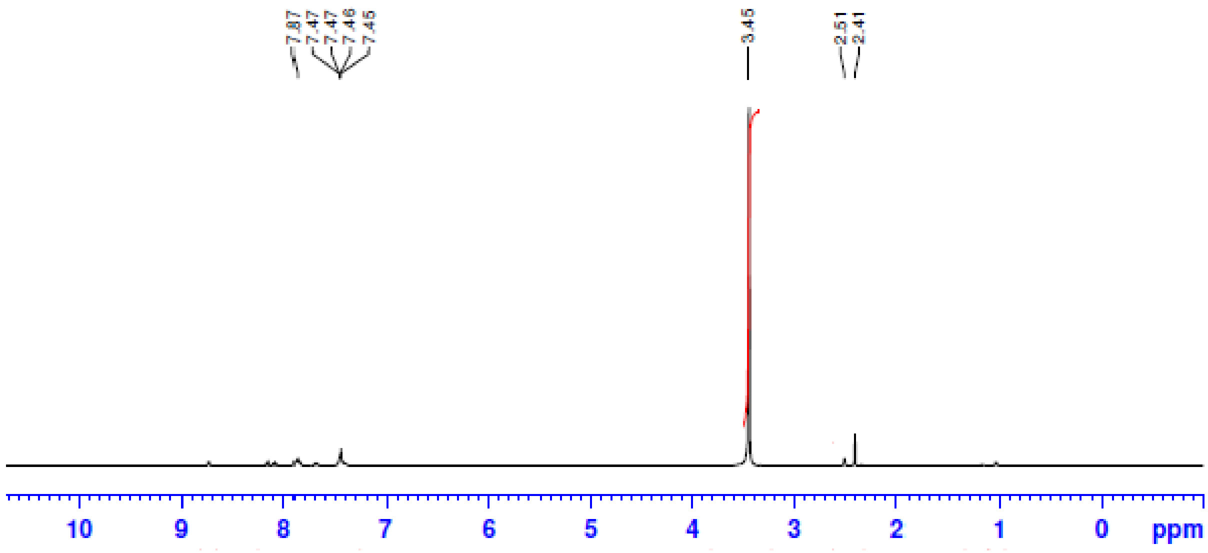

Figure S13. H[1] spectra of Metformin derivative Met2.

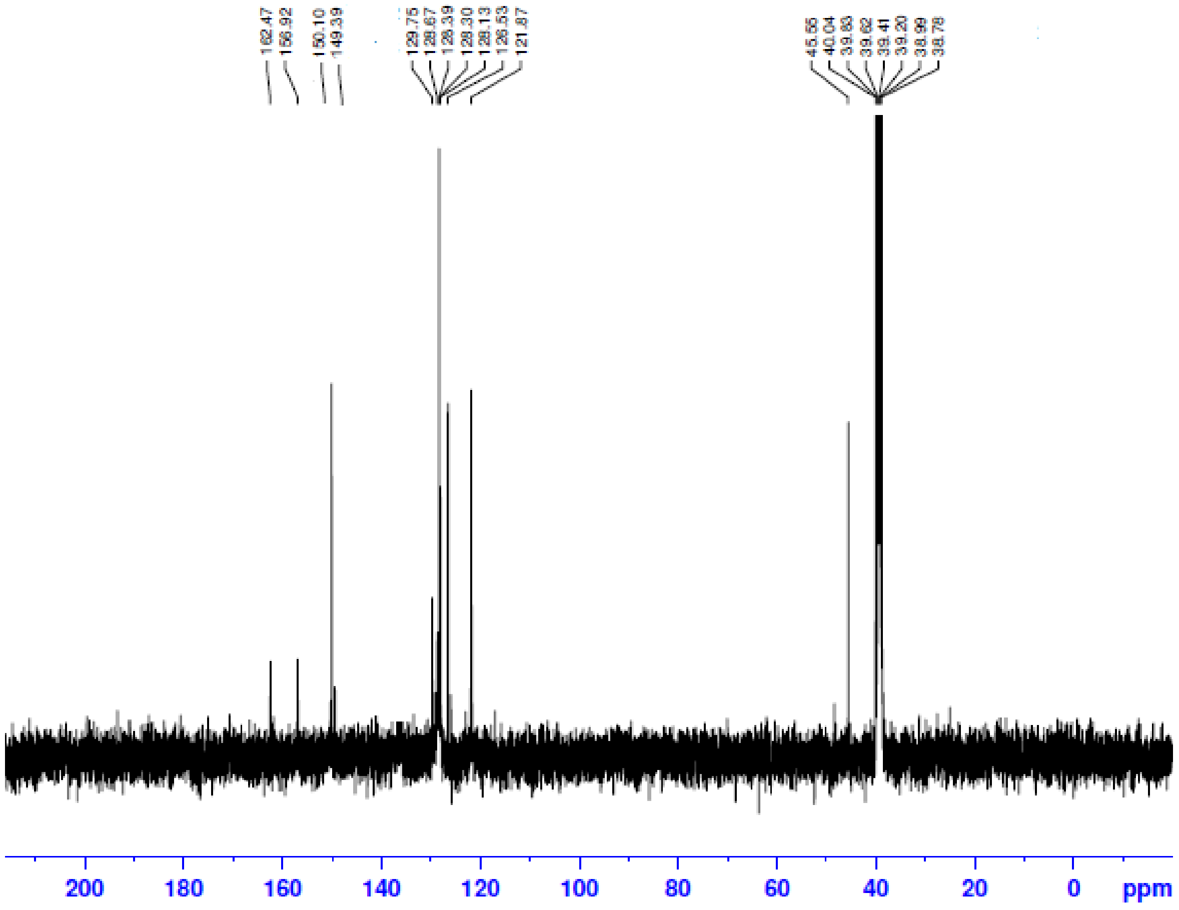

Figure S14. C[13] spectra of Metformin derivative Met2.

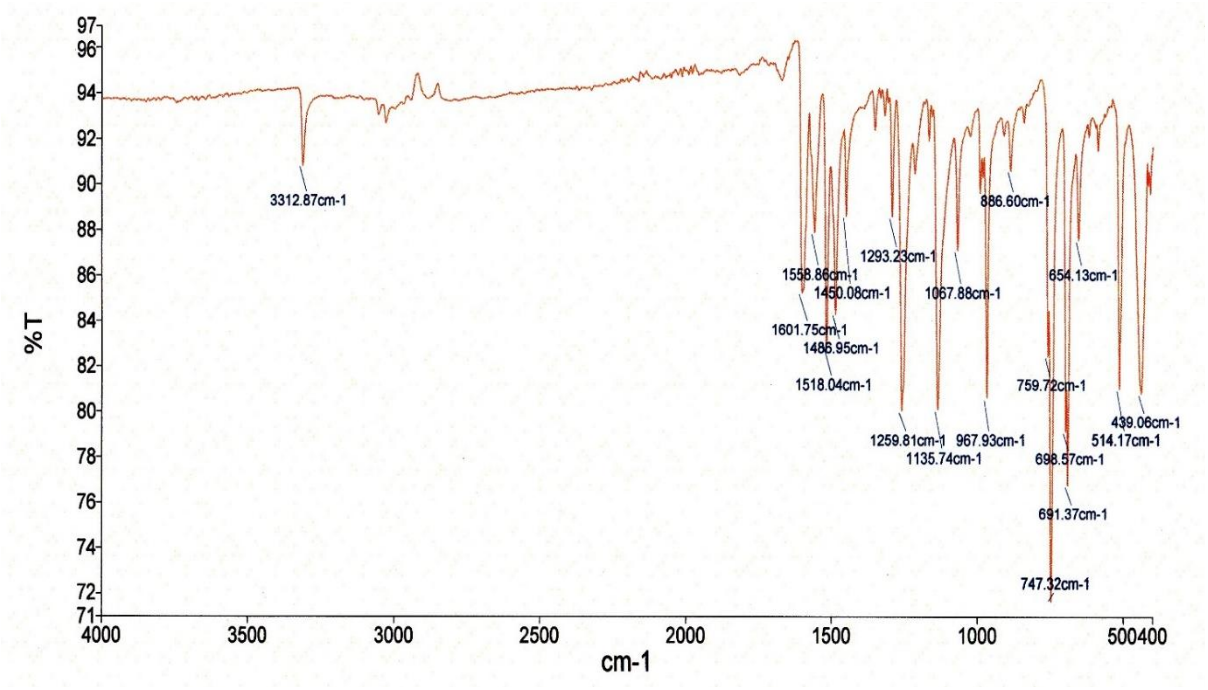

Figure S15. IR spectra of Metformin derivative Met3.

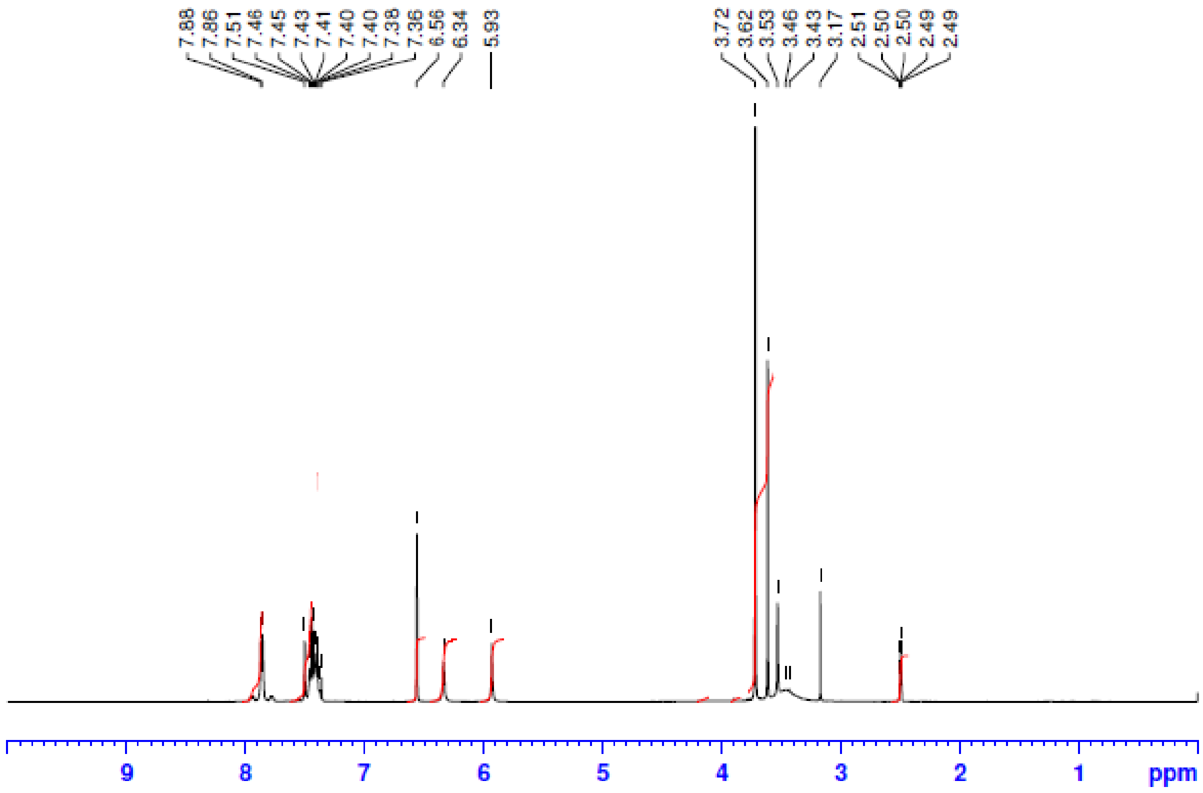

Figure S16. H[1] spectra of Metformin derivative Met3.

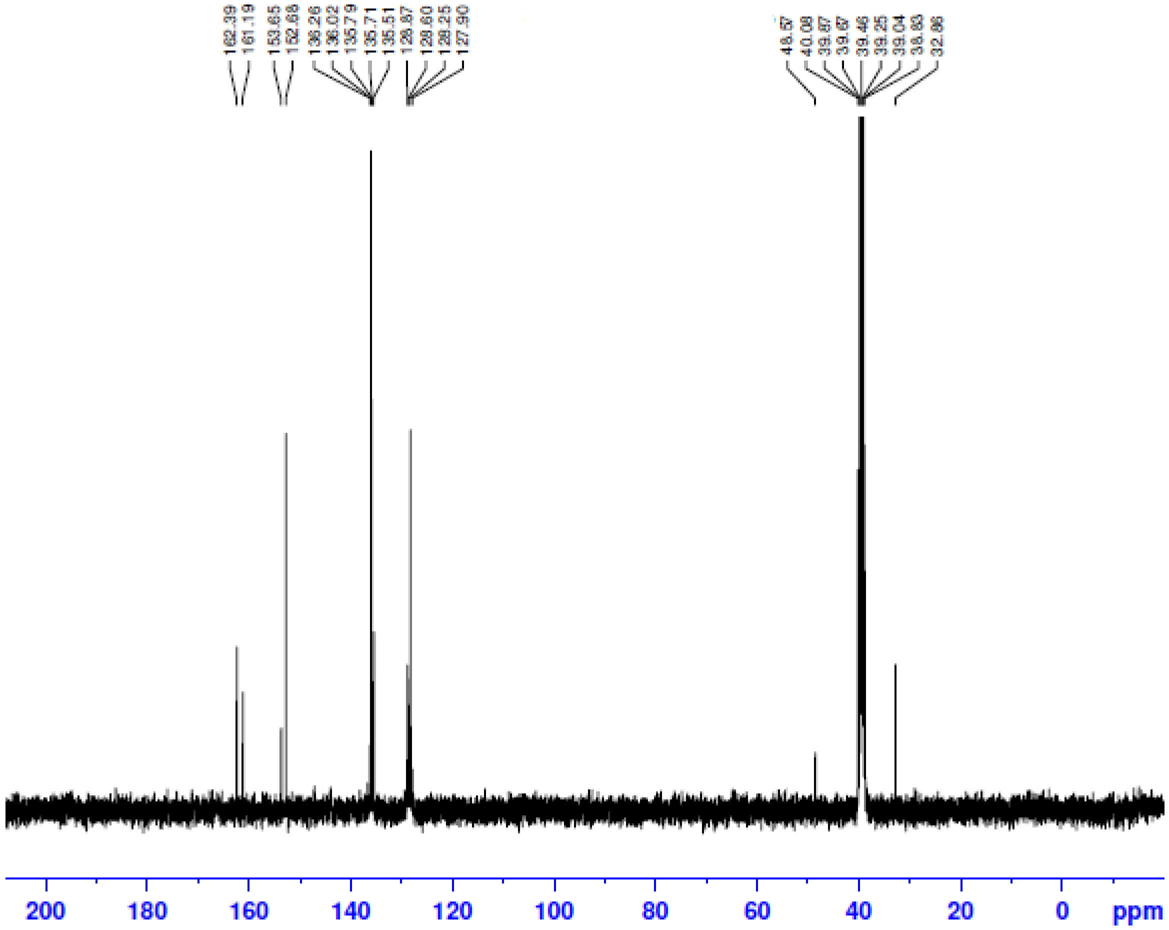

Figure S17. C^{13} spectra of Metformin derivative Met3.

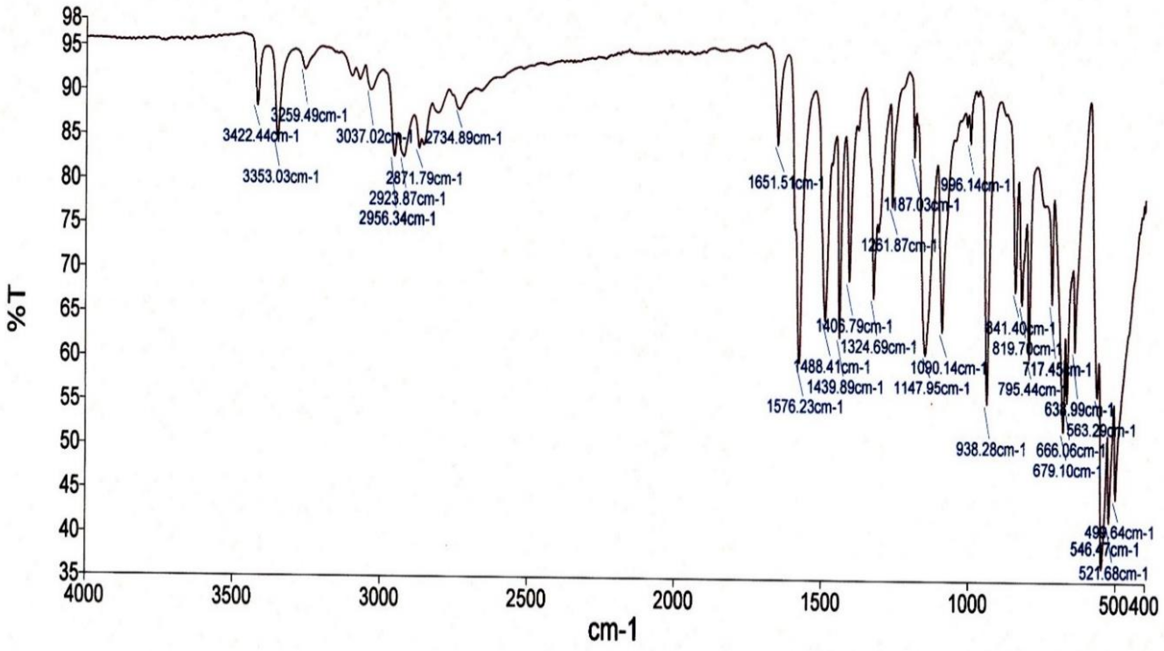

Figure S18. IR spectra of Metformin derivative Met4.

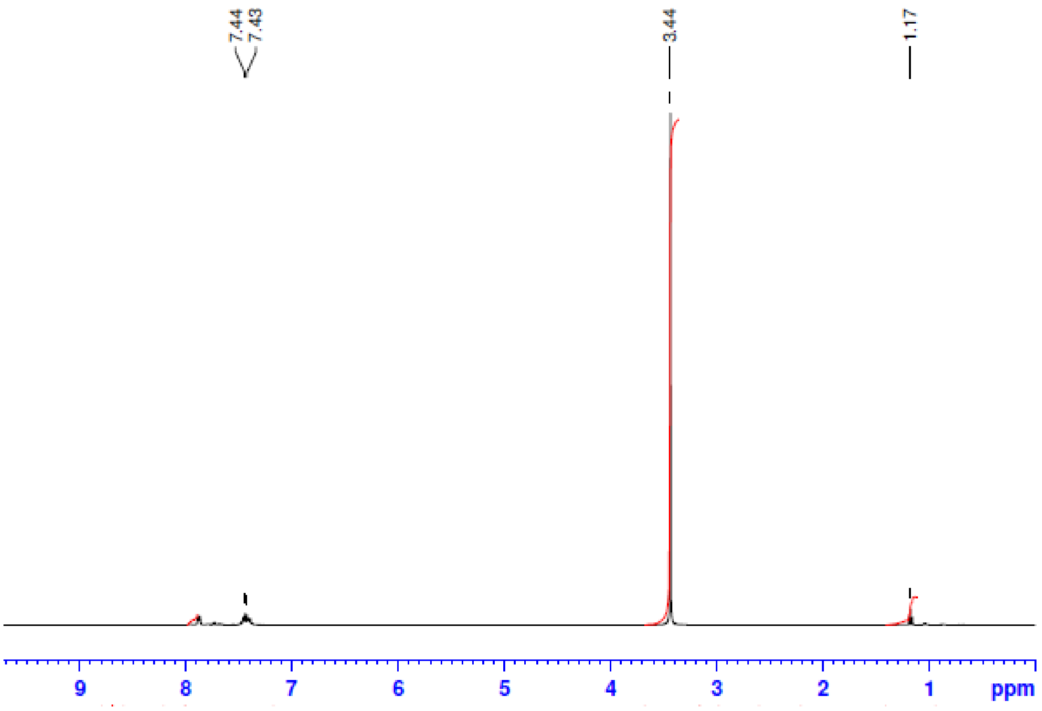

Figure S19. H[1] spectra of Metformin derivative Met4.

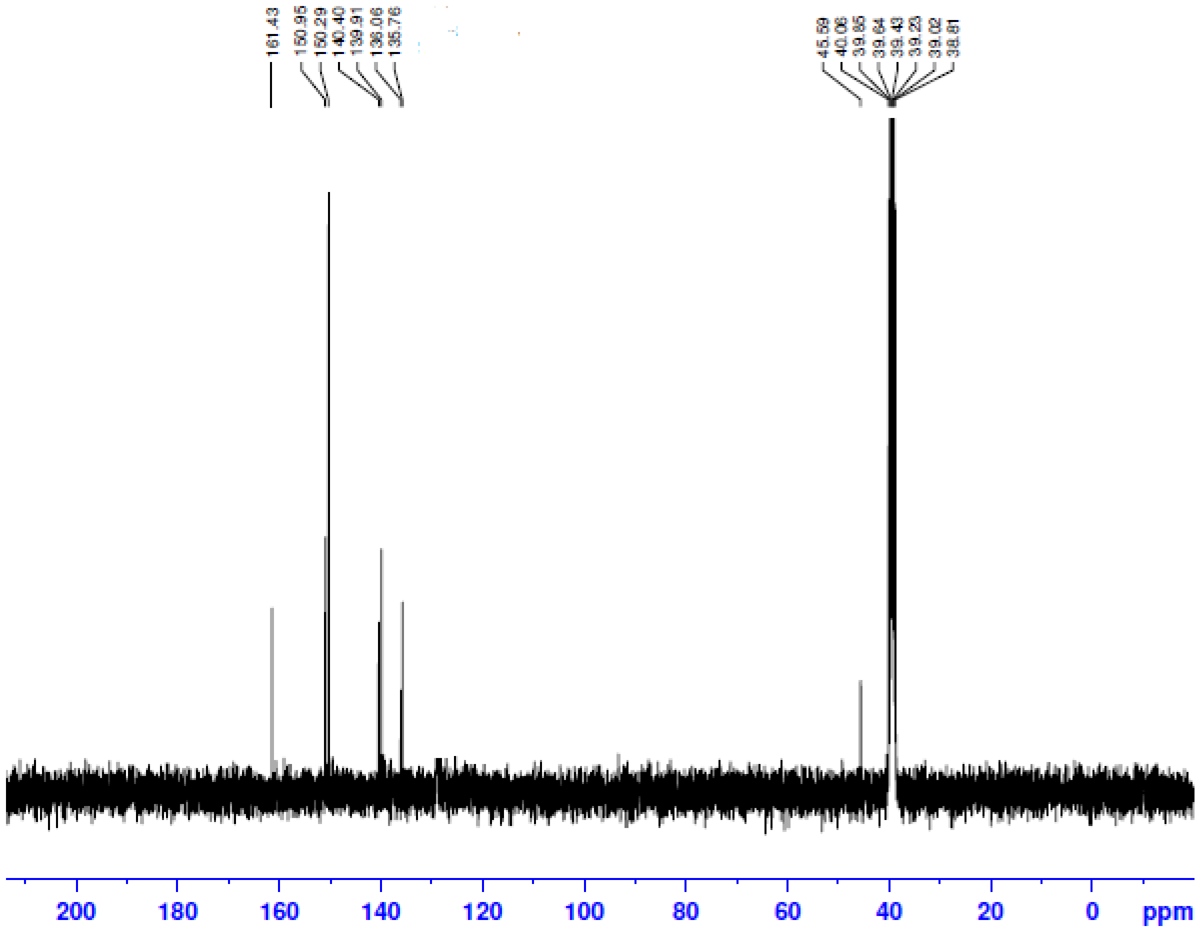

Figure S20. C[13] spectra of Metformin derivative Met4.

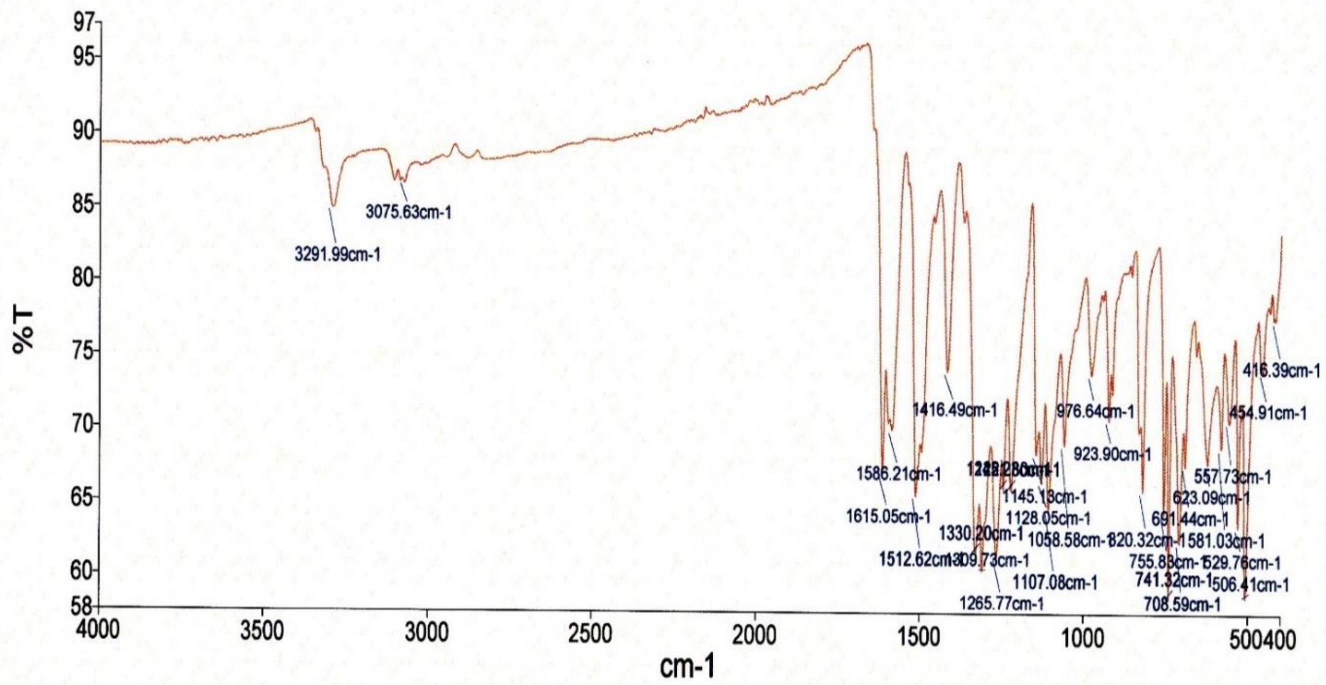

Figure S21. IR spectra of Metformin derivative Met5.

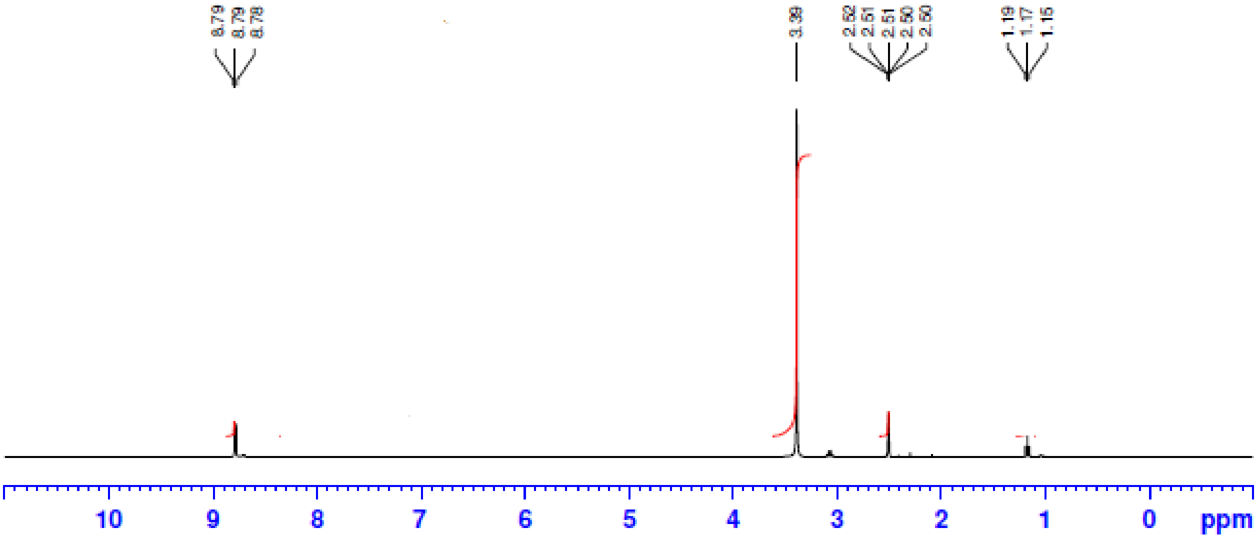

Figure S22. H[1] spectra of Metformin derivative Met5.

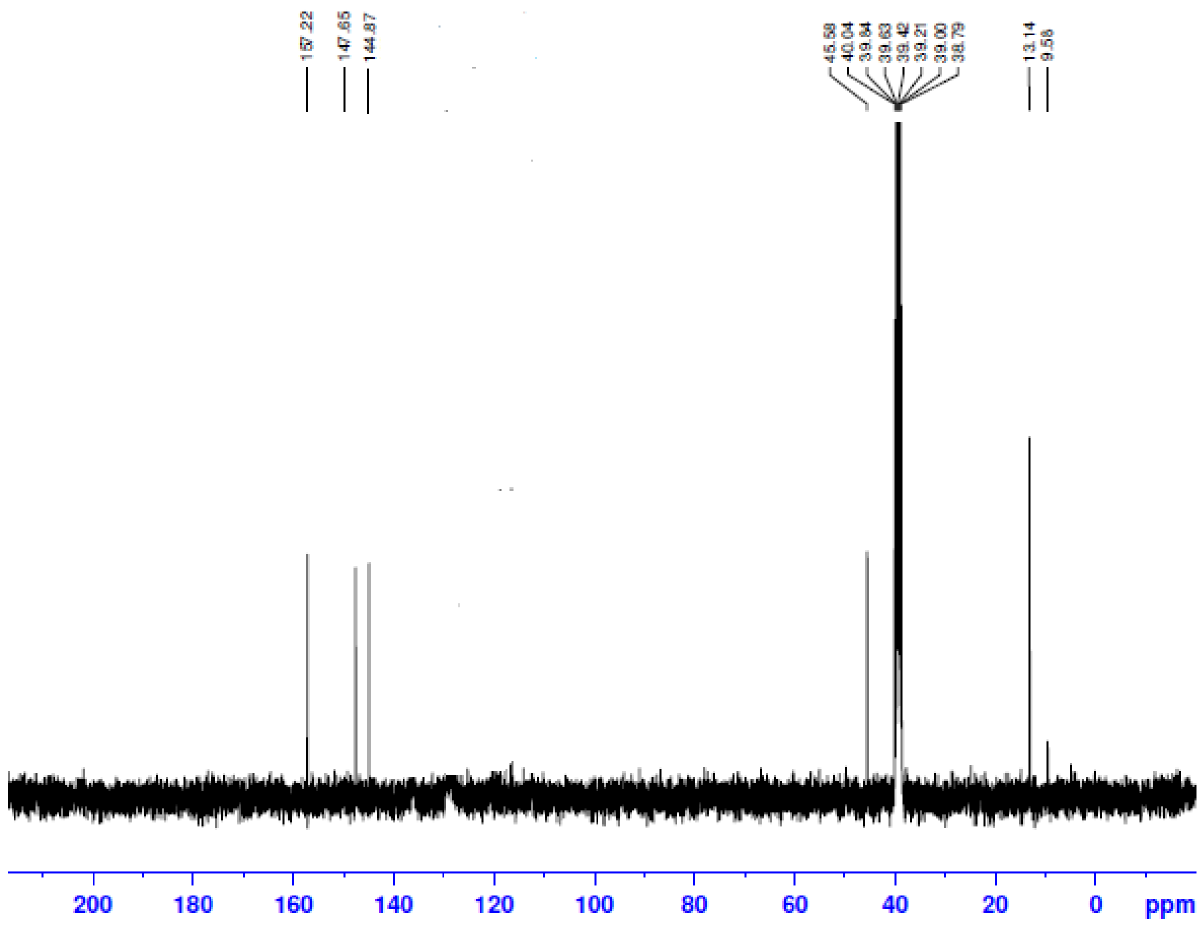

Figure S23. C^{13} spectra of Metformin derivative Met5.

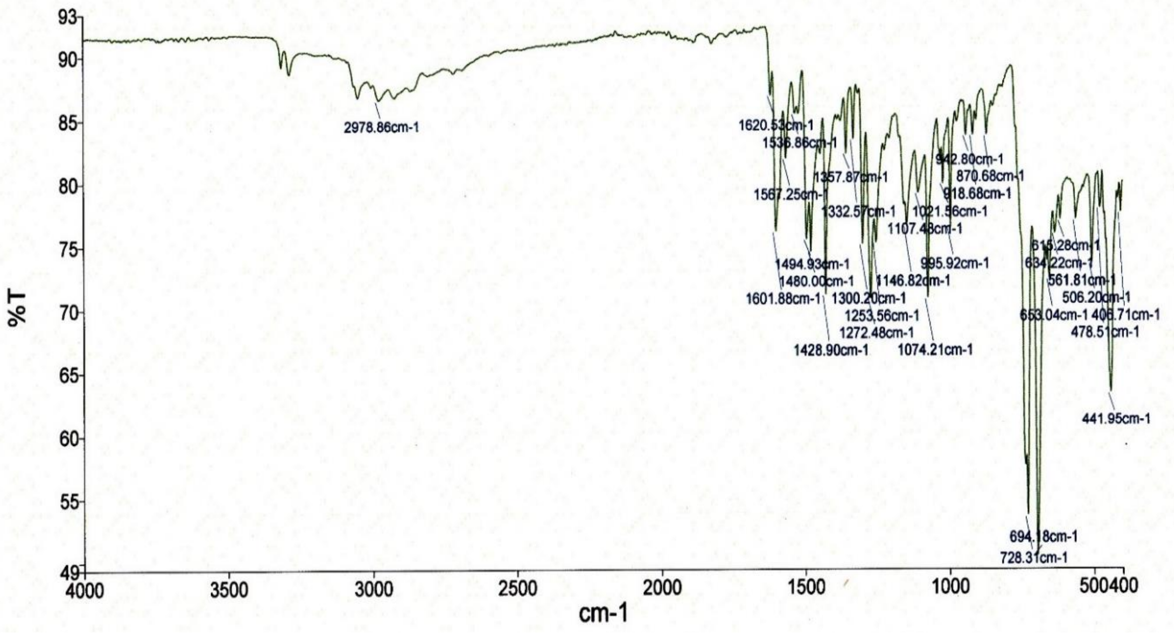

Figure S24. IR spectra of Metformin derivative Met6.

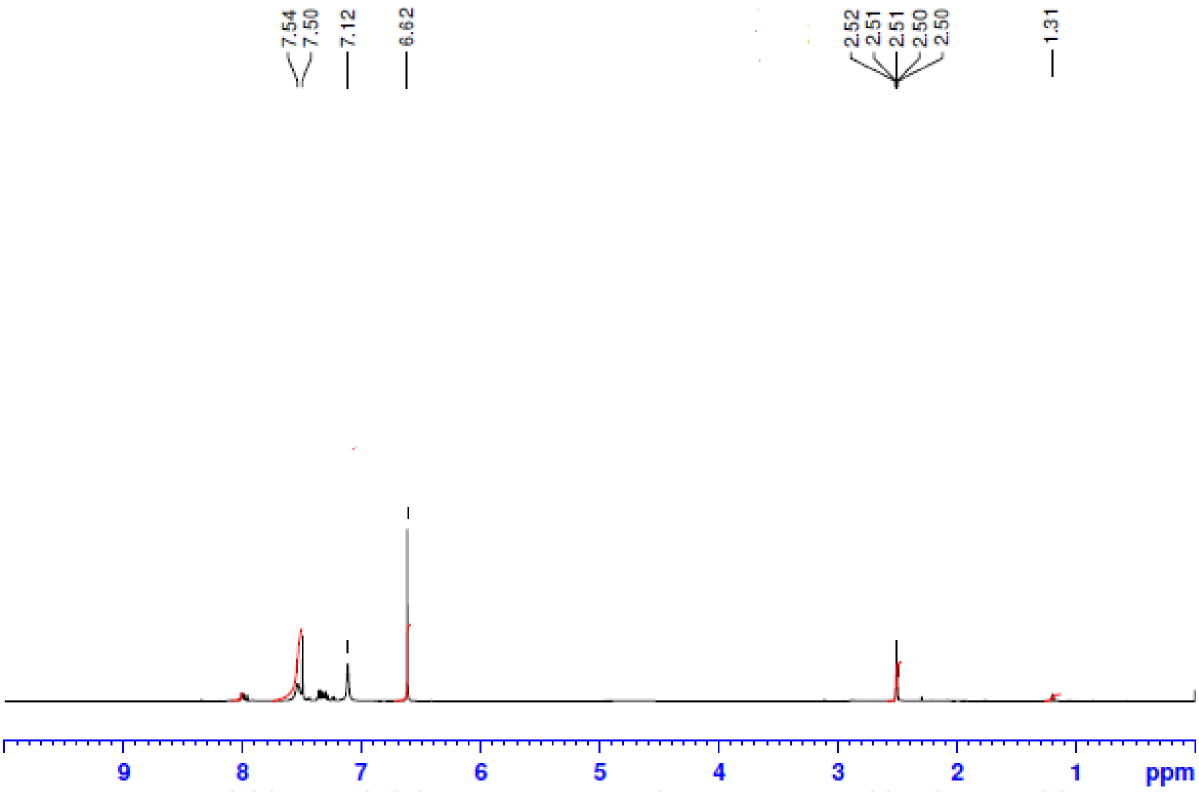

Figure S25. H^1 spectra of Metformin derivative Met6.

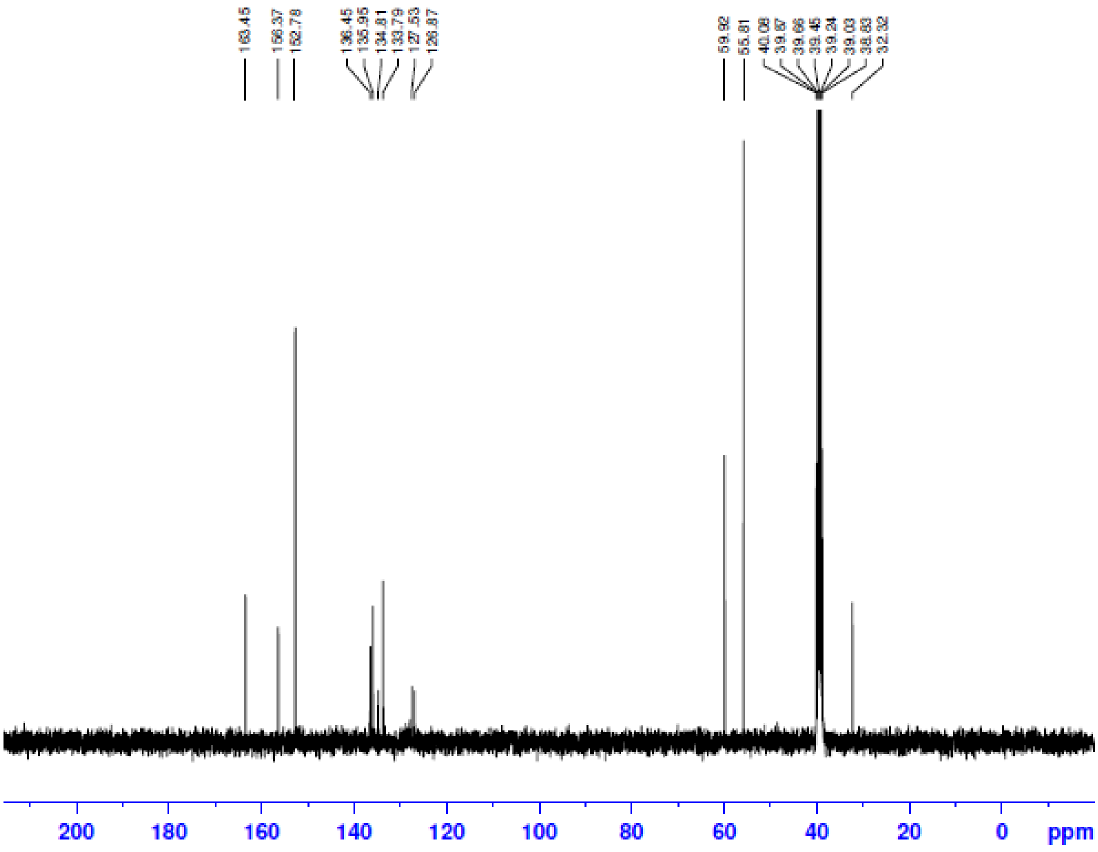

Figure S26. C^{13} spectra of Metformin derivative Met6.

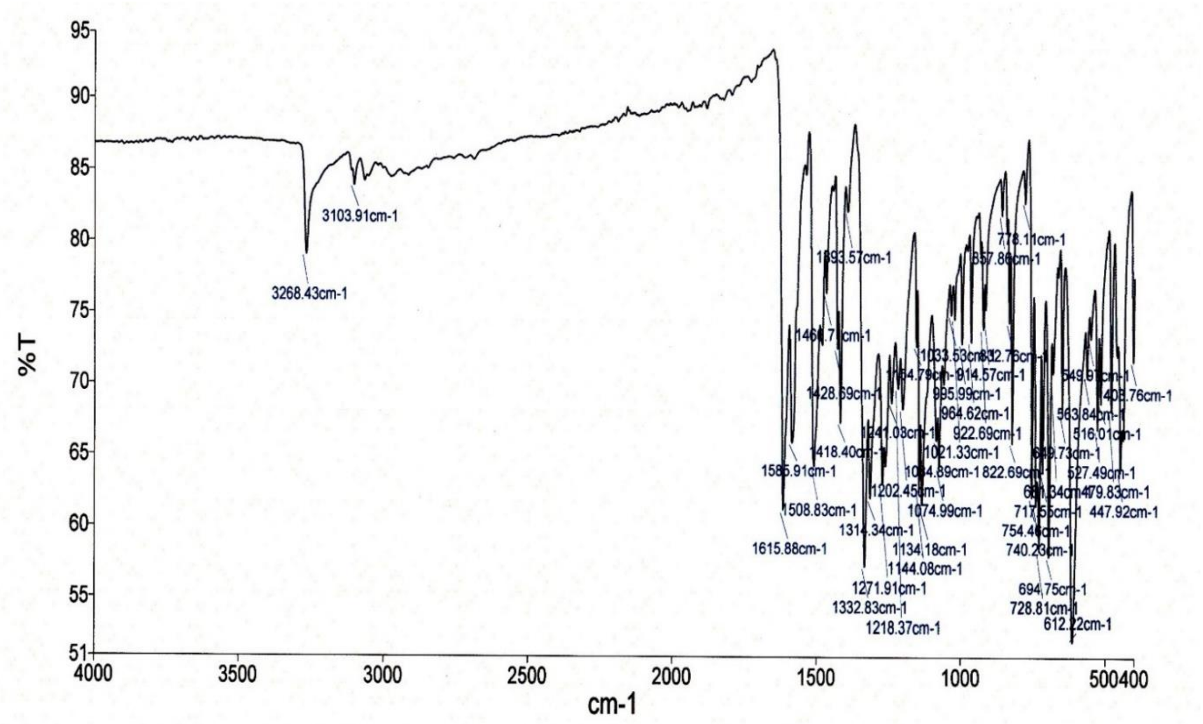

Figure S27. IR spectra of Metformin derivative Met7.

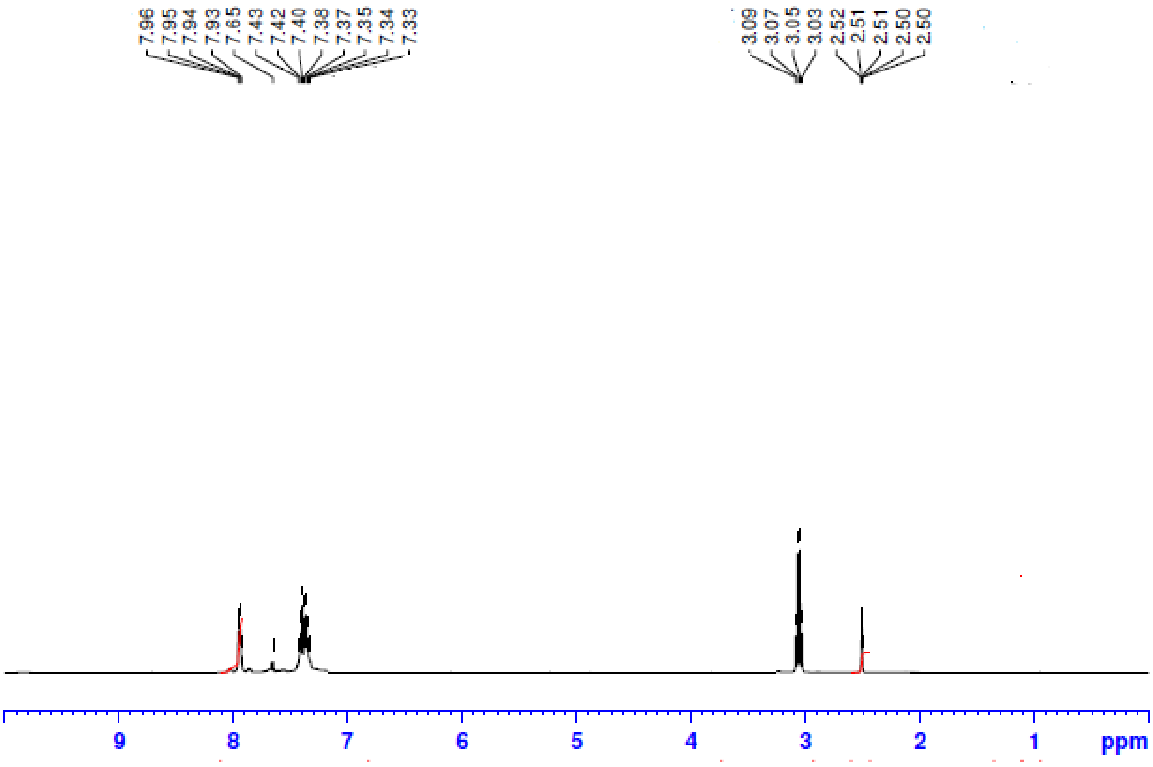

Figure S28. H[1] spectra of Metformin derivative Met7.

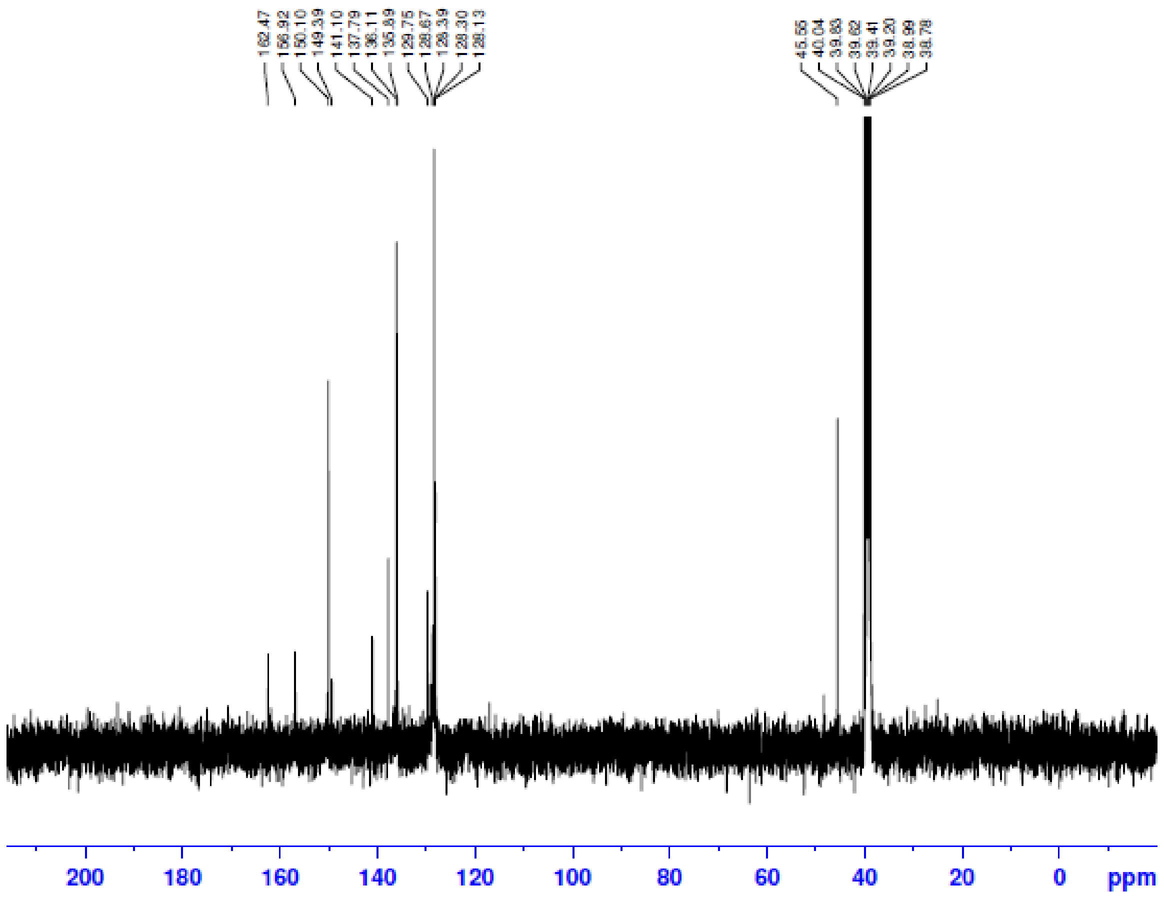

Figure S29. C^{13} spectra of Metformin derivative Met7.

Met 8;

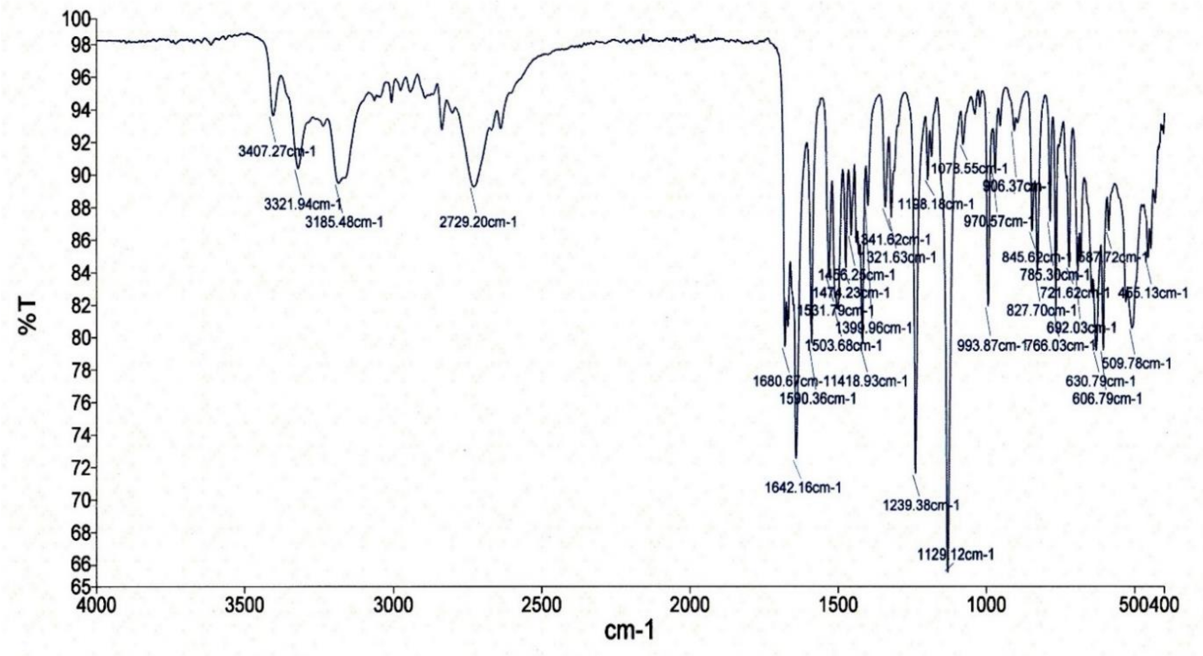

Figure S30. IR spectra of Metformin derivative Met8.

Met 9;

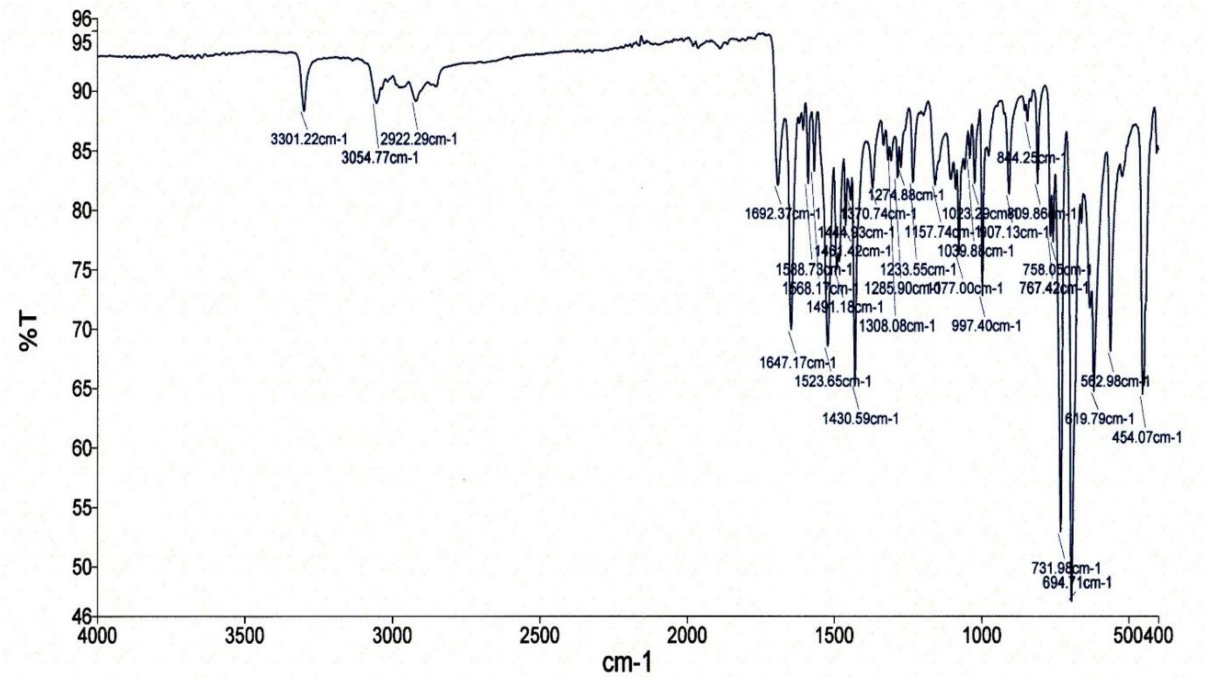

Figure S31. IR spectra of Metformin derivative Met9.
